# Community science designed ribosomes with beneficial phenotypes

**DOI:** 10.1038/s41467-023-35827-3

**Published:** 2023-02-21

**Authors:** Antje Krüger, Andrew M. Watkins, Roger Wellington-Oguri, Jonathan Romano, Camila Kofman, Alysse DeFoe, Yejun Kim, Jeff Anderson-Lee, Eli Fisker, Jill Townley, Eterna Participants, Anne E. d’Aquino, Rhiju Das, Michael C. Jewett

**Affiliations:** 1 Department of Chemical and Biological Engineering, Chemistry of Life Processes Institute, and Center for Synthetic Biology, Northwestern University, Evanston, IL 60208 USA; 2 Department of Biochemistry, Stanford University, Stanford, CA 94305 USA; 3 Eterna Massive Open Laboratory, Stanford, CA 94305 USA; 4 Department of Computer Science and Engineering, State University of New York at Buffalo, Buffalo, NY 14260 USA; 5 Howard Hughes Medical Institute, Stanford University, Stanford, CA 94305 USA; 6 Robert H. Lurie Comprehensive Cancer Center and Simpson Querrey Institute, Northwestern University, Chicago, IL 60611 USA; 7 Present Address: Resilience US Inc, 9310 Athena Circle, La Jolla, CA 92037 USA; 8 Present Address: Prescient Design, Genentech, 1 DNA Way, South San Francisco, CA 94080 USA

**Keywords:** Molecular biology, Synthetic biology

## Abstract

Functional design of ribosomes with mutant ribosomal RNA (rRNA) can expand opportunities for understanding molecular translation, building cells from the bottom-up, and engineering ribosomes with altered capabilities. However, such efforts are hampered by cell viability constraints, an enormous combinatorial sequence space, and limitations on large-scale, 3D design of RNA structures and functions. To address these challenges, we develop an integrated community science and experimental screening approach for rational design of ribosomes. This approach couples Eterna, an online video game that crowdsources RNA sequence design to community scientists in the form of puzzles, with in vitro ribosome synthesis, assembly, and translation in multiple design-build-test-learn cycles. We apply our framework to discover mutant rRNA sequences that improve protein synthesis in vitro and cell growth in vivo, relative to wild type ribosomes, under diverse environmental conditions. This work provides insights into rRNA sequence-function relationships and has implications for synthetic biology.

## Abstract (web summary)

While the ribosome has been harnessed for synthetic biology, designing ribosomes has remained challenging. Here, the authors demonstrate a community science approach for rational design of ribosomes with beneficial properties.

## Introduction

The bacterial ribosome is composed of three distinct ribosomal RNAs (rRNAs) and more than 50 ribosomal proteins (rProteins) separated into a small (30S) and large (50S) subunit^1–3^. The ribosome is responsible for the molecular translation of genetic templates into sequence-defined polymers of amino acids (i.e., proteins), with rRNA components facilitating messenger RNA (mRNA) decoding, accommodating amino acid substrates, catalyzing peptide bond formation, and excreting proteins from the exit tunnel. Motivated by the ribosome’s central role in controlling molecular translation, efforts are expanding to redesign, rebuild, and repurpose ribosomes to facilitate understanding of ribosome assembly and function^2,4–7^, fill knowledge gaps in the origins of life^8–11^, and advance biotechnology^12–16^.

Efforts to modify the ribosome typically focus on creating rRNA mutants with assigned defects, enhanced functions, or altered capabilities. However, several bottlenecks have made altering natural rRNA difficult. First, because the ribosome’s function is necessary for life and many mutations are dominantly lethal^17–22^, cell viability constrains the rRNA mutations that can be made. Cell-free approaches offer an alternative strategy, but cell-free built bacterial ribosomes, such as those from *Escherichia coli (E. coli)*, are not as active as wild type (WT) ribosomes assembled in vivo^8,23,24^. Second, the mutational space is massive. For example, the 1542-nucleotide long 16S and 2904-nucleotide long 23S rRNAs of the *E. coli* ribosome are critical for function; thus, the theoretical sequence space for rRNA mutation is intractable to study experimentally. Third, the ribosome’s shape, physiochemical, and dynamic properties have evolved to build proteins with 20 canonical α-amino acids, making redesign non-trivial^25^. Taken together, these features have resulted in a limited understanding of how to rationally design the structure and function of the rRNA that makes up the ribosome.

Community science has emerged as an approach to rationally design RNA structures and functions^26^. This approach has advantages over typical computational RNA design methods, which are thwarted by the non-polynomial scaling of design methods that produce a sequence with a desired minimum free energy RNA secondary structure^27^. Established in 2010, Eterna is an internet-scale community science game in which community scientists from around the world (players with an interest in science, helping humanity, and/or puzzle solving) ‘solve’ RNA secondary structure design puzzles subject to the constraints imposed by state-of-the-art thermodynamic energy models. In ‘lab challenges,’ players who have completed Eterna’s progression of game-teaching RNA tutorial puzzles for new users submit RNA puzzle ‘solutions’ and select a subset of these by voting, which then is synthesized and tested in research labs. Players share design strategies and help each other understand the game *via* in-game chat as well as in an online forum. Previously, Eterna has successfully targeted RNA design challenges inaccessible to other algorithms^28^. Despite recent progress in community science applied to RNAs and methods for computational design of 3D RNA structure and function^29,30^, no RNA design efforts to date have approached the size or complexity of the entire bacterial ribosome.

Here, we developed a design-build-test-learn (DBTL) approach, implemented through the Eterna platform, to create mutant bacterial ribosomes with improved rRNA secondary structure energetics. Specifically, our approach connects community scientists (Eterna players), university scientists, and game developers through three synergistic, interlocking, and mutually reinforcing DBTL cycles: game developers create 16S and 23S rRNA puzzles according to the needs of community scientists, community scientists design mutant 16S and 23S rRNAs by utilizing community and expert knowledge, and university scientists test these mutants by assessing them in an in vitro ribosome synthesis, assembly, and translation (iSAT) platform^8^. We demonstrate the power of our approach by conducting two iterations of a DBTL pipeline connecting three rounds of community science-derived ribosome design with the goal of improving protein expression in the iSAT cell-free platform. Through the course of this process, Eterna players exceeded state-of-the-art computational prediction and designed mutant ribosomes with up to 42 ± 10% greater protein expression than WT ribosomes under optimal conditions and across diverse stress conditions in vitro. Surprisingly, the mutant ribosomes also support life and enable improved growth compared to cells grown exclusively with WT ribosomes. We anticipate that our Eterna-based DBTL approach will be valuable for engineering complex RNA machines, advancing our knowledge about RNA sequence-folding-function relationships, and inspiring new directions to engineer ribosomes for synthetic biology applications.

## Results

### Eterna players design ribosomes that outperform computationally predicted designs

**Fig. 1:**
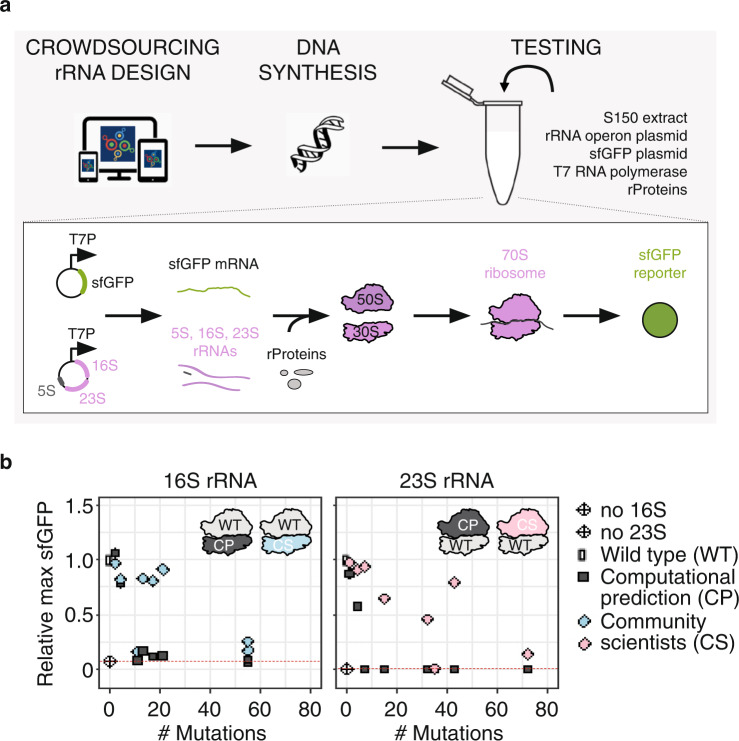
Crowdsourcing rRNA design enables functional mutant ribosomes and outperforms computational predictions. **a** rRNA design was crowdsourced to community scientists (CS) *via* the Eterna platform. In a “pilot round” (R0), community scientists solved 16S rRNA and 23S rRNA puzzles, submitted their “solutions” alongside a description, and voted on their favorite eight designs to be tested for each rRNA. The eight designs for each rRNA with the highest number of votes were synthesized and tested for activity in vitro by integrated ribosome synthesis, assembly, and translation (iSAT). **b** Comparison of sfGFP expression in iSAT reactions with pT7-rrnB-16S and pT7-rrnB-23S rRNA variants generated either by computational prediction (CP) or by community scientists. sfGFP expression was determined by fluorescence over 16 h and normalized to the maximum sfGFP of pT7-rrnB-wild type (WT). Data are shown as mean ± s.d.; *n* ≥ 3. Source data are provided as a Source Data file. The dotted red line indicates the background activity of the S150 extract due to residual ribosomal subunits.

We aimed to explore the use of the Eterna platform to crowdsource the design of functional 16S and 23S rRNA variants with beneficial phenotypes, as compared to the WT *E. coli* sequences. First, we carried out a “pilot round” (R0) of puzzles on the Eterna platform for the ribosome’s 16S and 23S rRNAs using a DBTL framework (Fig. 1a). In the Design phase, the Eterna platform released 16S and 23S rRNA puzzles, and asked players to provide eight mutant 16S rRNA and eight mutant 23S rRNA designs that have improved secondary structure energetics compared to the WT sequence while still being functional. Players designed mutant rRNA sequences by exchanging individual nucleotides against any other unmodified RNA nucleotide (A, C, G, U) in the puzzles, with some critical nucleotides “locked” to their WT identities (see Methods). The sequences were scored with a folding engine calculating the free energy (∆*G*) of the sequence’s secondary structure (see Methods, “Design of ribosome puzzles”). Players could discuss the challenge and exchange material, e.g., worksheets of mutants and their secondary structure energetics, resources, and tools they found online (Supplementary Notes), with each other in an online forum, or through comments on the Eterna platform. Until the end of the Design phase (15 weeks), each player could submit designs alongside a description and design details. At the end of the Design phase, players voted for their favorite eight designs per rRNA puzzle (their own designs and/or the designs of others). In the Build phase, university scientists synthesized plasmid DNA encoding the sequences of the eight 16S and eight 23S rRNA designs with the highest number of votes from players. In the Test phase, mutant 16S and 23S rRNAs were assessed in an in vitro ribosome construction platform, called iSAT^8,24,31^. iSAT enables one-pot co-activation of rRNA transcription, assembly of rRNA with native rProteins into *E. coli* ribosomes, and the synthesis of functional proteins from these ribosomes in a crude S150 extract lacking native ribosomes (Fig. 1a, bottom). A key feature of this system is the ability to generate ribosomal variants by simply changing the DNA input, which enables rapid screening of rRNA mutations^32,33^. Moreover, because ribosomes assembled in iSAT have lower activity than in vivo-assembled versions^8,23,24^ and there are known inefficiencies with ribosome reconstitution in vitro^8,23^, we hypothesized that iSAT would enable us to identify ribosomes where stabilized rRNAs could lead to improved activity. Finally, in the Learn phase, we shared the results with the players on the Eterna webpage in the form of detailed objective results postings. Players then interpreted and discussed the results with each other in an online forum.

Seventeen players submitted 129 16S rRNA designs and sixteen players submitted 157 23S rRNA designs. The players then voted for their top eight designs for each rRNA. The 16S rRNA designs comprised 2–55 mutations, and 23S rRNA designs harbored 1–72 mutations distributed over the entire rRNA sequences (Source Data file; Supplementary Fig. 1–32). DNA sequences were then synthesized and cloned in place of the corresponding 16S rRNA or 23S rRNA into plasmid pT7-rrnB, which encodes a copy of the *rrnB* operon controlled by the T7 promoter, tested in the iSAT platform, and results were shared with the Eterna community (Fig. 1a). Six of the selected 16S rRNA and seven of the 23S rRNA designs from the players were functional in iSAT, conferring activities between 12 and 96% compared to WT. We compared the iSAT activity of the 16S and 23S rRNA variants designed by community scientists head-to-head with a set of computational prediction designs (see Methods) with exactly matched mutation numbers (Fig. 1b; Supplementary Fig. 33a–e) and subject to the same secondary structure requirements. We found that the community scientists’ designs were able to maintain nearly WT-like performance despite installing >20 mutations (16S rRNA) or even >40 mutations (23S rRNA), while every computational prediction design with more than 4 mutations was inactive in iSAT. While we have previously used iSAT to show that more than 85% of 180 single nucleotide mutations within the ribosome active site possess some functional activity^32^, we were surprised that so many mutations could be designed into active ribosomes at once.

To determine the robustness of the initial Eterna 16S rRNA and 23S rRNA designs, we took advantage of the iSAT platform’s direct access to the reaction environment to perturb the magnesium (Mg^2+^) concentration, which impacts rRNA folding and intrinsic rRNA folding stability^34,35^. For this, we set up iSAT reactions with half of the experimentally optimized Mg^2+^ concentration (3.75 mM instead of 7.5 mM) and compared the activity of community scientist-designed rRNA variants with WT rRNA. While WT ribosome activity was still the highest, with 41% activity compared to optimal iSAT conditions, most community scientists’-designed rRNAs assembled into functional ribosomes at low Mg^2+^ concentration, leading to activities of up to 36% of WT at optimal iSAT conditions (Supplementary Fig. 33f–h). These results show that the community scientists’ rRNA designs, which were implemented based on RNA secondary structure folding energetics, are robust under non-optimal iSAT conditions. In sum, the “pilot round” (R0) allowed us to build a community of Eterna players with an interest in ribosome design challenges, implement puzzles for rRNAs in the Eterna design framework, and realize that community scientists can outperform the computational prediction method used in this study for designing functional 16S and 23S rRNAs by balancing numerous constraints in the design process and selecting the best designs through the voting process.

### Development of a progressive DBTL framework for ribosome design

**Fig. 2:**
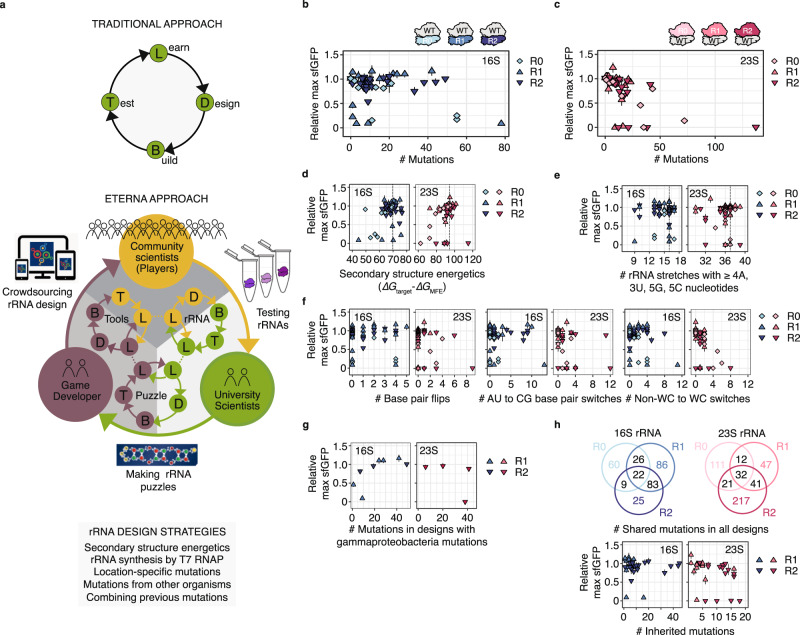
The Eterna approach transforms traditional design-build-test-learn cycles. **a** Our Eterna approach integrates the strengths of community scientists, university scientists, and game developers, who solve RNA design challenges by closely working together. We performed two rounds of our approach. Functionality comparison of Round 1 (R1) and Round 2 (R2) Eterna 16S rRNA **b** and 23S rRNA **c** designs in iSAT with the “pilot round” (R0) in dependence of the designs’ mutation counts. To approach the OpenRibosome Challenge, community scientists followed and combined different strategies: **d** secondary structure energetics, **e** breaking stretches of consecutive identical nucleotides, **f** altering base pairing in rRNA secondary structures, **g** integrating mutations from other gammaproteobacteria, and **h** integrating/ combining mutations from previous rounds. sfGFP expression in iSAT was determined by fluorescence and normalized to the maximum sfGFP of pT7-rrnB-wild type. The dotted line in **d** and **e** indicates the wild type value. Data are shown as mean ± s.d.; n ≥ 3. Source data are provided as a Source Data file. R0: “pilot round”, R1: round 1, R2: round 2, WT: wild type.

Following the “pilot round” (R0), we recognized that continuing our initial DBTL approach would limit the project’s progress. While the size and complexity of the rRNA molecules and the assay used to test them excited Eterna players, they were also overwhelming. The puzzles were difficult to play through the Eterna interface because the algorithm to display their structures would place multiple nucleotides at the same point in 2D space, making it impossible to mutate certain bases. In addition, the feedback available to players was limited to energetics calculated within the puzzles and the experimental results were available months later, limiting the players’ ability to explore diverse solution strategies. To this end, we realized that the Eterna platform itself—from the ribosome puzzles to the analysis tools offered by the game—would require DBTL iteration in parallel and that the same three parties—university scientists, game developers, and community scientists—would have to collaborate to advance an approach in which each of these typically disparate groups would work together to propel the science and gameplay in tandem (Fig. 2a).

To test this framework, we set up a multi-round “OpenRibosome Challenge” on the Eterna platform with the aim of generating further stabilized rRNAs that lead to improved iSAT activity. We improved upon R0 in two notable ways. First, we programmed software for RNA layout (https://github.com/ribokit/RiboDraw)^36^ and composed a diagram of the 16S and 23S rRNAs that reflects the relative position of elements in 3D space to avoid the overlapping rRNA sequences observed in the R0 puzzles. Second, we released constraints over “locked” nucleotides. We made this change because, during R0, we found that the “locked” nucleotides constrained player creativity, and after examining sequence variation across all gammaproteobacteria, we noted that some residues originally deemed immutable were not absolutely conserved. The Eterna players developed an in-game system for tracking which mutations violated gammaproteobacteria sequence conservation (see Methods, “Design of ribosome puzzles”); we anticipated that this system would enable fine-grained feedback, leading to greater freedom to make extensive but high-confidence mutations than was possible with “locked” nucleotides.

We conducted two rounds of the OpenRibosome Challenge (Supplementary Fig. 34a). In Round 1 (R1), a total of 31 players submitted 205 16S rRNA designs and 22 players submitted 161 23S rRNA designs. In Round 2 (R2), a total of 23 players submitted 139 16S rRNA designs and 20 players submitted 113 23S rRNA designs. During each round, the players selected 20 designs, each of 16S rRNA and 23S rRNA, which then were synthesized and tested at optimal iSAT conditions (Source Data file; Supplementary Figs. 35–114). After each round, the data were shared with the community in the form of posts and discussed in forums and community meetings. Players also discussed design strategies (Fig. 2a) in an online forum, occasionally reached out to university scientists for more background information, and agreed to test diverse hypotheses. We found that Eterna players learned from the iSAT data provided and improved their designs over time. For example, player designs in R1 and R2 had higher activity and more mutations than the R0 16S and 23S rRNA designs (Fig. 2b, c; Supplementary Fig. 34b, c).

Several sequence-function player strategies emerged for designing stabilized rRNAs. These included: optimizing secondary structure energetics to support intrinsic rRNA folding, especially in R0 (Fig. 2d), and minimizing sequence repeats (Fig. 2e; Supplementary Fig. 115a). When making mutations, players predominantly flipped base pairs, switched AU to CG pairs and vice versa, and wobble (non-Watson–Crick (non-WC)) base pairs to WC base pairs (Fig. 2f; Supplementary Fig. 115b). Furthermore, guided by scientific literature, players also successfully incorporated mutations from other gammaproteobacteria, with a focus on extremophilic bacteria that are known to have favorable RNA folding capabilities^4,5^ (Fig. 2g). The players used designs from current or previous rounds as inspiration, borrowing and recombining mutations with success (Fig. 2h). In addition, players found it beneficial to avoid exchanging nucleotides that are conserved or that directly contact rProteins^37,38^ (Supplementary Fig. 115c) and they avoided previously described deleterious mutations^21,22,32^.

**Fig. 3:**
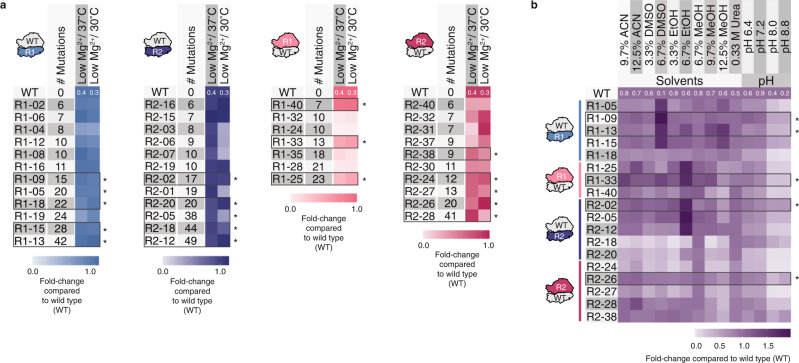
Eterna rRNA designs are robust across diverse stress conditions in vitro. Designs with ≥ 6 mutations and activity of ≥ 80% wild type (WT) at optimal iSAT conditions were tested in diverse iSAT stress conditions. **a** Heatmaps illustrating folding stress tolerance of mutant ribosome designs in iSAT. **b** Heatmap illustrating solvent and pH tolerance of the most diverse and active mutant ribosome designs from R1 and R2 in iSAT. sfGFP expression in iSAT was determined by fluorescence and normalized to the maximum sfGFP of pT7-rrnB-wild type at optimal iSAT conditions. Data are shown as mean; *n* ≥ 3. Source data are provided as a Source Data file. ACN: acetonitrile, DMSO: dimethylsulfoxide, EtOH: ethanol, MeOH: methanol, R1: round 1, R2: round 2, WT: wild type. As a reference, activities of WT at each iSAT stress condition compared to iSAT at optimal conditions and the number of mutations of the most mutated design are provided as white numbers in each heatmap. * selected in **a** as designs for being tested in **b**, in **b** as designs with high iSAT activity in multiple stress conditions.

To characterize and identify robust Eterna rRNA designs, we selected all R1 and R2 designs with ≥ 6 mutations that showed activity of ≥ 80% WT at optimal iSAT conditions as a curated set of high-performing, high-sequence diversity ribosomes and tested them under folding stress conditions: low Mg^2+^ (3.75 mM) concentration and optimal temperature (37 °C) or low Mg^2+^ (3.75 mM) concentration and low temperature (30 °C) (Fig. 3a; Supplementary Fig. 116; Source Data file). Several Eterna designs showed robustness to these folding stress conditions, especially the R1 16S rRNA designs.

From this set of 41 designs, we identified the 18 most diverse (i.e., high mutation rate) and robust (i.e., high iSAT activity) designs per rRNA and round. These 18 designs were next tested in iSAT under non-physiological conditions; the presence of organic solvent (ACN: acetonitrile, DMSO: dimethylsulfoxide, MeOH: methanol, EtOH: ethanol) or altered pH (Fig. 3b**;** Supplementary Figs. 117–120). We selected solvent conditions that reduce iSAT activity using WT rRNAs (Supplementary Table 1). Strikingly, several Eterna-designed ribosomes from R1 and R2 exceeded WT ribosome performance, including R1–13 and R1–15 at 9.7% MeOH, R1–09 at 12.5% MeOH, R2–02 at 6.7% EtOH, as well as R1–33 and R2–26 at 12.5% ACN (Fig. 3b; Supplementary Fig. 117–120). Together, these data highlight the potential for community science to develop ribosomes with beneficial phenotypes, and how computational design might be used to build modified ribosomes with altered chemical properties in the future.

### Combining Eterna rRNA designs increases in vitro ribosome activity

**Fig. 4:**
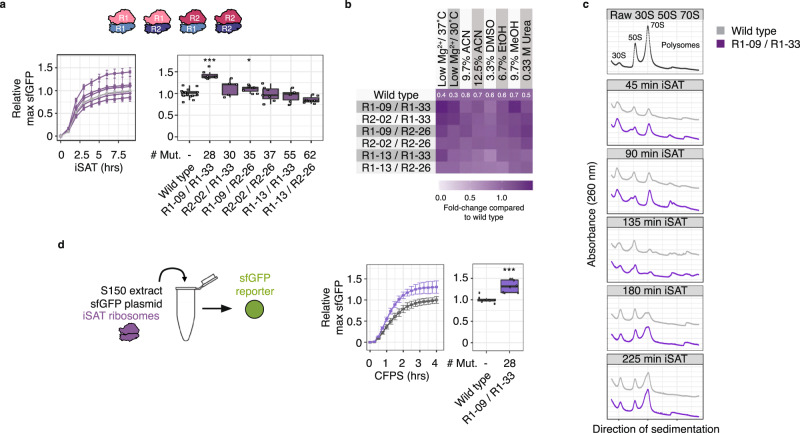
Eterna ribosomes confer beneficial phenotypes in vitro. Two 23S rRNA designs and three 16S rRNA designs with tolerant iSAT stress phenotypes were combined and tested in iSAT under various conditions: **a** optimal conditions, or **b** stress conditions. sfGFP expression in iSAT was determined by fluorescence and normalized to the maximum sfGFP of pT7-rrnB-wild type at optimal iSAT conditions. Fold changes in **b** illustrate iSAT activities normalized to wild type performance under solvent conditions. **c** Assembly of wild type and R1–09/R1-33 ribosomes in iSAT determined *via* ribosome sedimentation analysis. **d** Cell-free sfGFP synthesis in ribosome-free S150 extracts using 1.5 µg purified wild type and R1-09/R1-33 iSAT ribosomes. ACN: acetonitrile, DMSO: dimethylsulfoxide, EtOH: ethanol, MeOH: methanol, R1: round 1, R2: round 2, WT: wild type. Data are shown as boxplots with error bars representing s.d. **a** or mean **b** *n* ≥ 3. Source data are provided as a Source Data file. *p < 0.05 and ***p < 0.001 difference of means vs. wild type, both by two-sample *t*-test.

We next combined Eterna player 16S rRNA and 23S rRNA designs to assess the impact on in vitro ribosome synthesis and activity. We selected three 16S rRNA designs (R1–09, R1–13, R2–02) and two 23S rRNA (R1–33, R2–26) designs covering both rounds. These designs were selected because they had the best combination of sequence diversity, iSAT performance, and tolerance to iSAT stress conditions (Fig. 3). After building plasmids encoding the combined designs, we tested the six combinations (each designed small subunit paired with a designed large subunit) in iSAT reactions (Fig. 4; Supplementary Fig. 121). In iSAT at optimal conditions, the R1–09/R1–33 combination, totaling 28 mutations, showed an activity increase of 42 ± 10% relative to WT (Fig. 4a). This advantage was also observed in several solvent stress conditions (Fig. 4b). Under low Mg^2+^/37 °C and 9.7% MeOH condition, the R1–09/R1–33 combinations also performed about 1.4-times better than WT sequences in sfGFP expression.

We wondered if R1–09/R1–33 design’s improved functionality in vitro arises from the faster assembly in iSAT and/or increased protein synthesis. To investigate these possibilities separately, we first looked at ribosome assembly *via* ribosome sedimentation analysis. We sampled iSAT reactions containing WT or R1–09/R1–33 rRNA designs at different time points, layered them on top of 10–40% sucrose gradients, separated the individual subunits and 70S and polysome fractions *via* ultracentrifugation, and detected their 260 nm traces (Fig. 4c). Compared to WT ribosomes, R1–09/R1–33 showed higher 70S and polysome peaks at time points 45 and 90 min, indicating a faster assembly during iSAT. Next, we determined the cell-free protein synthesis capabilities of WT and R1-09/R1-33 ribosomes in cell-free reactions comparable to iSAT. We purified raw 70S and polysomes from iSAT reactions and tested their ability to translate sfGFP in S150 extracts supplemented with the same additives as in iSAT but without TP70 and pT7-rrnB constructs. Ribosomes containing rRNA design R1–09/R1–33 showed an activity increase of 32 ± 14% compared to WT. These results suggest that the increased in vitro ribosome activity of R1–09/R1–33 most likely results from both improved ribosome assembly and protein synthesis in vitro.

### Community science-designed ribosomes are functional in vivo

**Fig. 5:**
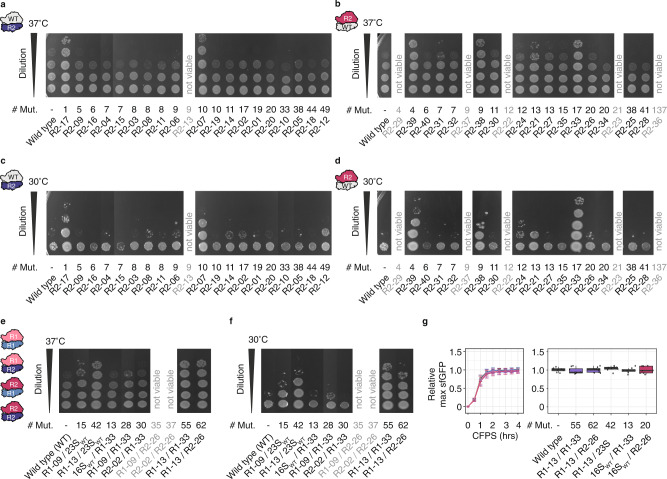
Eterna discovers diverse rRNAs which are functional in vivo. **a**–**f** Spotted SQ171fg cells growing with pL-rrnB-WT and pL-rrnB-R2 16S rRNA (**a**, **c**) 23S rRNA (**b**, **d**), and combinatorial 16S/23S rRNA designs imaged after 24 h at 37 °C (**a**, **b**, **e**) or 72 h at 30 °C (**c**, **d**, **f**). Stationary cells were diluted to an OD600 = 1, diluted stepwise 1:10, and spotted onto LB + Carb_100_ plates. Mut.: mutations, R1: Round 1, R2: Round 2, WT: wild type. Data representative of *n* = 3 independent in vivo growth experiments. **g** Cell-free sfGFP synthesis in ribosome-free S150 extracts using 10 µg purified ribosomes. Data are shown as mean or as boxplots with error bars representing s.d.; n = 8. Source data are provided as a Source Data file.

As highlighted in the introduction, a key challenge of ribosome design is that many rRNA mutants are dominantly lethal^17–22^. Despite this challenge, we wondered if the community science ribosomes designed and tested in vitro could support life. To test this, we individually cloned the R2 and “Combined” designs into the pL-rrnB plasmid, expressing the *rrnB* operon from a temperature-sensitive promoter, pL^39^, and conferring carbenicillin resistance. These plasmids were then individually transformed into the *E. coli* SQ171fg strain^12^, which evolved from the SQ171 strain^40^. The SQ171fg strain lacks chromosomal rRNA alleles and lives on the pCSacB plasmid, which carries an rRNA operon encoding a tethered ribosome, Ribo-T v2^41^, and the tRNA67 plasmid encoding missing tRNA genes. The pCSacB plasmid also contains a counter-selectable marker sacB gene which confers sucrose sensitivity and a kanamycin resistance cassette. Transformed SQ171fg cells were grown in the presence of carbenicillin and sucrose, and individual colonies were picked and tested for loss of kanamycin resistance, indicating loss of plasmid pCSacB-RiboT v2, and resistance to carbenicillin, indicating the presence of the corresponding pL-rrnB designs. Loss of pCSacB-RiboT v2 and presence and accuracy of the pL-rrnB-R2 plasmids was verified by Sanger sequencing. Strikingly, 18 of 20 R2 16S rRNA and 17 of 20 R2 23S rRNA designs support life—of the four designs that are non-functional in iSAT, three were lethal in vivo (R2–22, R2–23, R2–36), and one design with only about 50% iSAT activity (R2–13) harboring a mutation in the central 16S rRNA pseudoknot was lethal as well (Fig. 5a, b). Interestingly, one design is inactive in iSAT, but functional in cells (R2–25); suggesting it may suffer from assembly defects in vitro, which can be compensated for in vivo.

We next investigated how the ribosomes containing Eterna rRNA designs affect growth on solid nutritionally rich (LB) media. For this, we performed spot plating assays (Fig. 5a–d; Supplementary Fig. 121). Most of the R2 and combined 16S and 23S rRNA designs showed growth phenotypes on plates similar to WT, and 6 strains (R2–7, R2–17, R2–21, R2–33, R2–38, R2–39) grew better than WT on plates (Fig. 5a, b; Supplementary Fig. 121a, c). When incubating the plates at sub-optimal temperature (30 °C), the phenotypes were even more pronounced and additional strains showed growth phenotypes (R2–12, R2–24); Fig. 5c, d; Supplementary Fig. 121b, d). We also assessed the functionality of the combinatorial designs in vivo (Fig. 5e, f; Supplementary Fig. 121e, f), and found that, except for two designs (R1–09/R2–26 and R1–13/R2–26), the combined mutant ribosomes also support life. Two highly mutated ribosomes containing 55 and 62 mutations (combinatorial designs R1–13/R1–33 and R1–13/R2–26) show improved growth at 37 and 30 °C. This growth phenotype presumably arises from 16S rRNA design R1–13, which also confers improved growth when combined with WT 23S rRNA. Taken together, our results demonstrate the functionality of Eterna-designed ribosomes both in vitro and in vivo.

Finally, we wondered if the combinatorial designs R1–13/R1–33 and R1–13/R2–26 had altered protein synthesis capabilities and compared the designs’ and their parental designs’ ability to synthesize sfGFP in cell-free protein synthesis reactions (Fig. 5g). We found that cell-free protein synthesis from purified mutant ribosomes was comparable with WT, indicating that the observed phenotypes arise due to more complex biological processes within the cells.

## Discussion

In this study, we present an integrated computational and experimental workflow for constructing mutant ribosomes. This was accomplished by creating a DBTL framework that relies on game developers to create rRNA puzzles for stabilizing secondary structure, community scientists to design rRNA sequences, and university scientists to carry out high-throughput, in vitro reactions to assess ribosome synthesis, assembly, and translation. We applied this pipeline to create mutant ribosomes that are functionally active.

Our work has several important features. First, we found that mutant rRNA sequences can confer beneficial phenotypes. In vitro, we showed that Eterna-designed ribosomes constructed and assessed in iSAT outperformed WT ribosomes in in vitro translation under standard conditions, and were even more tolerant in stress phenotypes (e.g., protein expression in the presence of organic solvents). In vivo, we were surprised to find that more than 85% of designed ribosomes tested could support life, with more than 10% demonstrating improved growth phenotypes in strains that live exclusively off community scientists’-designed ribosomes. Second, our data provided insights into ribosome sequence-to-function relationships. For example, we found that mutations that slightly improve secondary structure energetics, or those that remove base repeats (R2–24), can be well tolerated and that these effects can be amplified when combining mutations from well-performing designs (R2–02, R2–33). We also found that exchanging nucleotides against nucleotides from another gammaproteobacterial rRNA can improve protein translation in vitro (R1–13, R2–26) and growth in vivo (R1–13, R2–12, R2–38) at optimal and low-temperature conditions. Third, we applied crowdsourcing to ribosome design for the first time. Crowdsourcing design presents complex problems to a diverse population of community scientists, resulting in a variety of solutions that, taken together, can avoid trapping by local optima in a fashion reminiscent of classic nonconvex optimization algorithms^42^. For example, in our case, we found that community scientists were able to improve at each stage of the process (from R0 through R2), as the community worked together to infer how to iterate on a family of solutions in light of newly disseminated experimental data. Finally, rules learned from Eterna players could guide the development of future algorithms. Examples of such rules are: (i) avoiding mutations in sequence motifs that are necessary for maintaining rRNA folding and structure, (ii) avoiding mutations of nucleotides involved in important protein contacts, and (iii) incorporating mutations that are based on nucleotides or sequence variations found in gammaproteobacterial rRNA.

Looking forward, we expect that our Eterna-based DBTL approach to crowdsourcing ribosome design will provide a rapid and powerful strategy for developing engineered ribosomes for synthetic and chemical biology. This could be used to deepen our understanding of the ribosome’s RNA-based active site, make simpler ribosomes to fill in knowledge gaps in the origins of life, and tailor the ribosome active site to accommodate non-canonical monomers to yield new classes of enzymes, therapeutics, and materials.

## Methods

### S150 extract preparation

*E. coli* MRE600 cells for S150 extract and TP70 preparation were grown in 2× YPTG at 37 °C until OD600 = 3.0. Cells were pelleted, and washed three times in S150 lysis buffer (20 mM Tris-HCl (pH 7.2 at 4 °C), 100 mM NH_4_Cl, 10 mM MgCl_2_, 0.5 mM EDTA, 2 mM DTT), flash-frozen in liquid nitrogen, and stored at −80 °C. For cell lysis, about 4 g of cells were resuspended in S150 lysis buffer at a ratio of 5 ml of buffer per 1 g of cells and supplemented with 200 µl of Halt Protease Inhibitor Cocktail (Thermo Fisher Scientific Inc.) and 75 µl RNase Inhibitor (Qiagen) per 4 g of cells. The cells were lysed at ∼20,000 psi with an EmulsiFlex-C3 homogenizer (Avestin). The lysate was supplemented with an equivalent dose of RNase Inhibitor and 3 µl of 1 M DTT per ml suspension and clarified twice by centrifugation at 30,000 × *g* and 4 °C for 30 min. The resulting S30 extract was recovered and layered in a 1:1 volumetric ratio on a high sucrose cushion composed of Buffer B (20 mM Tris-HCl (pH 7.2 at 4 °C), 500 mM NH_4_Cl, 10 mM MgCl_2_, 0.5 mM EDTA, 2 mM DTT, 37.7% sucrose) into Ti70 ultracentrifuge tubes. Samples were centrifuged at 90,000 × *g* and 4 °C for 20 h. Clear ribosome pellets were used for TP70 preparation, and supernatants were recovered and spun at 150,000 × *g* and 4 °C for an additional 4 h. The top two-thirds of the supernatants were collected without disturbing the pellet and dialyzed in SnakeSkin dialysis tubing (Thermo Fisher Scientific Inc.; 3.5 kDa MWCO) against 50 volumes of high-salt S150 extract buffer (10 mM Tris-OAc (pH 7.5 at 4 °C), 10 mM Mg(OAc)_2_, 20 mM NH_4_OAc, 30 mM KOAc, 200 mM KGlu, 1 mM spermidine, 1 mM putrescine, 1 mM DTT). Four dialysis steps with fresh dialysis buffer were performed: three steps for 2 h and a final step overnight. Extracts were then clarified at 4000 × *g* for 10 min and concentrated to ~4 mg/mL total protein concentration using 3 kDa molecular weight cutoff (MWCO) Centriprep concentrators (EMD Millipore) to account for dilution during preparation. S150 extract samples were aliquoted, flash-frozen, and stored at −80 °C. Protein concentration was determined using Bradford assay with bovine serum albumin (BioRad) as a standard.

### Total protein of 70S ribosomes (TP70) preparation

Clear ribosome pellets from the S150 extract preparation were washed and resuspended in 10 mM Tris-OAc pH 7.5, 60 mM NH_4_Cl, 7.5 mM Mg(OAc)_2_, 0.5 mM EDTA, and 2 mM DTT. The concentration of resuspended ribosomes was determined from A260 NanoDrop readings (1 A260 unit of the 70S = 24 pmol 70S^43^). Ribosomes were aliquoted, flash-frozen, and stored at −80 °C until further use. To precipitate rRNA, two volumes of glacial acetic acid were added to purified 70S ribosomes in Buffer C (10 mM Tris-OAc (pH 7.5 at 4 °C), 60 mM NH_4_Cl, 7.5 mM Mg(OAc)_2_, 9.5 mM EDTA, 2 mM dithiothreitol (DTT)) with 0.2 mM spermine and 2 mM spermidine, and 100 mM Mg(OAc)_2_. Samples were mixed well and centrifuged at 16,000 × *g* for 30 min to pellet rRNA. Supernatants containing rProteins were collected, mixed with five volumes of chilled acetone, and stored at −20 °C overnight. Precipitated protein was collected by centrifugation at 10,000 × *g* for 30 min, dried, and resuspended in simplified high-salt/ urea buffer (50 mM HEPES (pH 7.6 at RT), 10 mM Mg(Glu)_2_, 200 mM KGlu, 0.5 mM EDTA, 2 mM DTT, 1 mM putrescine, 1 mM spermidine, 6 M urea). The protein sample was then transferred to midi-size 1 kDa MWCO Tube-O-Dialyzers (G-Biosciences) and first dialyzed against 100 volumes of simplified high-salt buffer with urea overnight, then three times against 100 volumes of the simplified high-salt buffer without urea for 90 min each. The dialyzed protein sample was next clarified at 4000 × *g* for 10 min, and the supernatant’s protein concentration was determined from A230 NanoDrop readings (1 A230 unit of TP70 = 240 pmol TP70^39^). TP70 samples were aliquoted, flash-frozen, and stored at −80 °C until use.

### iSAT reactions

iSAT reactions were set up as previously described^8,30,40,41^. Briefly, 6.5 µl reactions were prepared by mixing salts, substrates, cofactors, and additives (7.5 mM Mg(Glu)_2_, 167 mM K(Glu), 1.2 mM ATP, 0.85 mM GTP, 0.85 mM UTP, 0.85 mM CTP, 0.034 mg/ml folinic acid, 0.1706 mg/ml tRNAs, 0.33 mM NAD, 0.27 mM CoA, 4 mM oxalic acid, 1 mM putrescine, 1.5 mM spermidine, 57 mM HEPES, 3 mM amino acids, 42 mM PEP, 4% PEG8000, 2 mM DTT), with 4 nM sfGFP reporter plasmid, 4 nM pT7rrnB construct, 60 µg/ml T7 RNA polymerase, 200 nM TP70, and S150 extract. Reactions were incubated in 384-well plates (Greiner, catalog number 781096) at 37 °C in a BioTek Synergy H1 plate reader, and fluorescence of superfolder GFP (sfGFP) was monitored (excitation: 450–490 nm, emission: 510–530 nm) over the course of the reaction.

### iSAT plasmid construction

DNA templates of 16S and 23S rRNAs designed by community scientists (Eterna designs) or computationally predicted were synthesized by Twist Biosciences as clonal genes exchanged against WT 16S rRNA and 23S rRNA of the 7311-bp plasmid pT7rrnB carrying the *E. coli* *rrnB* operon under the control of the T7 promoter and the β-lactamase resistance gene as a selective marker (Supplementary Methods – Plasmid sequences).

### Plasmid construction for in vivo tests

DNA templates of 16S and 23S rRNA round 2 Eterna designs were synthesized by Twist Biosciences and exchanged against WT 16S rRNA or mutant 23S rRNA A2058G of the 7415-bp plasmid pLrrnB carrying the *E. coli rrnB* operon under the control of the pL-G-12T promoter^43^ and the β-lactamase resistance gene as a selective marker (Supplemental Information—Plasmid sequences). Constructs carrying the 16S rRNA Eterna designs, therefore, harbored the A2058G point mutation in the 23S. This point mutation was corrected back to WT by site-directed mutagenesis using Q5® Hot Start High-Fidelity DNA Polymerase (New England Biolabs) and primers: ACGGAAAGACCCCGTGAACC and CTTGCCGCGGGTACACTGC. Linear PCR products were purified using DNA Clean and Concentrator-5 kit (Zymo Research), DpnI digested, phosphorylated by T4 PNK (New England Biolabs), blunt-end ligated using T4 ligase (New England Biolabs), transformed into 50 μL of electrocompetent POP cells, recovered in 800 µL SOC media, plated onto LB-agar/ carbenicillin plates and grown at 30 °C. Clones were picked, streaked out onto fresh LB-agar/carbenicillin plates, and grown overnight in 3 ml of LB/carbenicillin media at 30 °C. Plasmids were prepped (Zymo Research), and *rrnB* operons were sequence-verified using Sanger sequencing (Northwestern University Sanger Sequencing Facility).

### Replacement of Ribo-Tv2 by Eterna-pLrrnB plasmid in SQ171fg cells

SQ171fg cells harboring the Ribo-Tv2 plasmid containing the kanamycin resistance gene as a selection marker were transformed with pLrrnB plasmids carrying the 16S and 23S rRNA Eterna designs and the β-lactamase resistance gene. In brief, 20–100 ng of an Eterna-pLrrnB plasmid was transformed into 50 μL of electrocompetent cells. Cells were resuspended in 850 μL of SOC media and incubated for 1 h at 37 °C with shaking. 250 μL of recovering cells were transferred to 1.75 ml of SOC containing 50 μg/ ml of carbenicillin and 0.25% sucrose (final concentrations) and grown for 16–18 h at 37 °C with shaking. Cells were pelleted and plated on LB-agar plates containing 50 μg/ml carbenicillin and 5% sucrose. Colonies were tested for loss of the original Ribo-Tv2 plasmid and containment of the desired pLrrnB plasmid by selecting clones only living on LB-agar/carbenicillin plates, but not on LB-agar/kanamycin plates. The presence and correctness of the Eterna-design *rrnB* operon in identified clones were Sanger sequence-verified.

### Spotting assay

Eterna-pLrrnB-containing SQ171fg cells were grown overnight in 3 mL of LB media containing 75 μg/ml of carbenicillin. The cultures were diluted to OD600 = 1, 0.1, 0.01, 0.001, and 0.0001 with water. 3 µl of the dilutions were spotted onto LB-agar/100 µg/ml carbenicillin plates and grown for 24 h at 37 °C or 72 h at 30 °C.

### Ribosome sedimentation analysis

For each iSAT timepoint, 3 × 15 µl iSAT reactions were prepared, incubated at 37 °C, pooled together, and quenched by flash-freezing in liquid nitrogen. Sucrose gradients were prepared from gradient buffer (20 mM Tris–HCl (pH 7.5 at 4 °C), 100 mM NH4Cl, 10 mM MgCl_2_) with 10 and 40% sucrose in SW41 polyclear centrifuge tubes (Seton Scientific) using a Biocomp Gradient Master and chilled to 4 °C. The pooled iSAT reactions were thawed on ice, diluted with gradient buffer to 300 µl, layered on the gradients, and ultra-centrifuged at 288,000 × *g* (41,000 × rpm) for 3 h at 4 °C using an Optima L-80 XP ultracentrifuge (Beckman-Coulter) at maximum acceleration and braking. Gradients were analyzed with a Piston Gradient Fractionator^TM^ (Biocomp) coupled to a Triax^TM^ FC-2 UV-260/280 flow cell (Biocomp).

### Cell-free protein synthesis (CFPS) using purified ribosomes

To test ribosome designs supporting life in cell-free protein synthesis, overnight cultures were diluted to OD600 = 0.05 in 750 mL fresh LB media and grown at 37 °C with shaking to the mid-exponential phase (OD600 = 0.5–0.9). Cells were pelleted, washed with lysis buffer (20 mM Tris-HCl (pH 7.2 at 4 °C), 100 mM NH_4_Cl, 10 mM MgCl_2_, 0.5 mM EDTA, 2 mM DTT), flash-frozen in liquid nitrogen and stored until further use at −80 °C. Cell pellets were thawed on ice, resuspended in lysis buffer at a ratio of 5 ml of buffer per 1 g of cells, lysed at ∼24,000 psi with an EmulsiFlex-B15 homogenizer (Avestin), and lysates clarified twice by centrifugation at 12,000 × *g* and 4 °C for 30 min. The lysates were layered in a 1:1 volumetric ratio on a high sucrose cushion composed of Buffer B into Ti70 ultracentrifuge tubes. Samples were centrifuged at 90,000 × *g* and 4 °C for 18 h. Clear ribosome pellets were dissolved in 10 mM Tris-OAc pH 7.5, 60 mM NH_4_Cl, 7.5 mM Mg(OAc)_2_, 0.5 mM EDTA, and 2 mM DTT, flash-frozen in liquid nitrogen and stored at −80 °C until further use.

To test iSAT ribosomes in cell-free protein synthesis, sixteen 15 µL iSAT reactions were incubated for 2 h at 37 °C, pooled together, flash-frozen in liquid nitrogen, and stored at −80 °C until further use. iSAT reactions were layered on a high sucrose cushion composed of Buffer B into Ti70 ultracentrifuge tubes and centrifuged at 90,000 × *g* and 4 °C for 18 h. Clear ribosome pellets were dissolved as described above, flash-frozen in liquid nitrogen and stored at −80 °C until further use.

Cell-free protein synthesis reactions were performed as described for iSAT reactions with the following modifications: instead of pT7rrnB construct and TP70, 1.5 µg purified iSAT ribosomes or 10 µg in vivo-built ribosomes were added.

### Computational prediction

In order to provide a realistic comparison for the designs from the Eterna “pilot round” (R0), a Python script was used to launch hundreds of Metropolis criterion Monte Carlo trajectories starting from the WT sequence for the 16S and 23S rRNAs. These trajectories attempted to make mutations to the rRNA that would minimize the difference in energy between the “delta”—the energy of the sequence’s minimum free energy structure in the Vienna2 engine and the energy of the “target” structure used in the corresponding “pilot round” (R0) puzzle. Moves reducing “delta” were accepted unconditionally, while moves increasing “delta” were accepted conditionally (if they passed the Metropolis criterion). Following these trajectories, the resulting set of sequences were taken together and for each mutation count found in an Eterna design, the computational prediction sequence with the lowest “delta” was selected. Scripts to carry out these simulations are included at https://github.com/everyday847/vienna_guided_mc^44^. In total, 8544 sequences were generated for the 23S rRNA, and 40,689 sequences were generated for the 16S rRNA. To attempt a fair comparison, for each mutation count observed among the community scientists’ sequences, we selected the computational prediction sequence with that number of mutations exhibiting the best Vienna2 secondary structure energy for the stipulated subunit secondary structure.

### Design of ribosome puzzles

The initial “pilot round” (R0) ribosome puzzles were created with the conventional tools already available in Eterna, which permitted the specification of a sequence and secondary structure, an energy model, and some design restrictions (i.e., that certain bases could not be mutated (i.e., “locked”) and that at most a certain number of mutations would be tolerated).

For that round, we prompted players to minimize the energy gap for each rRNA sequence between its experimental secondary structure and its minimal free energy (MFE) secondary structure. So that players could receive real-time feedback on their design progress, we created 16S and 23S rRNA puzzles and equipped them with the LinearFold implementation of the Vienna2 energy model^45^. As a precaution, we limited the total number of mutations to 5% of the 16S and 23S rRNA (76 and 145 mutations, respectively). We additionally prohibited 40 nucleotides in the 16S rRNA and 70 nucleotides in the 23S rRNA from being mutated; these “locked” bases included contacts with rProteins, tertiary contacts within one ribosomal subunit or across both subunits, or they formed base pairs that could not be recovered in the Vienna energy model (e.g., pseudoknots or single base pairs bridging two individually unstable two-way junctions (Supplementary Methods).

Eterna developers created several features for the first full round of puzzles. In Eterna, RNA secondary structures are displayed either in “target mode” (in which the RNA is made to adopt its desired secondary structure and is laid out accordingly) or “natural mode” (in which the RNA adopts its MFE conformation). To ameliorate the innumerable overlaps resulting from the naïve layout algorithm available in Eterna, the puzzles were modified to accept a “custom” layout for the target structure, whereby the correct RNA secondary structure had fixed Cartesian coordinates for each nucleotide, and every time a junction’s orientation was solved in “natural mode”, it would “snap” to the target mode coordinates, eliminating all target mode overlaps and improving the situation in natural mode. Furthermore, to provide more detailed feedback to players, a constraint was implemented using sequence conservation of each nucleotide in the rRNA sequences across gammaproteobacteria. We encoded the possible mutations at each position in terms of the IUPAC symbols for ambiguous nucleotides, e.g., Y = U or C; W = A or U. This “IUPAC constraint” ensured that players had real-time access to the mutations that are tolerated in related organisms, thus allowing players to vote for solutions that might have many mutations, but relatively few “IUPAC violations.” The first round also included several “subpuzzles” permitting players to explore the individual domains from these large molecules in more detail.

For the second round, two significant changes were made to improve the 16S rRNA puzzle specifically. First, because the energy models used in Eterna—especially for ribosome puzzles—omit pseudoknots, the 16S helix ‘h2’, which is properly a pseudoknot, had only been modeled implicitly via IUPAC constraints or locks. In the second round, a second version of the 16S rRNA puzzle was provided with h2 defined instead of h1, permitting players to test their designs in each structural context. Second, the sequence used for the “pilot” (R0) and R1 16S rRNA was only 1534 nucleotides, omitting the anti-Shine Dalgarno (aSD) sequence. In the R2 16S rRNA puzzles, the aSD sequence appears and is locked to its WT sequence identity. The science team also created resources that the players could use as a supplement to their design process. Several players concerned with the prospect of disrupting contacts with rProteins asked the science team for annotations of what nucleotides contacted proteins in each rRNA. The science team used a 3D structure of the *E. coli* ribosome (PDB code: 4YBB^37^) to annotate each protein-contacting residue. After designs were collected, to enable players to analyze these contacts in detail, the science team additionally annotated which contacts directly influenced the allowed nucleotides at that position (i.e., a contact through the phosphate backbone does not constrain the nucleotide sequence, a contact with adenosine N7 could be satisfied by guanosine, but a contact with guanosine’s keto group could not be satisfied by another nucleotide).

### Statistics and reproducibility

All data were collected in replicates. Data analysis was conducted using R versions 4.0.2 or higher or Excel Version 2022. For each iSAT or CFPS experiment, at least three wild type replicates were included, sfGFP expression time course data (RFUs) of all wild type replicates in the data set were collected, their average RFU calculated for each timepoint, and the maximal average RFU used to normalize all individual RFUs to relative RFU rates so that the highest relative rate for wild type for the collected data set was 1. For the iSAT or CFPS time courses, the relative average RFU of each ribosome design and its standard deviation at each time point were calculated and plotted versus iSAT or CFPS time to assess sfGFP during iSAT or CFPS. Maximal individual RFU rates of each ribosome design were determined from each data set and plotted as boxplots with error bars representing standard deviation to assess the functionality of a ribosome design in iSAT or CFPS. Provided means and standard deviations for maximal sfGFP expression in iSAT or CFPS were calculated using the individual maximal RFU rates. Statistical analysis of the iSAT and CFPS data for the combinatorial designs was conducted using a two-sided, unpaired *t*-test in R. Prior to the *t*-test, the equality of the variance of the compared designs was determined using a two-sided *F*-test in R. Dependent on the *F*-test result, Two Sample *t*-test (equal variance in both data sets) or Welch Two Sample *t*-test (different variance in both data sets) was conducted.

Ribosome sedimentation experiments were performed twice with similar results. To depict the ribosome sedimentation profiles of wild type and R1–09/R1–33 in the same plot, A260 absorbance data of each sample were collected, the minimum determined, subtracted from all A260 values of the corresponding data set, and plotted versus the direction of sedimentation. To plot the sedimentation profile of wild type above the one of R1–09/R1–33, all wild type A260 values were increased by 0.1 (arbitrary number).

### Reporting summary

Further information on research design is available in the Nature Portfolio Reporting Summary linked to this article.

## Supplementary information

Supplementary Information

Reporting Summary

## Data Availability

The authors declare that the data supporting the findings of this study are available within the paper and its Supplementary Information files. Supplementary Information contains Supplementary Figures, a Source Data file describing all data presented and computational parameters derived for this work, describing each computationally predicted or community science-designed rRNAs, Supplementary Table 1 contains data for the performance of the wild type *E. coli* ribosome in iSAT subjected to increasing concentrations of diverse solvents, and Supplemental Methods providing details on ribosome puzzle construction and plasmid sequences. The data used in this study and designs’ RNA sequences have been deposited as a Source Data file available at https://purl.stanford.edu/hp267yt1382. Source data are provided in this paper.

## Source data

Source Data
