# Supplementary material for: Community science designed ribosomes with beneficial phenotypes
Source: Nat Commun. 2023 Feb 21;14:961. doi: 10.1038/s41467-023-35827-3 (PMC9944925; doi:10.1038/s41467-023-35827-3)
Supplement: Supplementary file 1 — Supplementary Information [file 41467_2023_35827_MOESM1_ESM.pdf]

SUPPLEMENTARY INFORMATION

Community science designed ribosomes with beneficial phenotypes

Antje Krüger[1,2], Andrew M. Watkins[3,4], Roger Wellington-Oguri[5], Jonathan Romano[3,5,6], Camila Kofman[1], Alysse DeFoe[1], Yejun Kim[1], Jeff Anderson-Lee[5], Eli Fisker[5], Jill Townley[5], Eterna Participants[5,*], Anne E. d'Aquino[1], Rhiju Das[3,7], Michael C. Jewett[1,8]

Affiliations:

[1]Department of Chemical and Biological Engineering, Chemistry of Life Processes Institute, and Center for Synthetic Biology, Northwestern University, Evanston, IL 60208, USA. [2]Present address: Resilience US Inc, 9310 Athena Circle, La Jolla, CA 92037, USA. [3]Department of Biochemistry, Stanford University, Stanford, CA 94305, USA. [4]Present address: Prescient Design, Genentech, 1 DNA Way, South San Francisco, CA 94080, USA. [5]Eterna Massive Open Laboratory, Stanford, CA 94305, USA. [6]Department of Computer Science and Engineering, State University of New York at Buffalo, Buffalo, NY 14260, USA. [7]Department of Physics, Stanford University, Stanford, CA 94305, USA. [8]Robert H. Lurie Comprehensive Cancer Center and Simpson Querrey Institute, Northwestern University, Chicago, IL 60611, USA. *A list of authors and their affiliations appears at the end of the Supplementary Information.

These authors contributed equally: Antje Krüger, Andrew M. Watkins.

Correspondence should be addressed to R.D. (rhiju@stanford.edu) or to M.C.J. (email: m-jewett@northwestern.edu).

TABLE OF CONTENTS

SUPPLEMENTARY FIGURES **3**

SUPPLEMENTARY TABLES **130**

SUPPLEMENTARY NOTES **131**

SUPPLEMENTARY METHODS **132**

Energetic rationale for base locks in ribosome puzzle definitions 132

Plasmid sequences 133

SUPPLEMENTARY REFERENCES **143**

ETERNA PARTICIPANTS **145**

SUPPLEMENTARY FIGURES

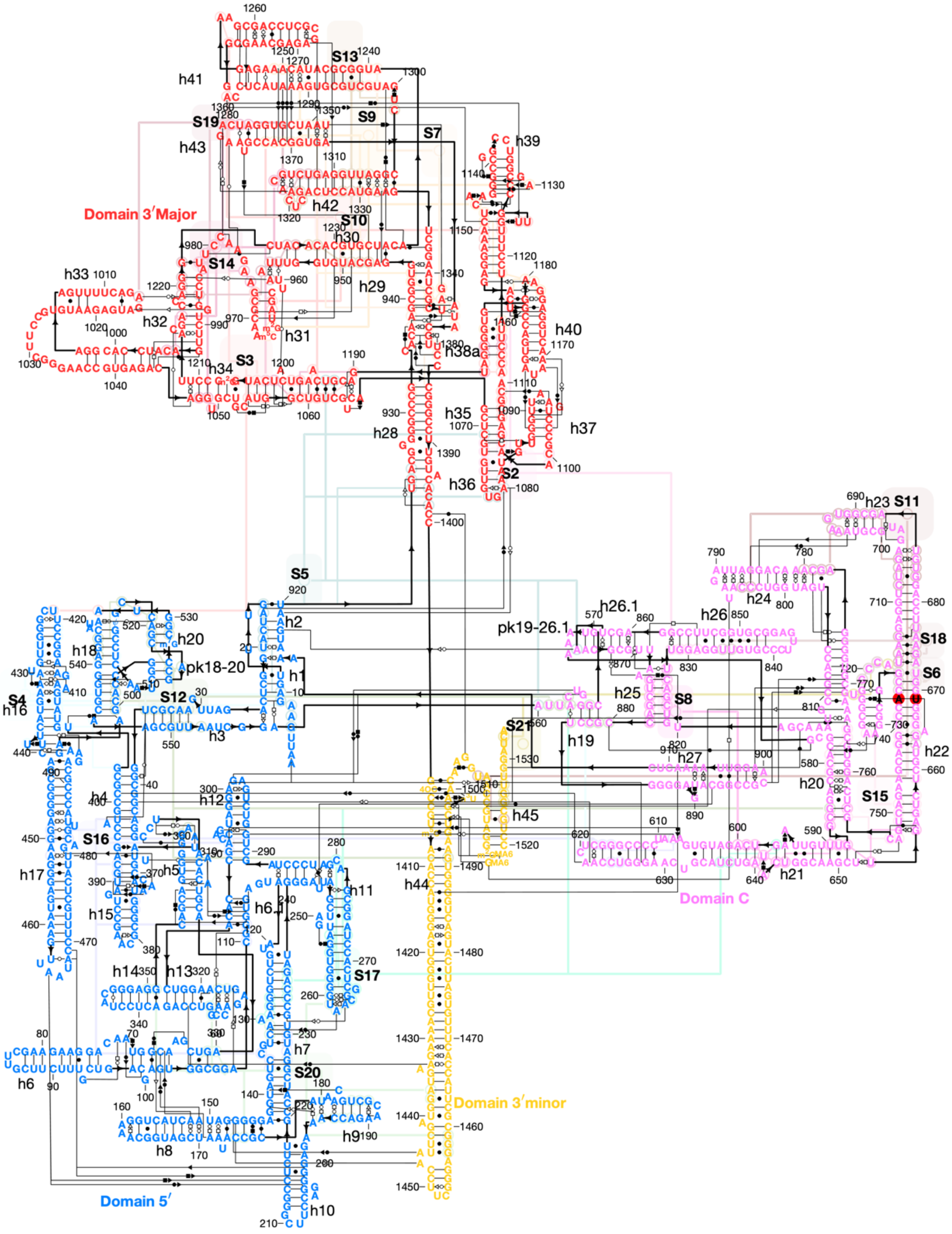

Supplementary Figure 1. Eterna participants'-designed ribosomal RNA design CS-01 prepared with RiboDraw[1].

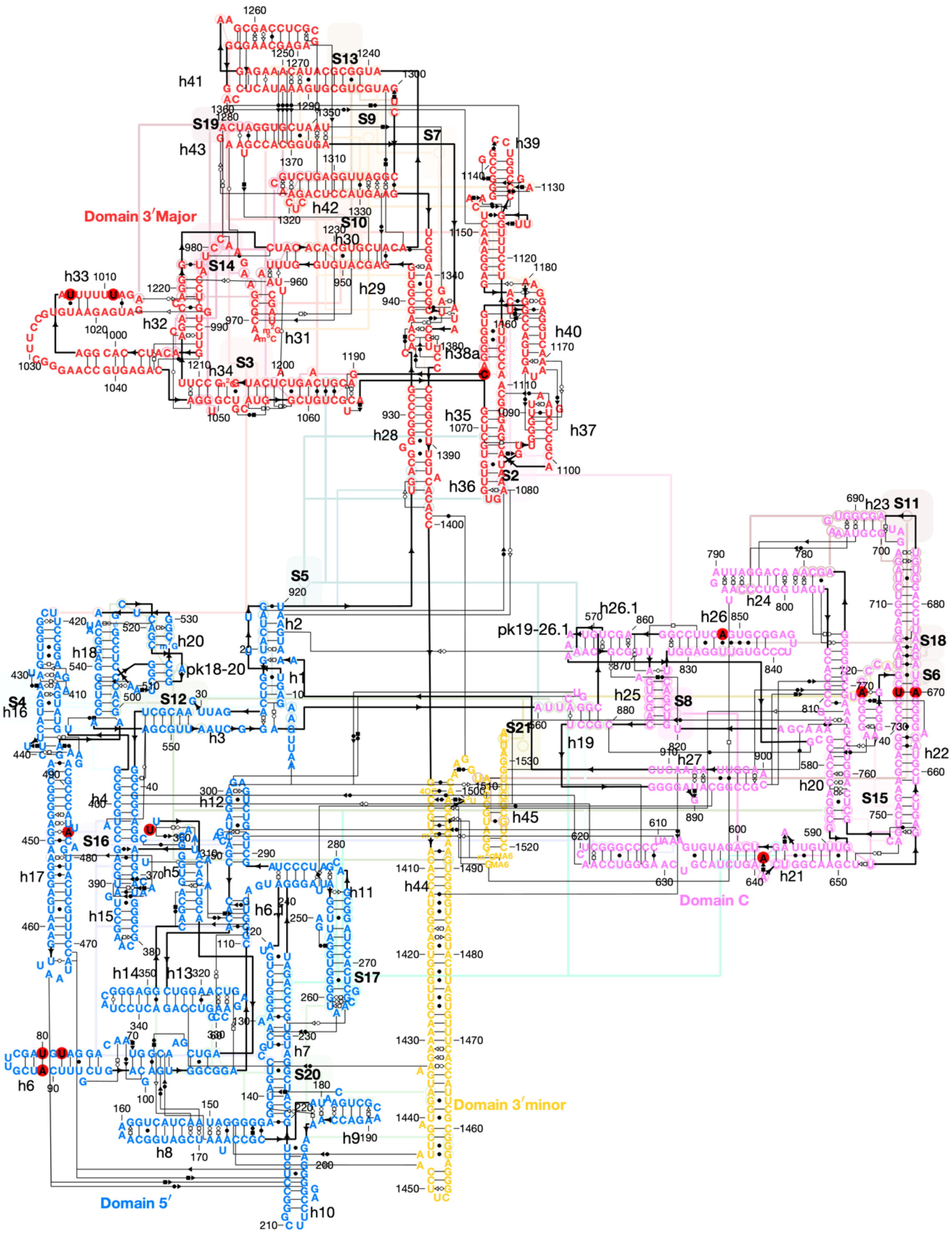

Supplementary Figure 2. Eterna participants'-designed ribosomal RNA design CS-02 prepared with RiboDraw[1].

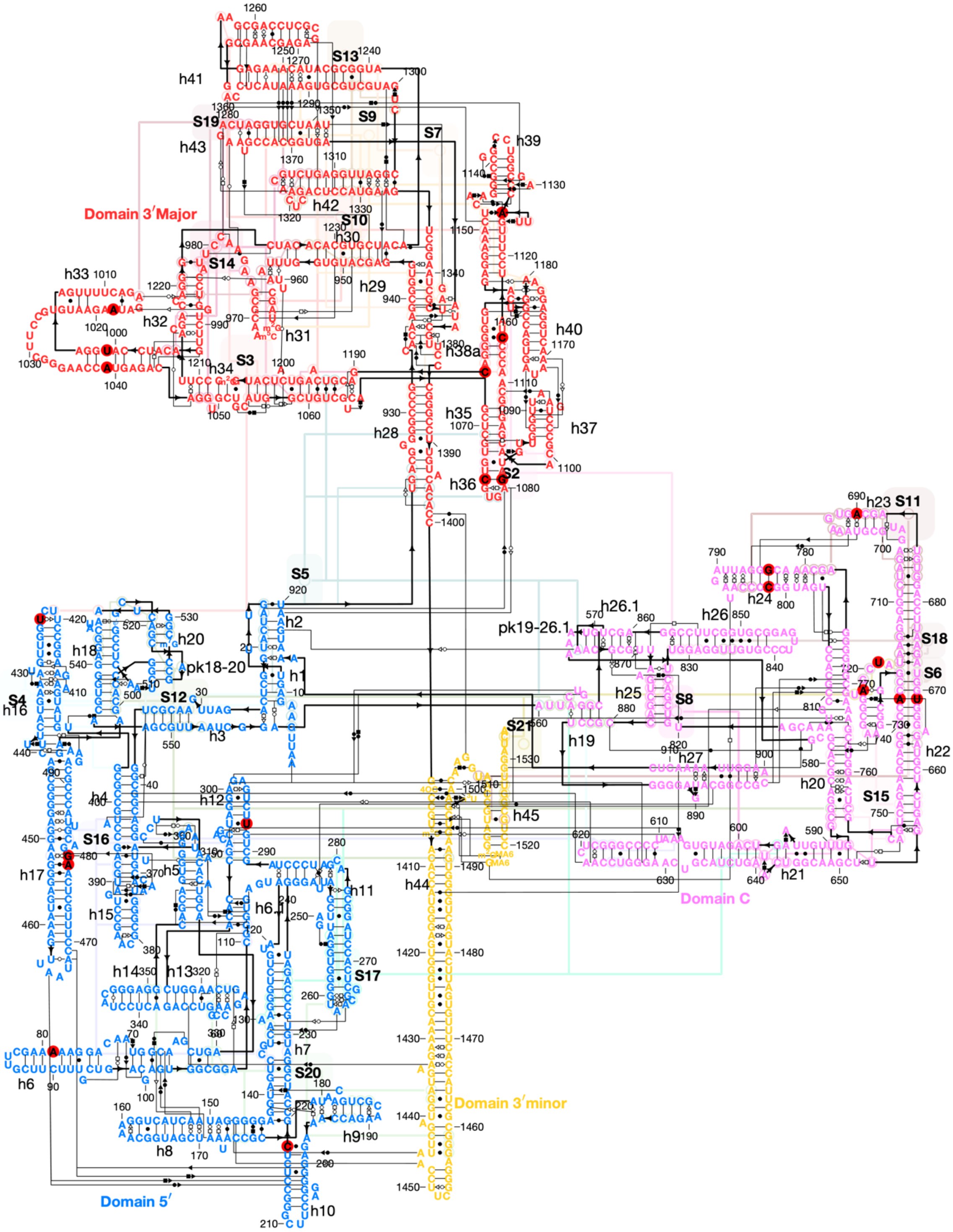

Supplementary Figure 3. Eterna participants'-designed ribosomal RNA design CS-03 prepared with RiboDraw[1].

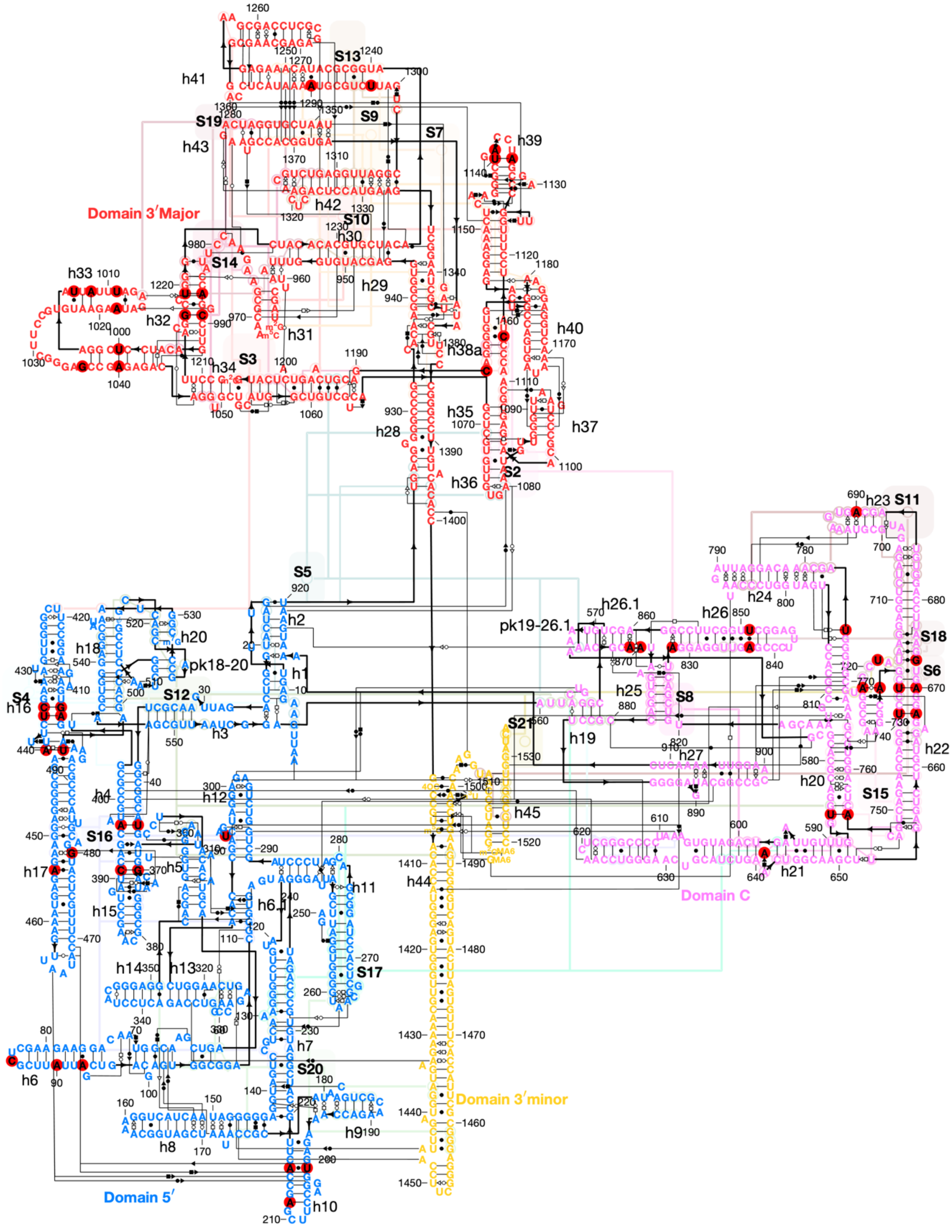

Supplementary Figure 4. Eterna participants'-designed ribosomal RNA design CS-04 prepared with RiboDraw[1].

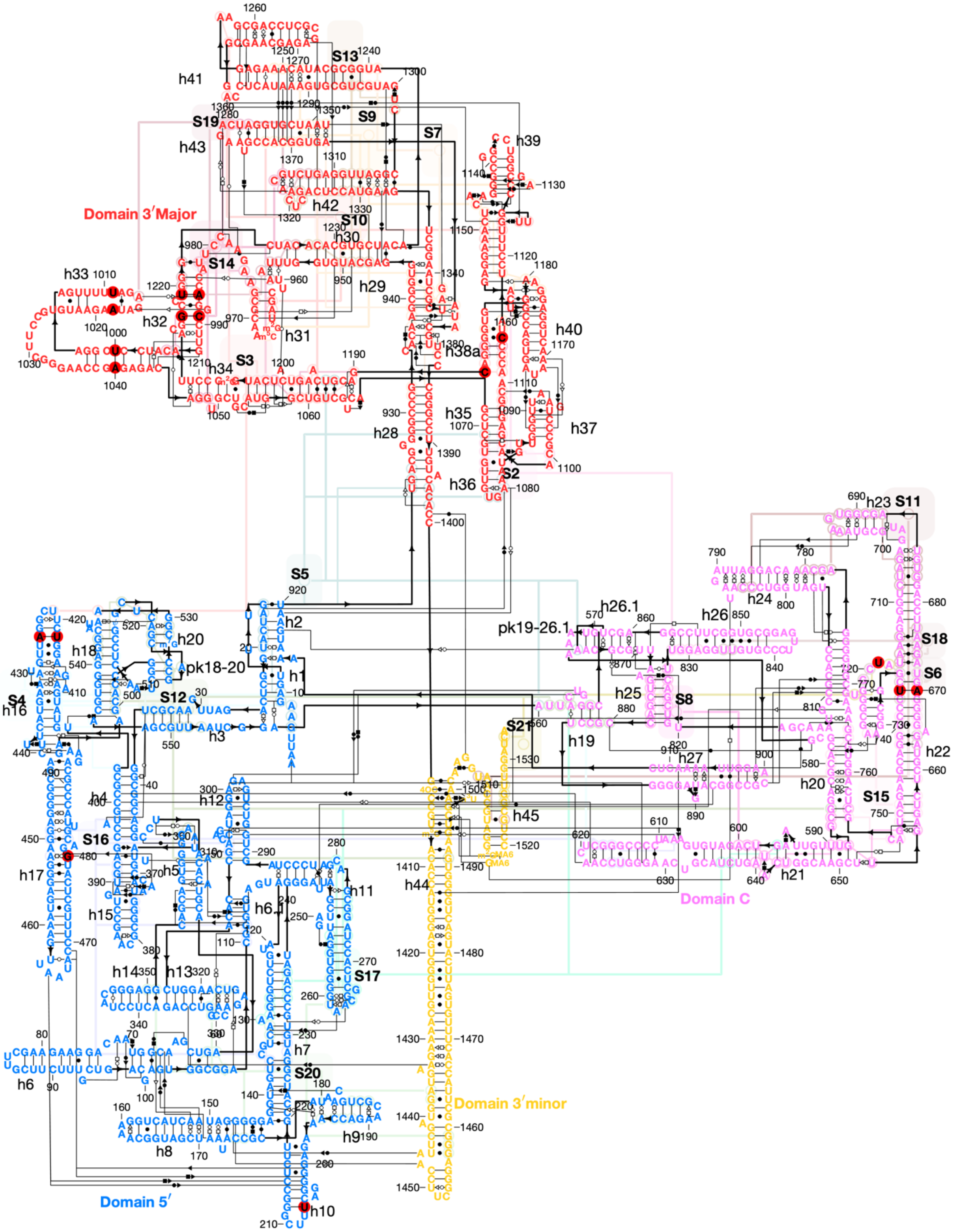

Supplementary Figure 5. Eterna participants'-designed ribosomal RNA design CS-05 prepared with RiboDraw[1].

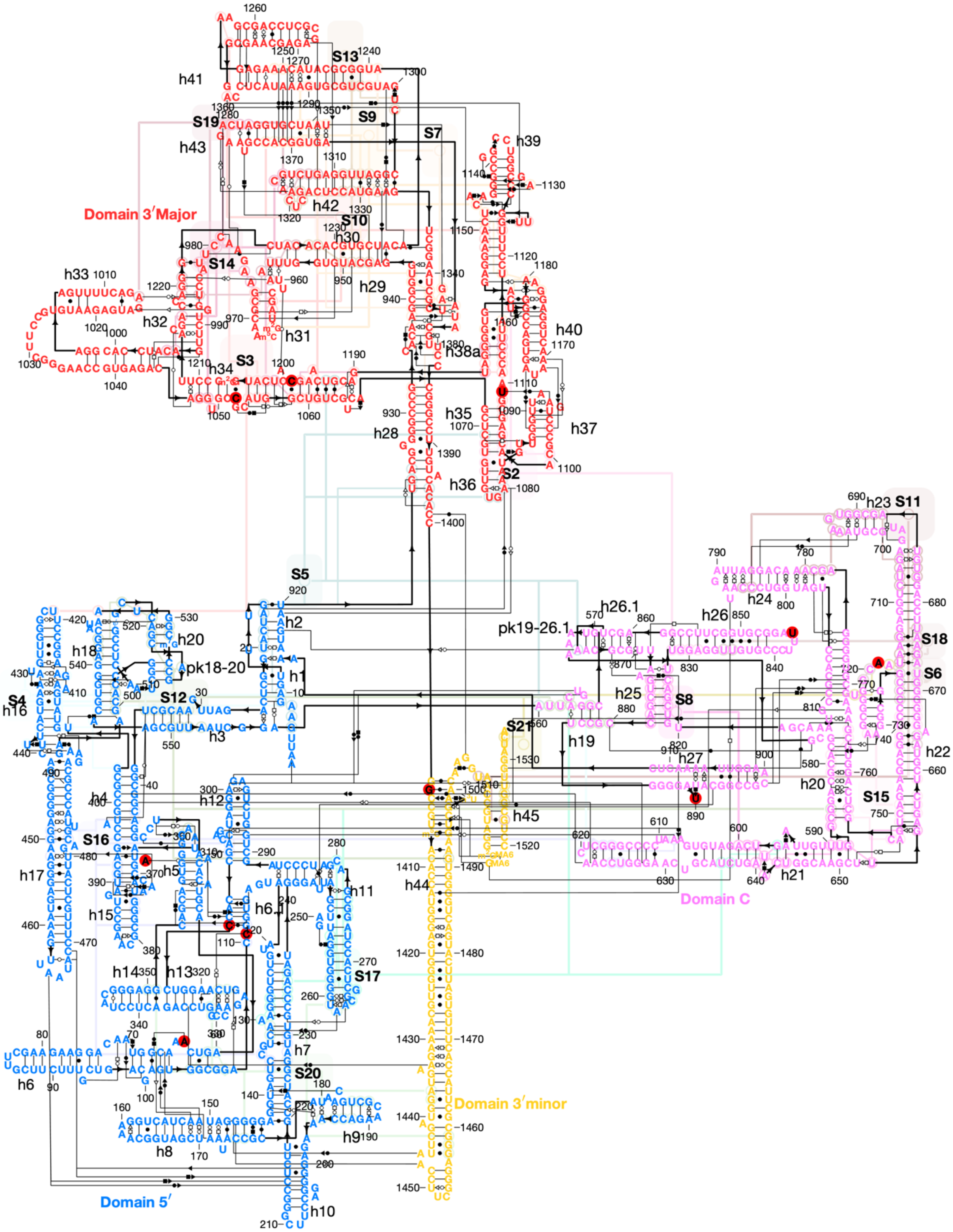

Supplementary Figure 6. Eterna participants'-designed ribosomal RNA design CS-06 prepared with RiboDraw[1].

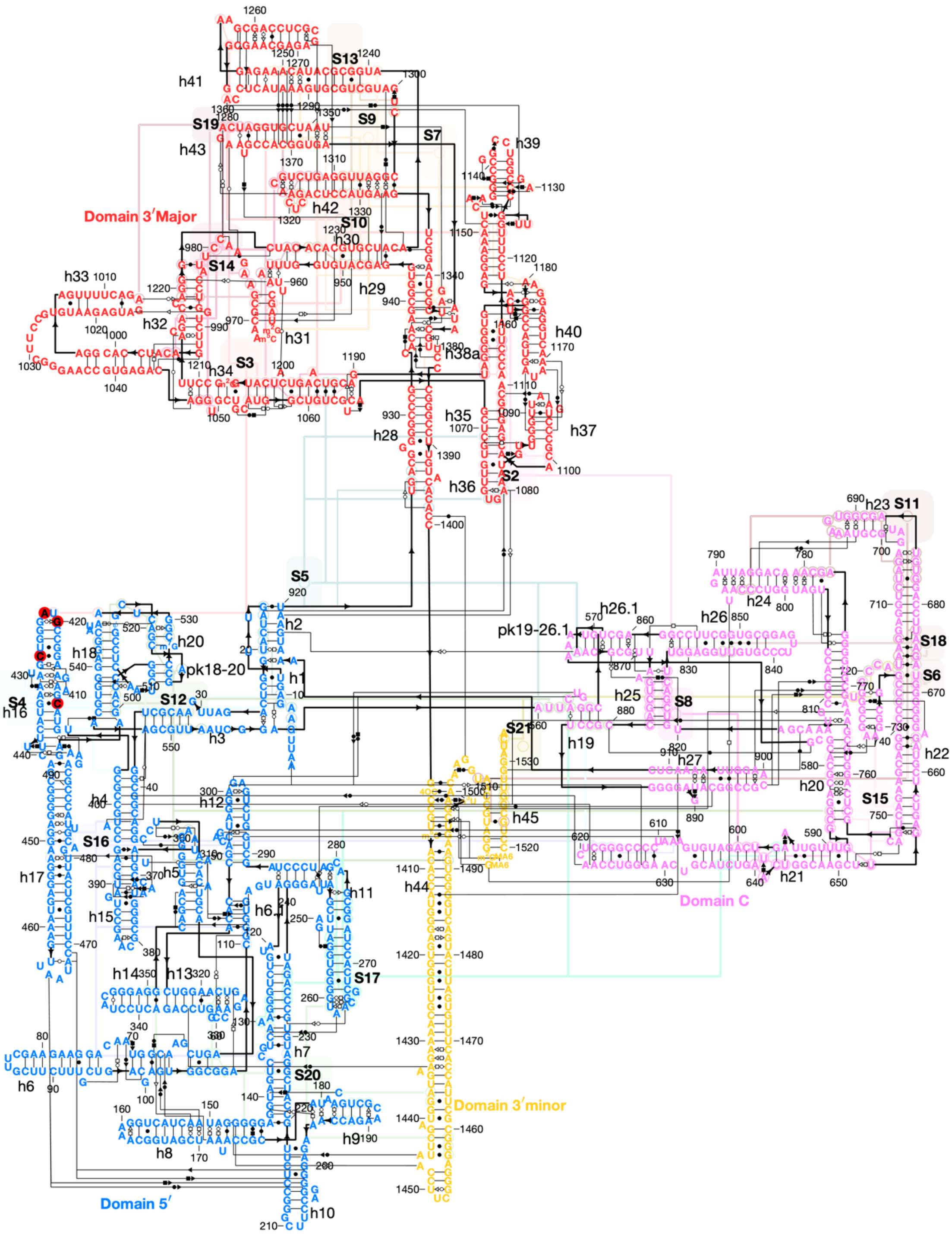

Supplementary Figure 7. Eterna participants'-designed ribosomal RNA design CS-07 prepared with RiboDraw[1].

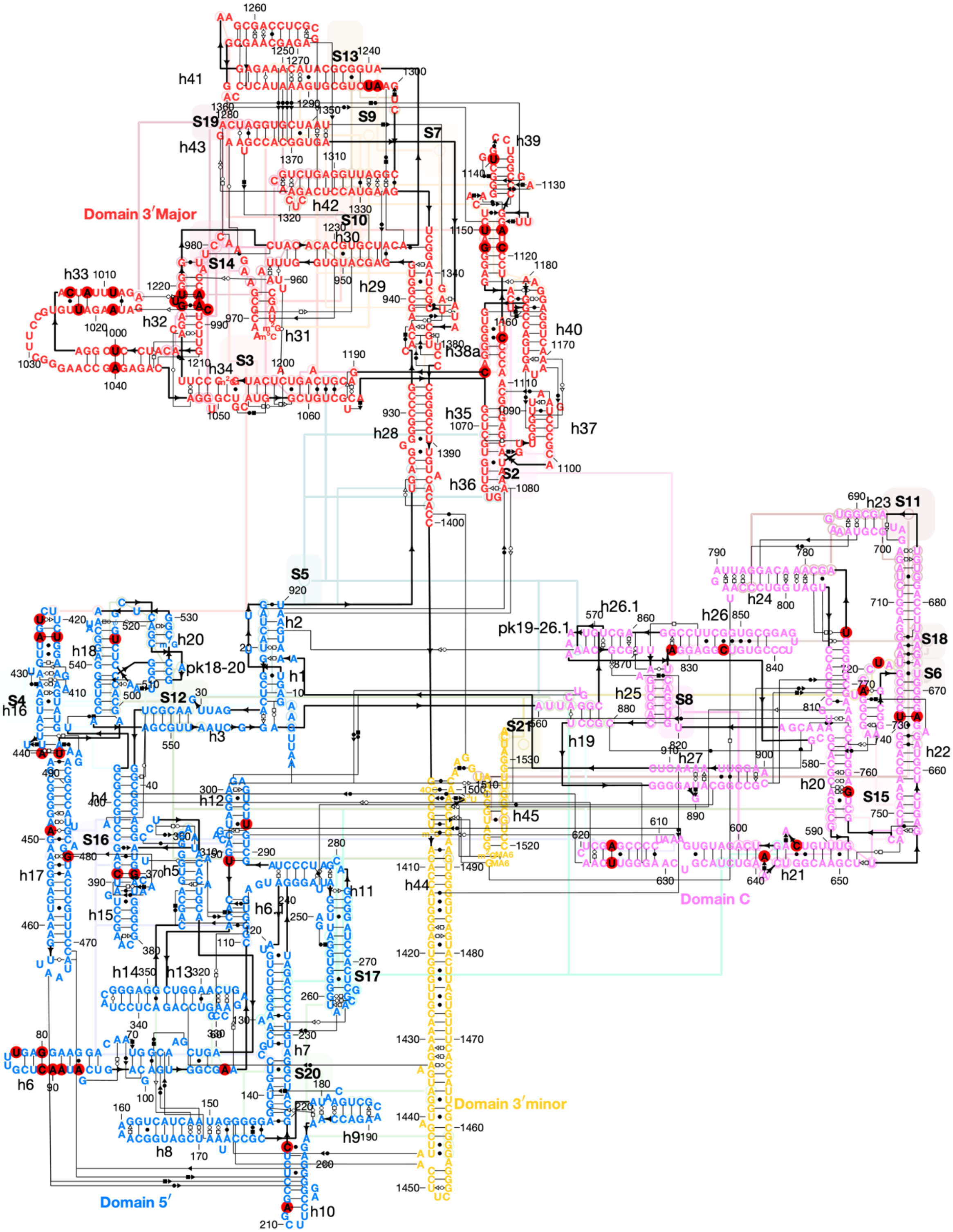

Supplementary Figure 8. Eterna participants'-designed ribosomal RNA design CS-08 prepared with RiboDraw[1].

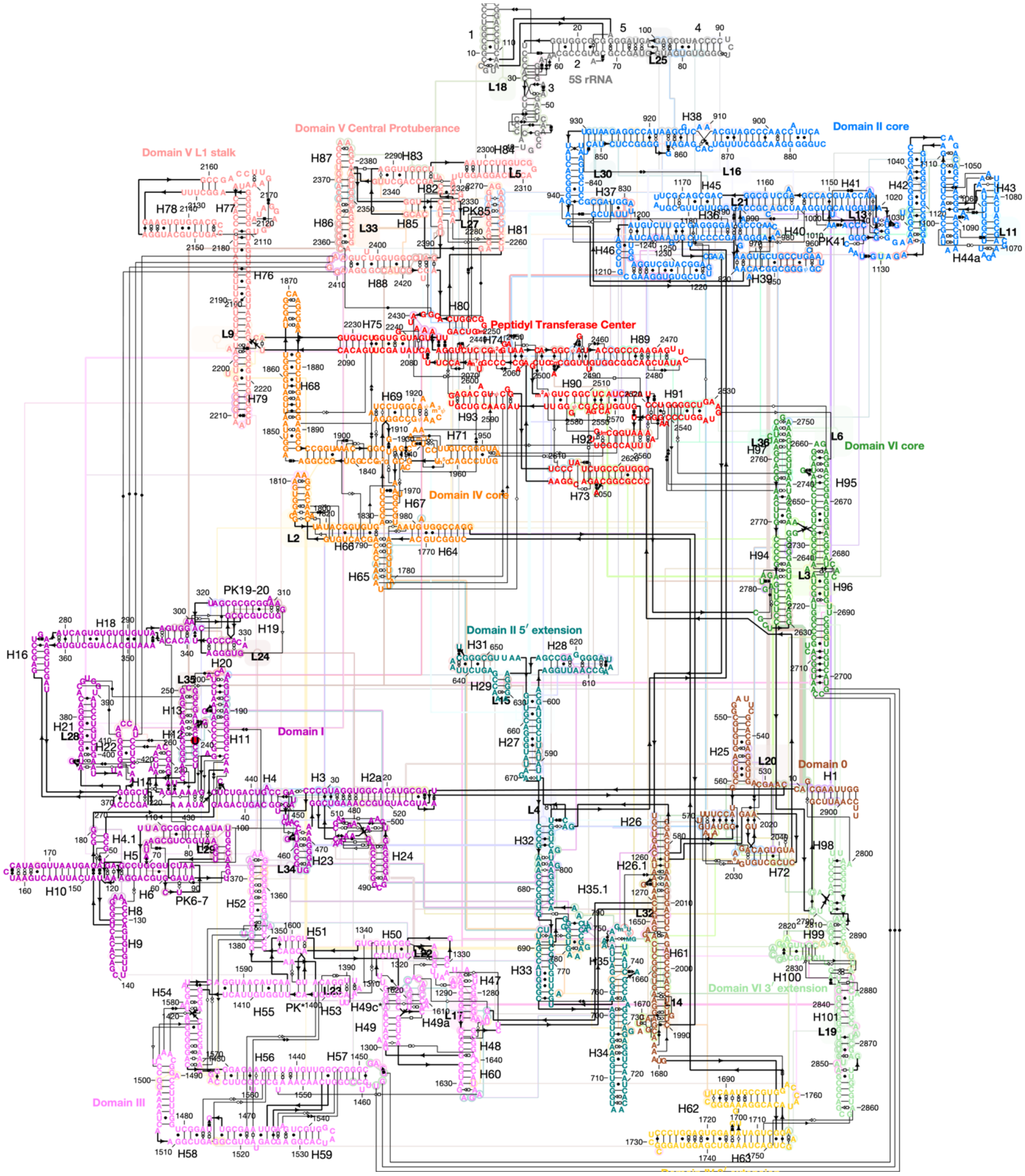

Supplementary Figure 9. Eterna participants'-designed ribosomal RNA design CS-09 prepared with RiboDraw[1].

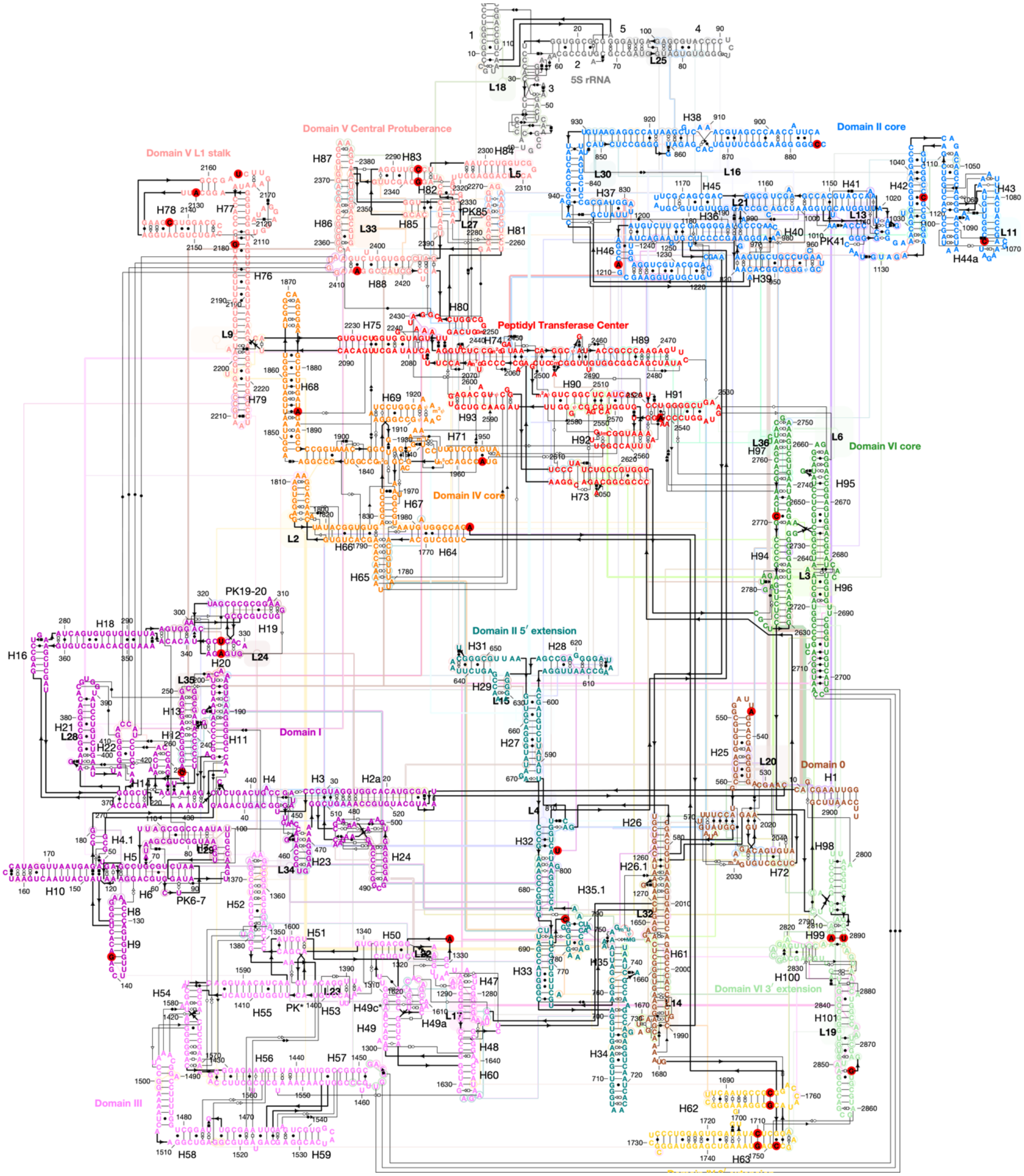

Supplementary Figure 10. Eterna participants'-designed ribosomal RNA design CS-10 prepared with RiboDraw[1].

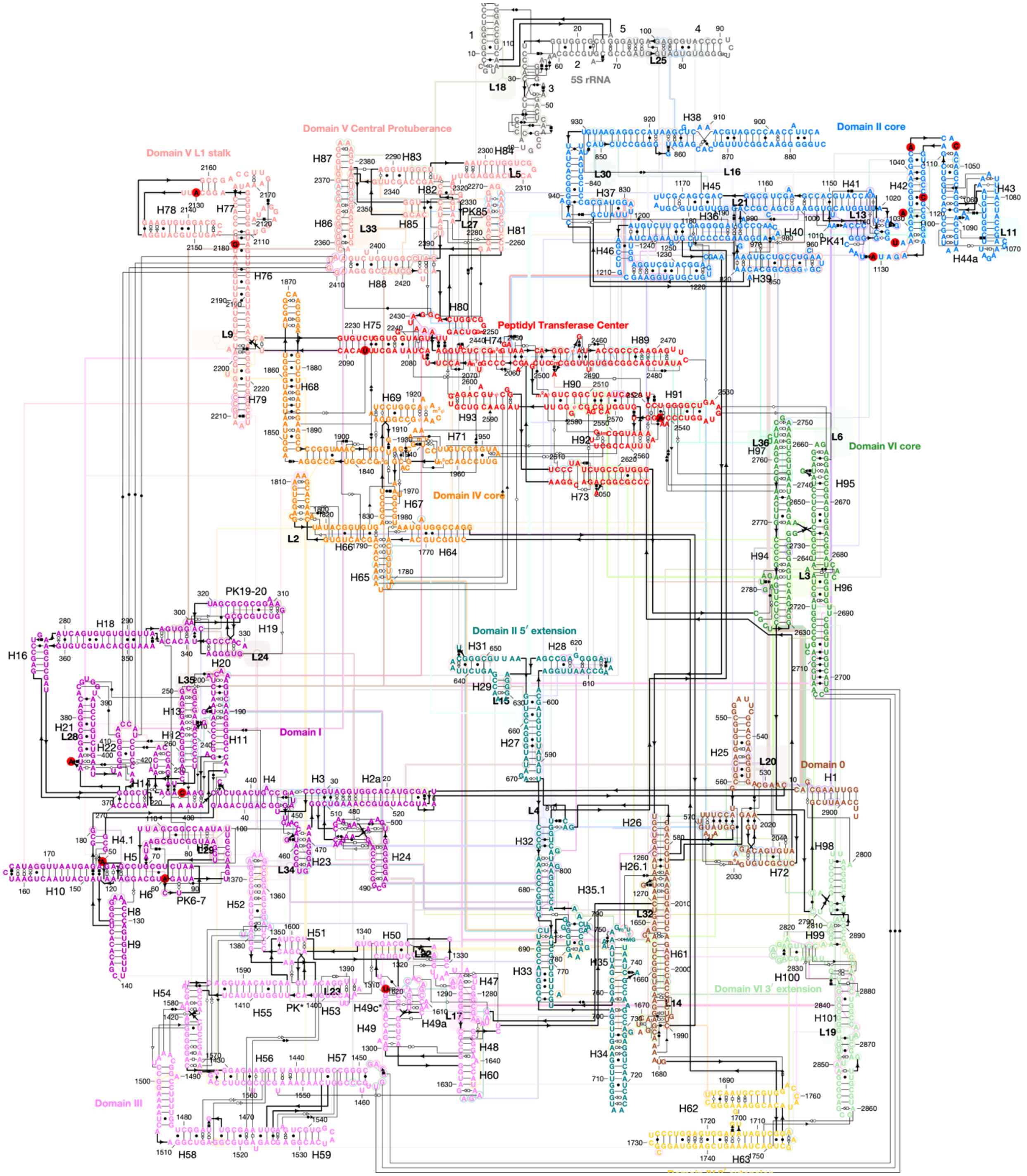

Supplementary Figure 11. Eterna participants'-designed ribosomal RNA design CS-11 prepared with RiboDraw[1].

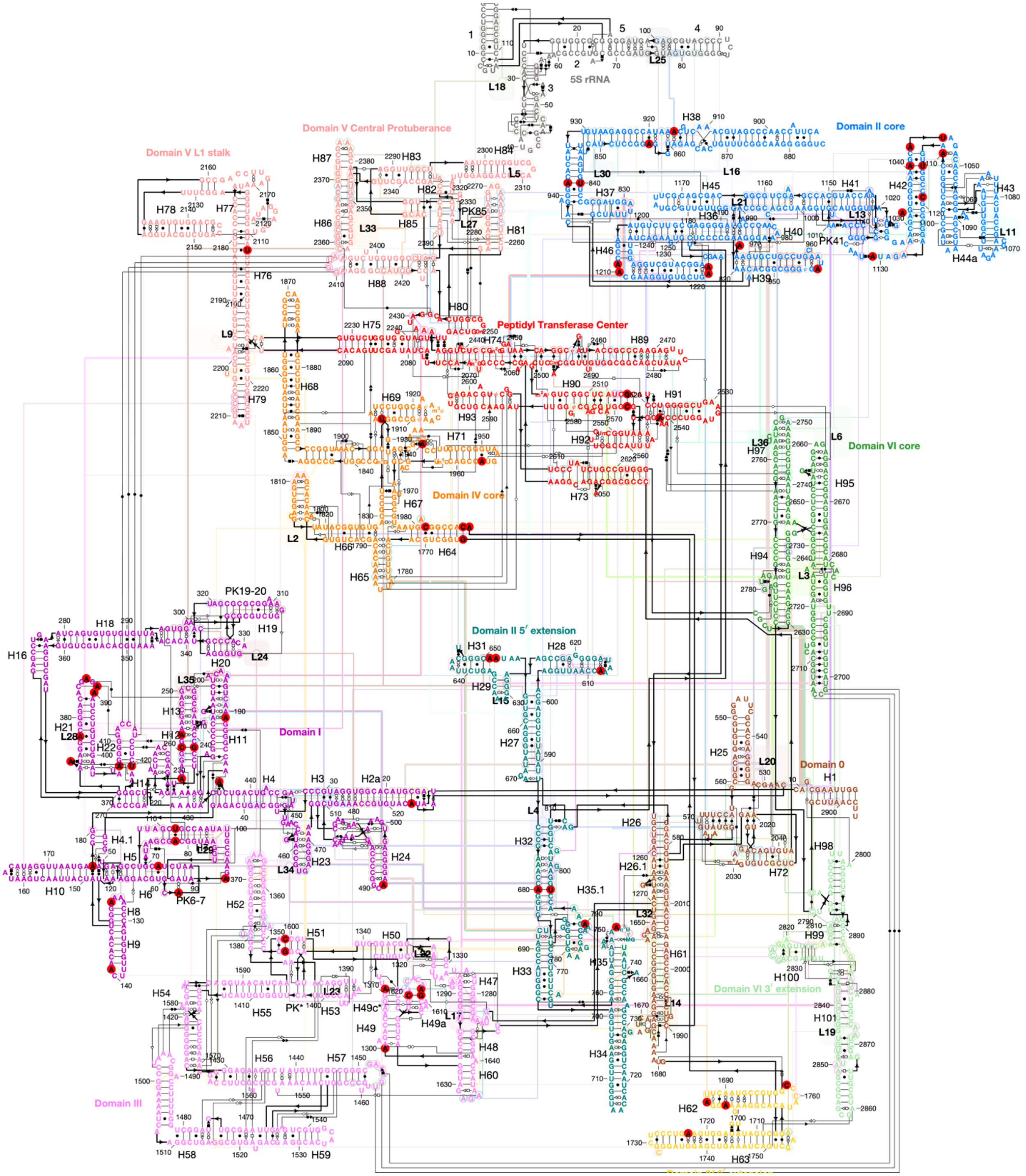

Supplementary Figure 12. Eterna participants'-designed ribosomal RNA design CS-12 prepared with RiboDraw[1].

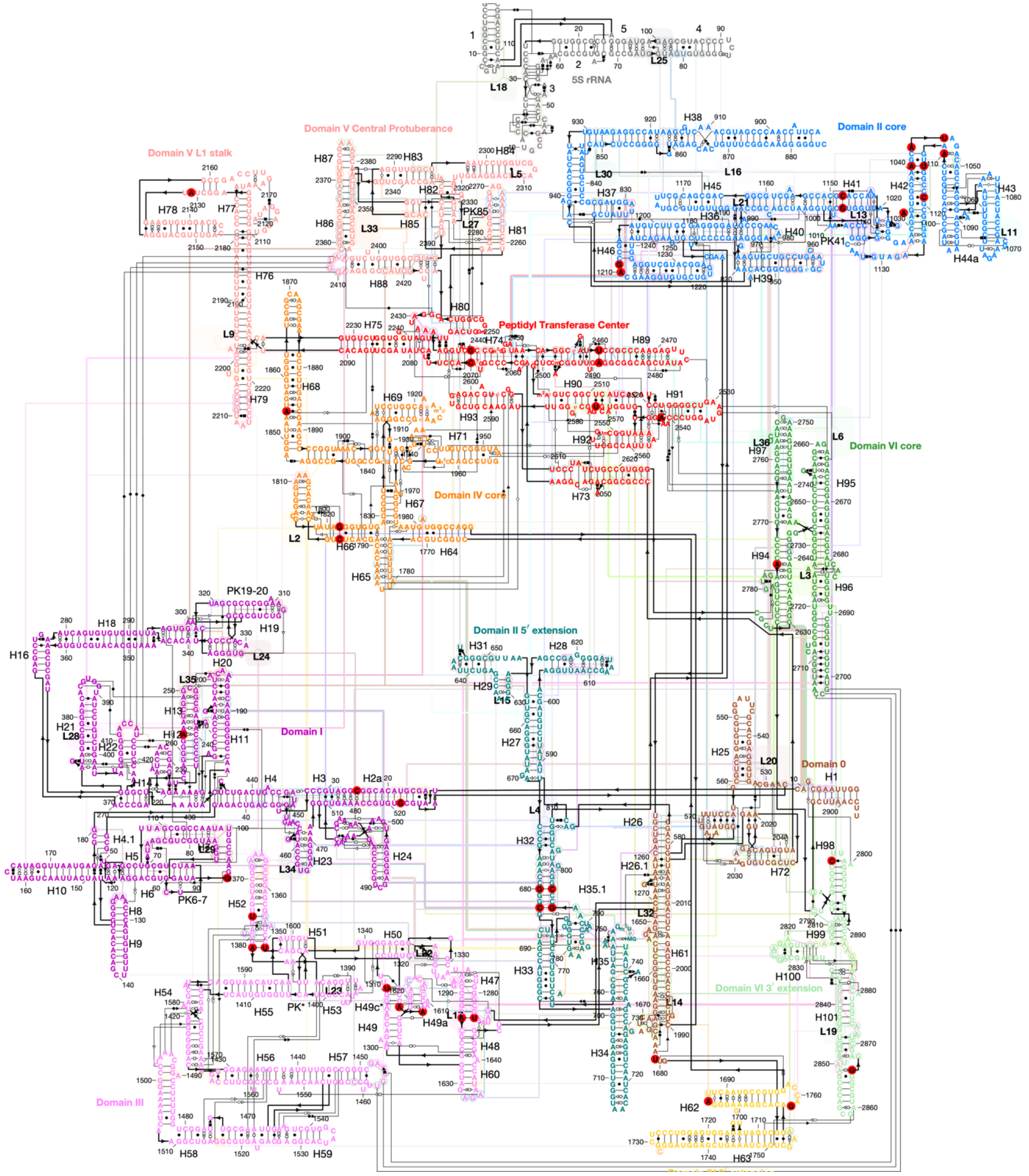

Supplementary Figure 13. Eterna participants'-designed ribosomal RNA design CS-13 prepared with RiboDraw[1].

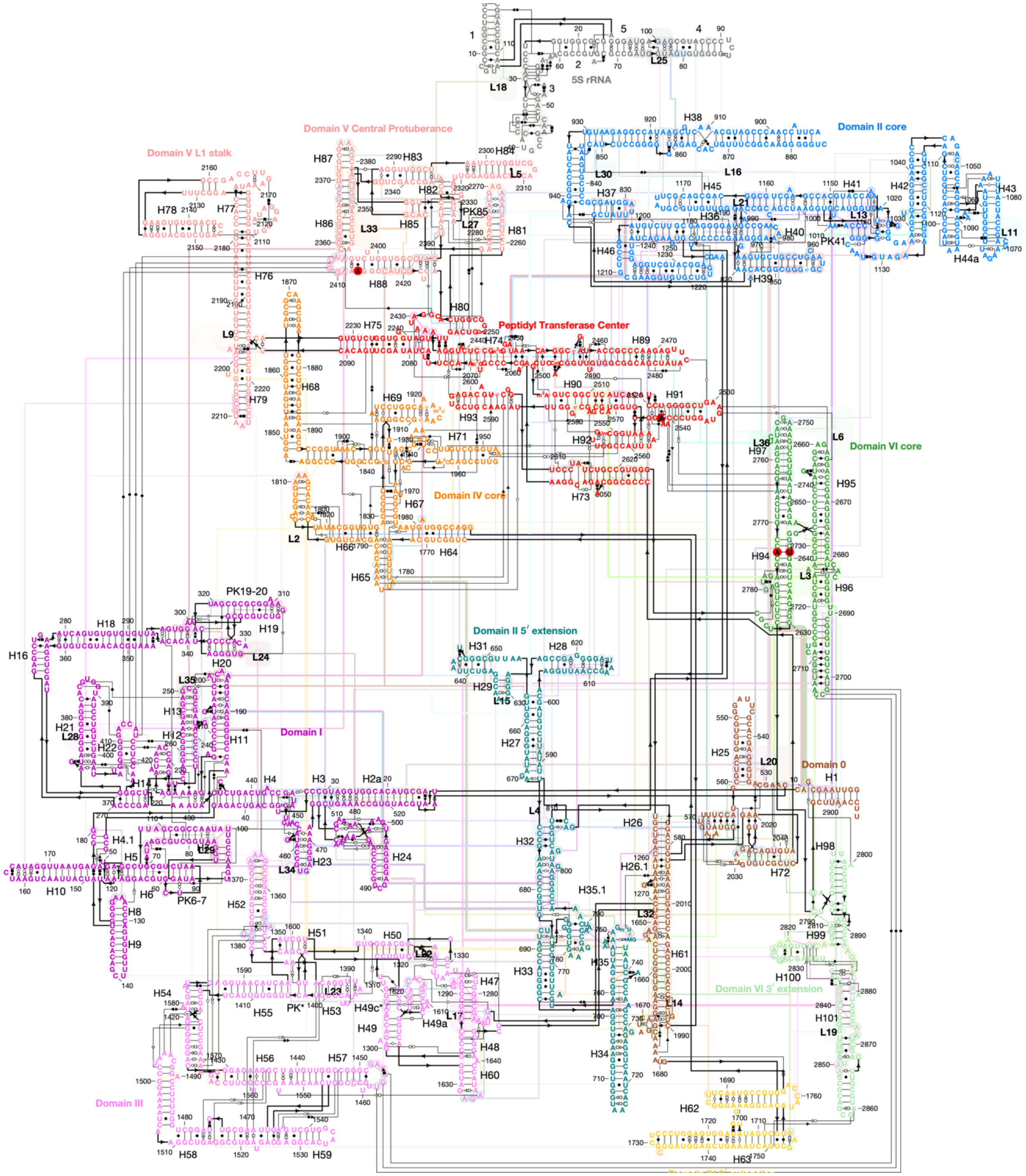

Supplementary Figure 14. Eterna participants'-designed ribosomal RNA design CS-14 prepared with RiboDraw[1].

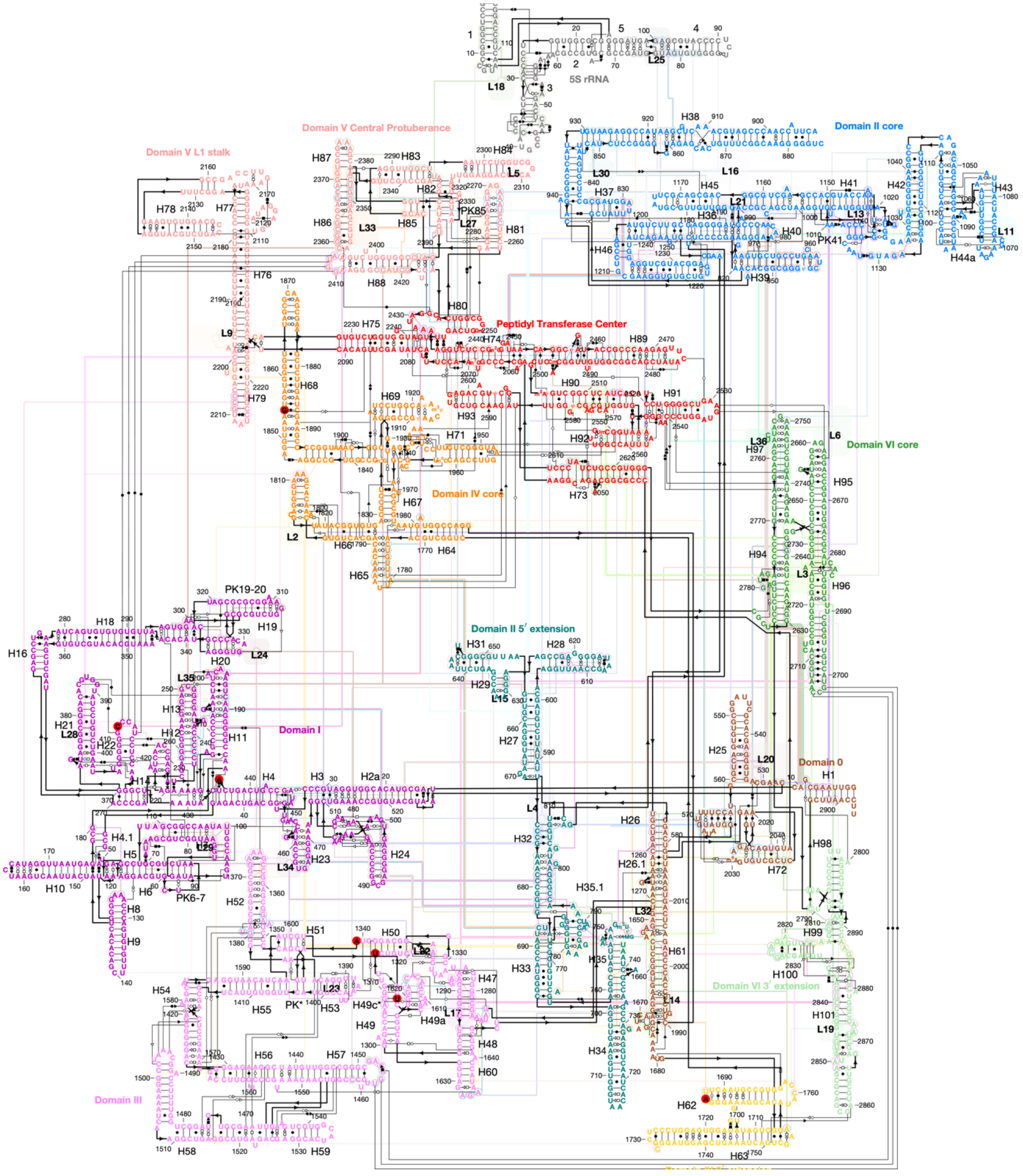

Supplementary Figure 15. Eterna participants'-designed ribosomal RNA design CS-15 prepared with RiboDraw[1].

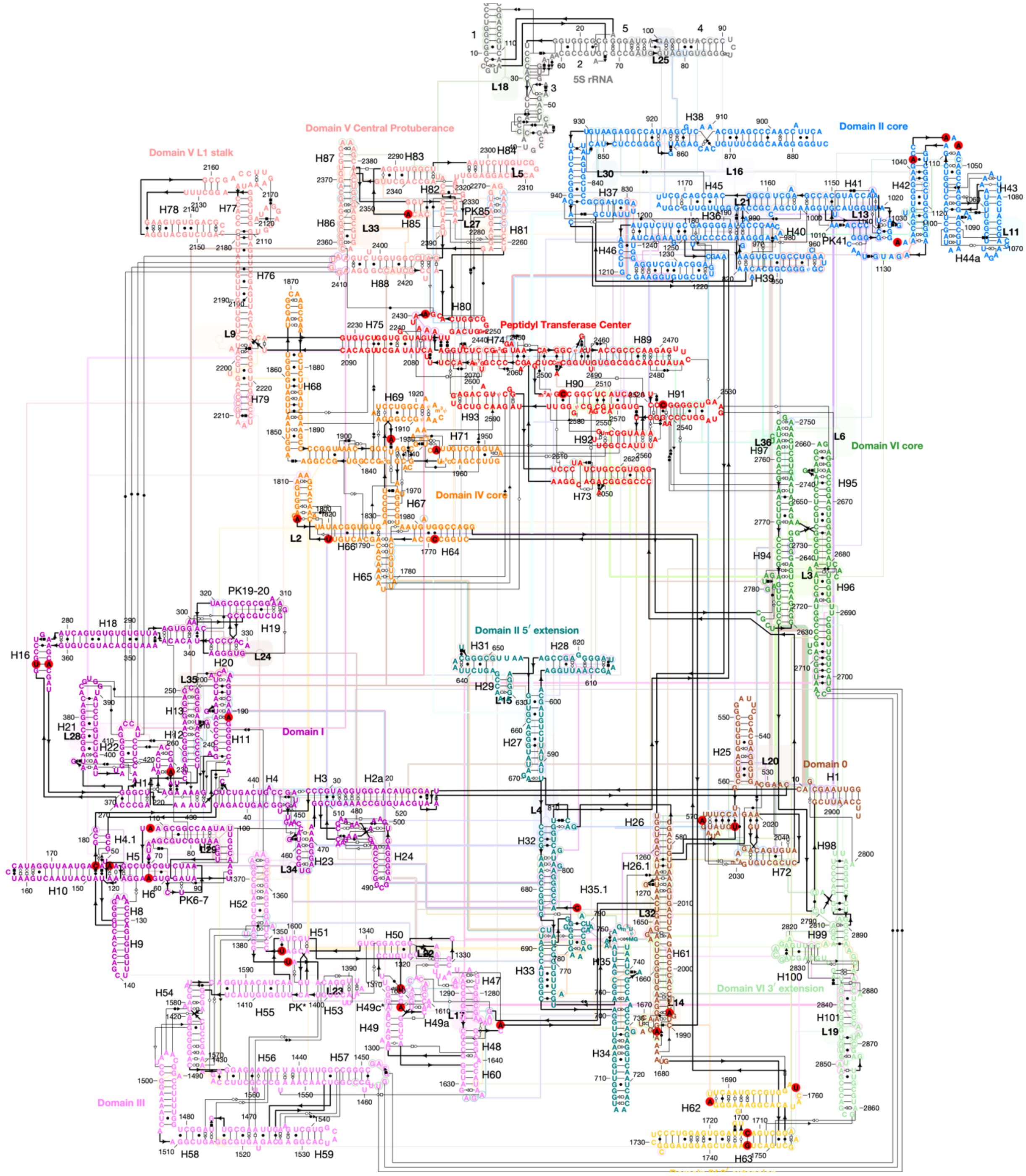

Supplementary Figure 16. Eterna participants'-designed ribosomal RNA design CS-16 prepared with RiboDraw[1].

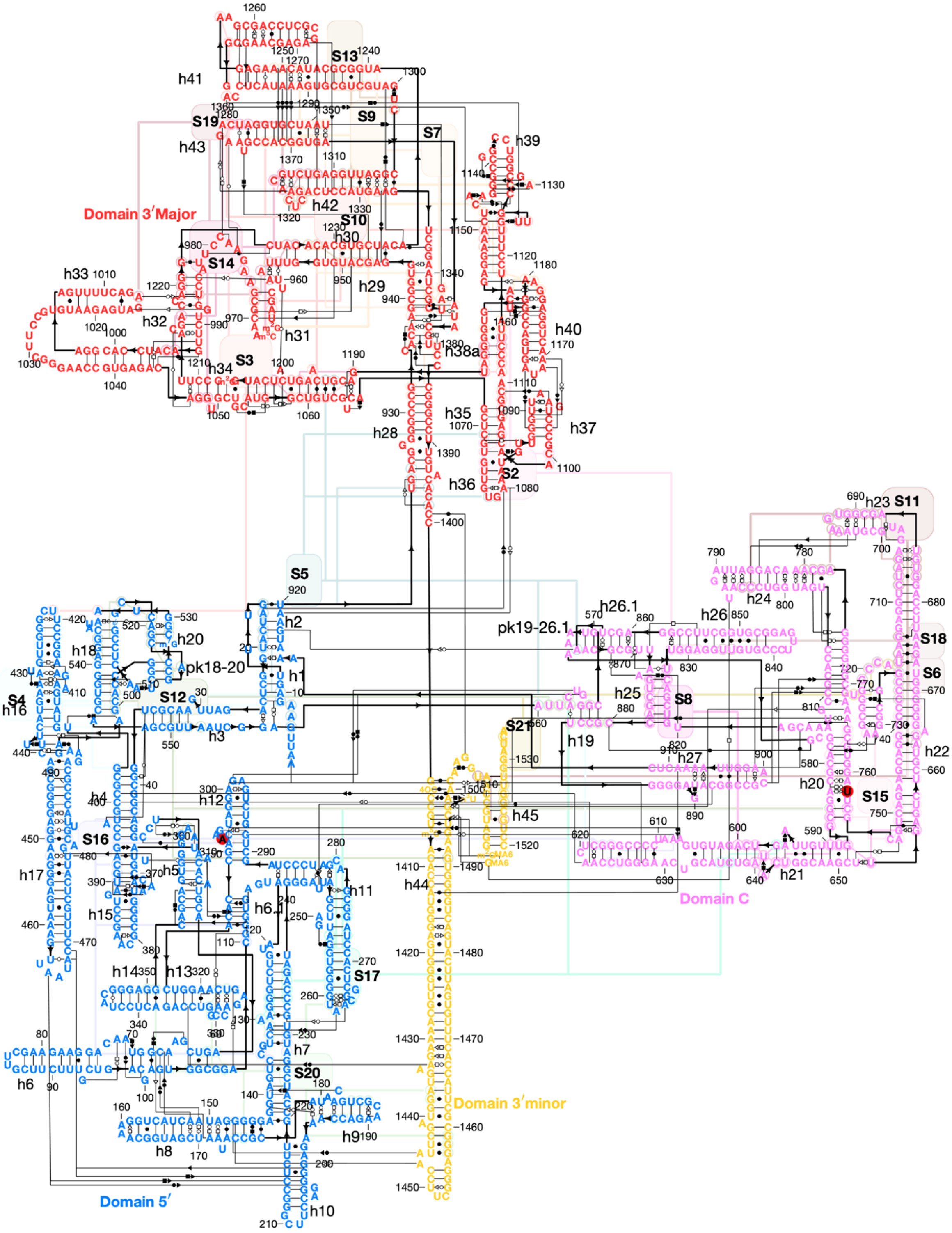

Supplementary Figure 17. Computationally predicted ribosomal RNA design CP-01 prepared with RiboDraw[1].

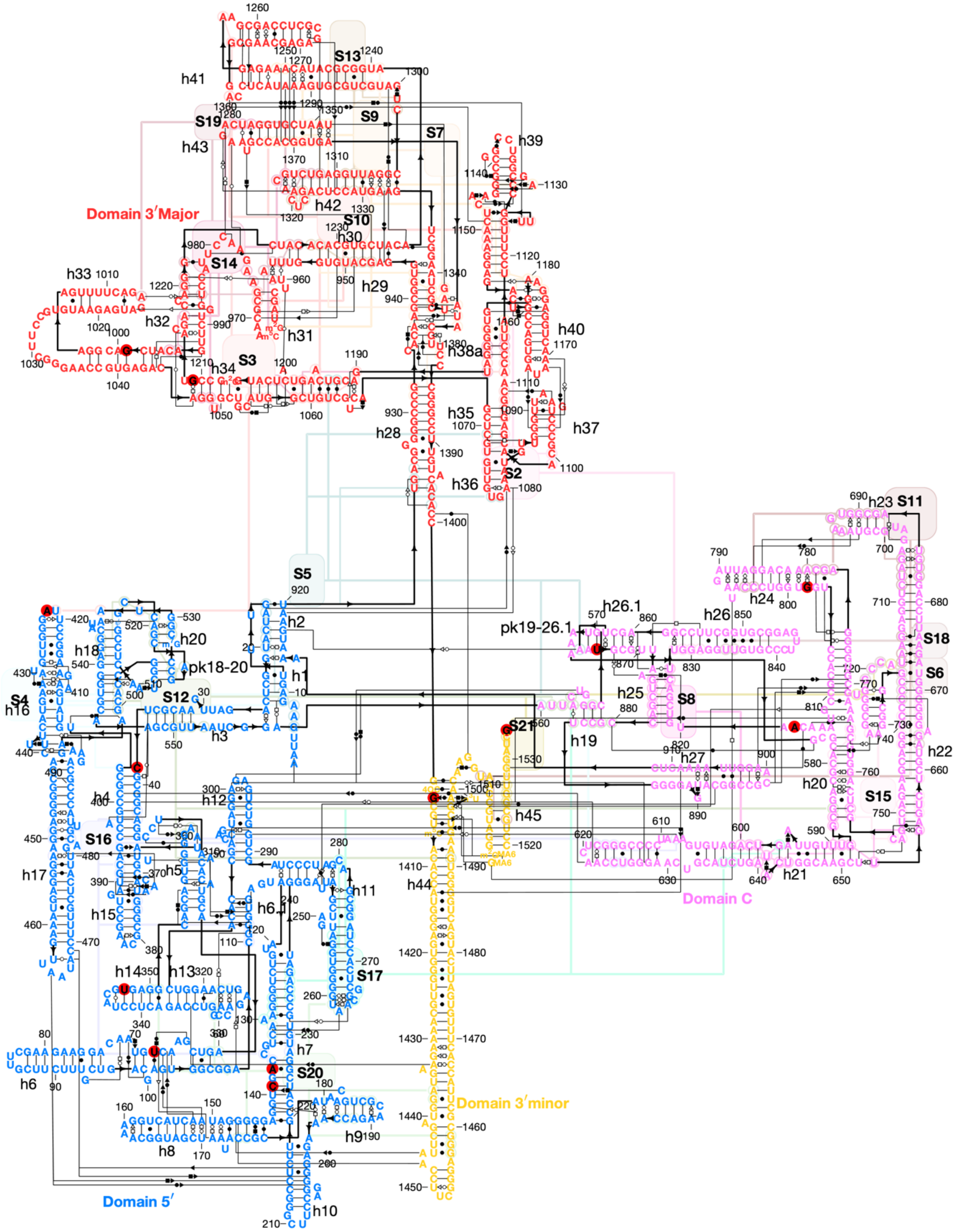

Supplementary Figure 18. Computationally predicted ribosomal RNA design CP-02 prepared with RiboDraw[1].

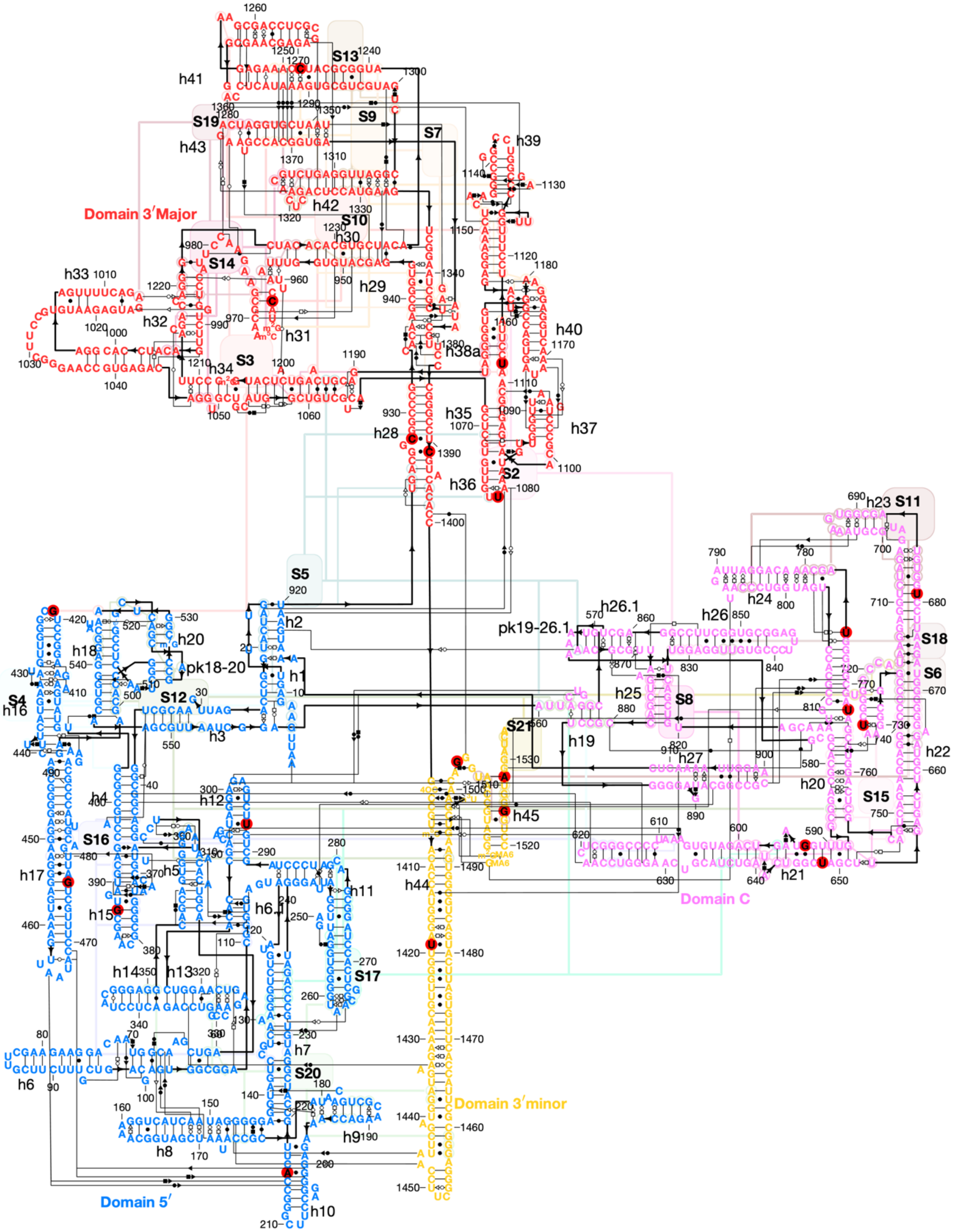

Supplementary Figure 19. Computationally predicted ribosomal RNA design CP-03 prepared with RiboDraw[1].

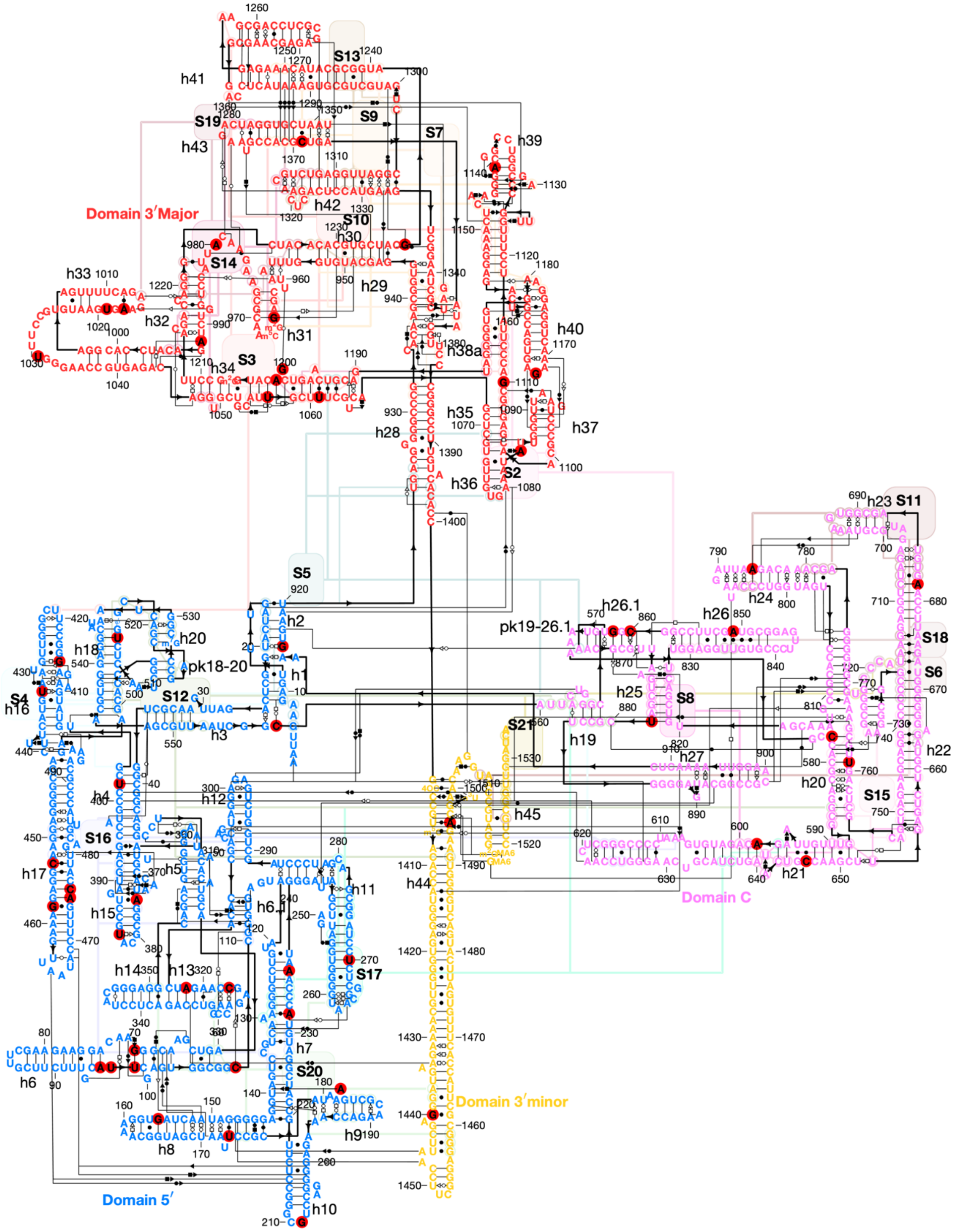

Supplementary Figure 20. Computationally predicted ribosomal RNA design CP-04 prepared with RiboDraw[1].

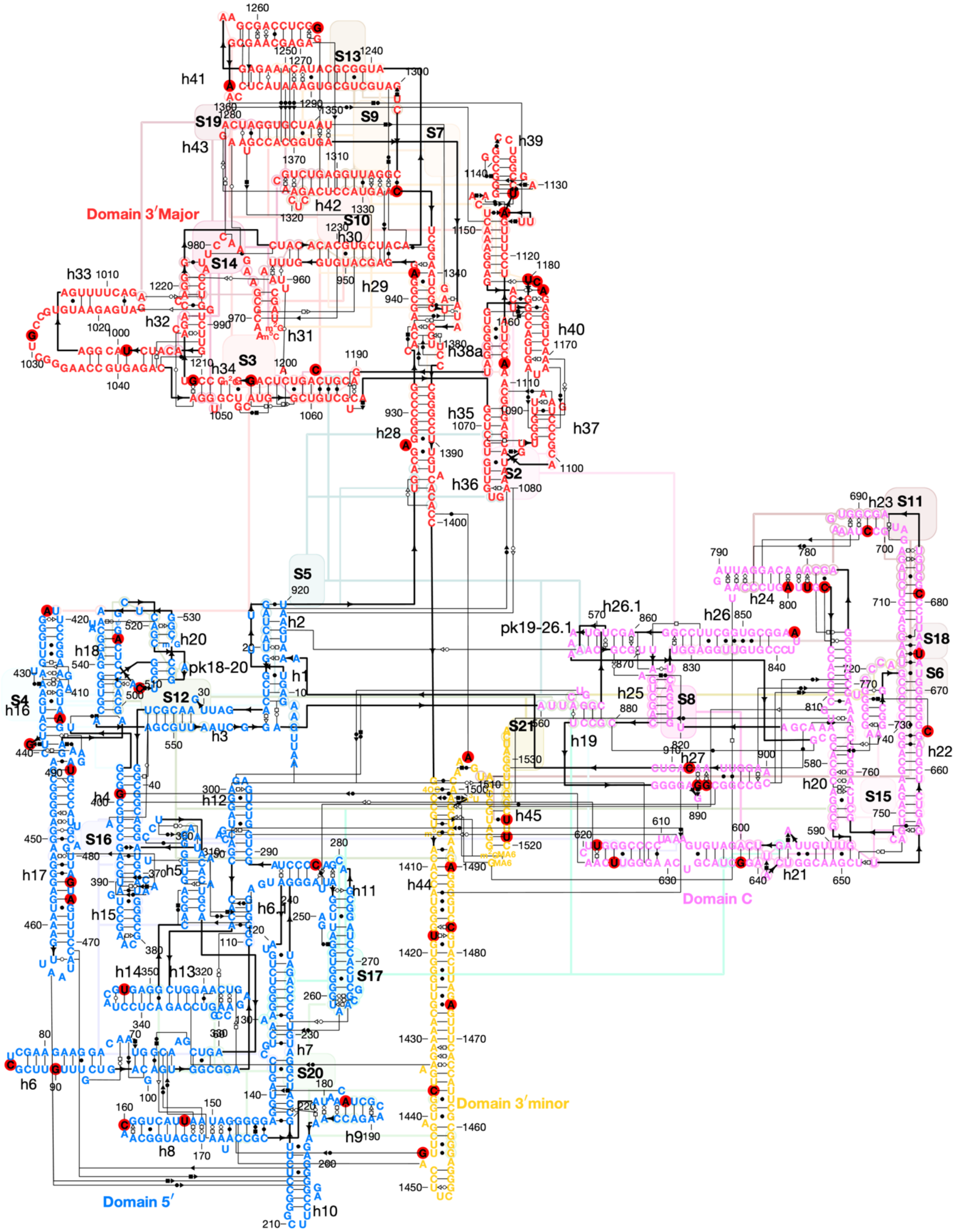

Supplementary Figure 21. Computationally predicted ribosomal RNA design CP-05 prepared with RiboDraw[1].

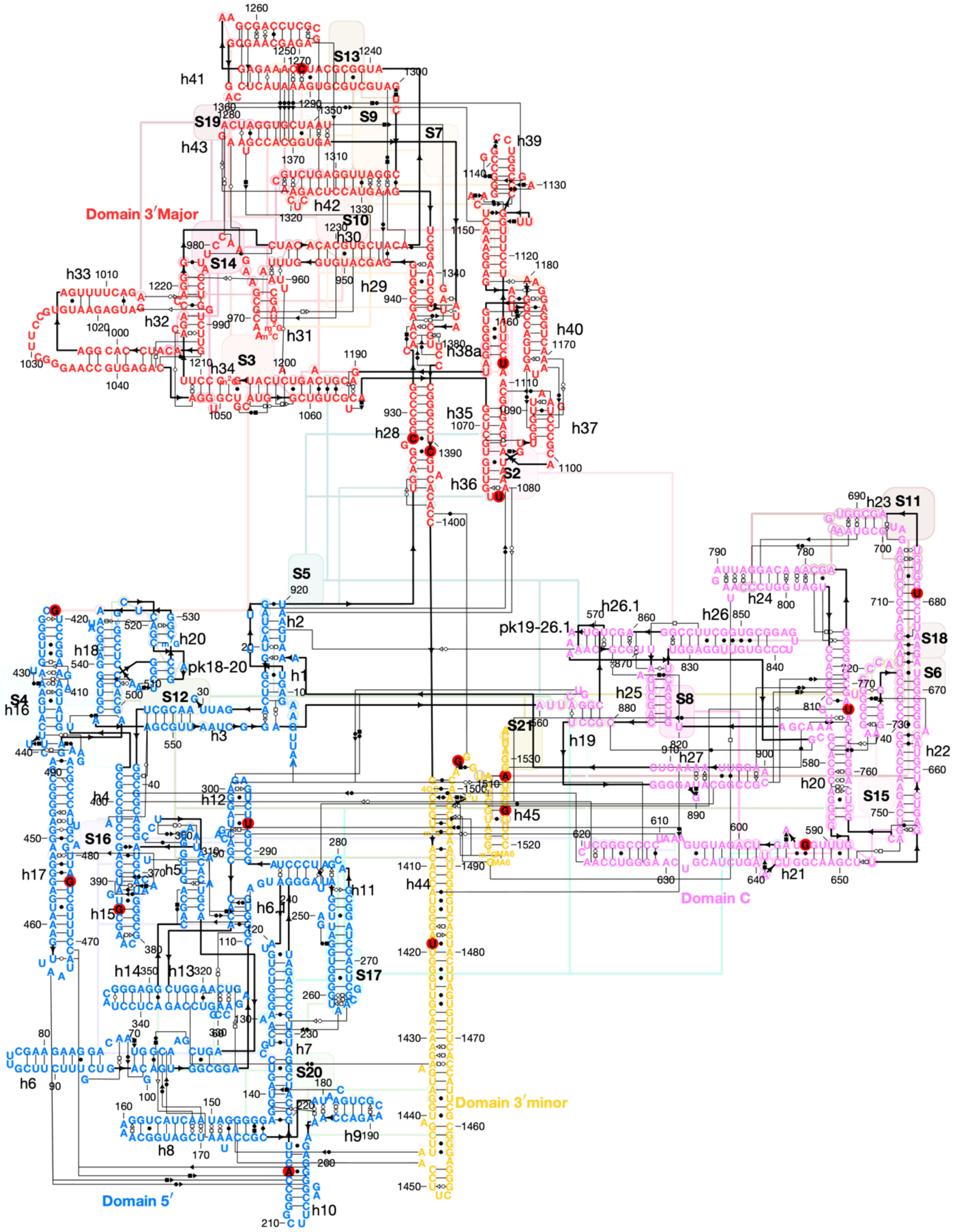

Supplementary Figure 22. Computationally predicted ribosomal RNA design CP-06 prepared with RiboDraw[1].

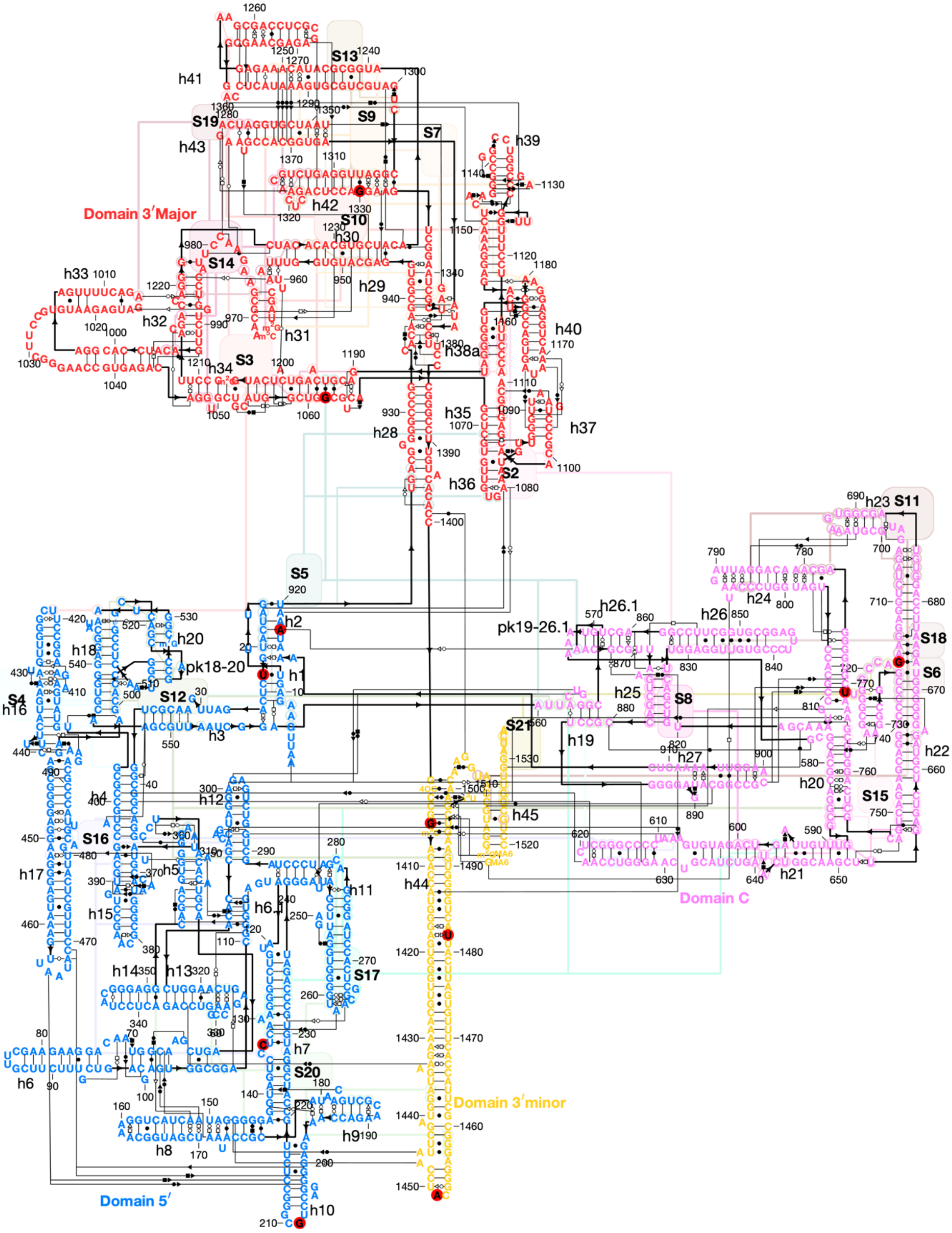

Supplementary Figure 23. Computationally predicted ribosomal RNA design CP-07 prepared with RiboDraw[1].

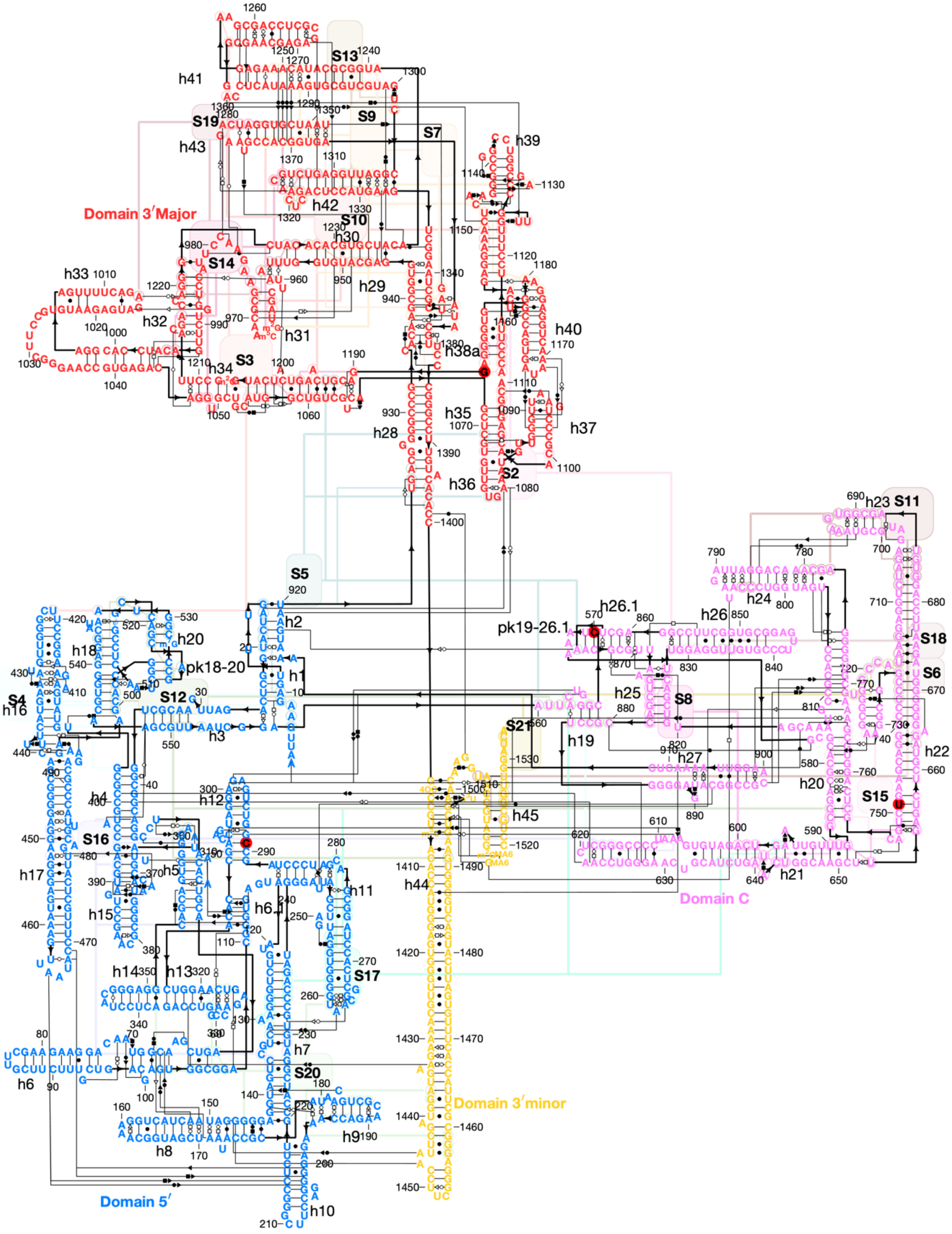

Supplementary Figure 24. Computationally predicted ribosomal RNA design CP-08 prepared with RiboDraw[1].

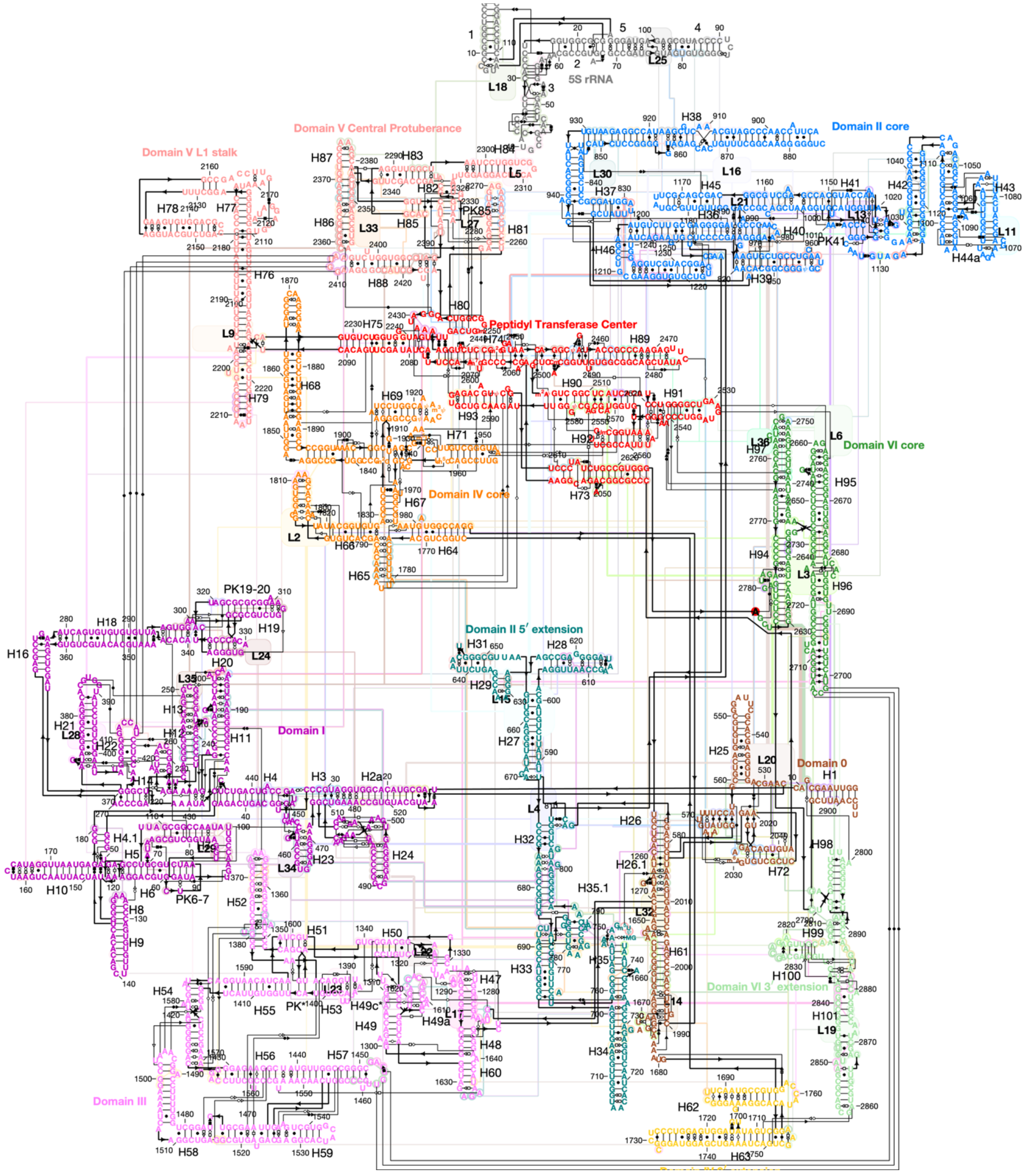

Supplementary Figure 25. Computationally predicted ribosomal RNA design CP-09 prepared with RiboDraw[1].

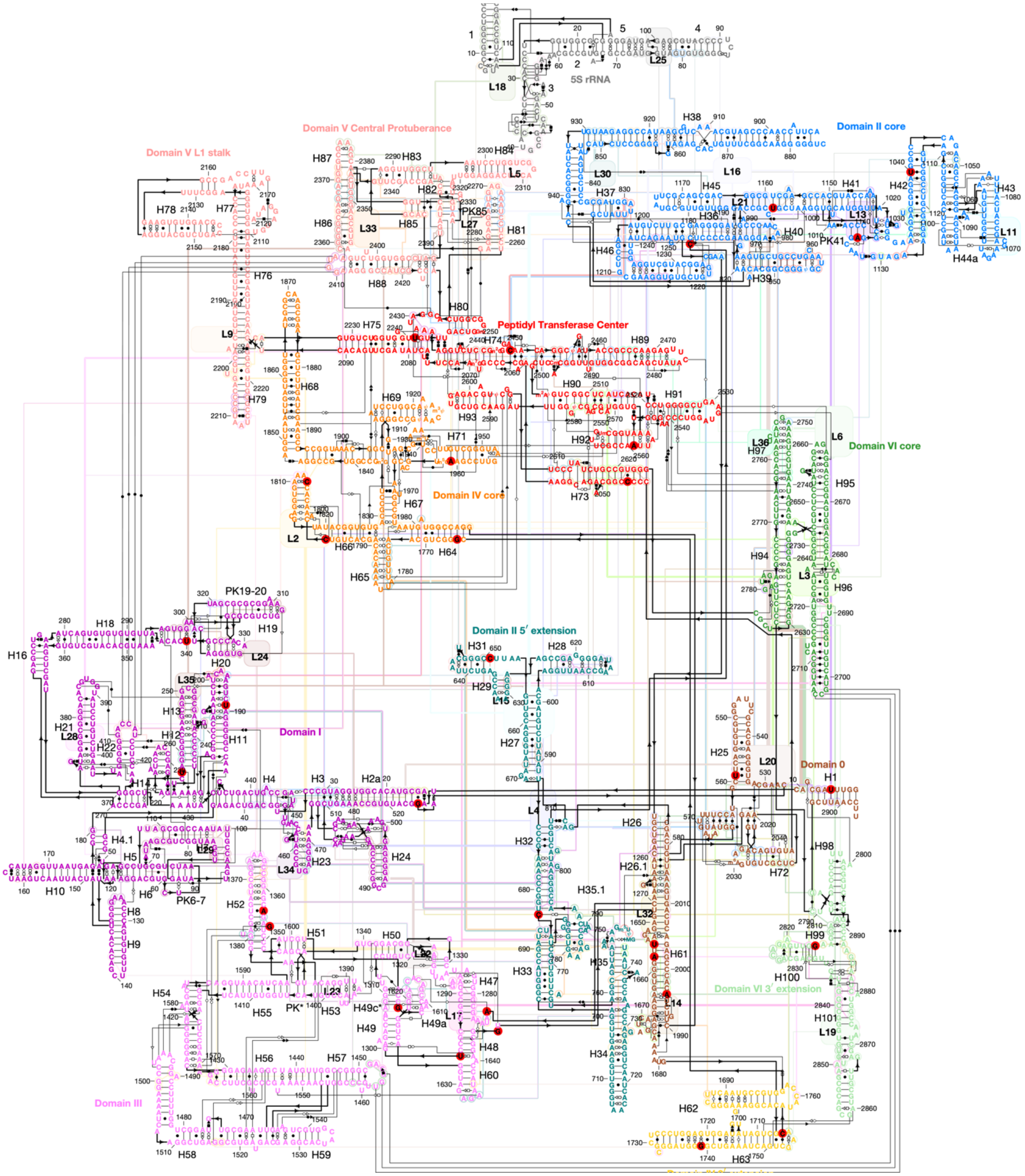

Supplementary Figure 26. Computationally predicted ribosomal RNA design CP-10 prepared with RiboDraw[1].

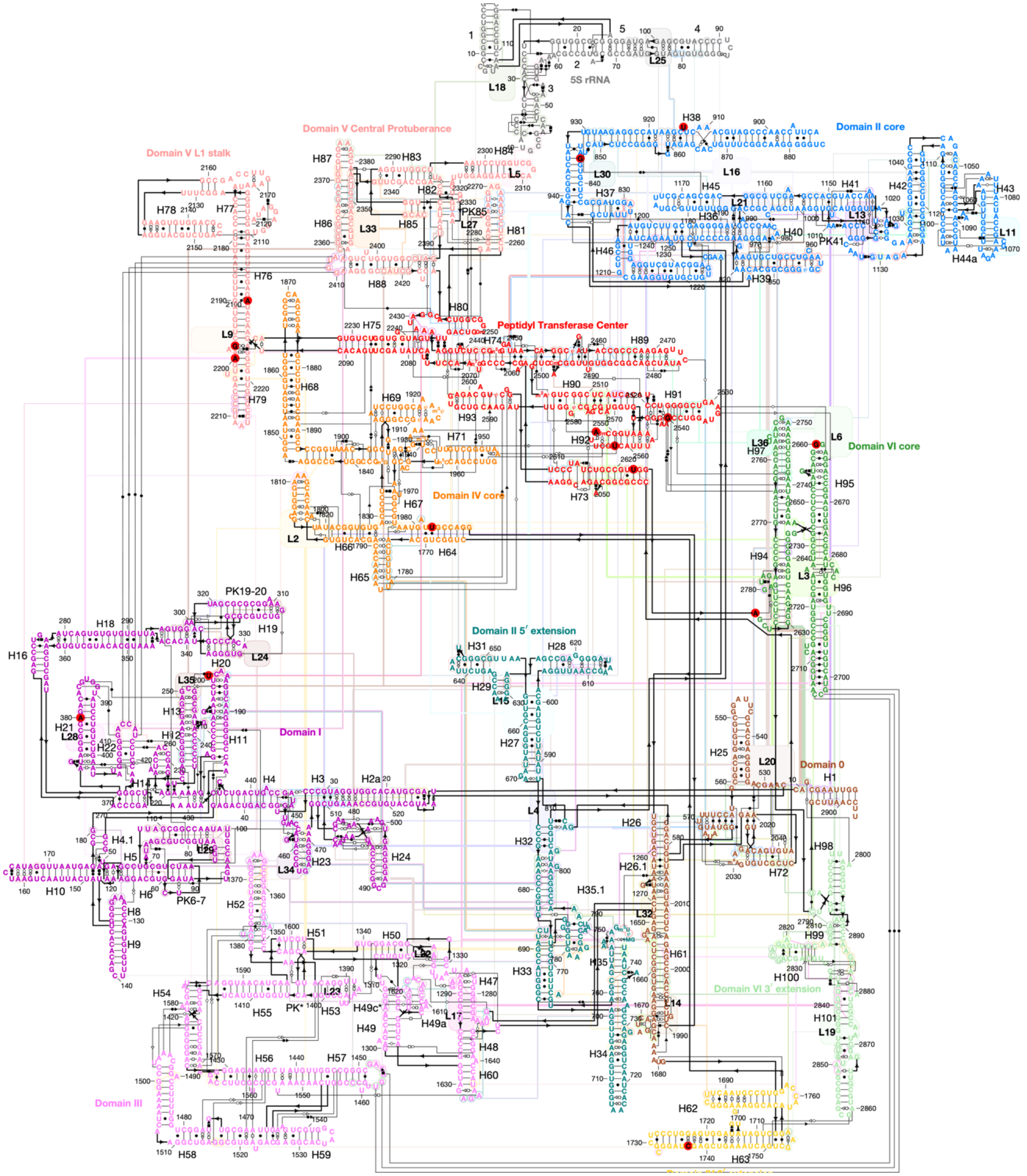

Supplementary Figure 27. Computationally predicted ribosomal RNA design CP-11 prepared with RiboDraw[1].

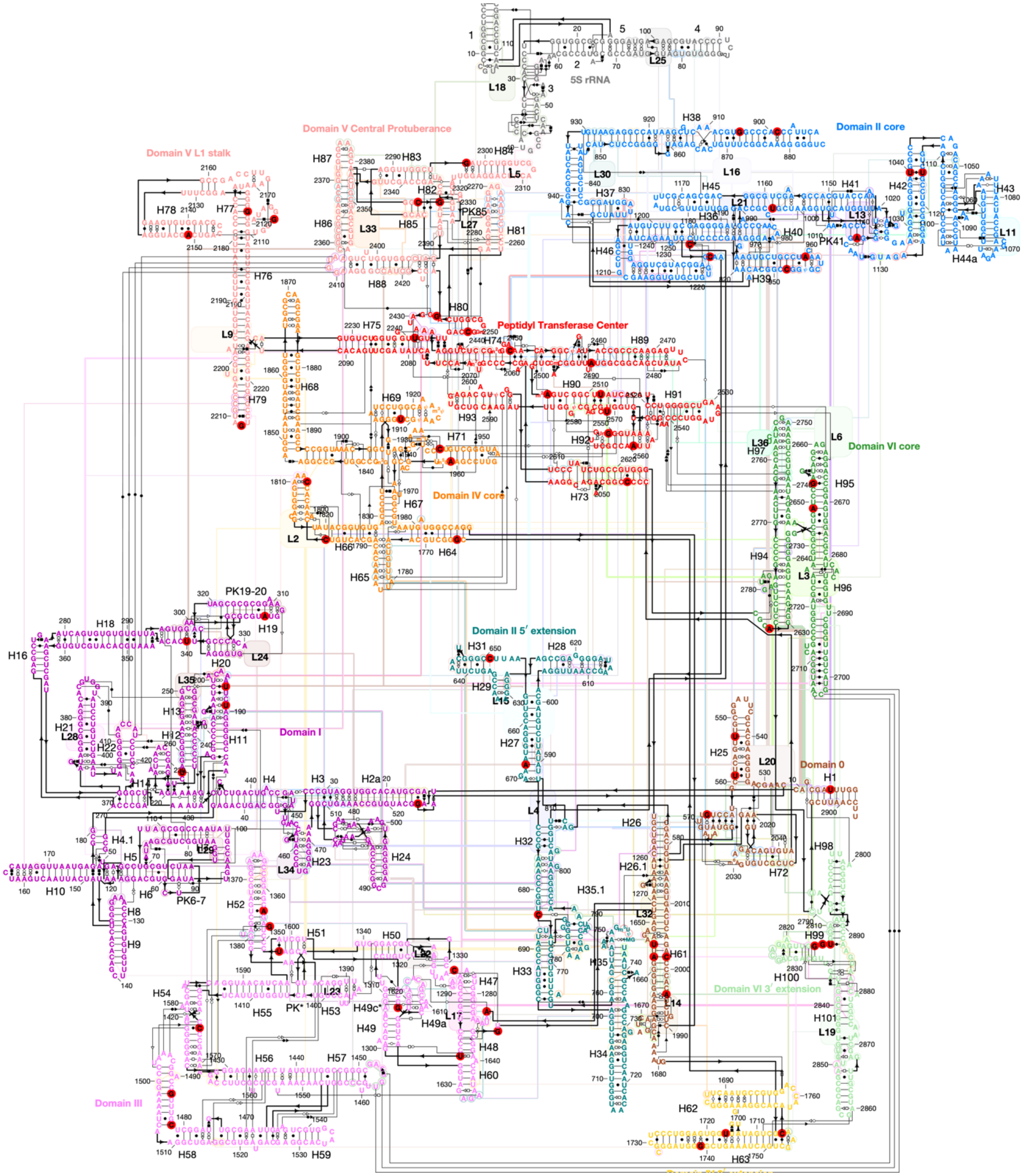

Supplementary Figure 28. Computationally predicted ribosomal RNA design CP-12 prepared with RiboDraw[1].

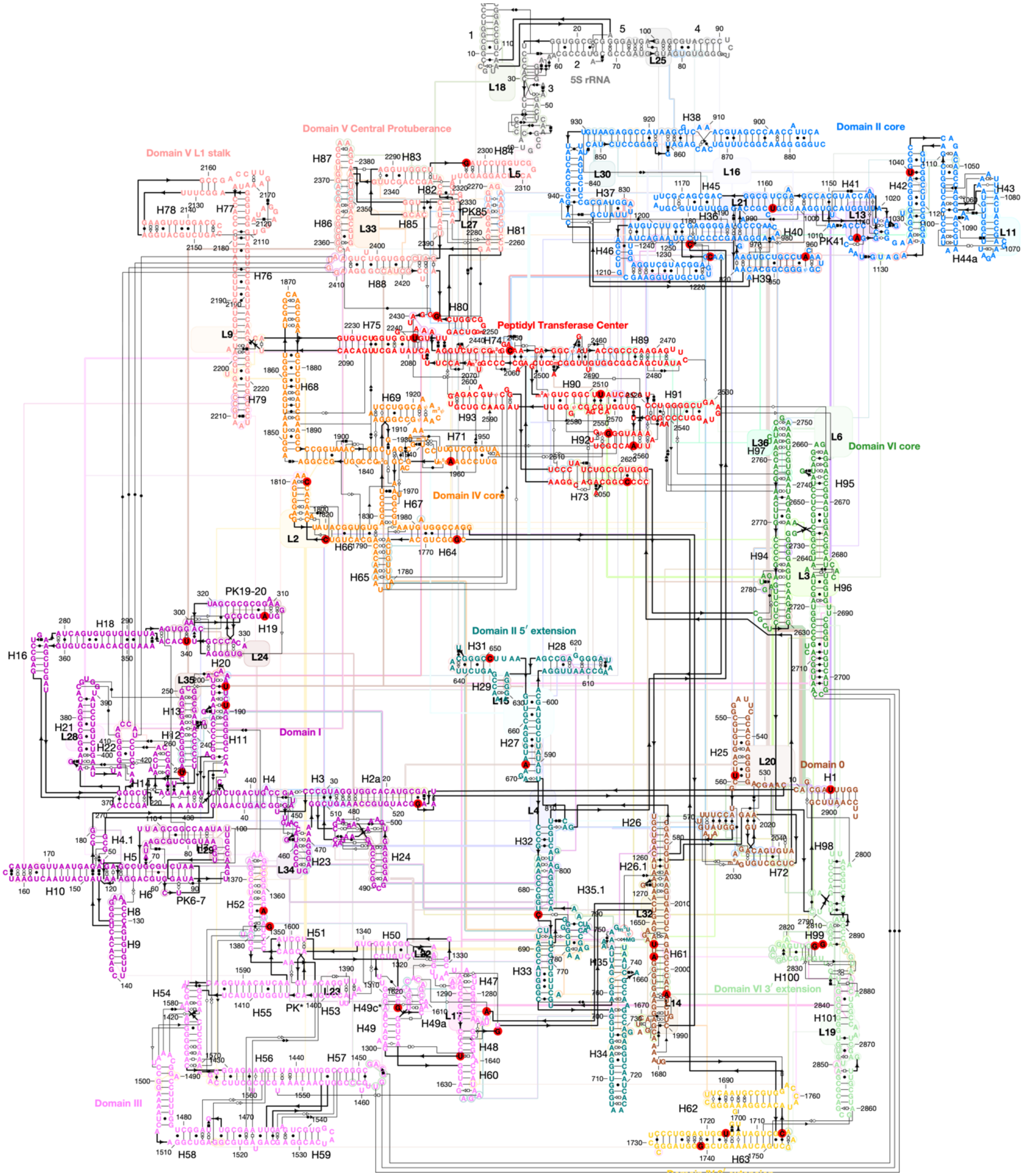

Supplementary Figure 29. Computationally predicted ribosomal RNA design CP-13 prepared with RiboDraw[1].

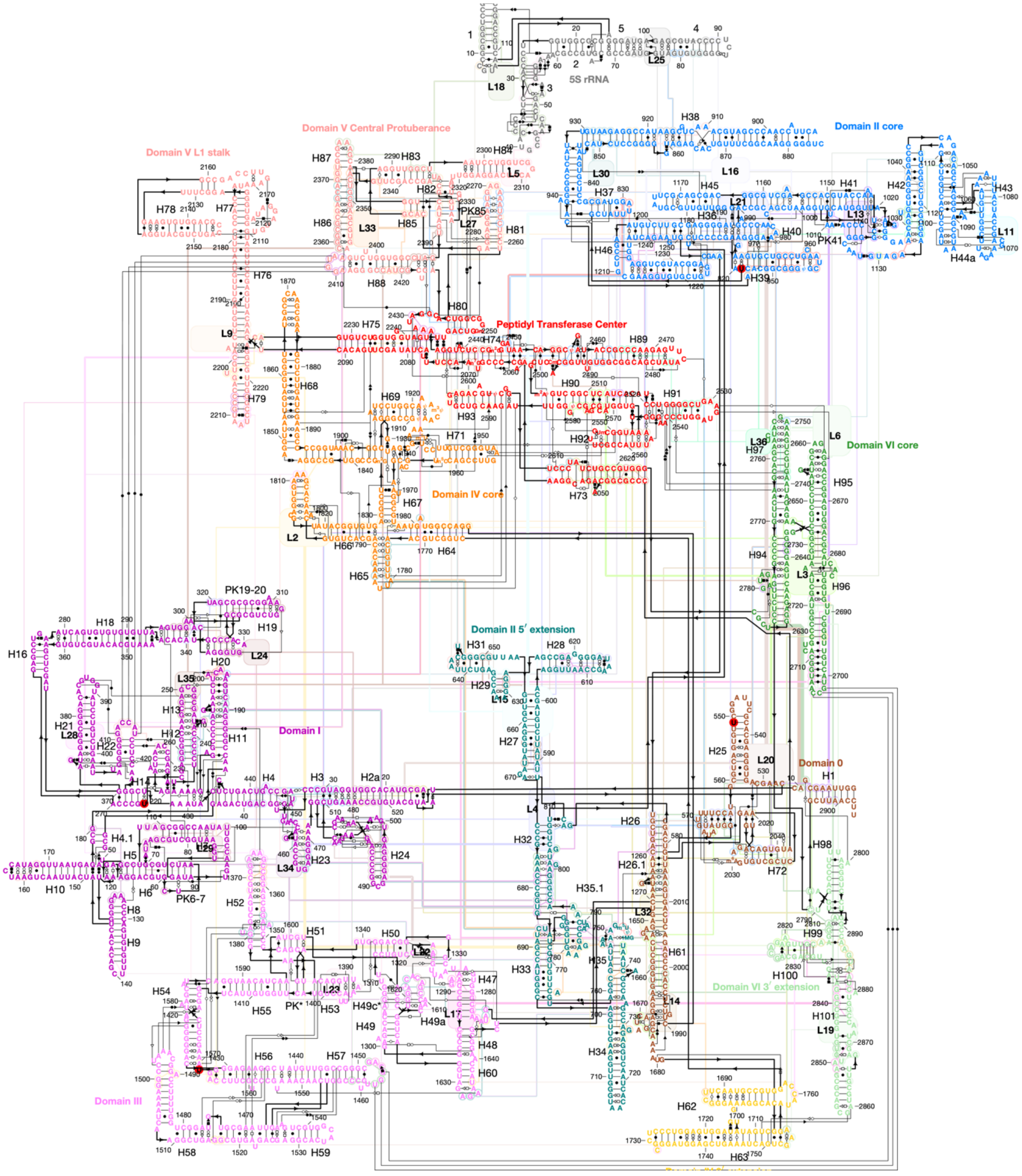

Supplementary Figure 30. Computationally predicted ribosomal RNA design CP-14 prepared with RiboDraw[1].

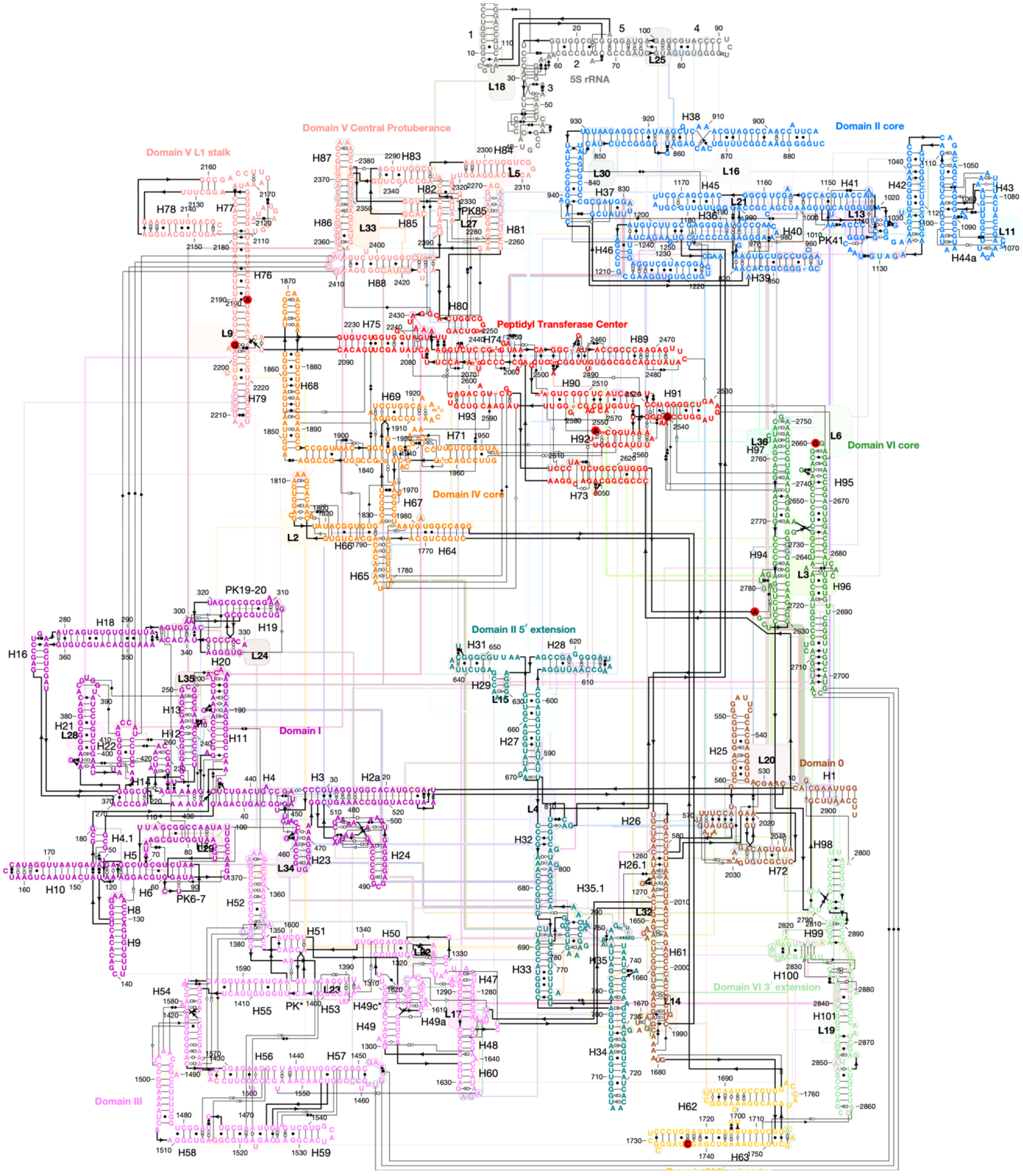

Supplementary Figure 31. Computationally predicted ribosomal RNA design CP-15 prepared with RiboDraw[1].

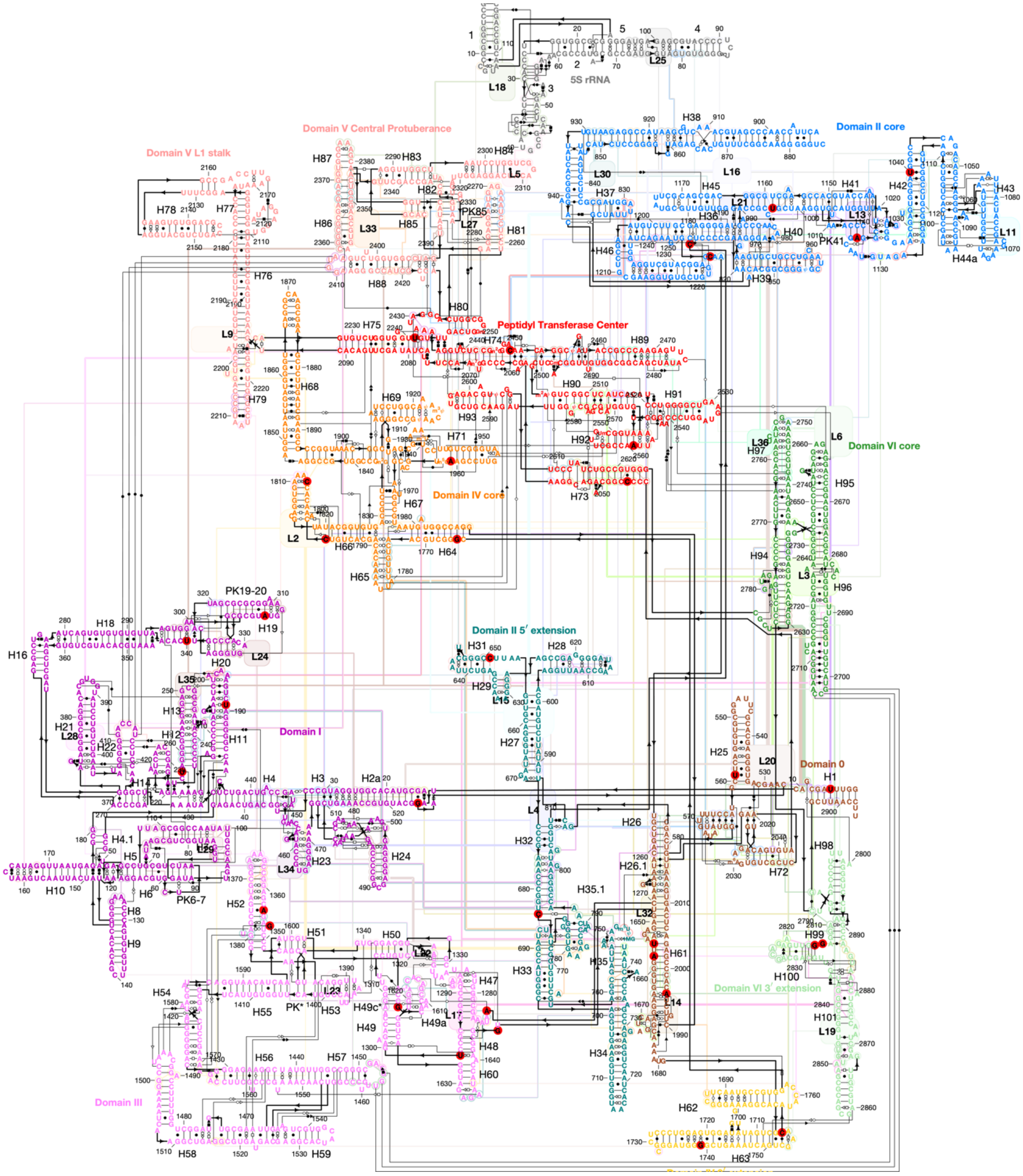

Supplementary Figure 32. Computationally predicted ribosomal RNA design CP-16 prepared with RiboDraw[1].

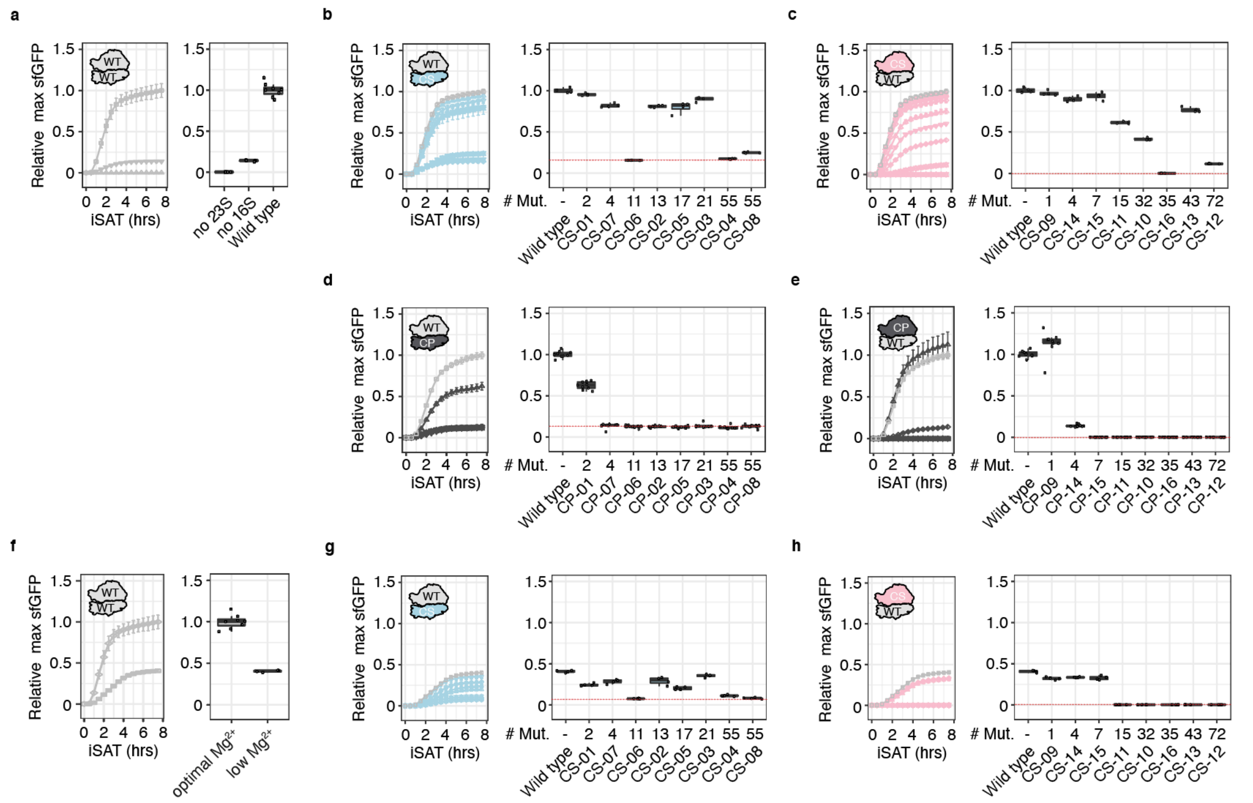

Supplementary Figure 33. sfGFP expression of 16S and 23S rRNA designs during iSAT at optimal and folding stress conditions. (**a**) Relative sfGFP expression in iSAT at optimal conditions of pT7-rrnB-wild type, pT7-rrnB-Δ23S, and pT7-rrnB-Δ16S. Community scientist-designed (CS) 16S rRNA (**b**) and 23S rRNA (**c**) designs. Computationally predicted (CP) 16S rRNA (**d**) and 23S rRNA (**e**). Relative sfGFP expression of wild type ribosomes (**f**) under optimal and low (3.75 mM)-magnesium (Mg^{2+}) iSAT conditions. Performance of Eterna 16S rRNAs (**g**) and 23S rRNAs (**h**) at low magnesium iSAT conditions. sfGFP expression in iSAT was determined by fluorescence over the course of 8 hours and normalized to the maximum sfGFP made by pT7-rrnB- wild type at optimal iSAT conditions. Time course data are shown as mean ± s.d. on the left of each panel and the relative max sfGFP generated by each design as boxplots on its right side. Error bars represent s.d.; n ≥ 4. Source data are provided as a Source Data file. The dotted red line indicates background activity arising from the extract. Mut: mutations; WT: wild type.

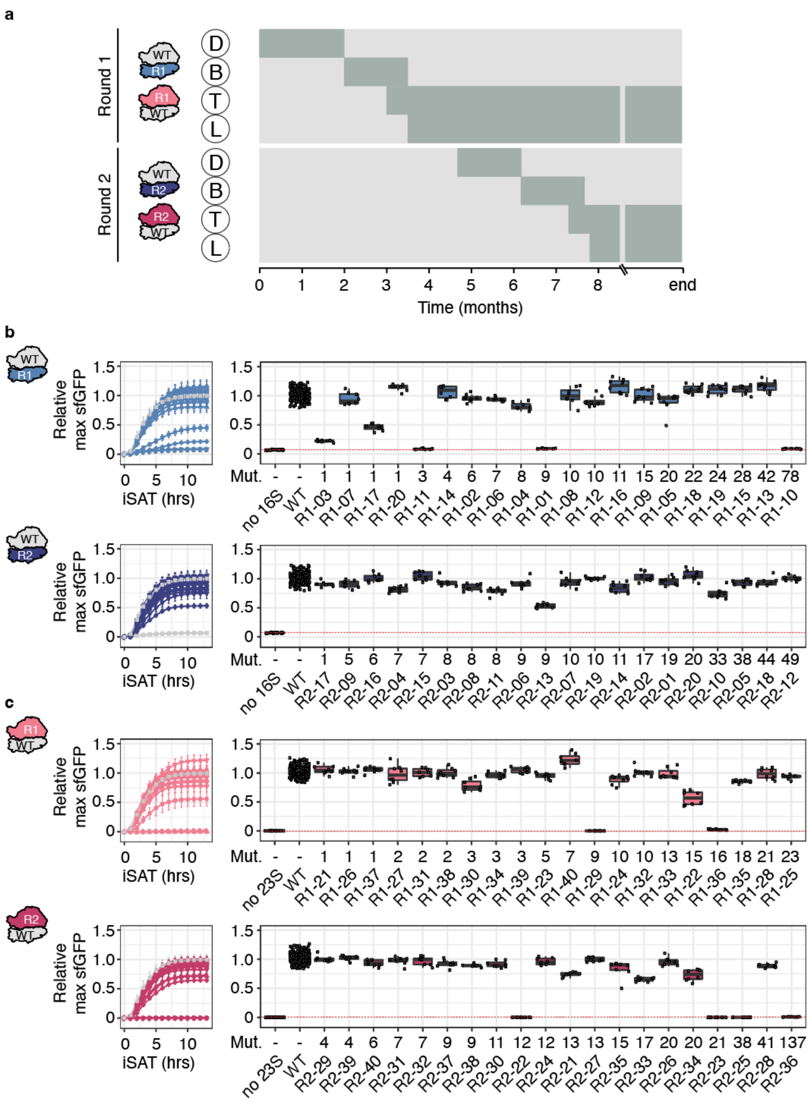

Supplementary Figure 34. Timeline for Design-Build-Test-Learn (DBTL) cycles for rRNA re-engineering by community scientists and iSAT time courses of Round (R1) and Round 2 (R2) Eterna designed ribosomes. (**a**) DBTL timeline for engineering rRNA by community scientist over the course of two DBTL rounds. (**b, c**) sfGFP expression of pT7-rrnB-R1 and pT7-rrnB-R2 16S rRNA (**b**) and pT7-rrnB-R1 and pT7-rrnB-R2 23S rRNA (**c**) designs for 16-hour iSAT reactions. sfGFP expression in iSAT was determined by fluorescence and normalized to max sfGFP of pT7-rrnB-wild type. Error bars represent s.d.; n ≥ 3. Source data are provided as a Source Data file. Dotted red line indicates background activity arising from the extract. D: design, B: build, T: test, L: learn, Mut: mutations, R1: round 1, R2: round 2, WT: wild type.

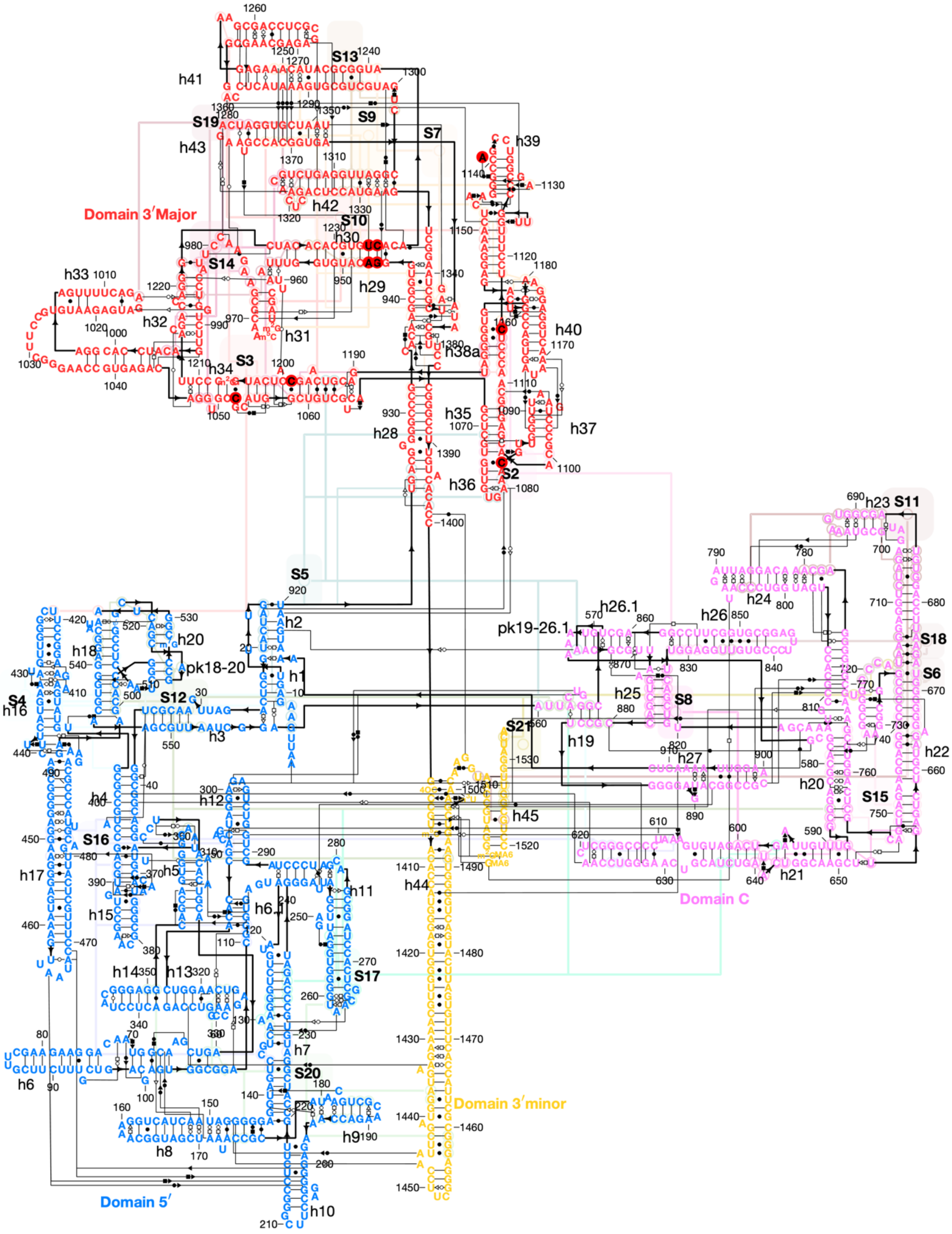

Supplementary Figure 35. Eterna participants'-designed ribosomal RNA design R1-01 prepared with RiboDraw[1].

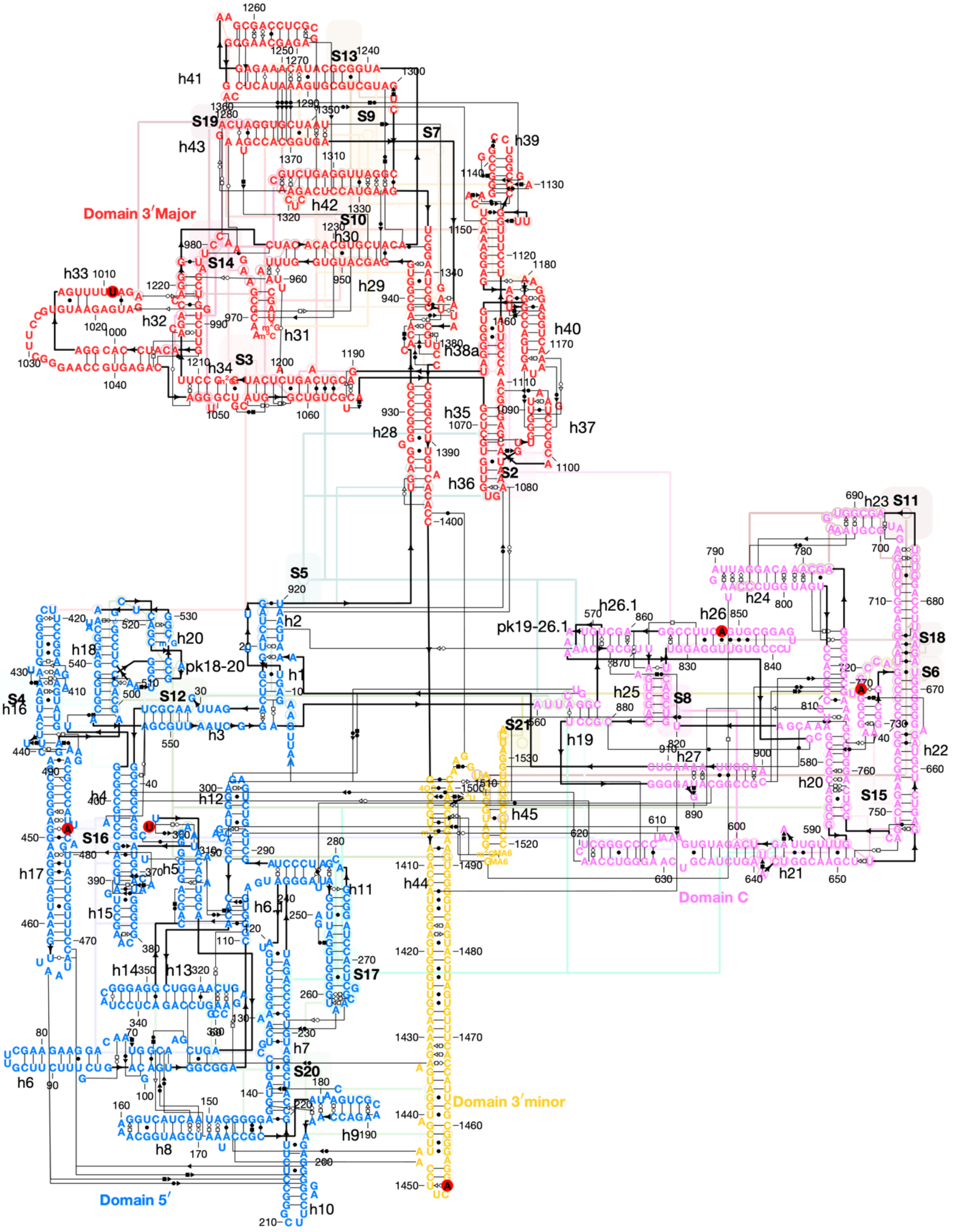

Supplementary Figure 36. Eterna participants'-designed ribosomal RNA design R1-02 prepared with RiboDraw[1].

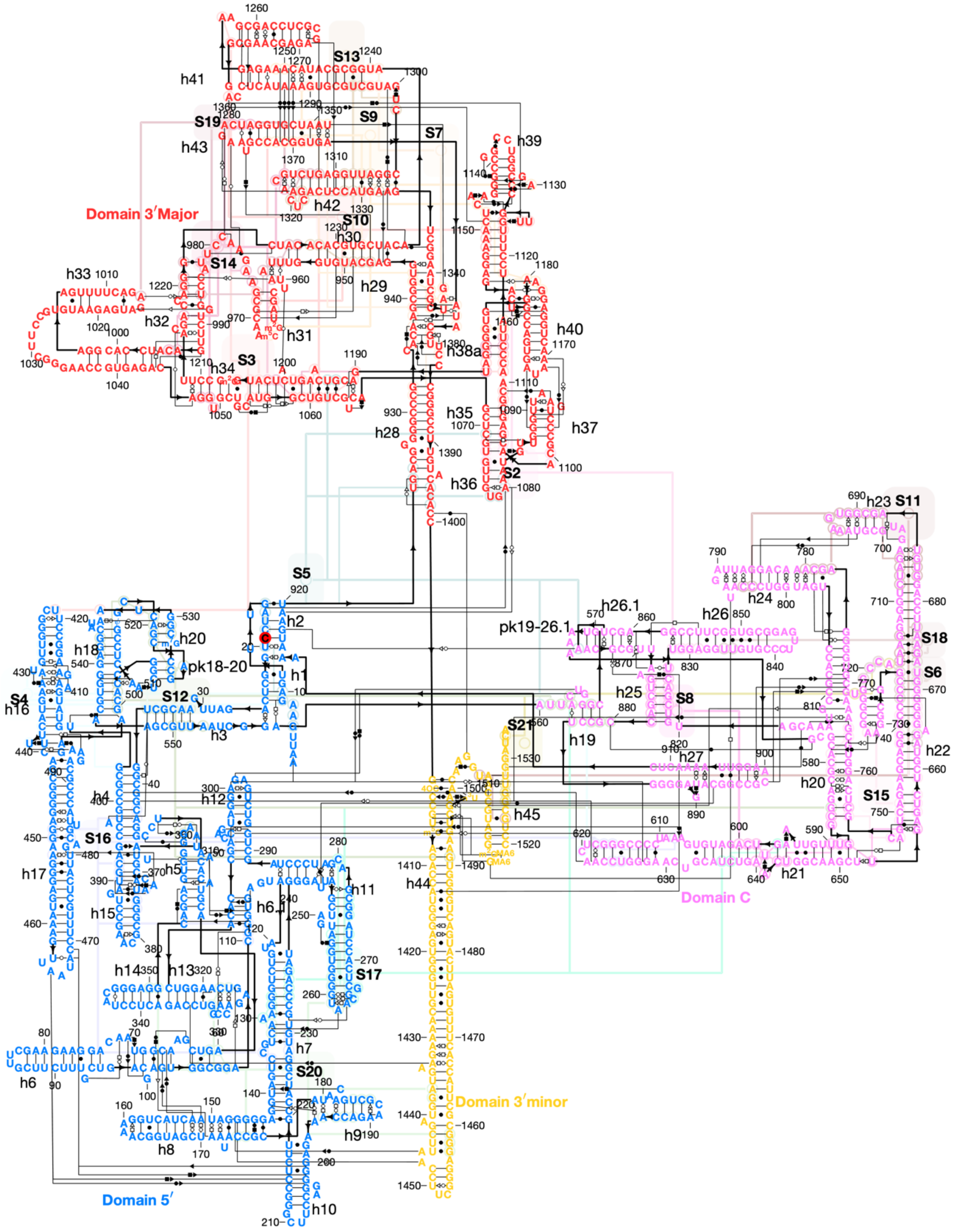

Supplementary Figure 37. Eterna participants'-designed ribosomal RNA design R1-03 prepared with RiboDraw[1].

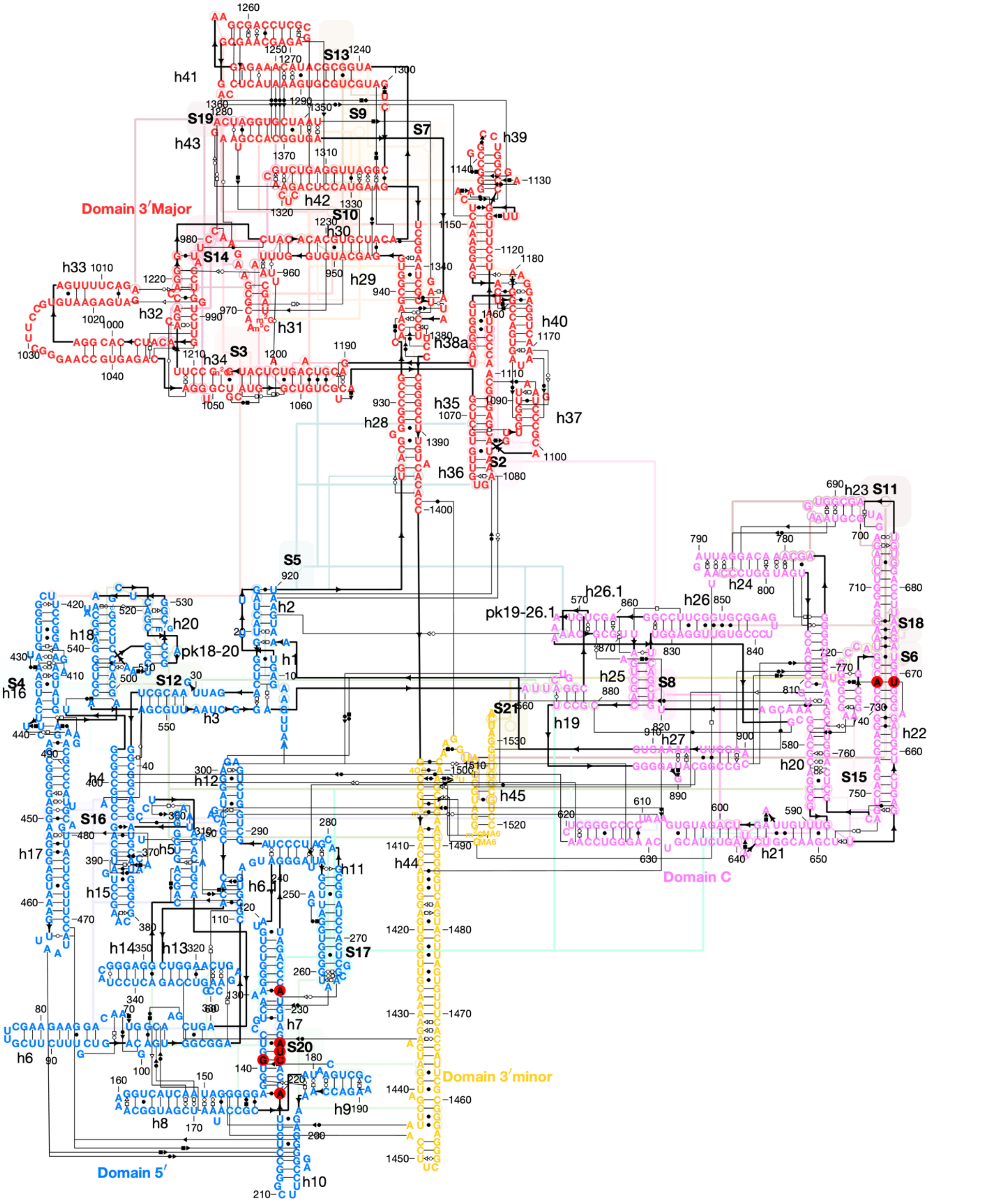

Supplementary Figure 38. Eterna participants'-designed ribosomal RNA design R1-04 prepared with RiboDraw[1].

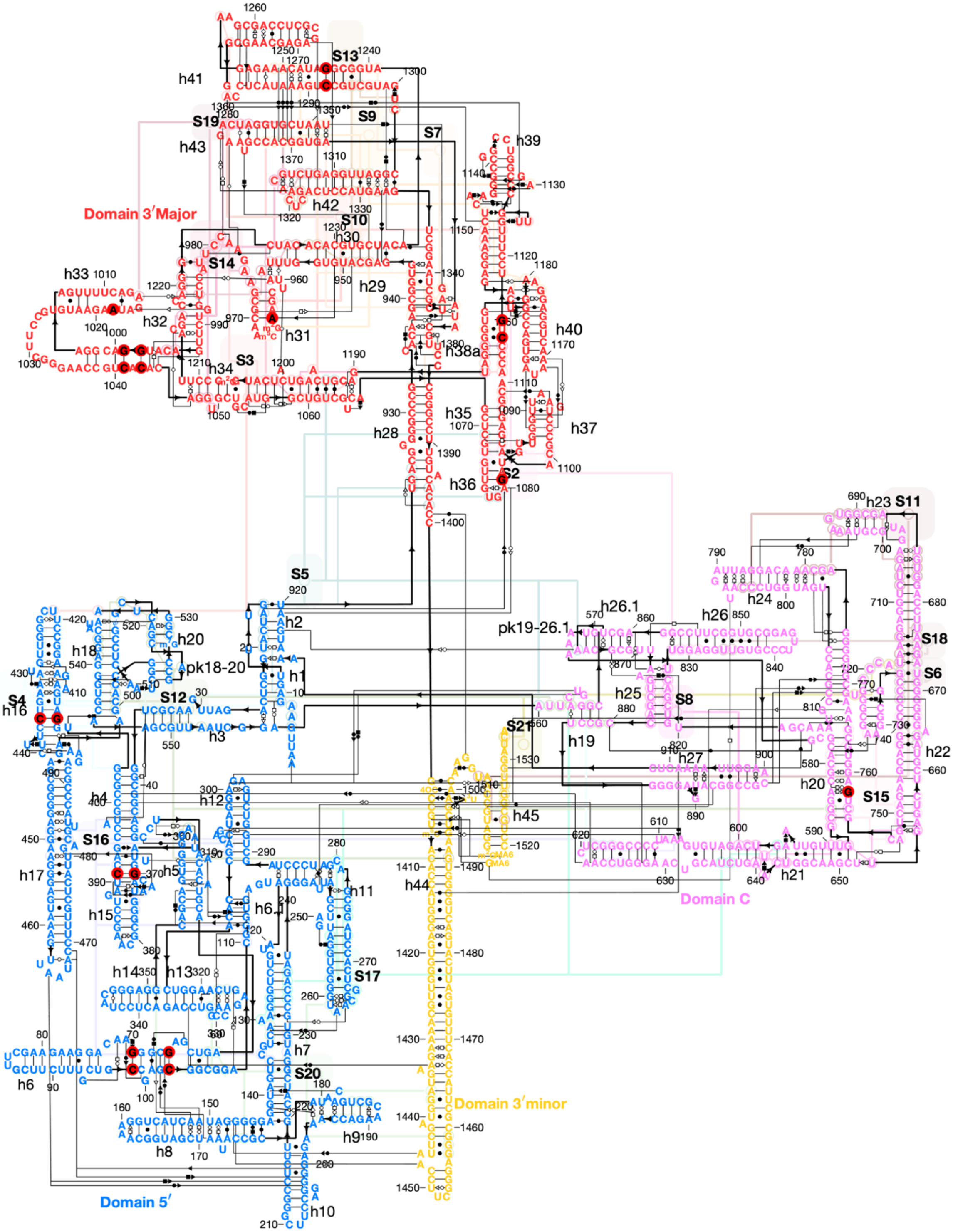

Supplementary Figure 39. Eterna participants'-designed ribosomal RNA design R1-05 prepared with RiboDraw[1].

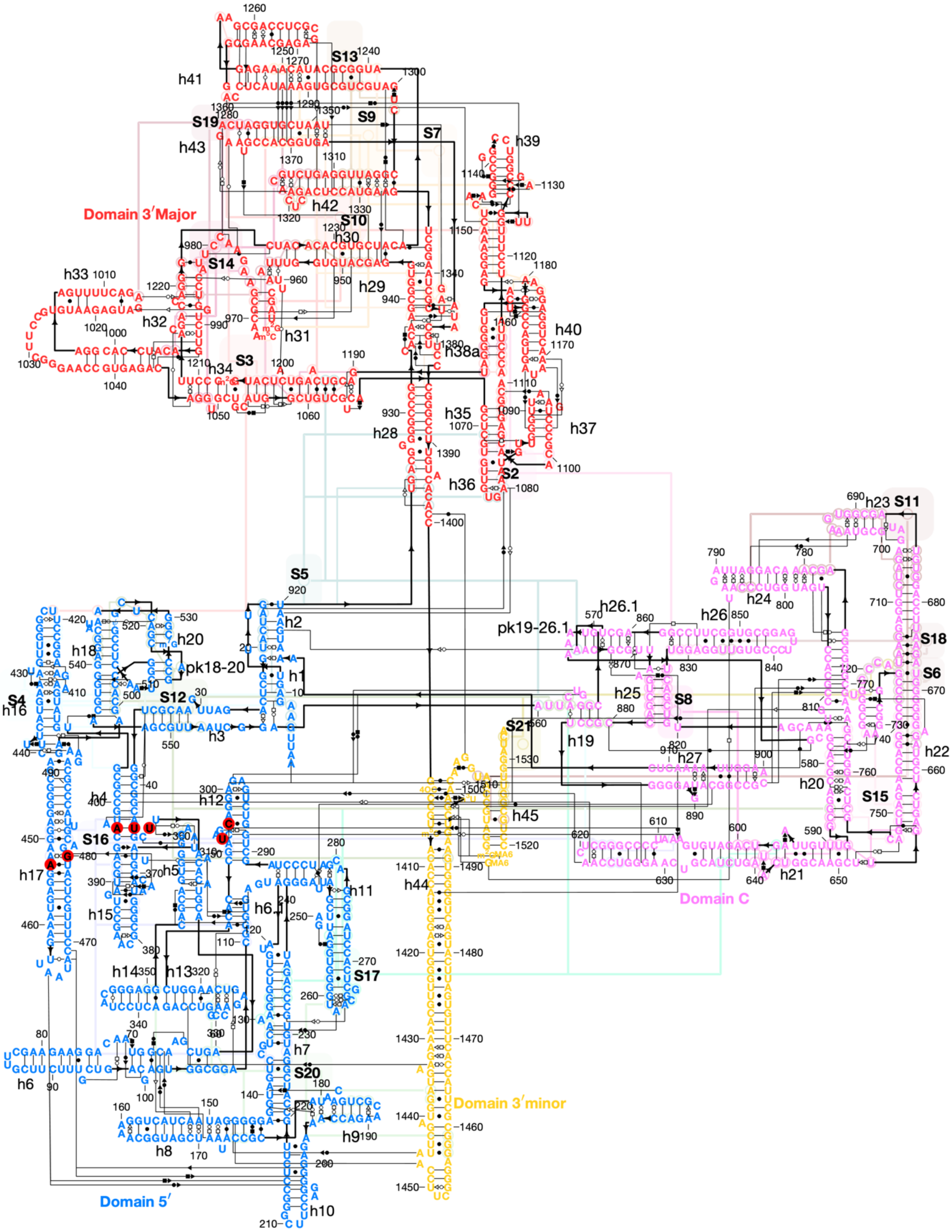

Supplementary Figure 40. Eterna participants'-designed ribosomal RNA design R1-06 prepared with RiboDraw[1].

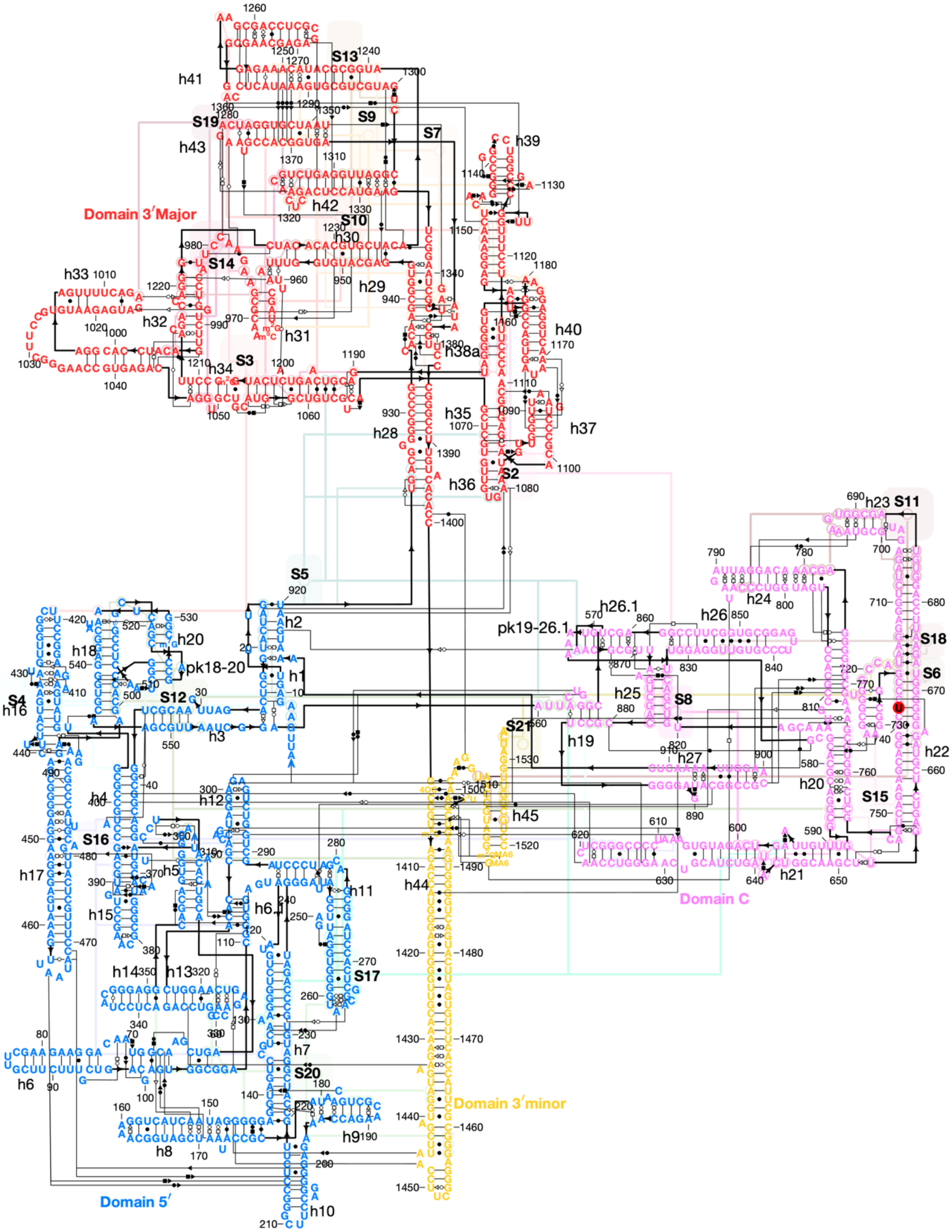

Supplementary Figure 41. Eterna participants'-designed ribosomal RNA design R1-07 prepared with RiboDraw[1].

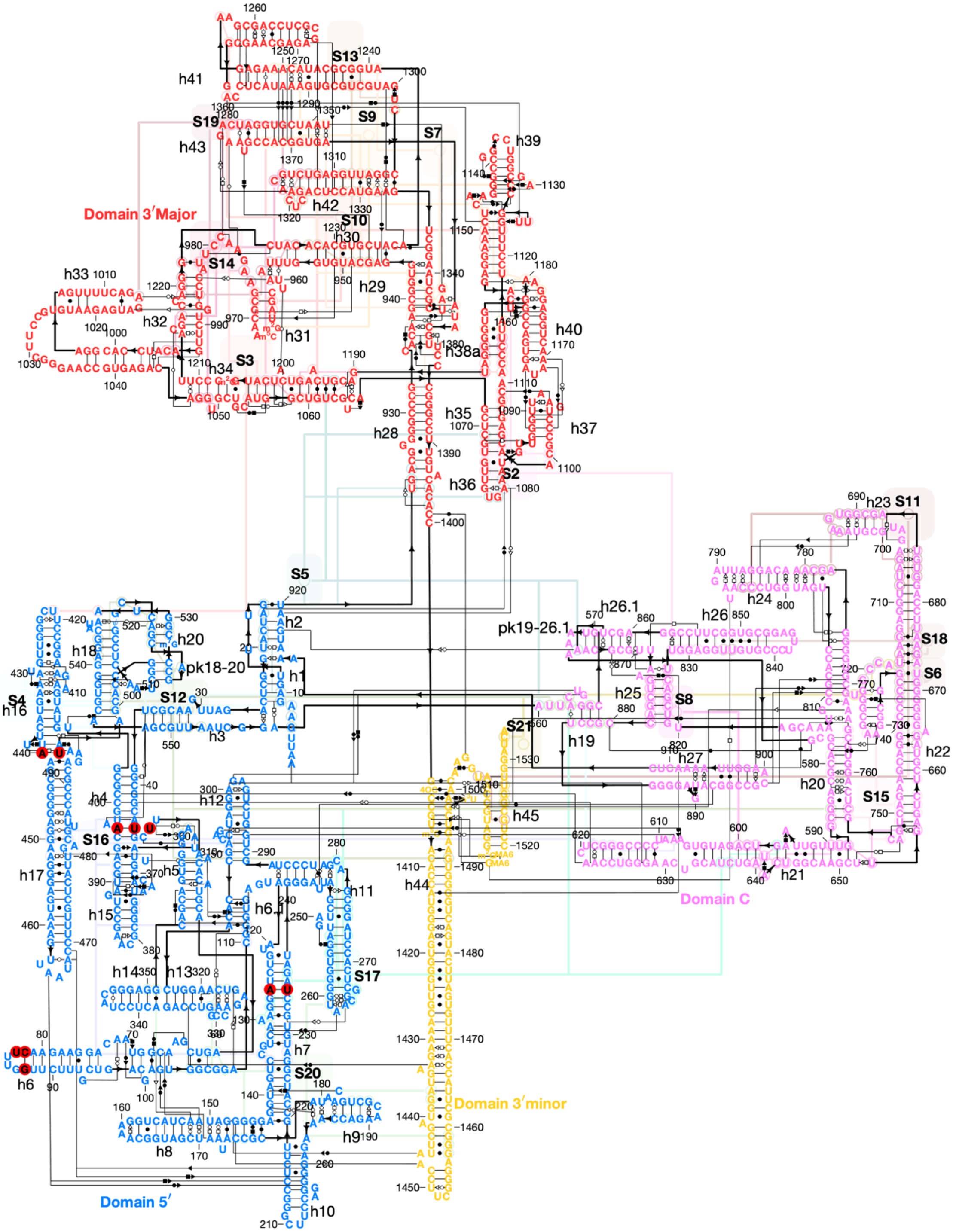

Supplementary Figure 42. Eterna participants'-designed ribosomal RNA design R1-08 prepared with RiboDraw[1].

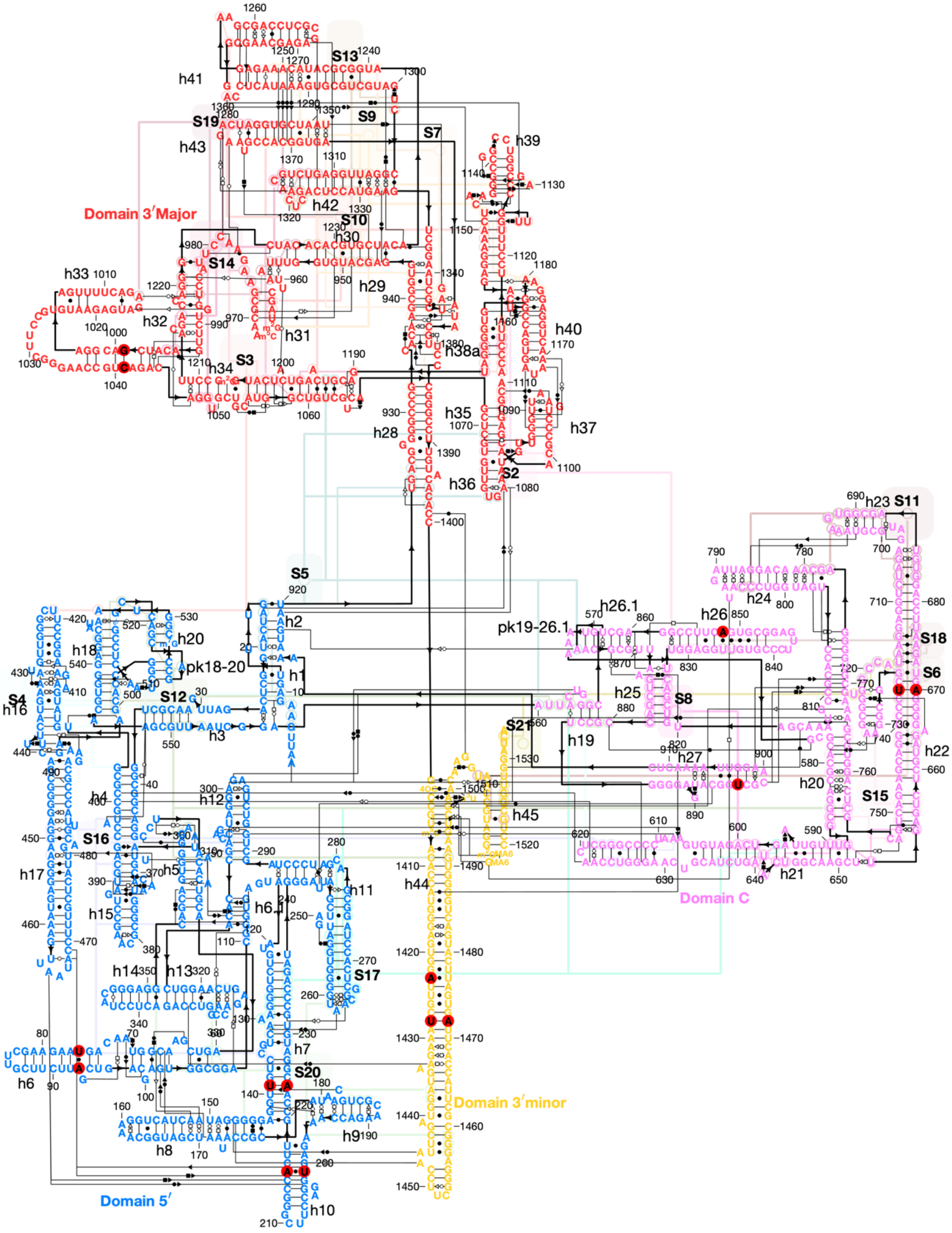

Supplementary Figure 43. Eterna participants'-designed ribosomal RNA design R1-09 prepared with RiboDraw[1].

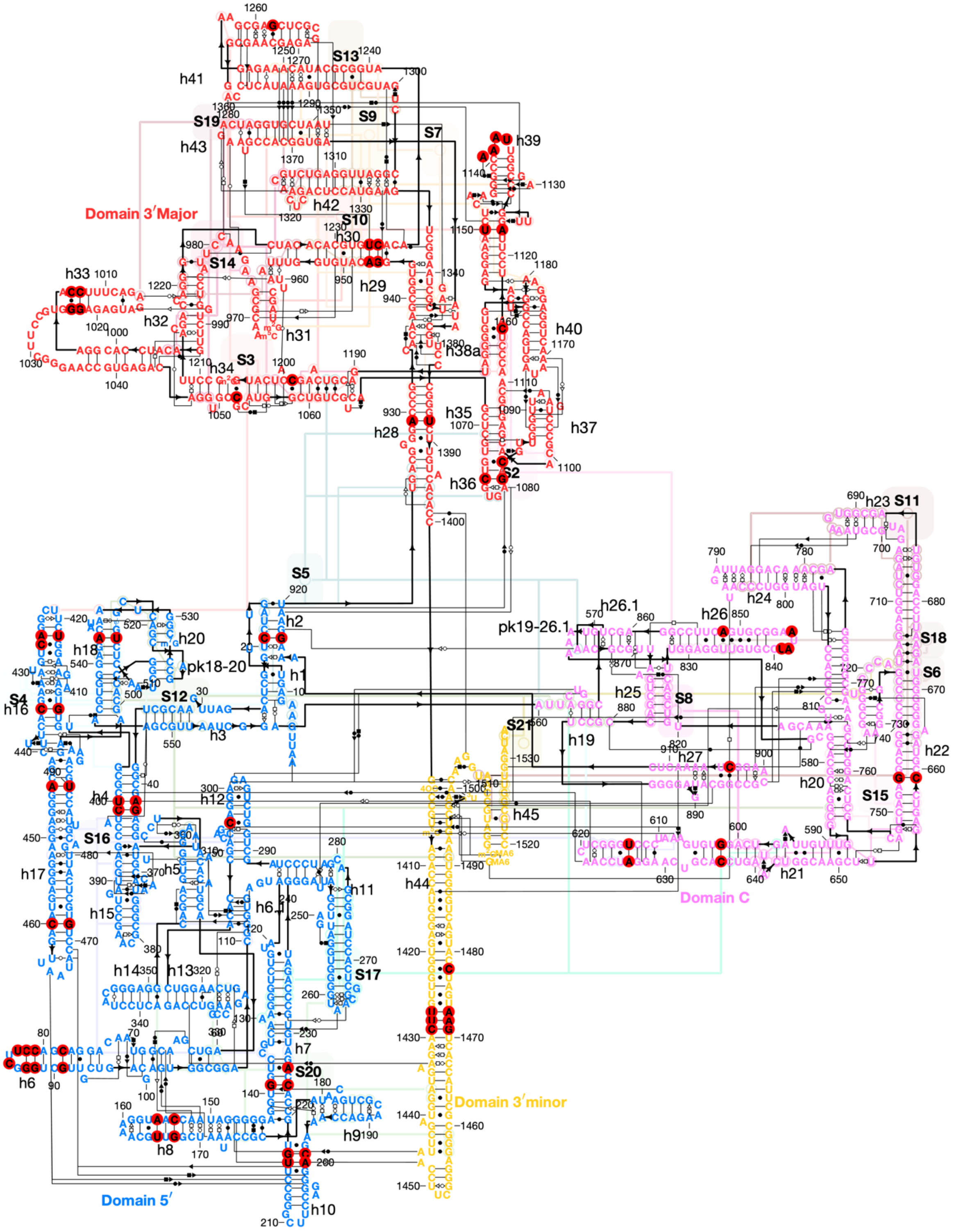

Supplementary Figure 44. Eterna participants'-designed ribosomal RNA design R1-10 prepared with RiboDraw[1].

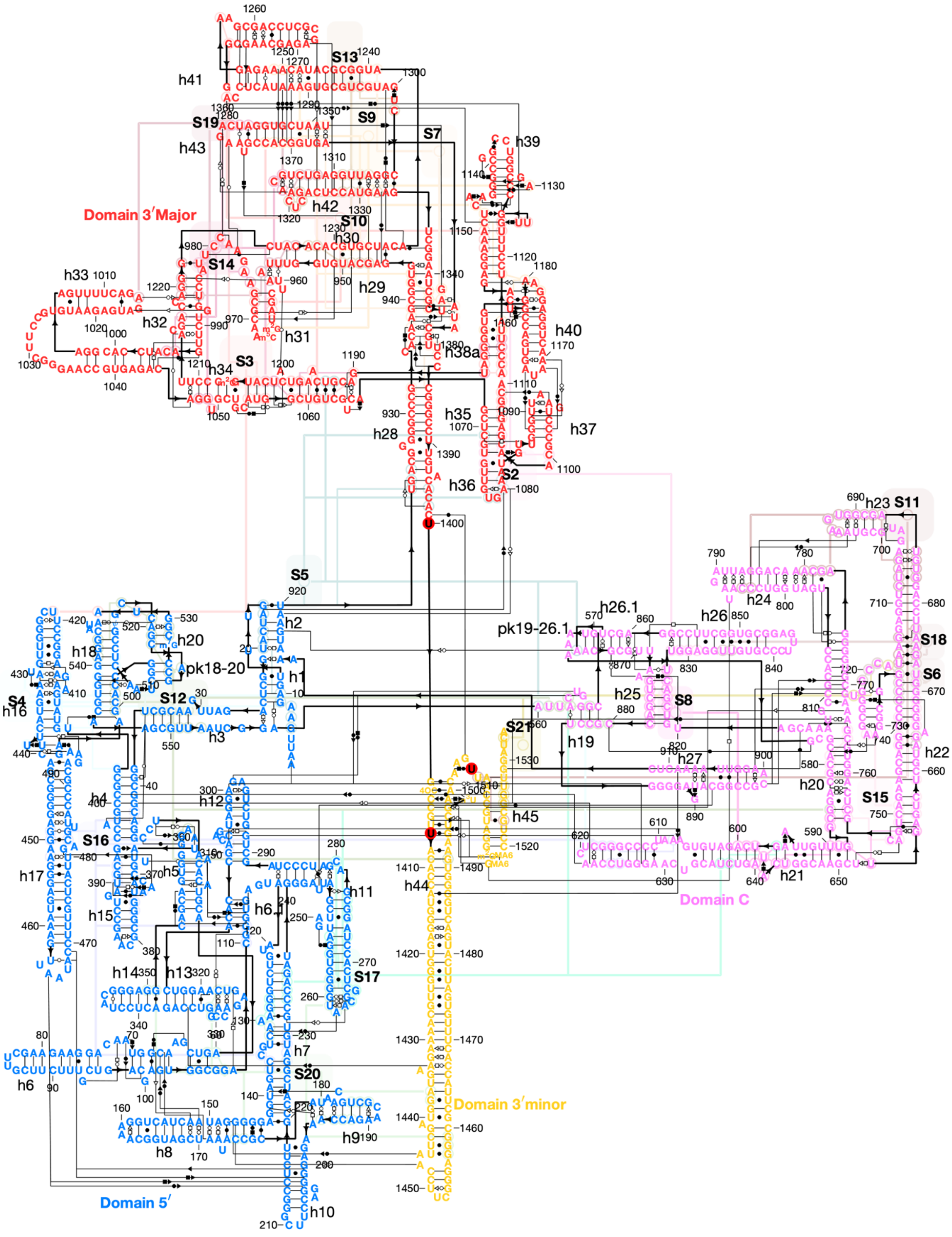

Supplementary Figure 45. Eterna participants'-designed ribosomal RNA design R1-11 prepared with RiboDraw[1].

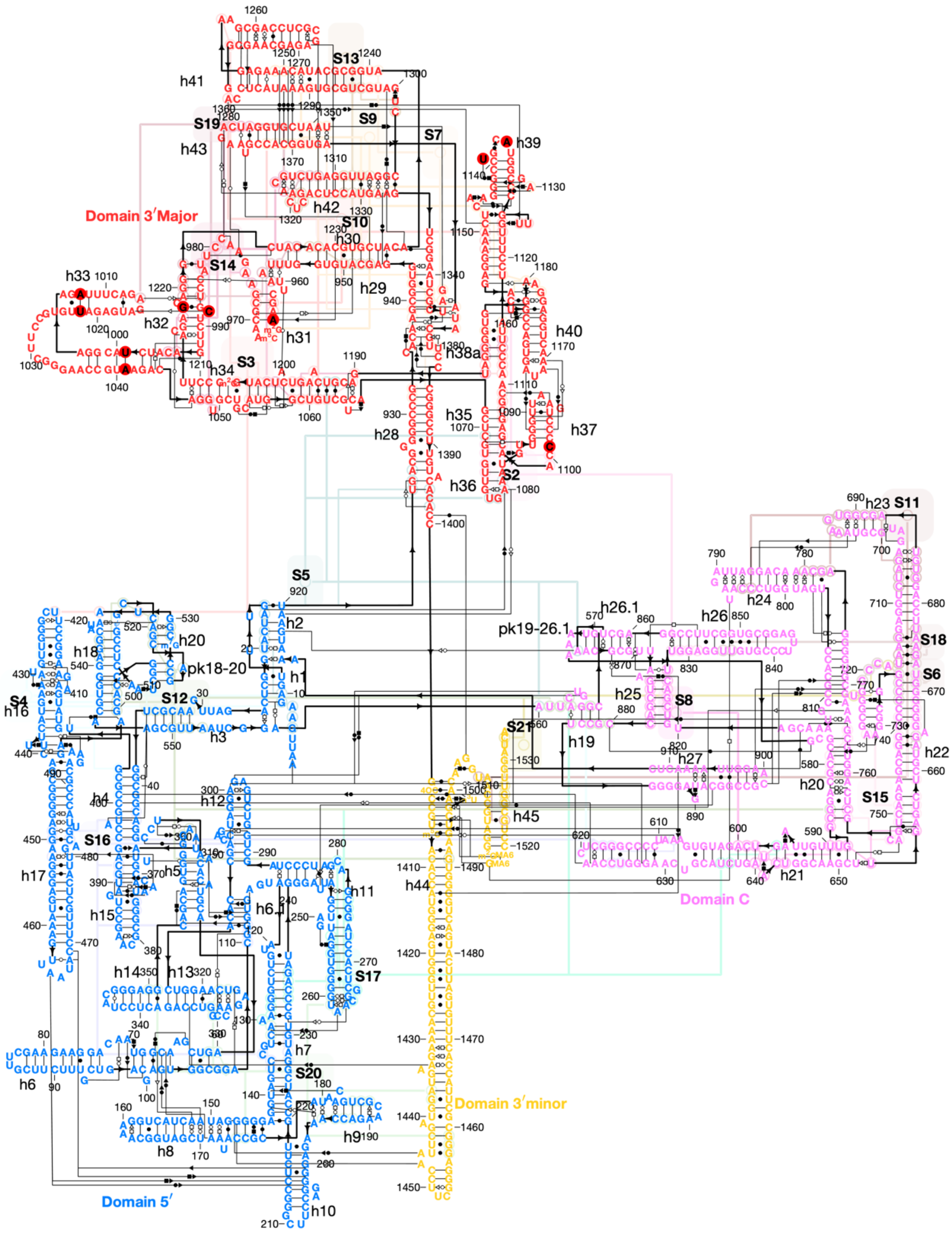

Supplementary Figure 46. Eterna participants'-designed ribosomal RNA design R1-12 prepared with RiboDraw[1].

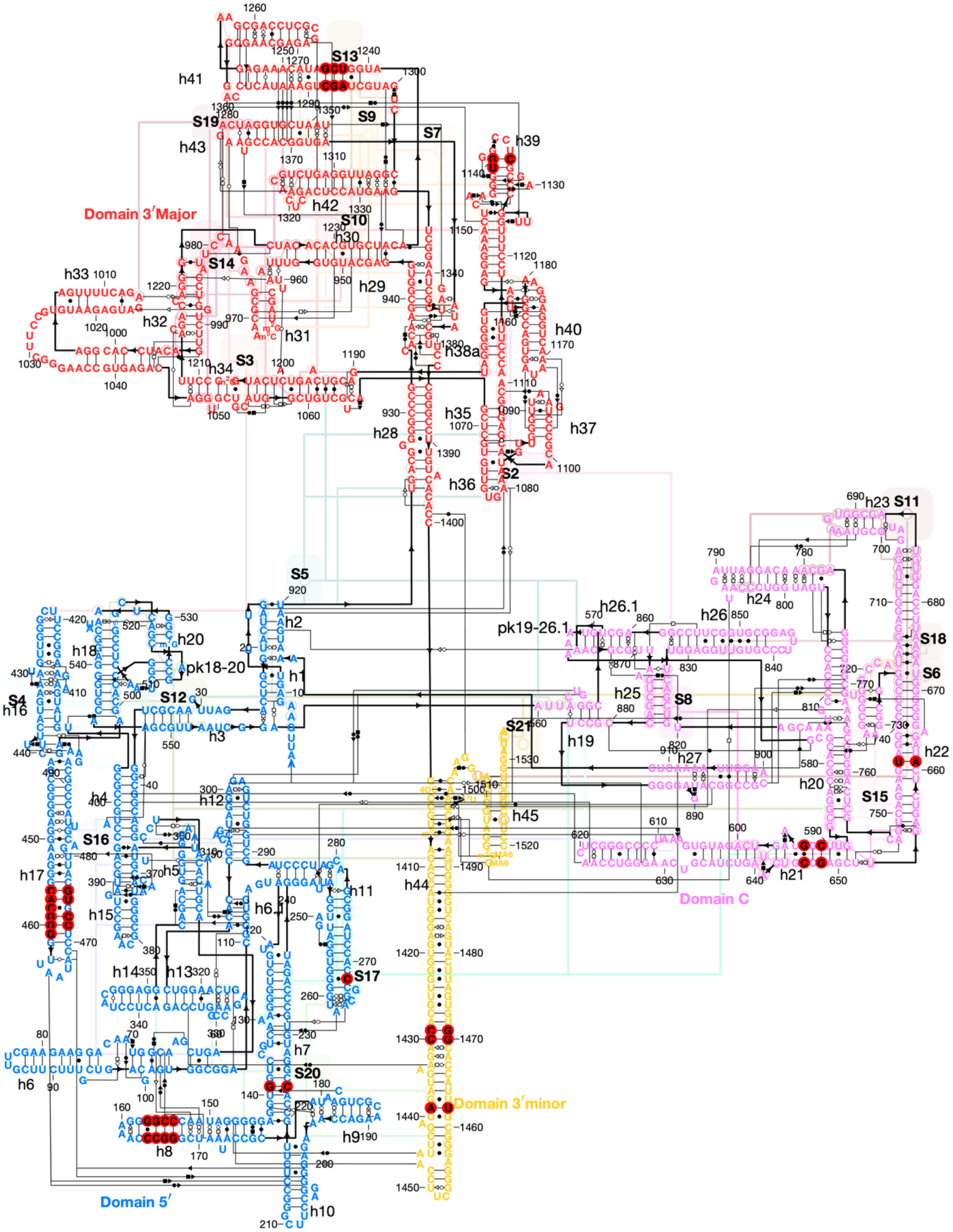

Supplementary Figure 47. Eterna participants'-designed ribosomal RNA design R1-13 prepared with RiboDraw[1].

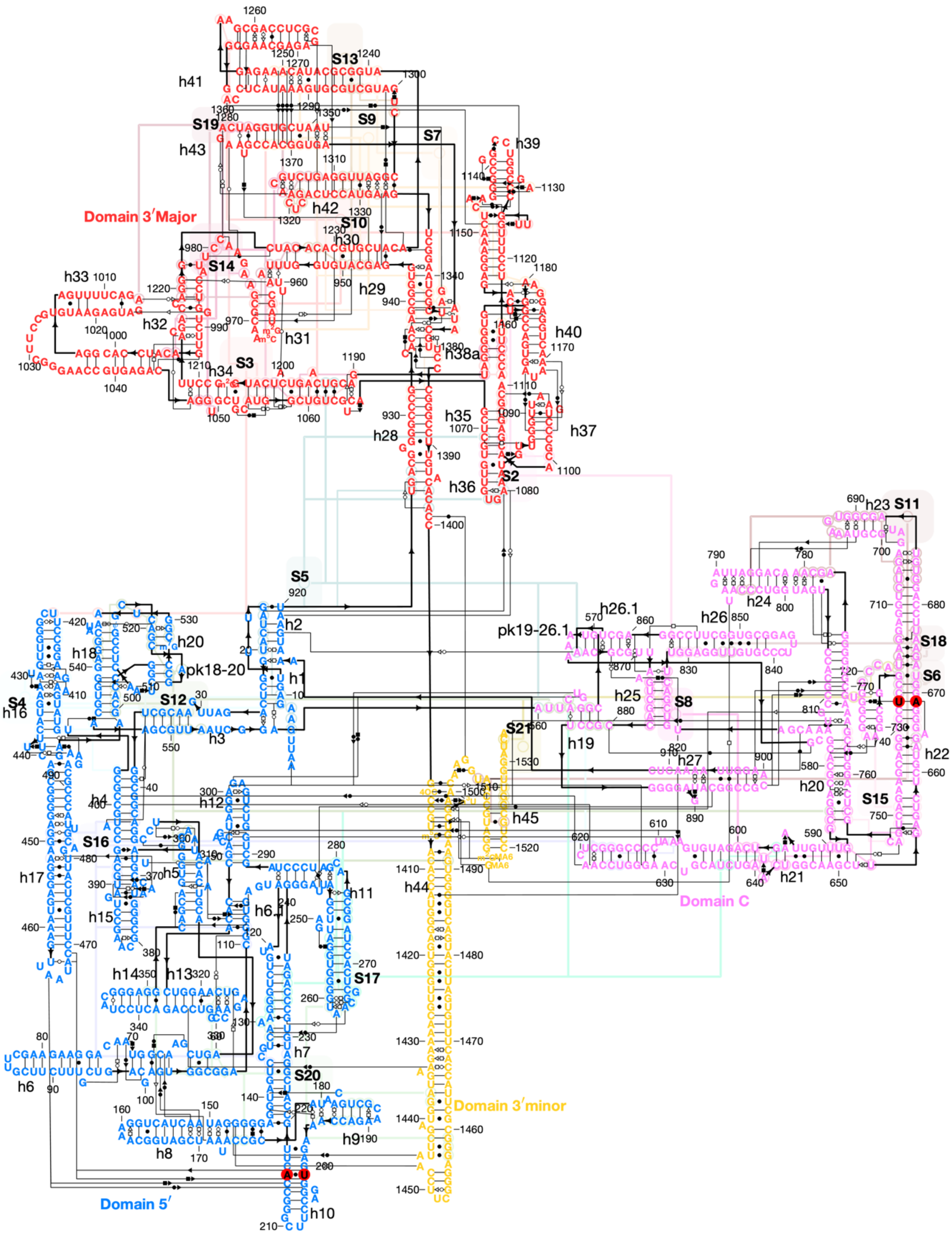

Supplementary Figure 48. Eterna participants'-designed ribosomal RNA design R1-14 prepared with RiboDraw[1].

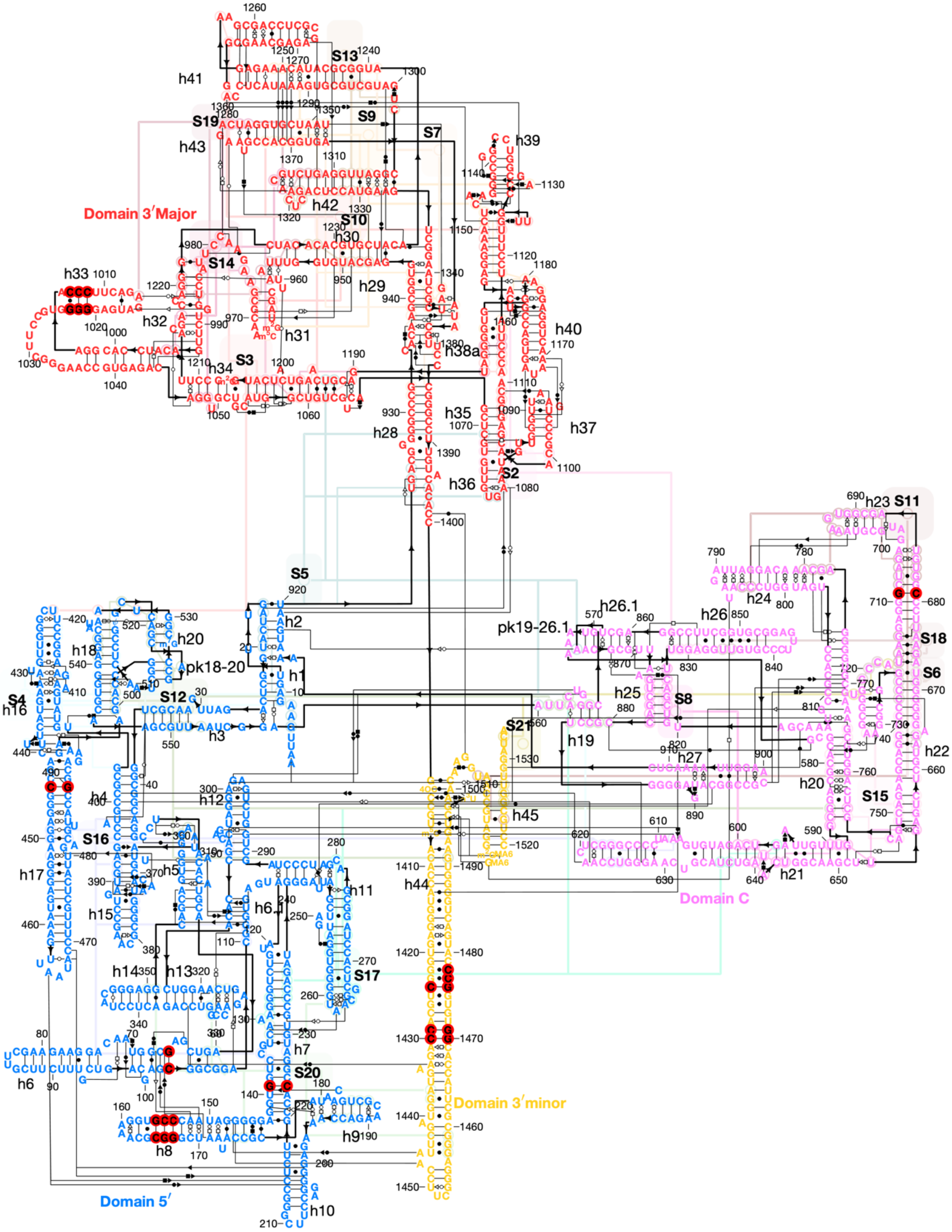

Supplementary Figure 49. Eterna participants'-designed ribosomal RNA design R1-15 prepared with RiboDraw[1].

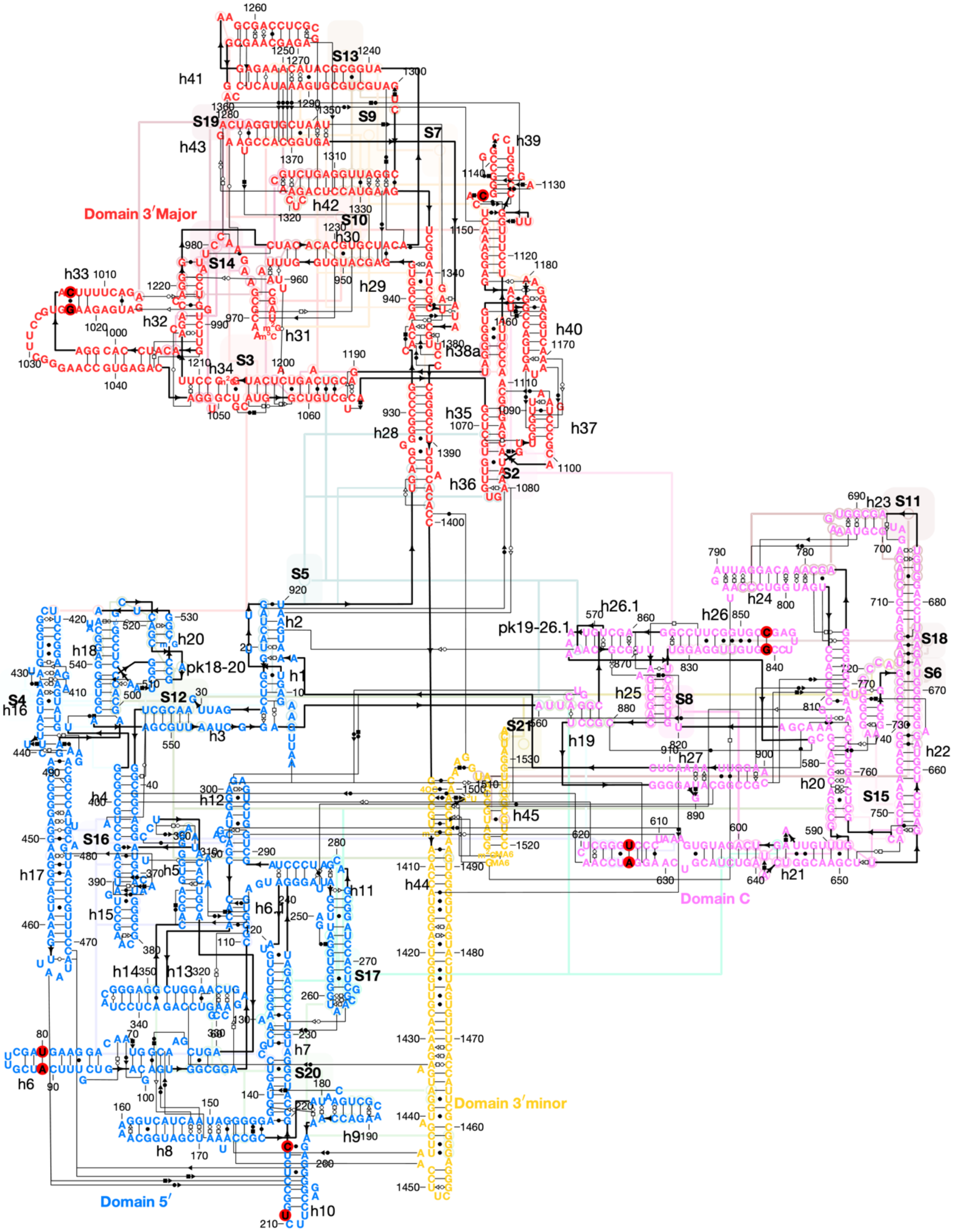

Supplementary Figure 50. Eterna participants'-designed ribosomal RNA design R1-16 prepared with RiboDraw[1].

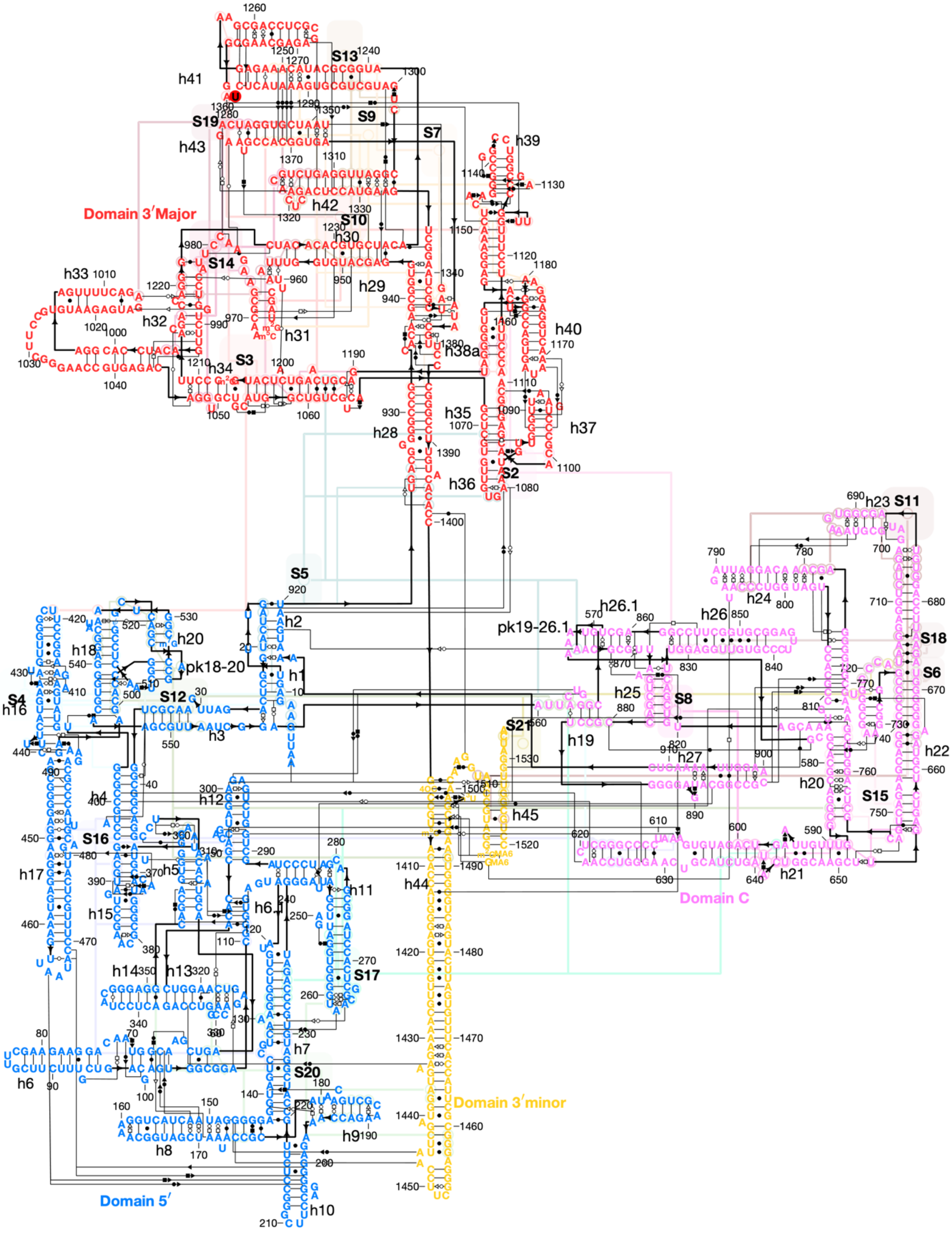

Supplementary Figure 51. Eterna participants'-designed ribosomal RNA design R1-17 prepared with RiboDraw[1].

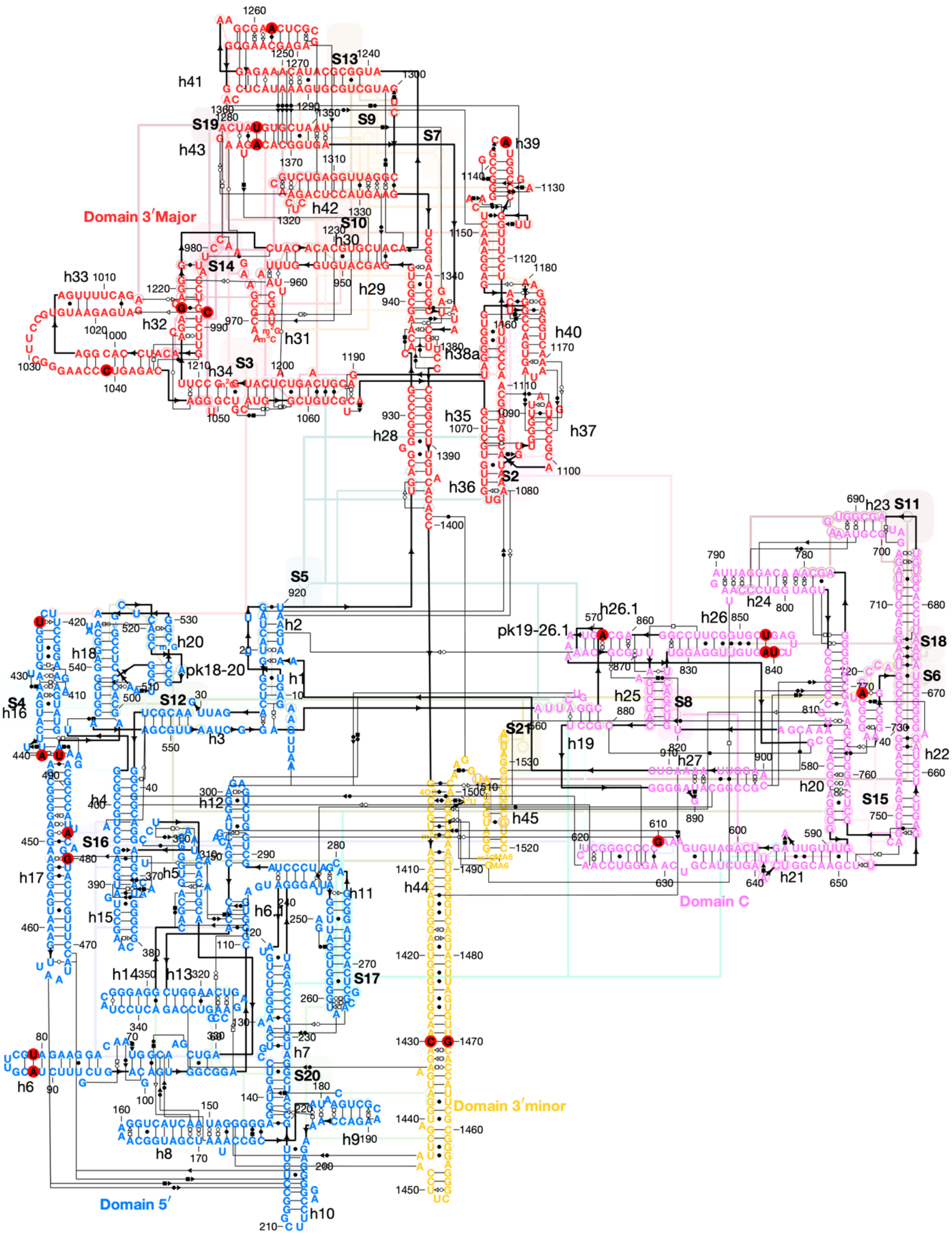

Supplementary Figure 52. Eterna participants'-designed ribosomal RNA design R1-18 prepared with RiboDraw[1].

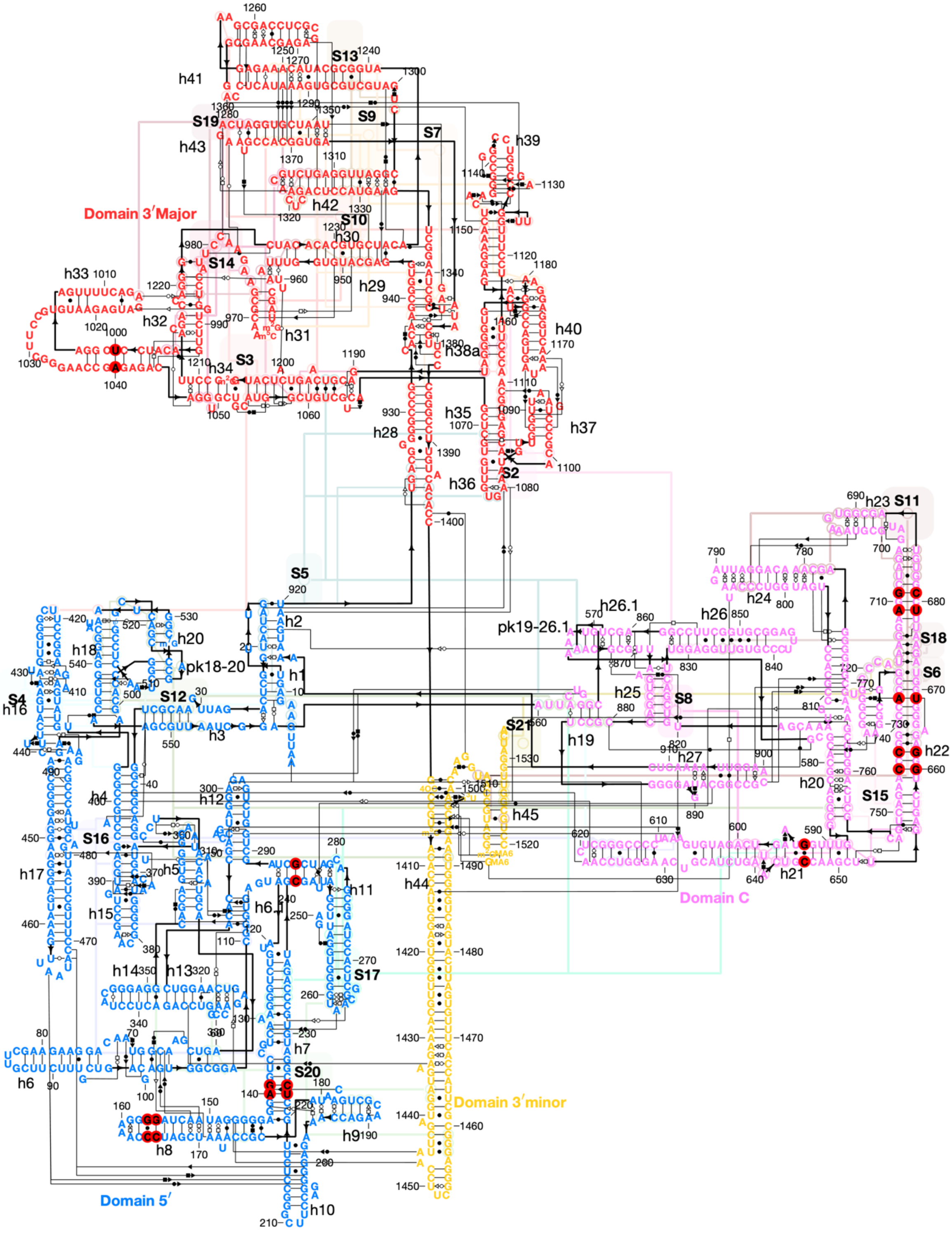

Supplementary Figure 53. Eterna participants'-designed ribosomal RNA design R1-19 prepared with RiboDraw[1].

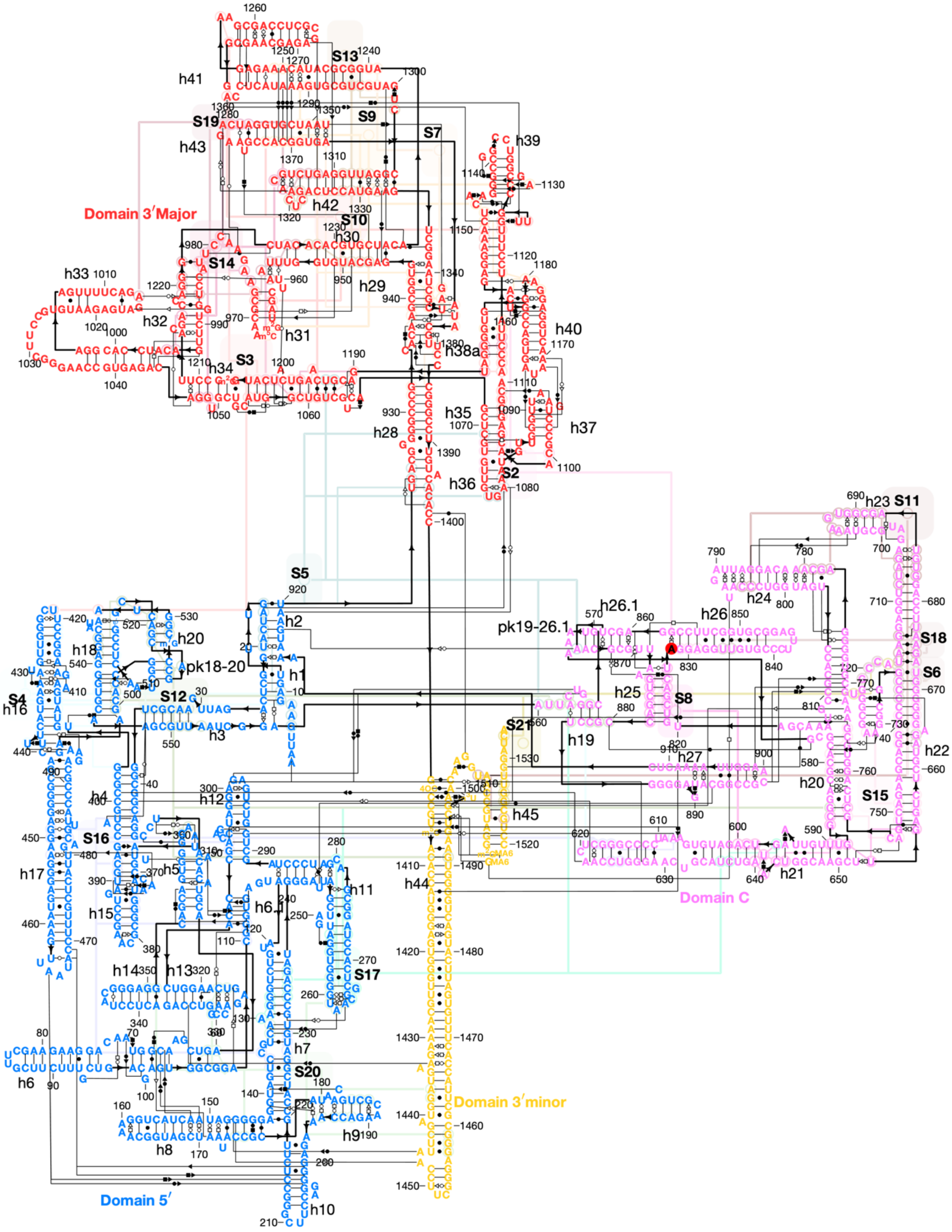

Supplementary Figure 54. Eterna participants'-designed ribosomal RNA design R1-20 prepared with RiboDraw[1].

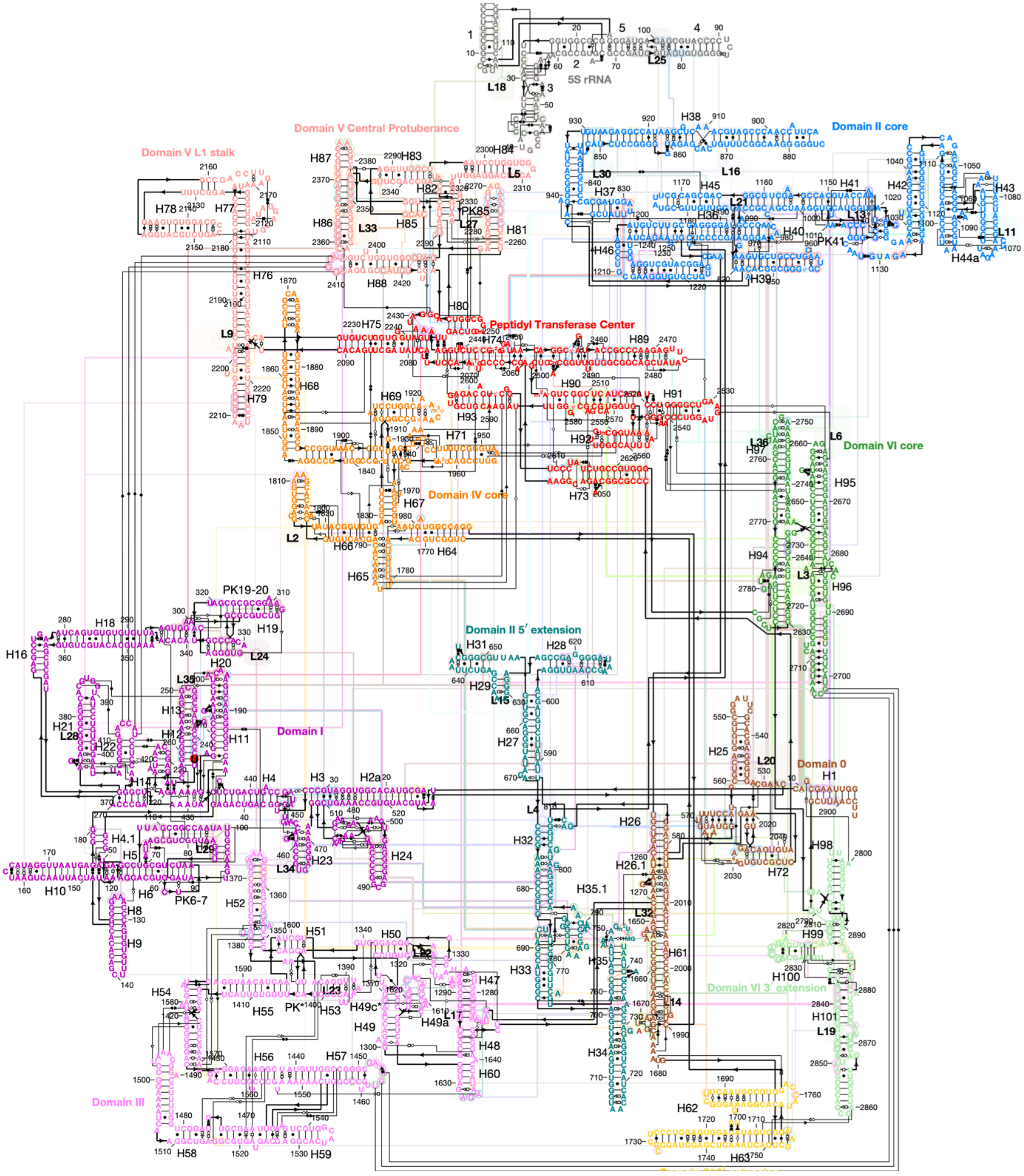

Supplementary Figure 55. Eterna participants'-designed ribosomal RNA design R1-21 prepared with RiboDraw[1].

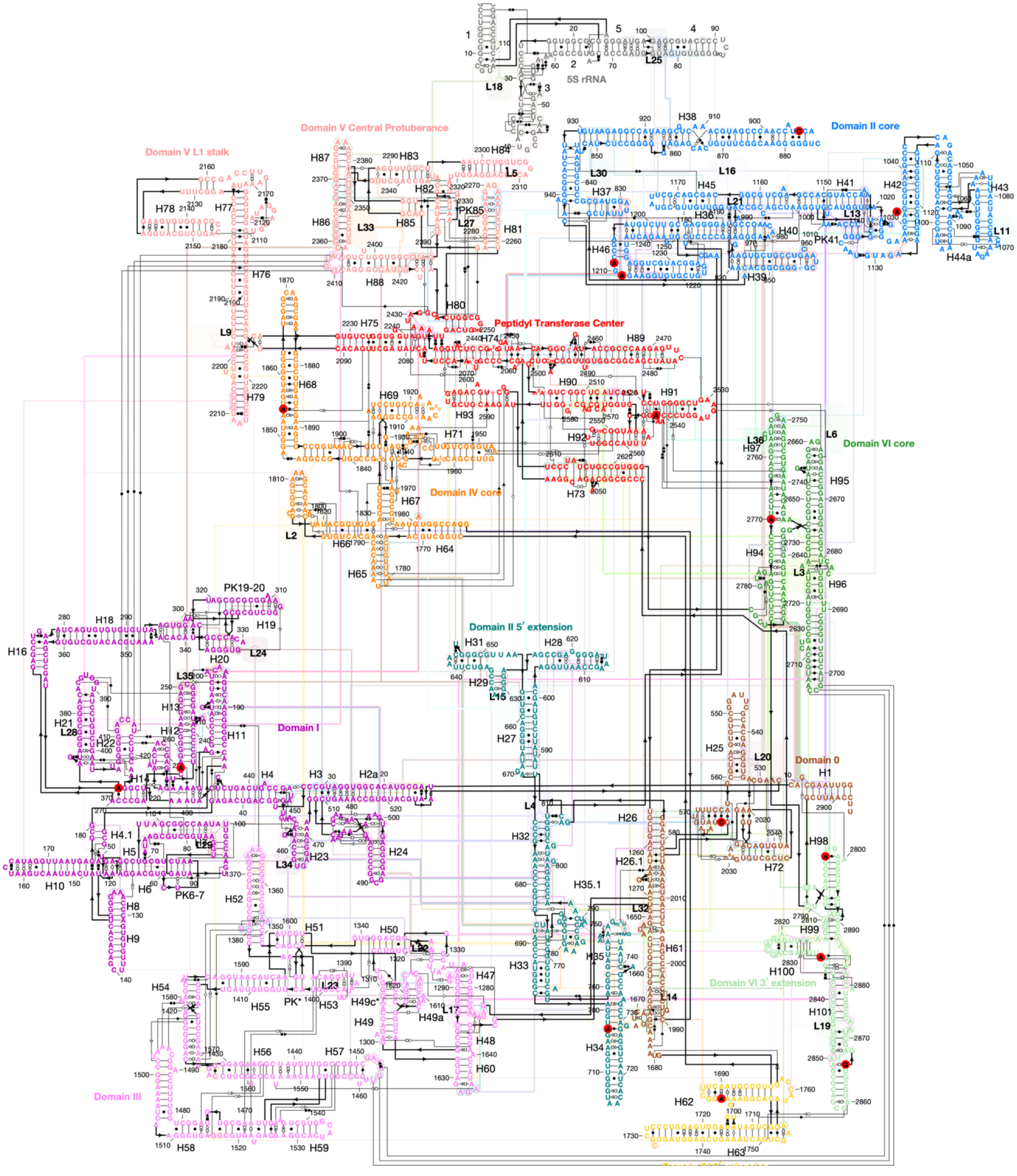

Supplementary Figure 56. Eterna participants'-designed ribosomal RNA design R1-22 prepared with RiboDraw[1].

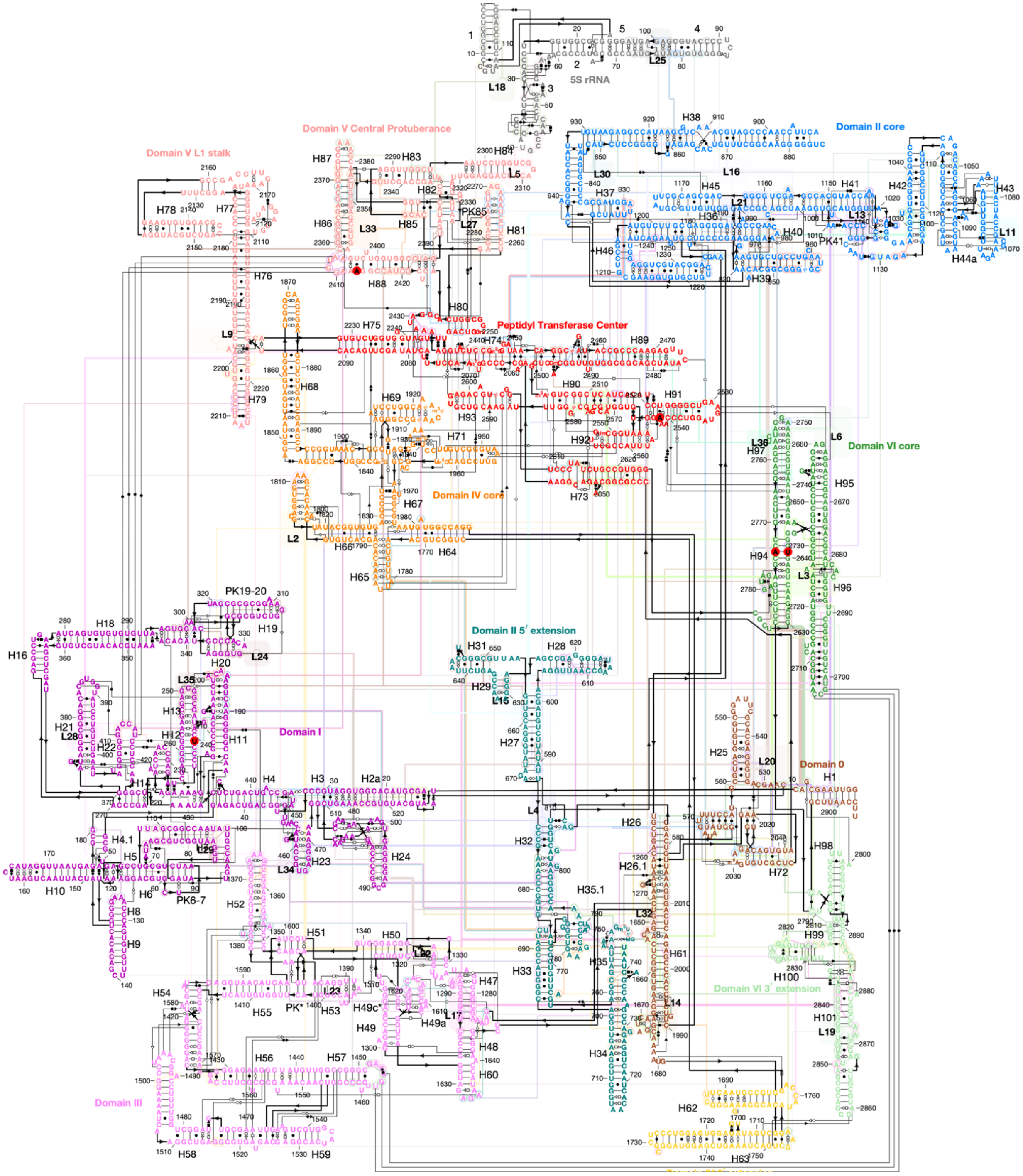

Supplementary Figure 57. Eterna participants'-designed ribosomal RNA design R1-23 prepared with RiboDraw[1].

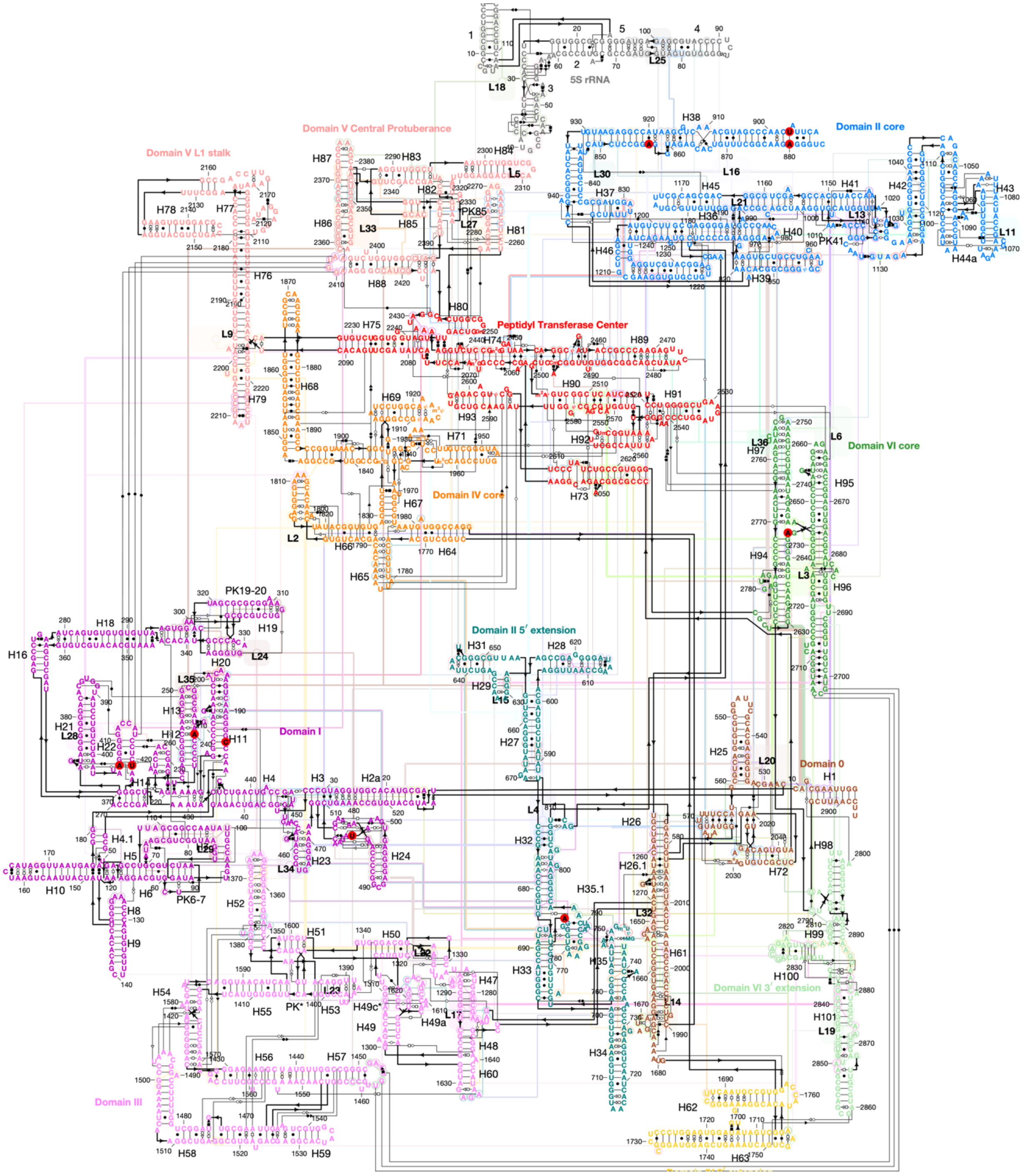

Supplementary Figure 58. Eterna participants'-designed ribosomal RNA design R1-24 prepared with RiboDraw[1].

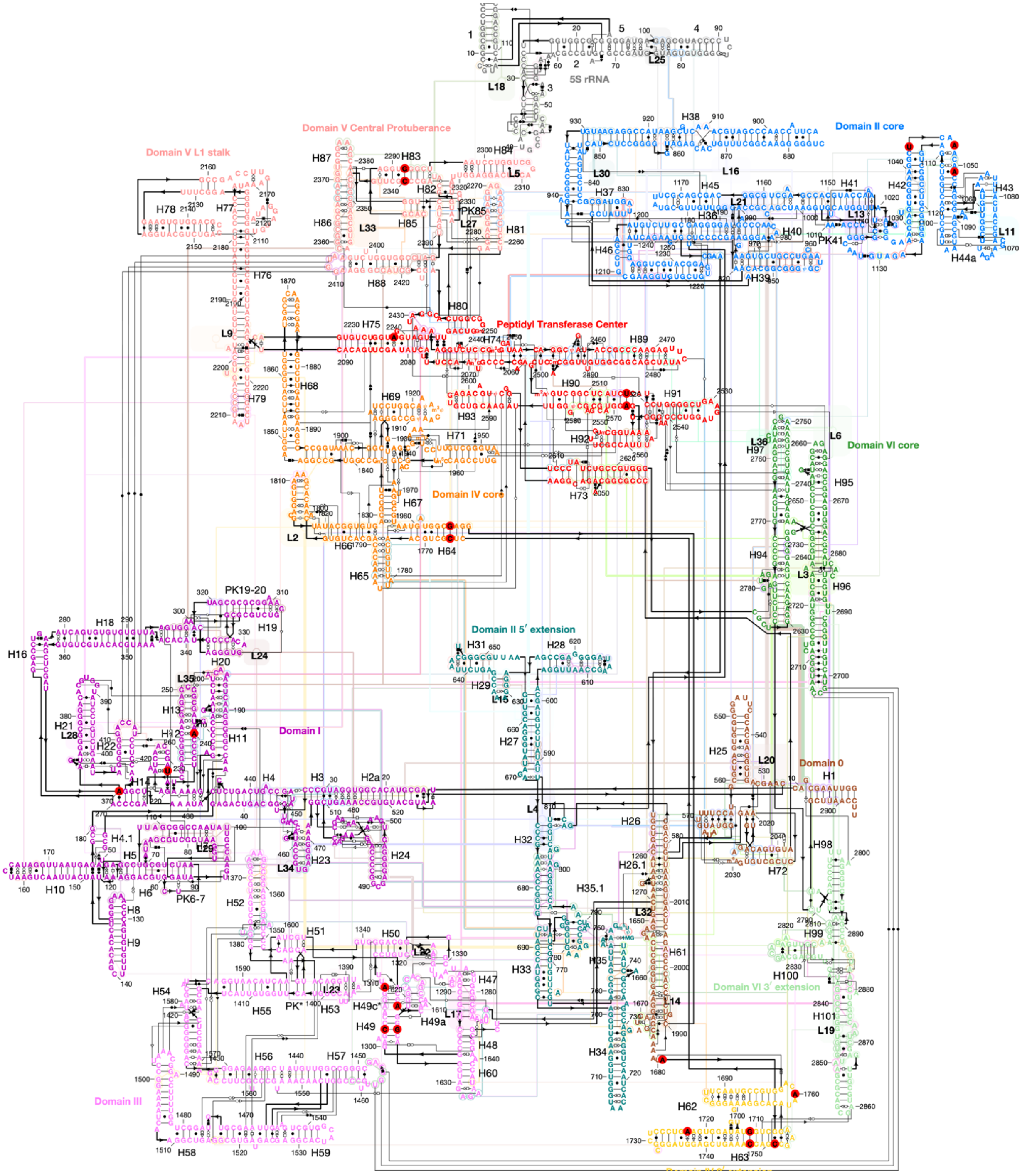

Supplementary Figure 59. Eterna participants'-designed ribosomal RNA design R1-25 prepared with RiboDraw[1].

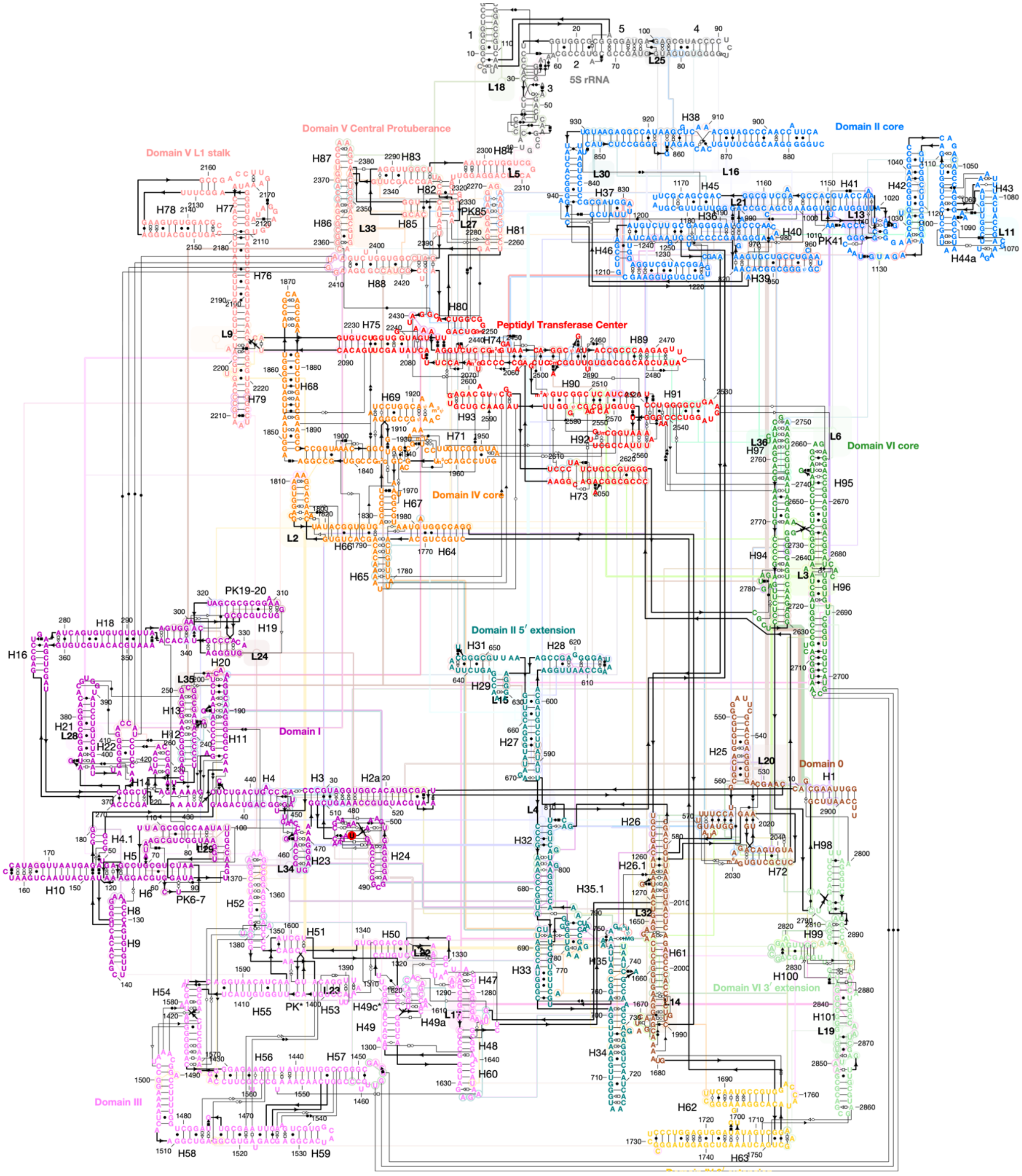

Supplementary Figure 60. Eterna participants'-designed ribosomal RNA design R1-26 prepared with RiboDraw[1].

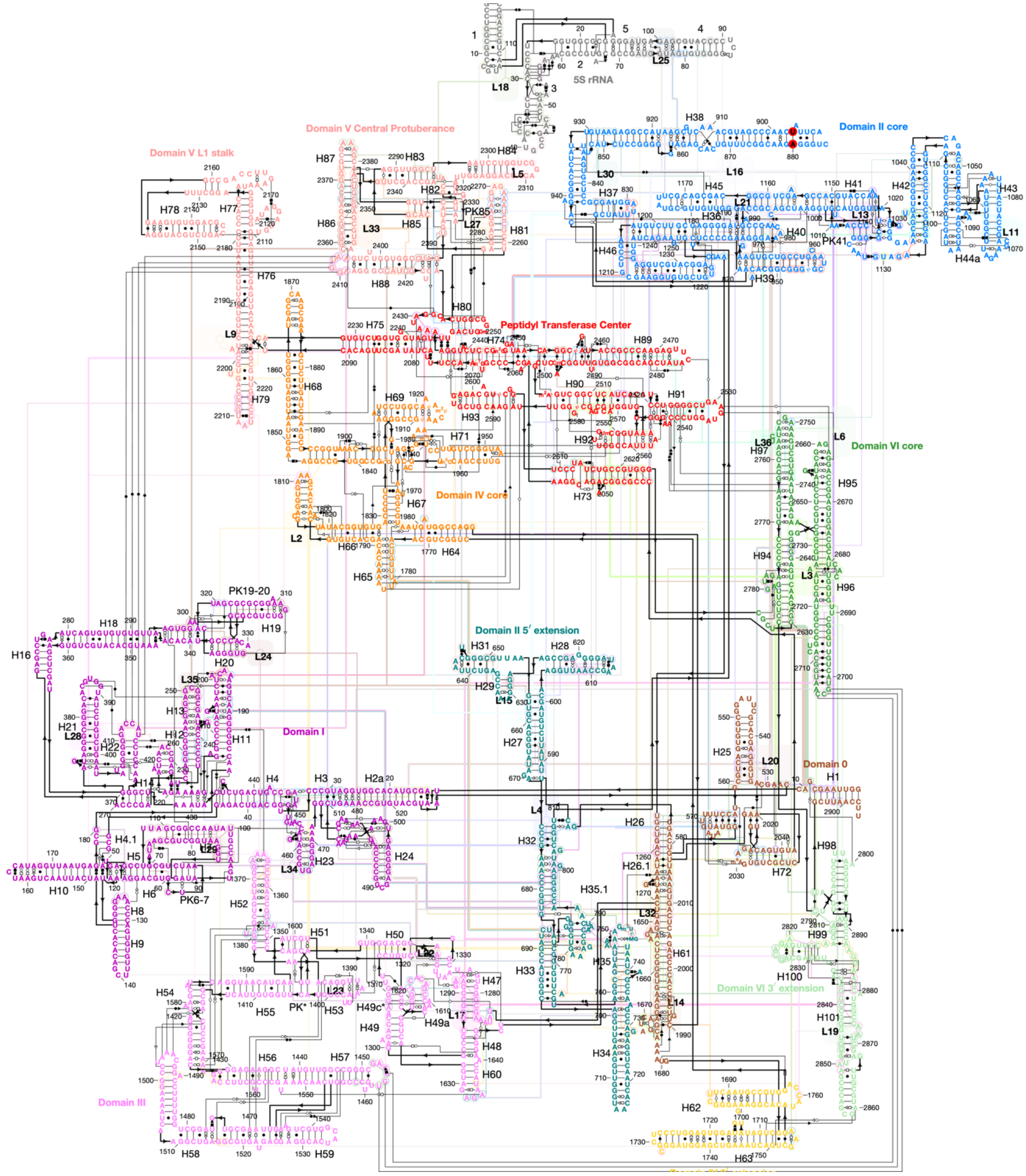

Supplementary Figure 61. Eterna participants'-designed ribosomal RNA design R1-27 prepared with RiboDraw[1].

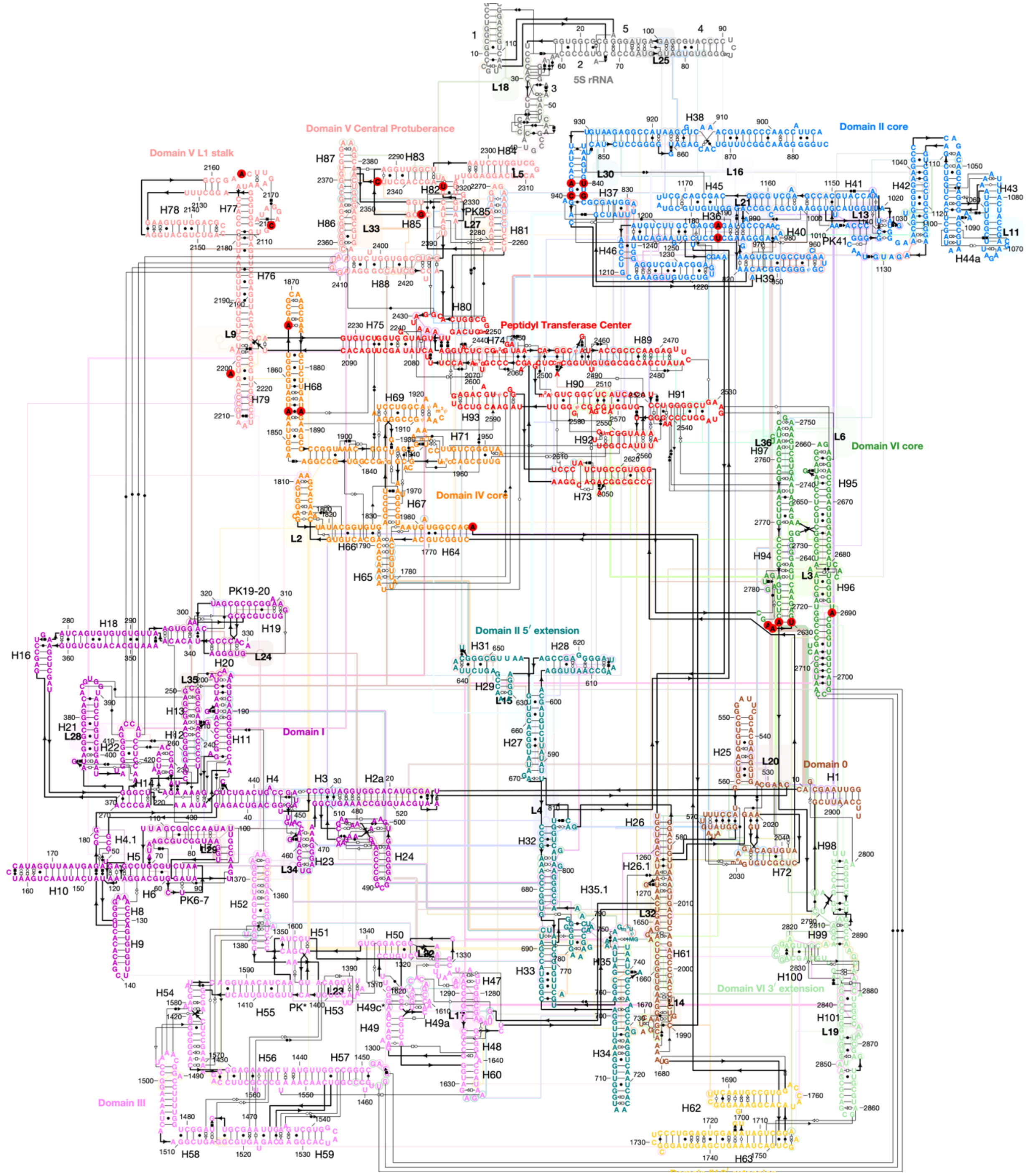

Supplementary Figure 62. Eterna participants'-designed ribosomal RNA design R1-28 prepared with RiboDraw[1].

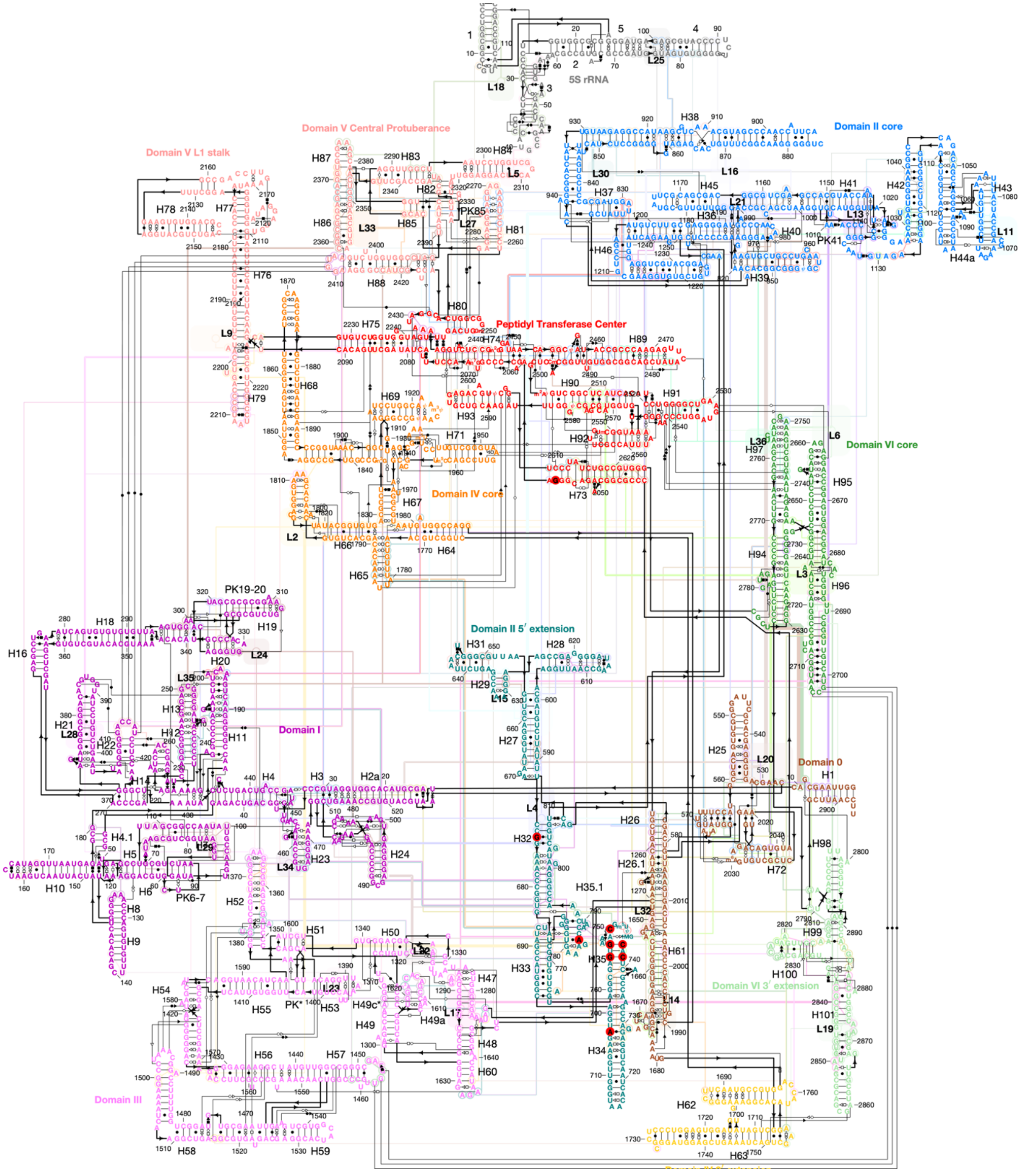

Supplementary Figure 63. Eterna participants'-designed ribosomal RNA design R1-29 prepared with RiboDraw[1].

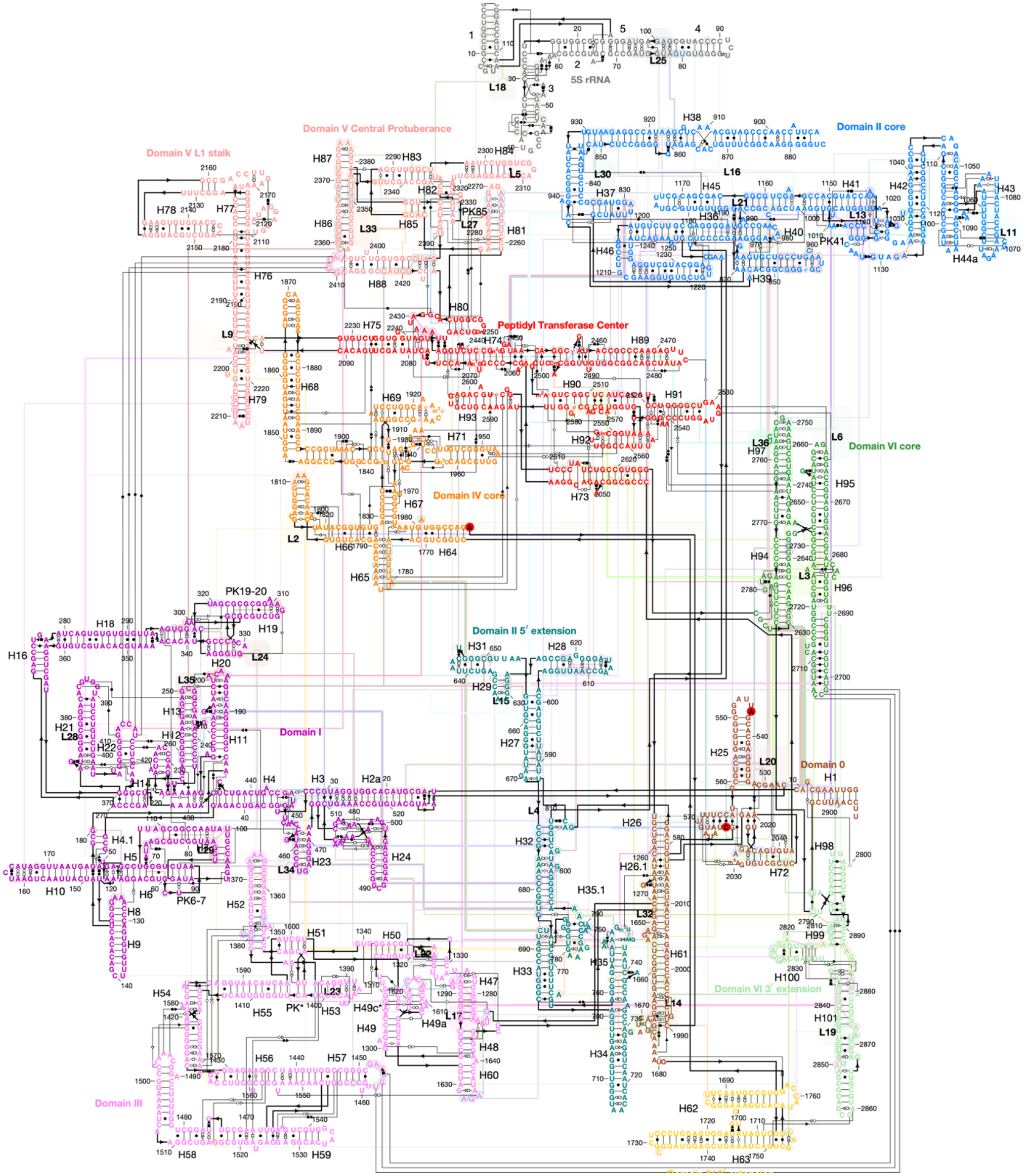

Supplementary Figure 64. Eterna participants'-designed ribosomal RNA design R1-30 prepared with RiboDraw[1].

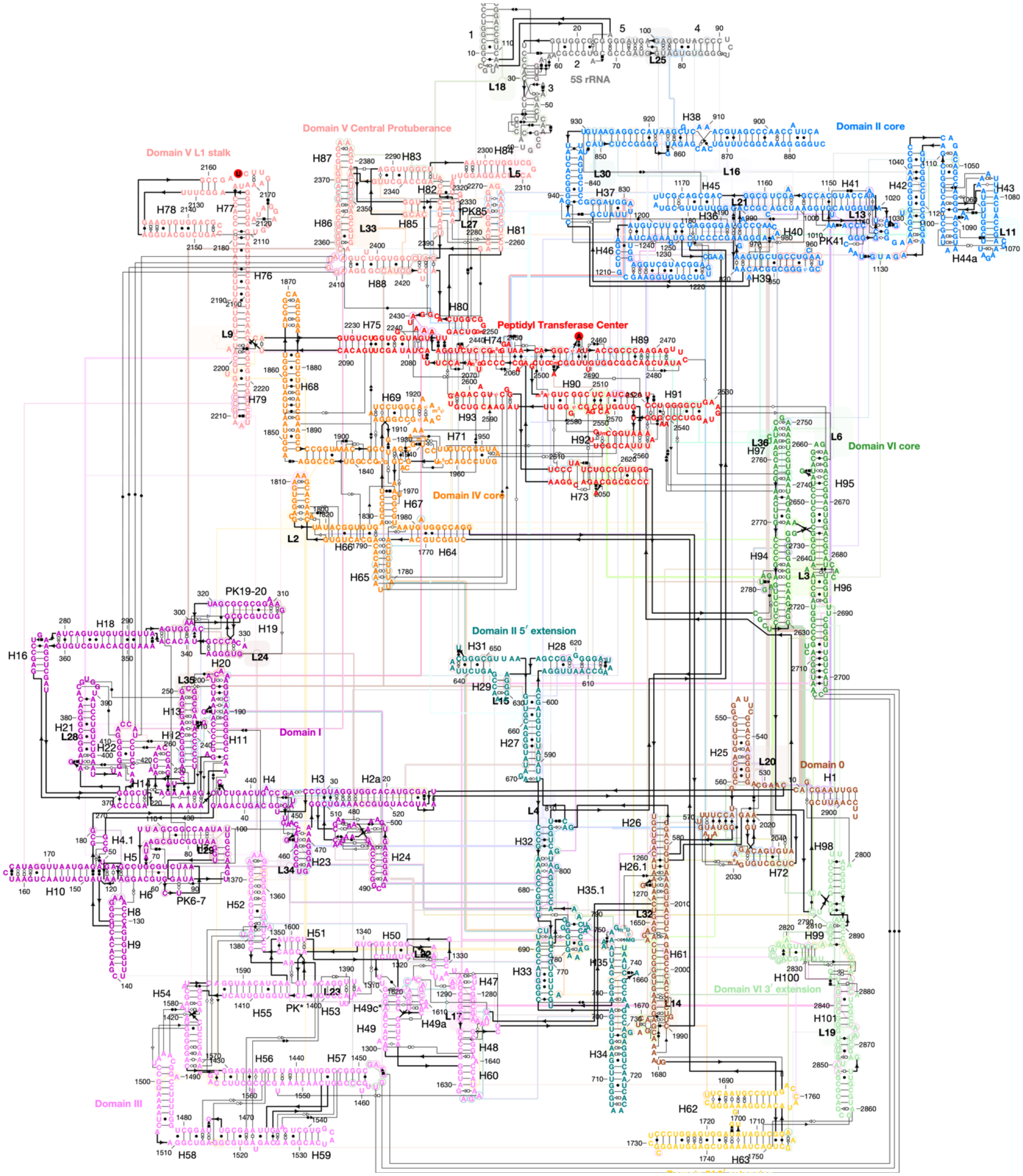

Supplementary Figure 65. Eterna participants'-designed ribosomal RNA design R1-31 prepared with RiboDraw[1].

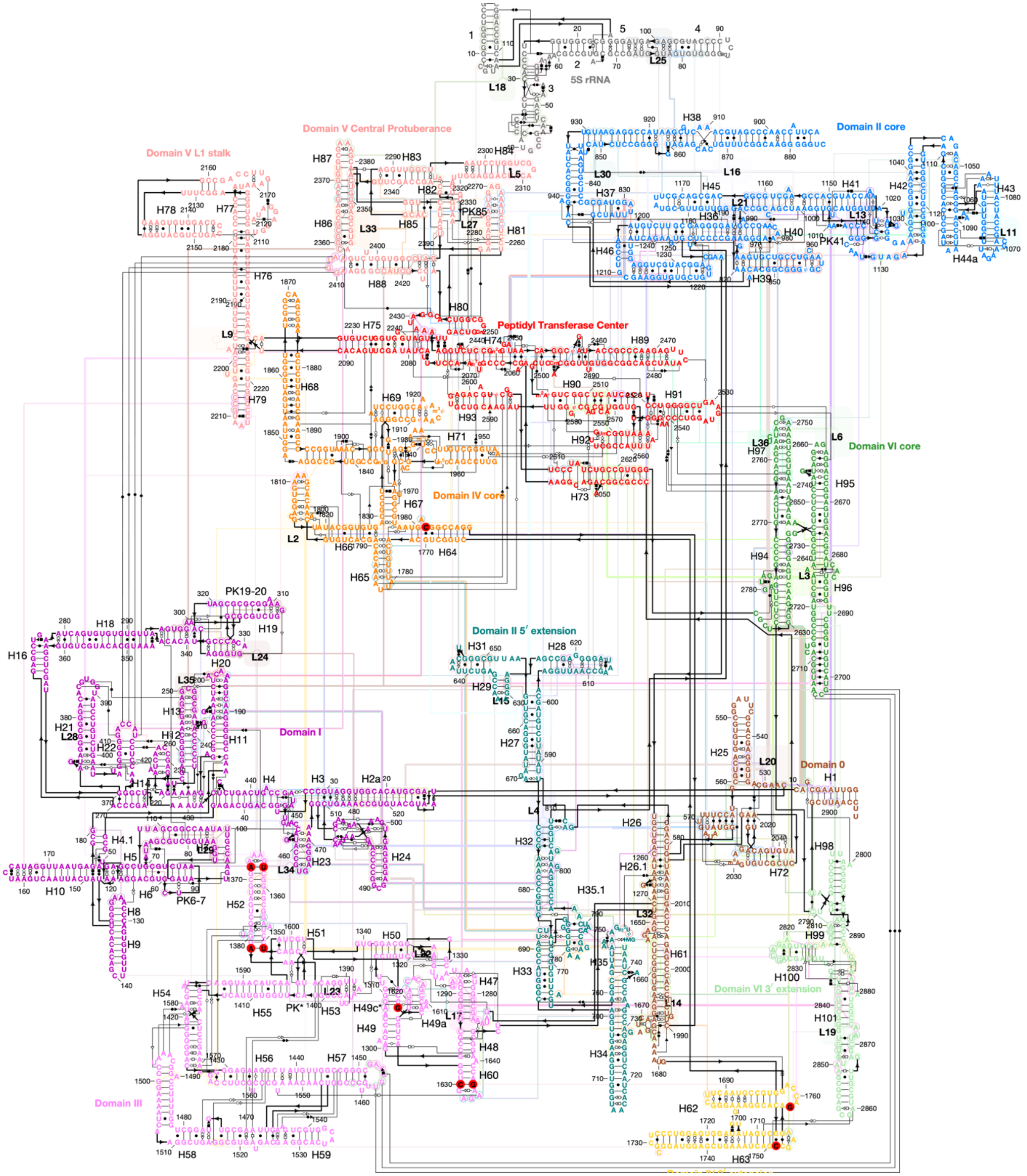

Supplementary Figure 66. Eterna participants'-designed ribosomal RNA design R1-32 prepared with RiboDraw[1].

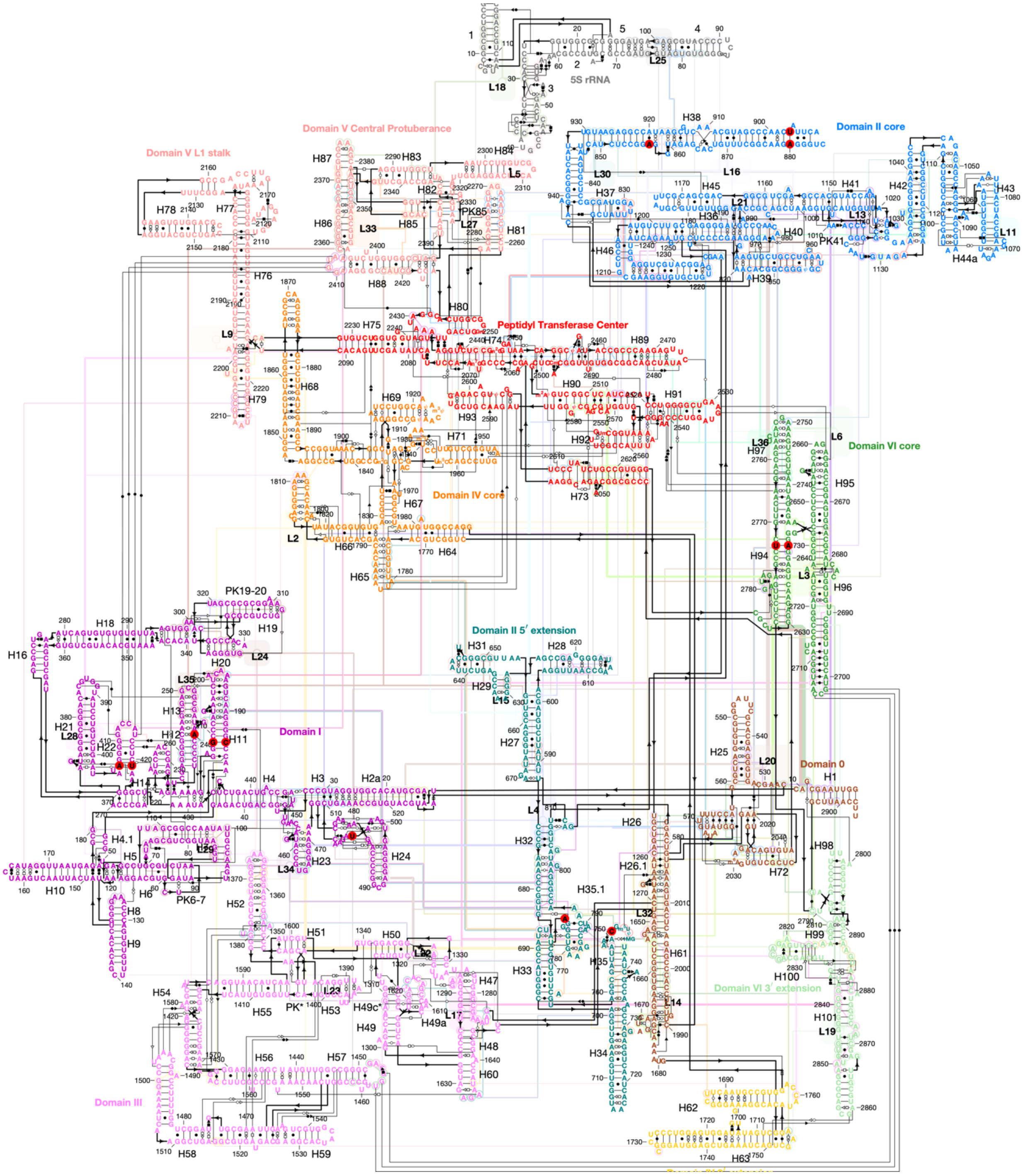

Supplementary Figure 67. Eterna participants'-designed ribosomal RNA design R1-33 prepared with RiboDraw[1].

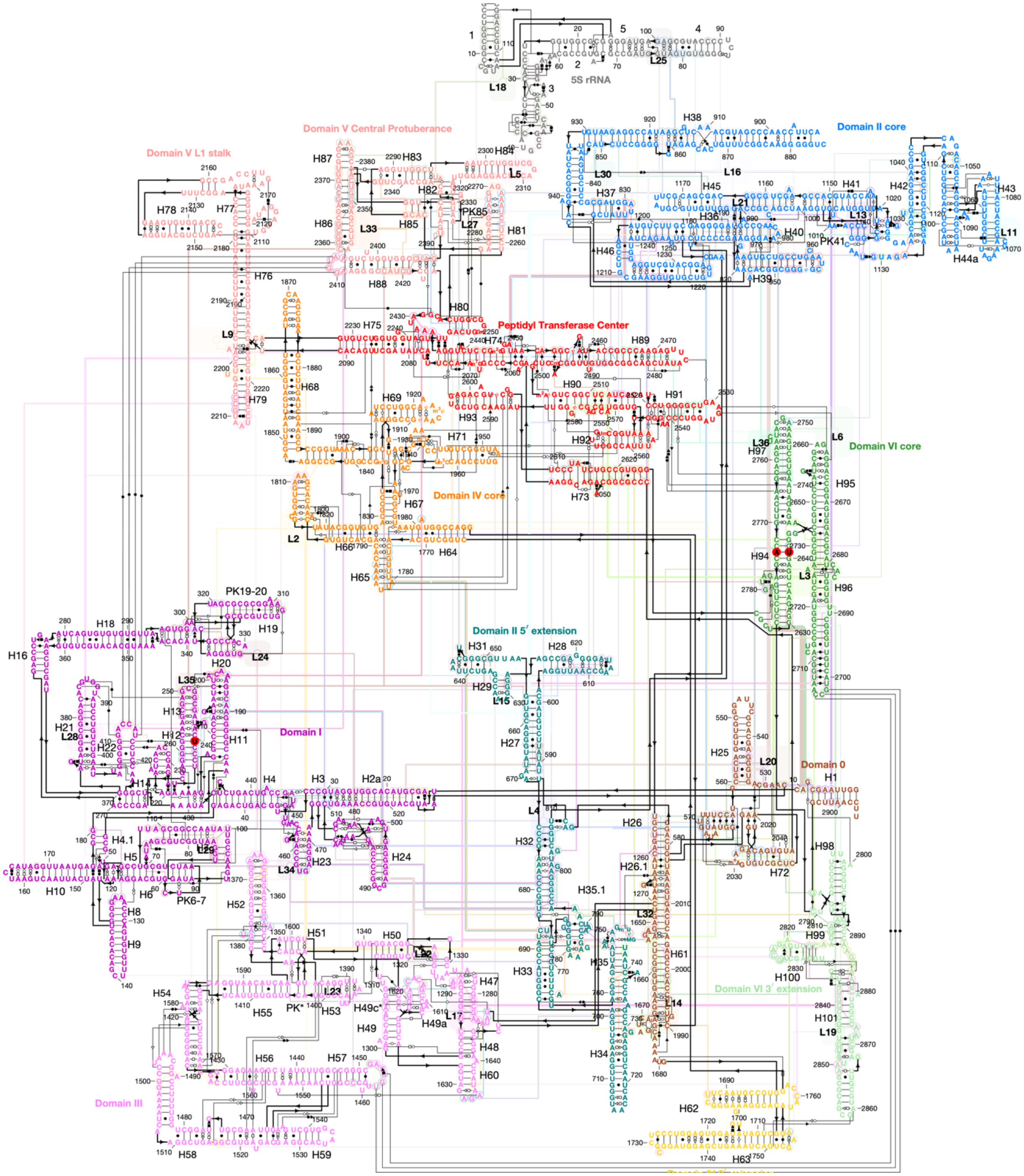

Supplementary Figure 68. Eterna participants'-designed ribosomal RNA design R1-34 prepared with RiboDraw[1].

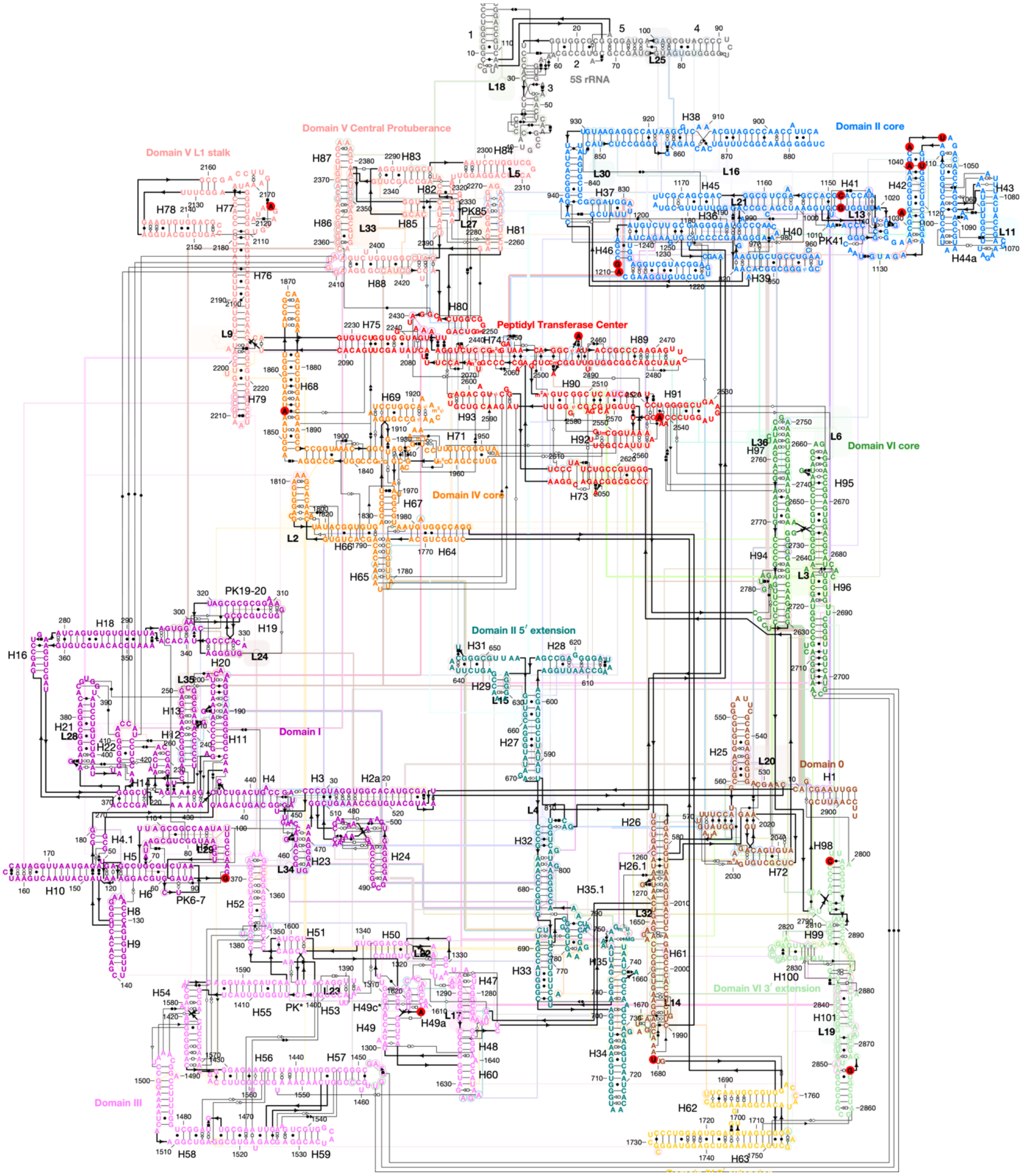

Supplementary Figure 69. Eterna participants'-designed ribosomal RNA design R1-35 prepared with RiboDraw[1].

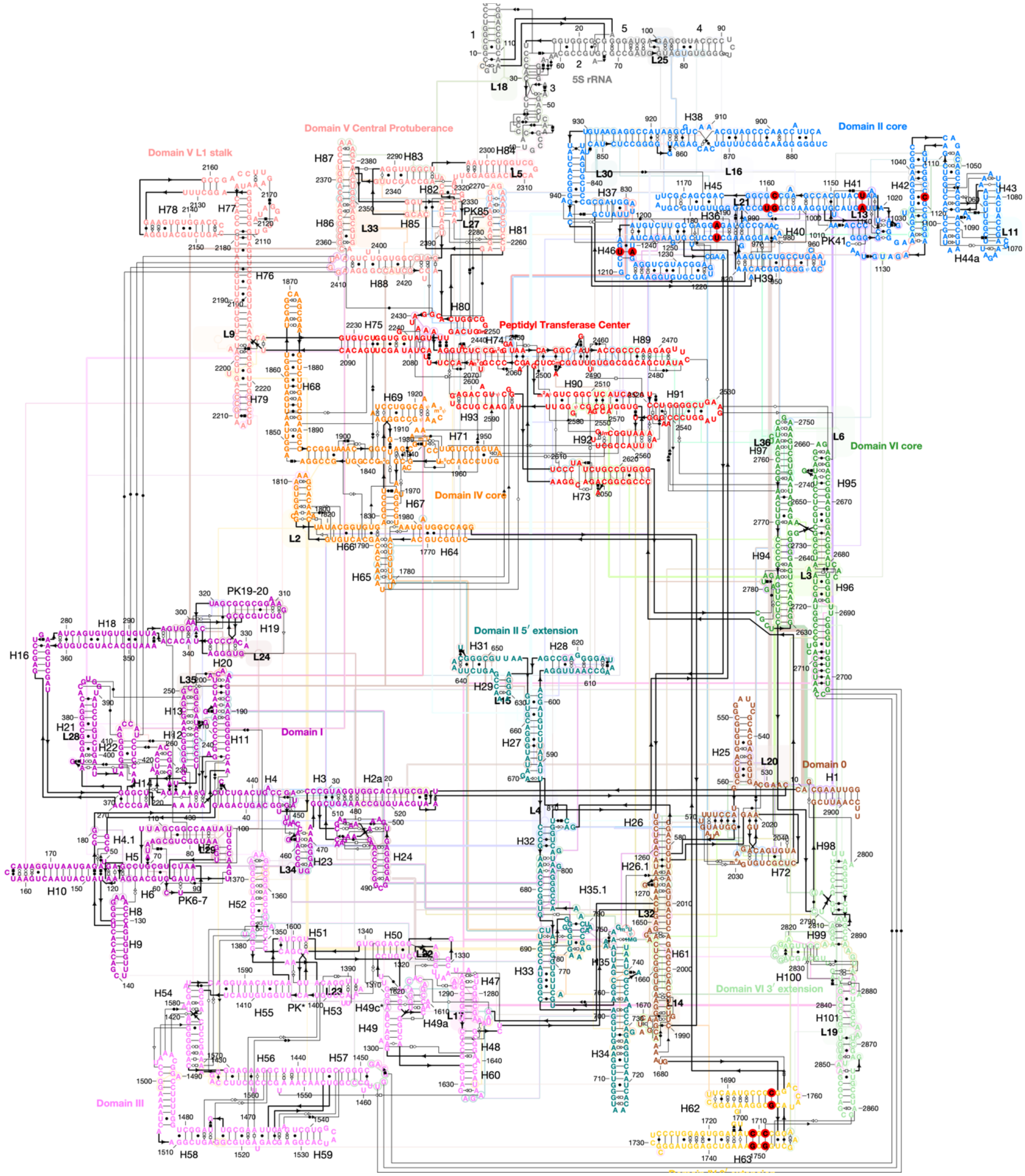

Supplementary Figure 70. Eterna participants'-designed ribosomal RNA design R1-36 prepared with RiboDraw[1].

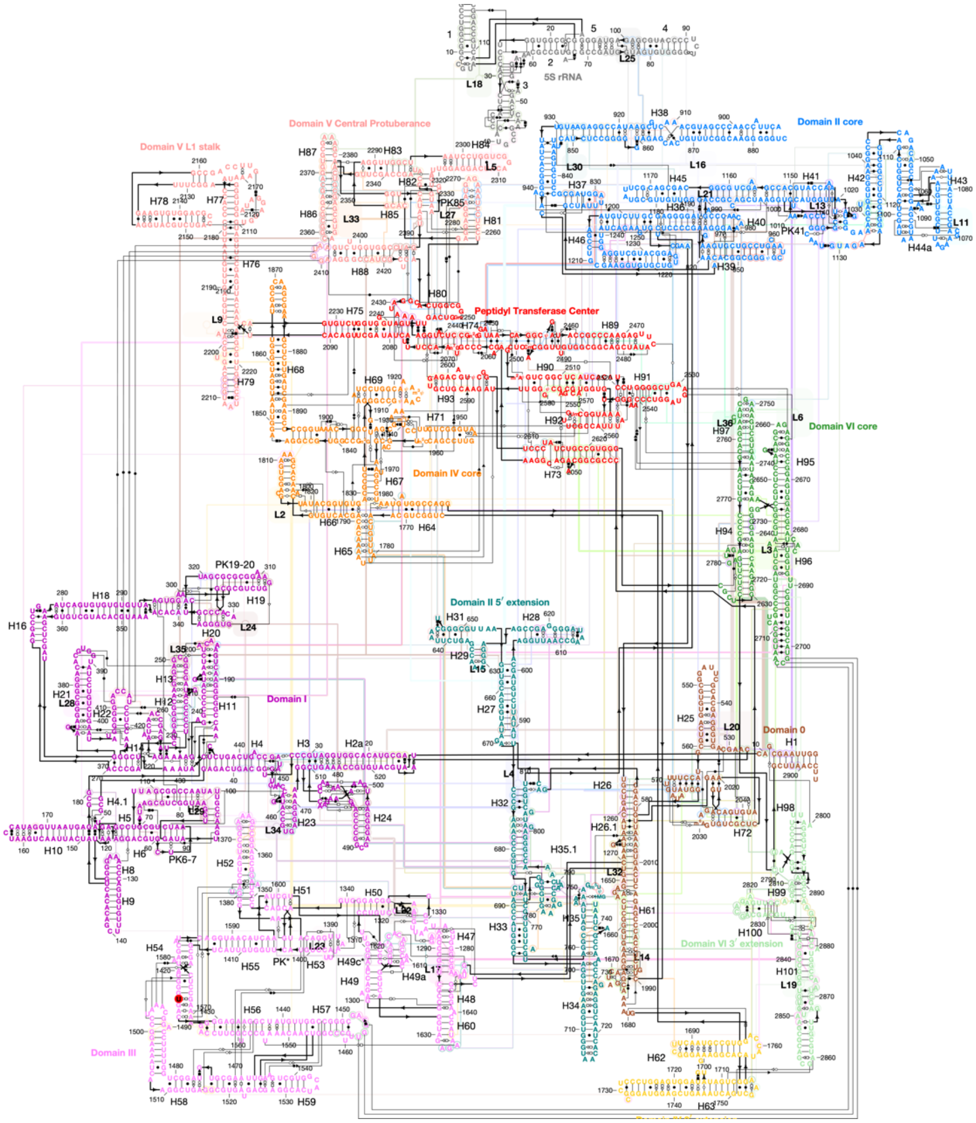

Supplementary Figure 71. Eterna participants'-designed ribosomal RNA design R1-37 prepared with RiboDraw[1].

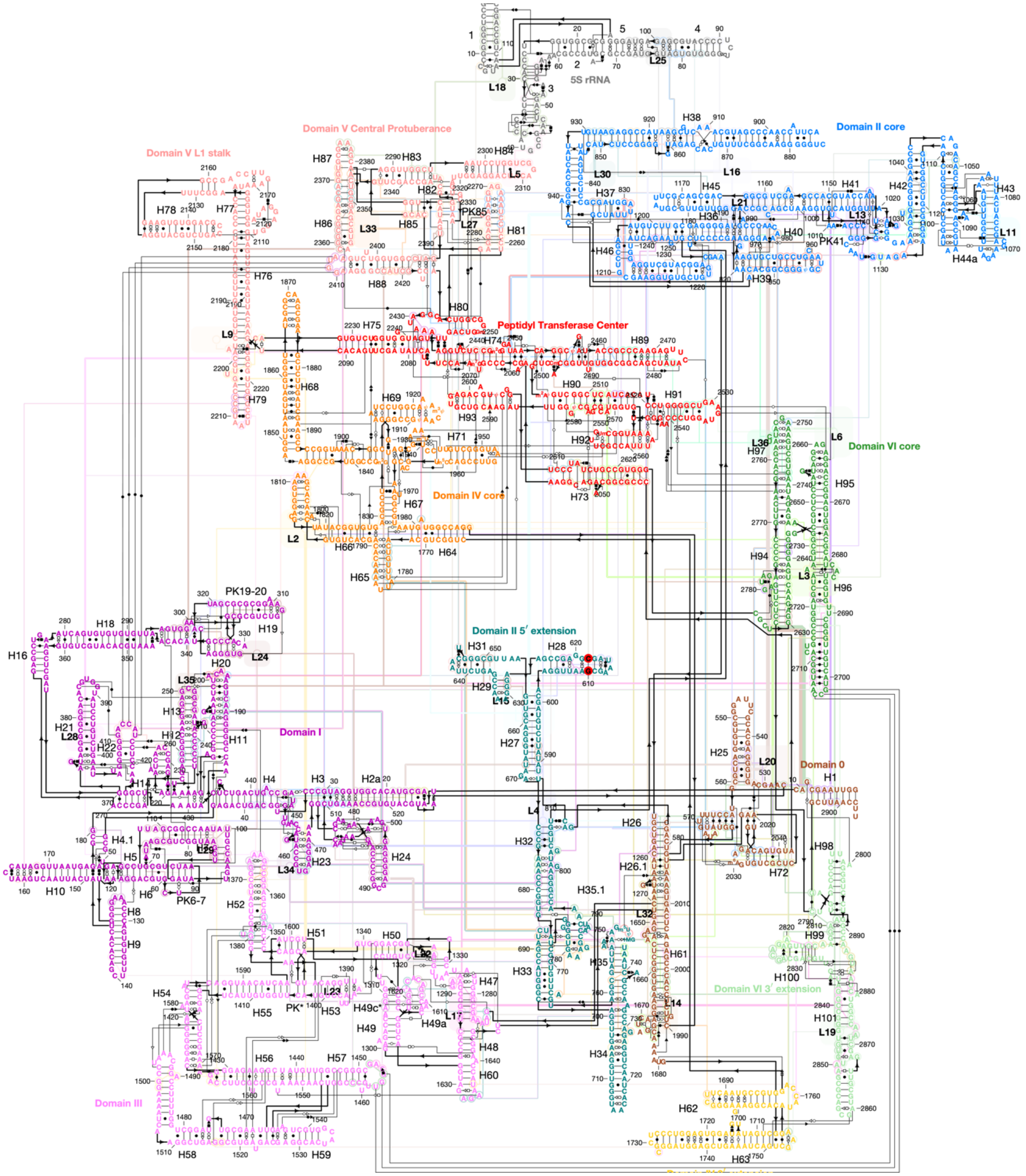

Supplementary Figure 72. Eterna participants'-designed ribosomal RNA design R1-38 prepared with RiboDraw[1].

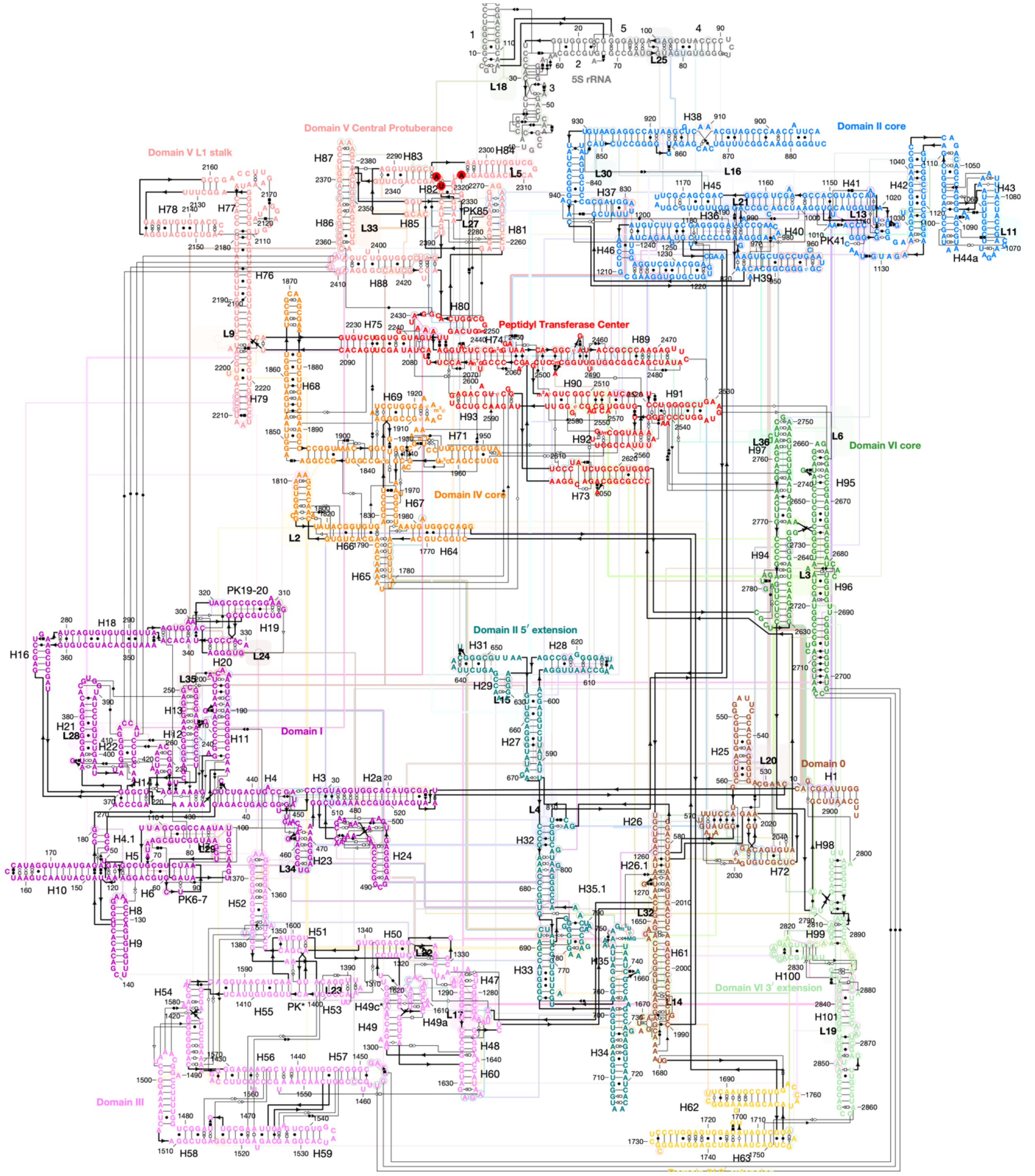

Supplementary Figure 73. Eterna participants'-designed ribosomal RNA design R1-39 prepared with RiboDraw[1].

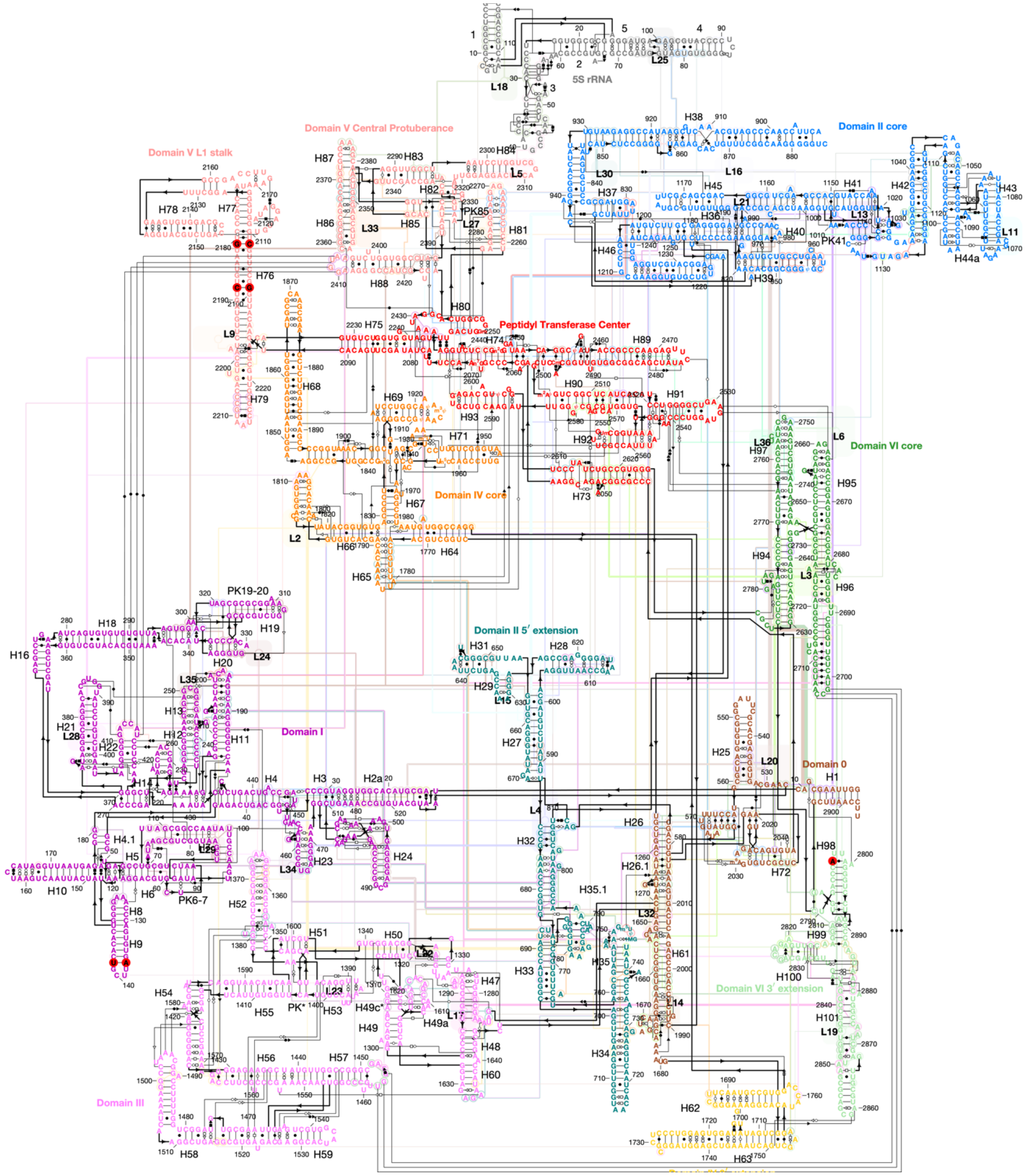

Supplementary Figure 74. Eterna participants'-designed ribosomal RNA design R1-40 prepared with RiboDraw[1].

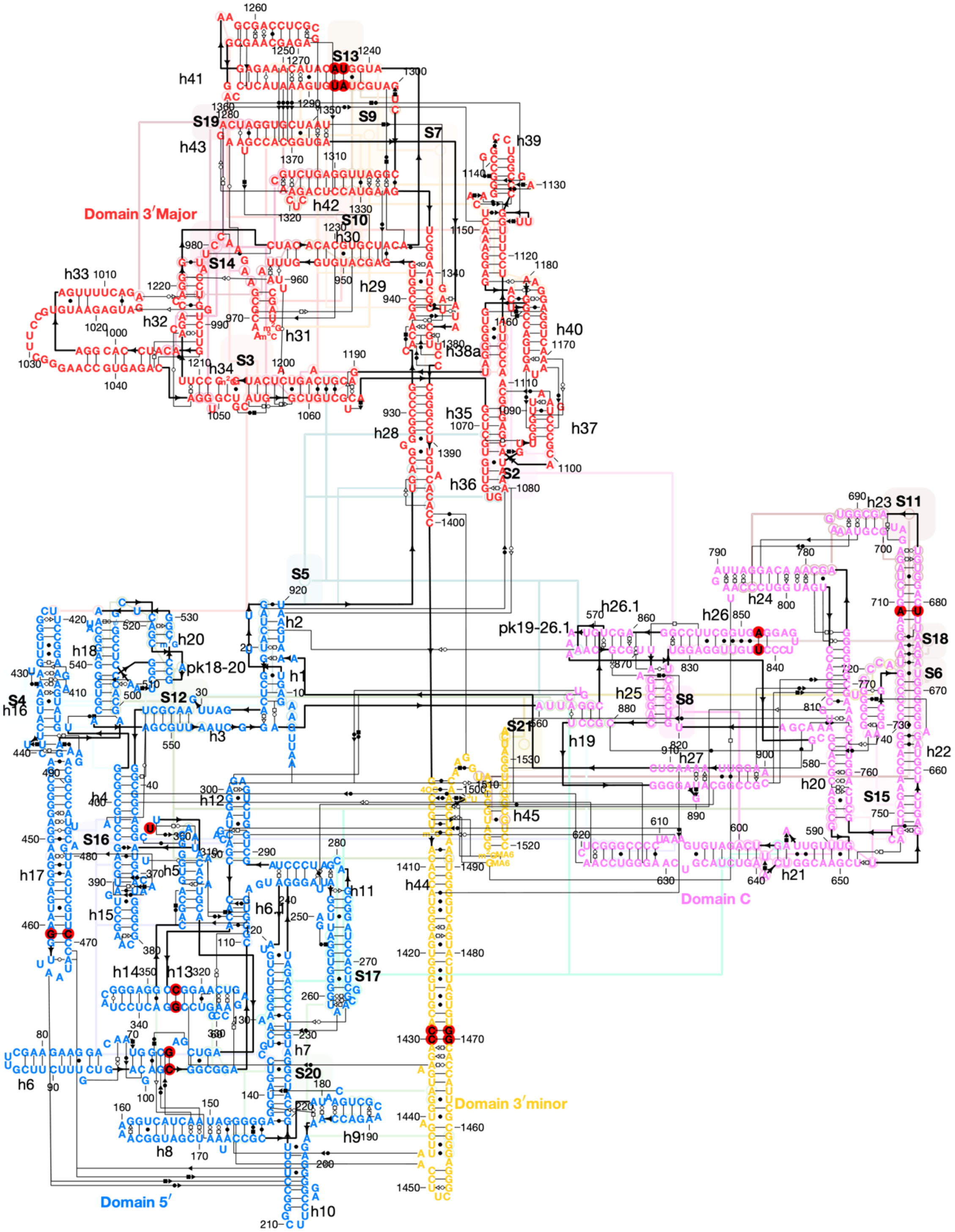

Supplementary Figure 75. Eterna participants'-designed ribosomal RNA design R2-01 prepared with RiboDraw[1].

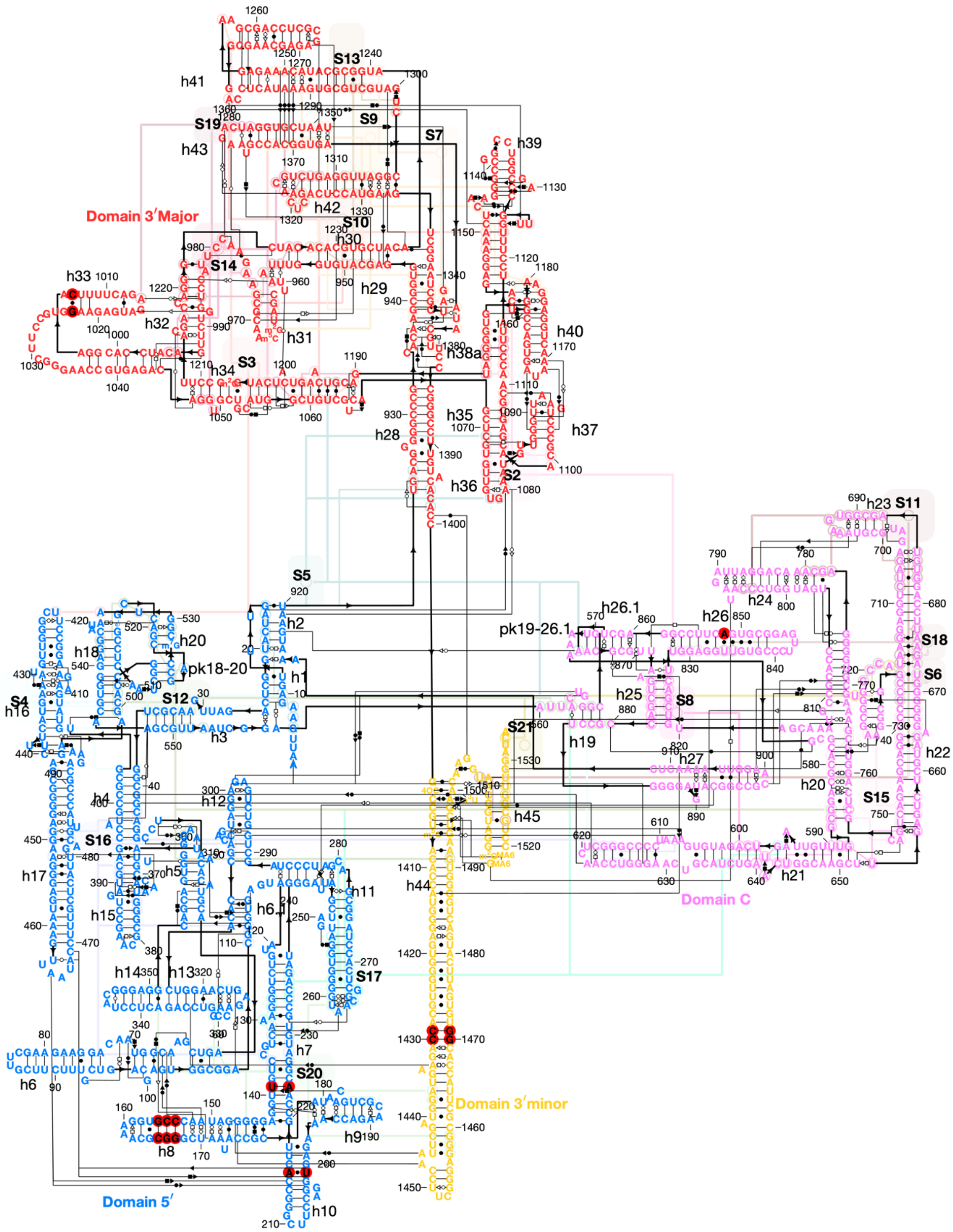

Supplementary Figure 76. Eterna participants'-designed ribosomal RNA design R2-02 prepared with RiboDraw[1].

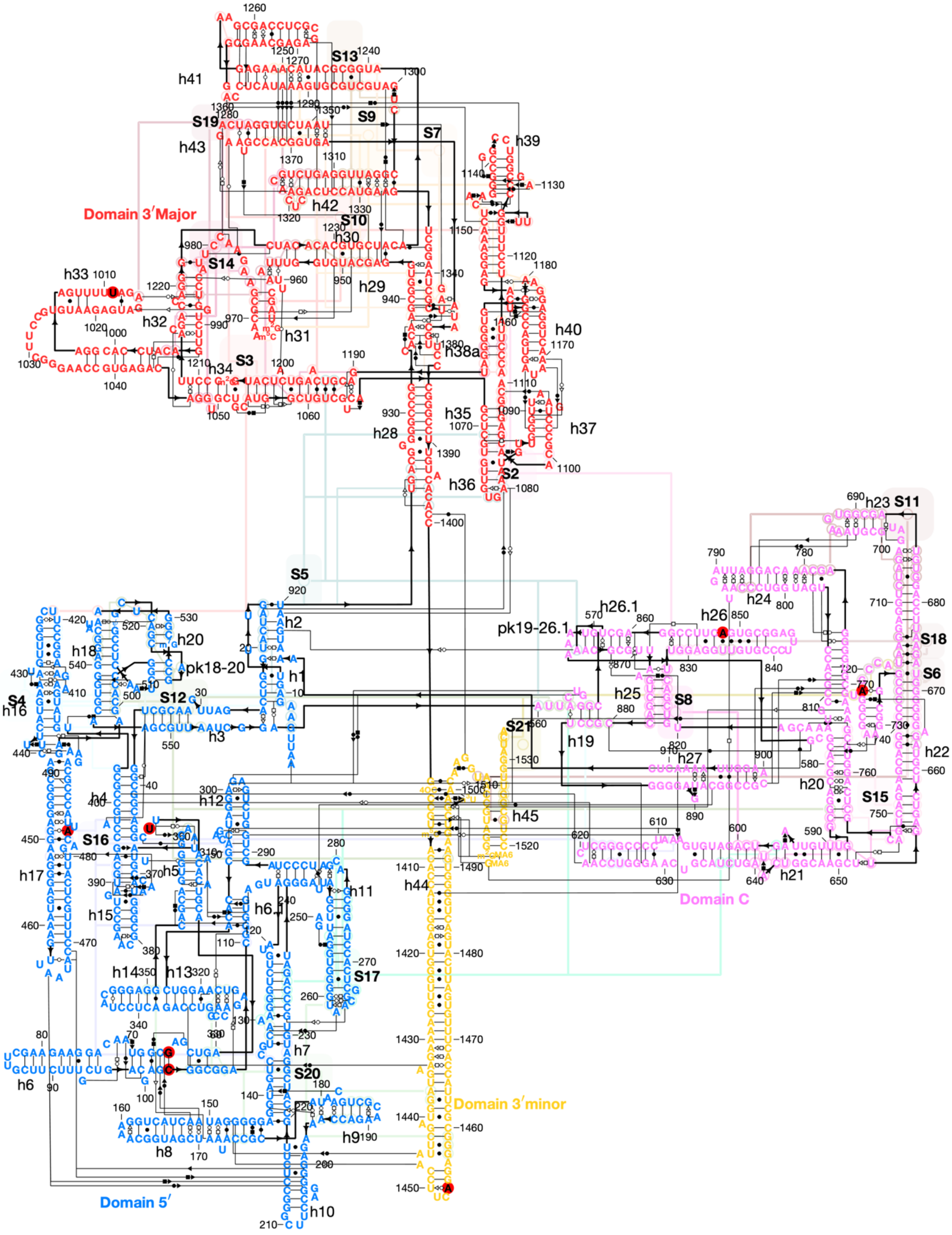

Supplementary Figure 77. Eterna participants'-designed ribosomal RNA design R2-03 prepared with RiboDraw[1]**.**

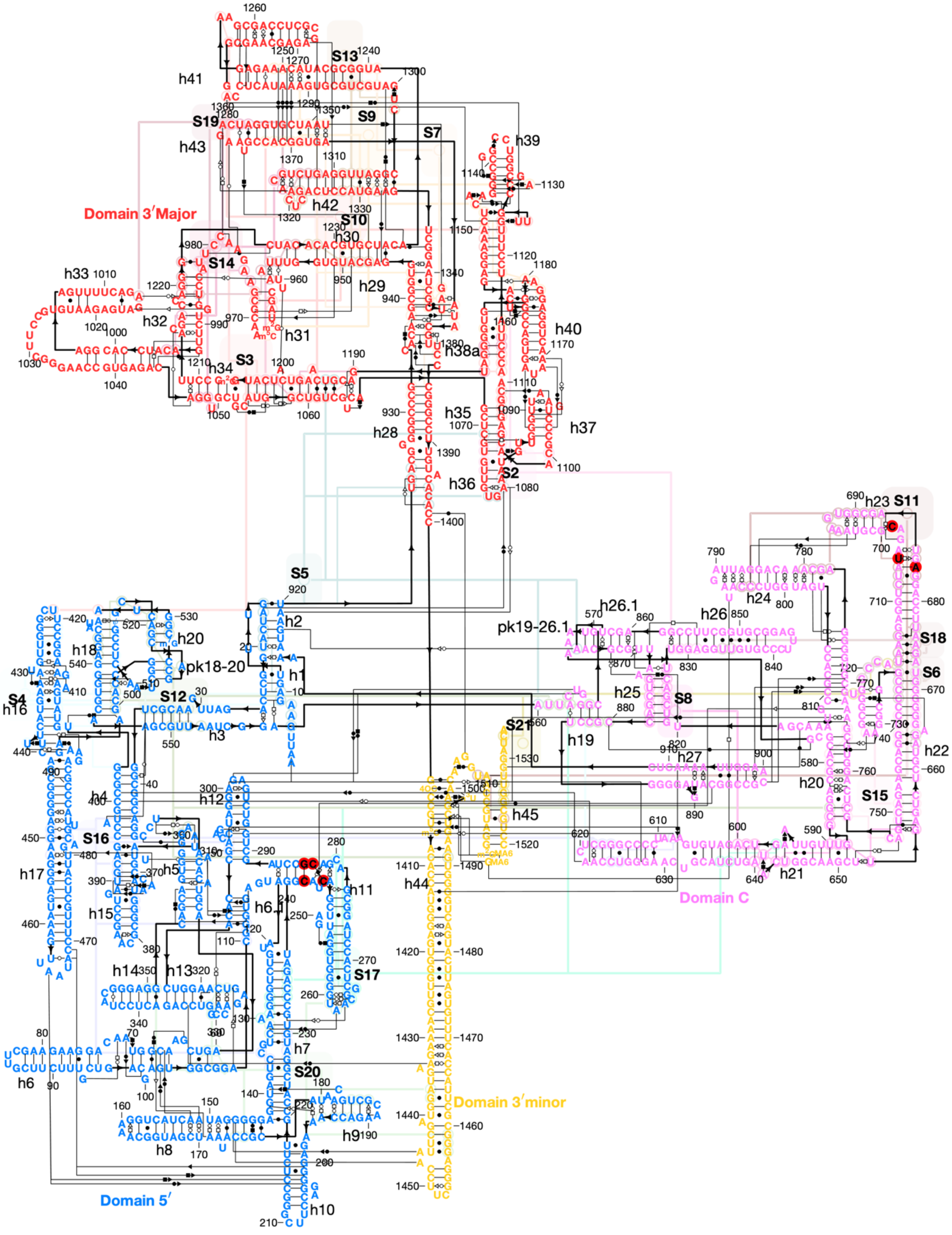

Supplementary Figure 78. Eterna participants'-designed ribosomal RNA design R2-04 prepared with RiboDraw[1].

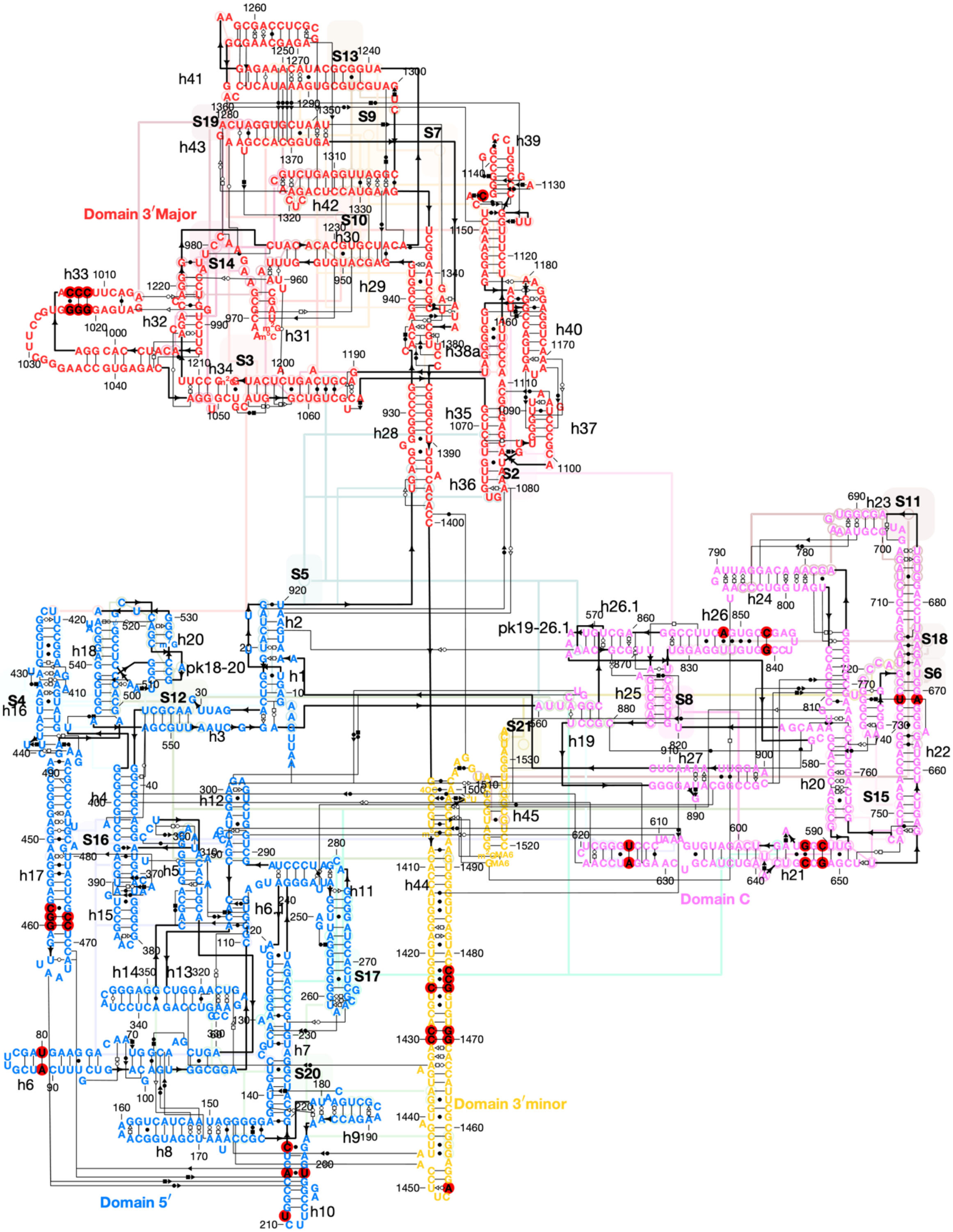

Supplementary Figure 79. Eterna participants'-designed ribosomal RNA design R2-05 prepared with RiboDraw[1].

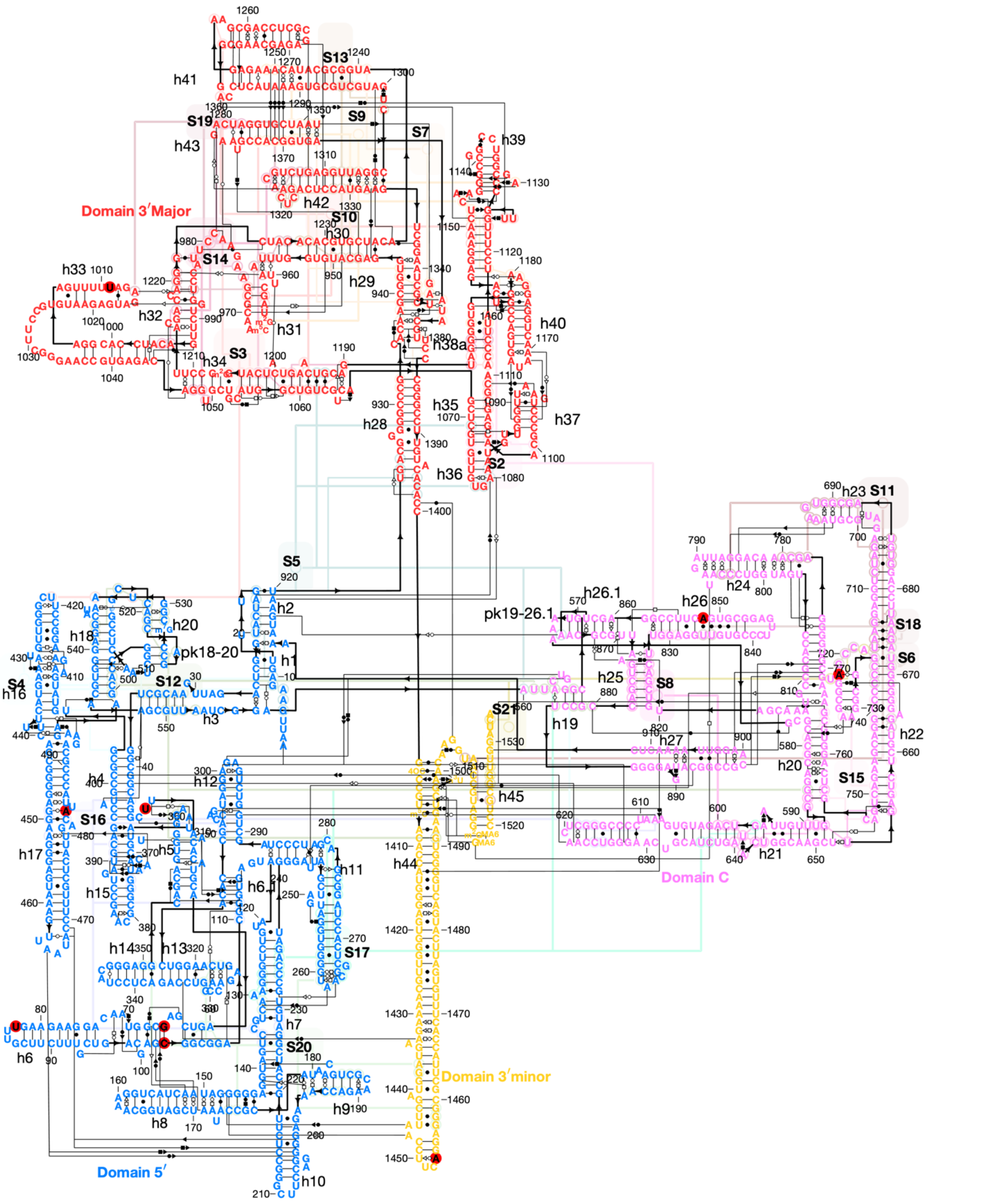

Supplementary Figure 80. Eterna participants'-designed ribosomal RNA design R2-06 prepared with RiboDraw[1].

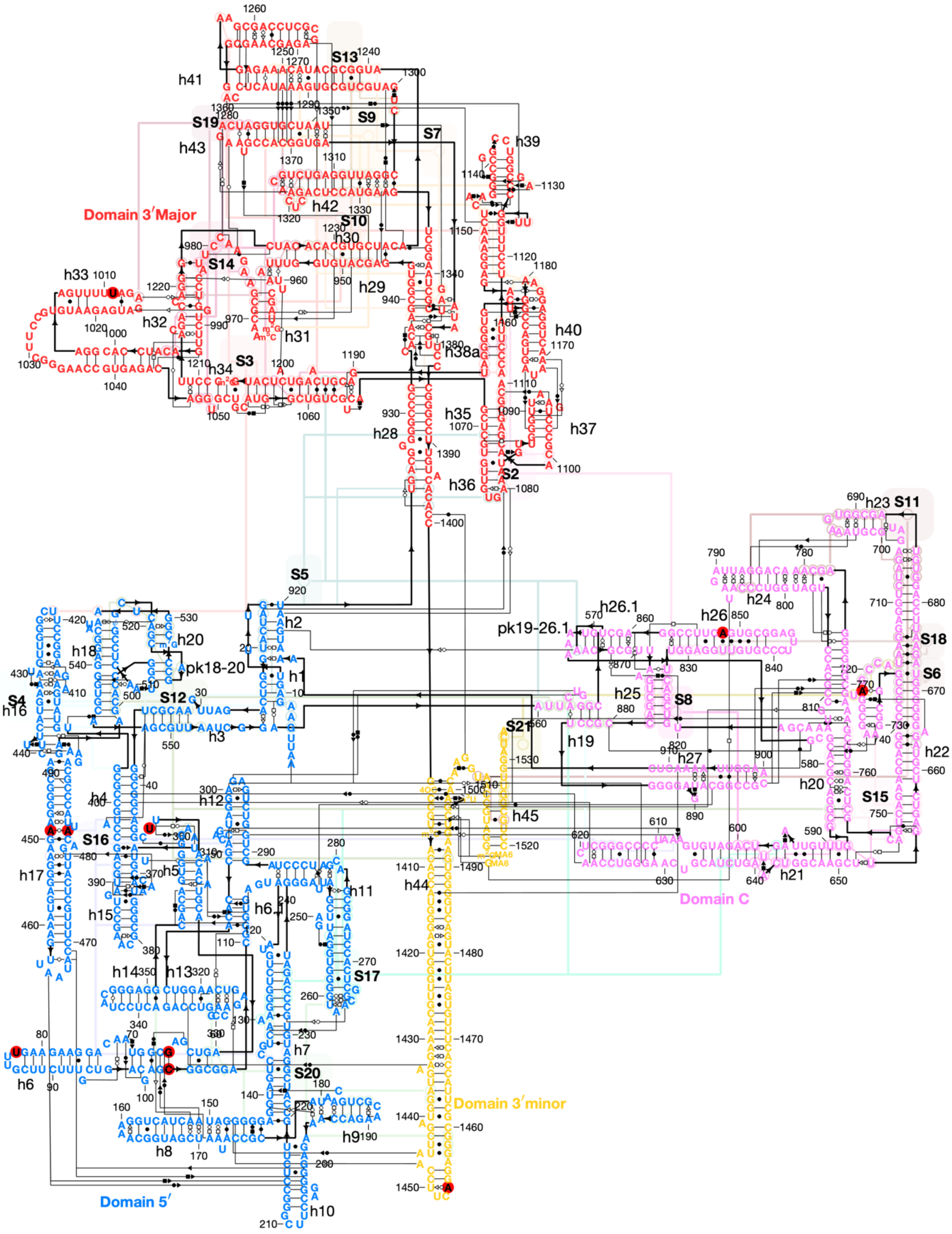

Supplementary Figure 81. Eterna participants'-designed ribosomal RNA design R2-07 prepared with RiboDraw[1].

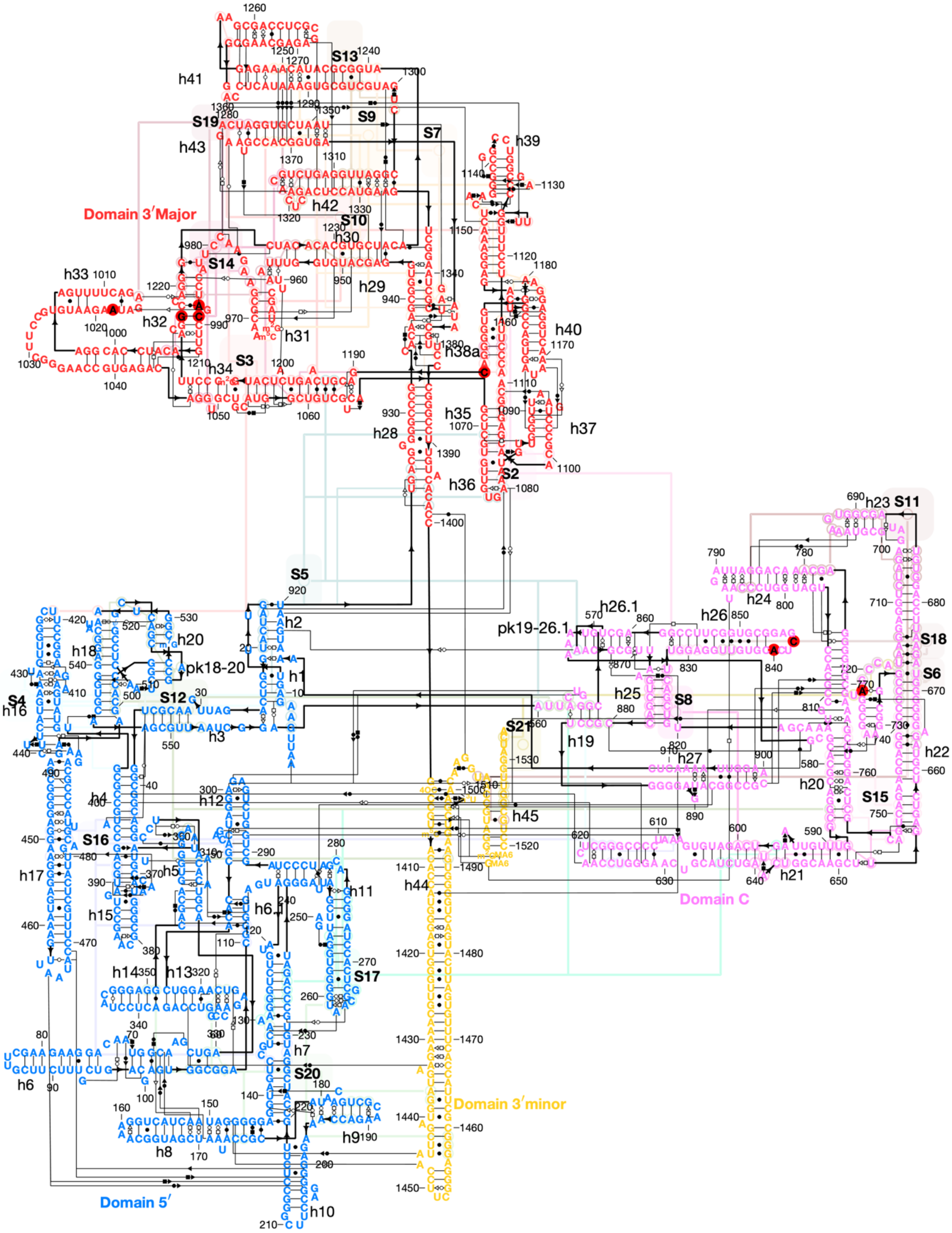

Supplementary Figure 82. Eterna participants'-designed ribosomal RNA design R2-08 prepared with RiboDraw[1].

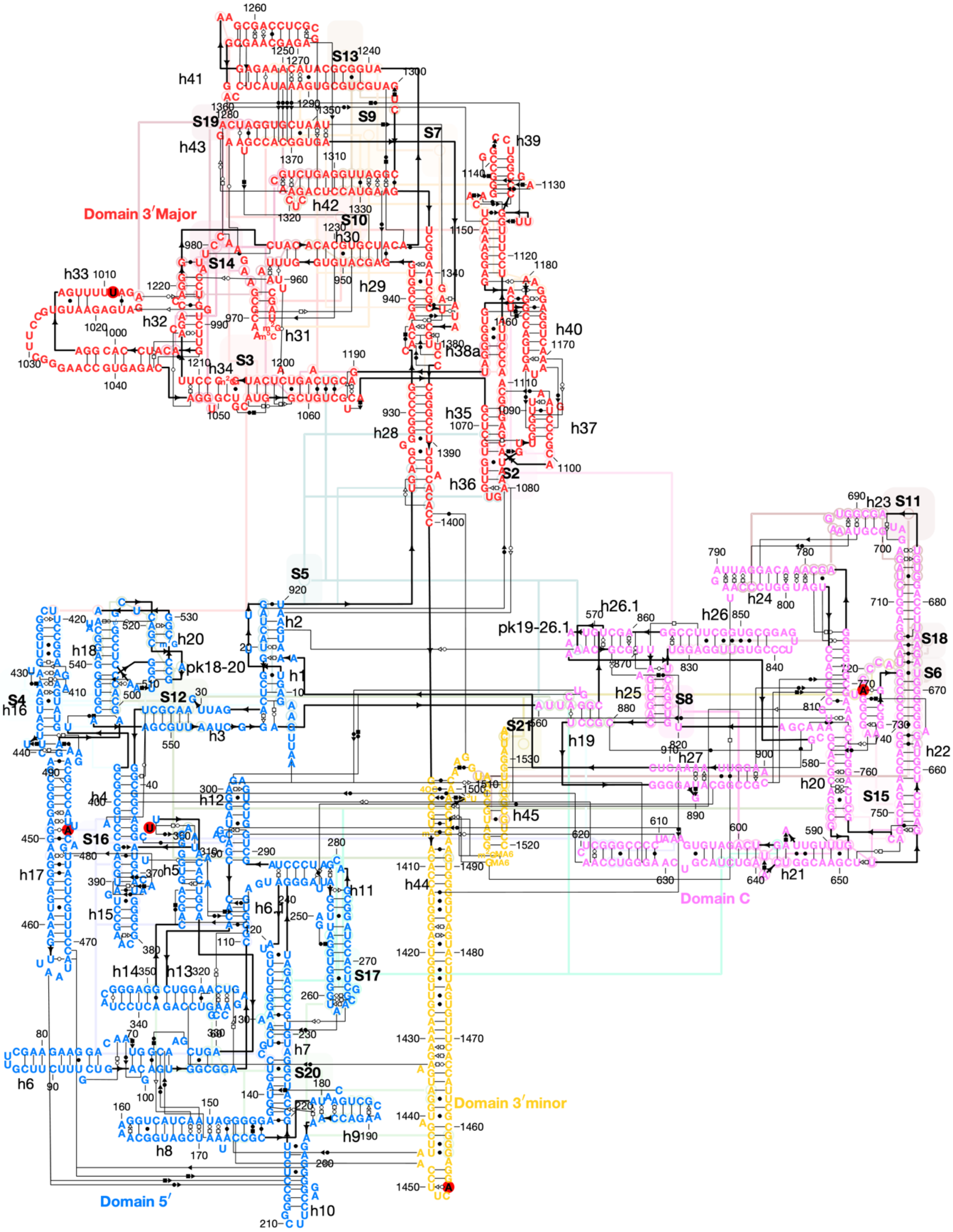

Supplementary Figure 83. Eterna participants'-designed ribosomal RNA design R2-09 prepared with RiboDraw[1].

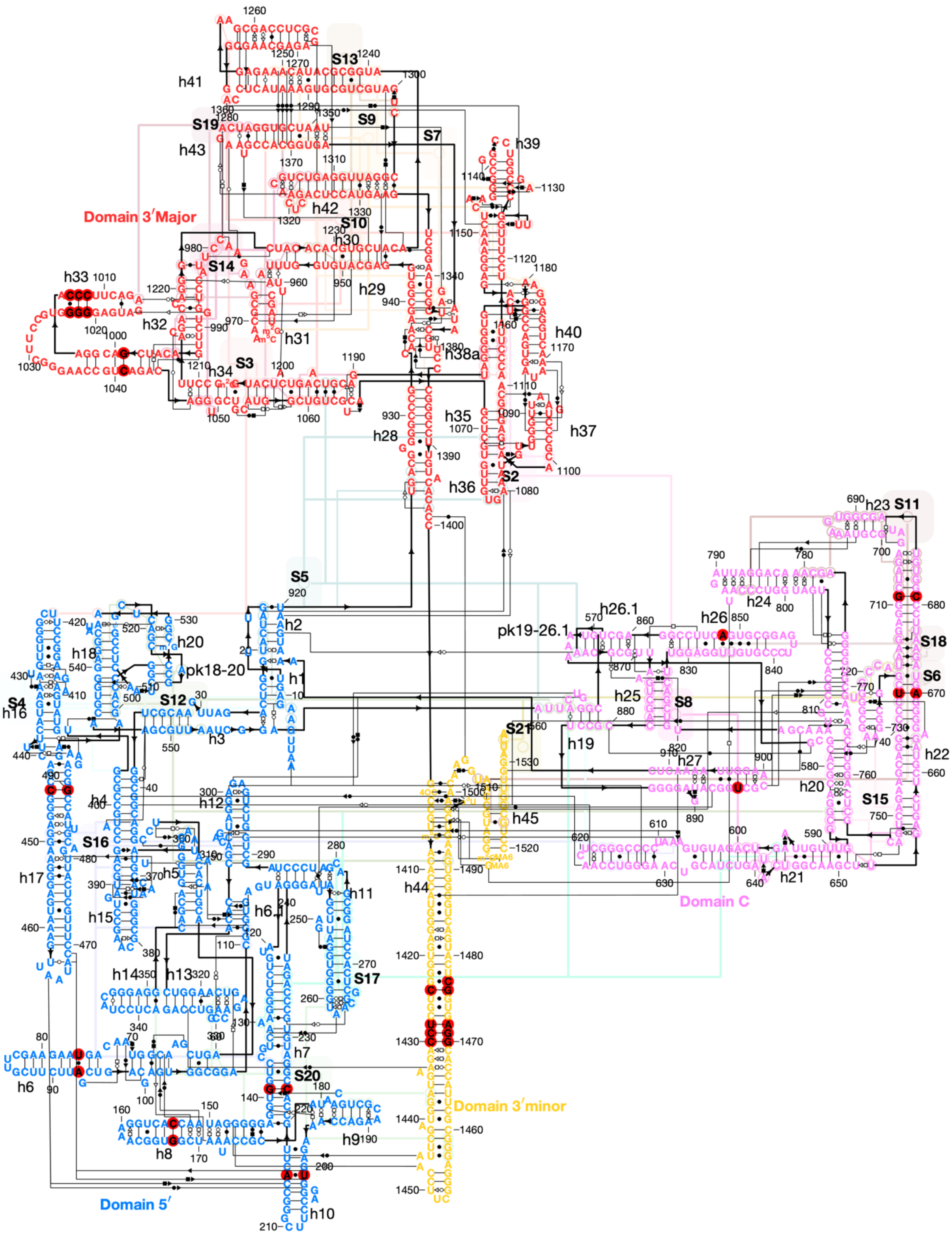

Supplementary Figure 84. Eterna participants'-designed ribosomal RNA design R2-10 prepared with RiboDraw[1].

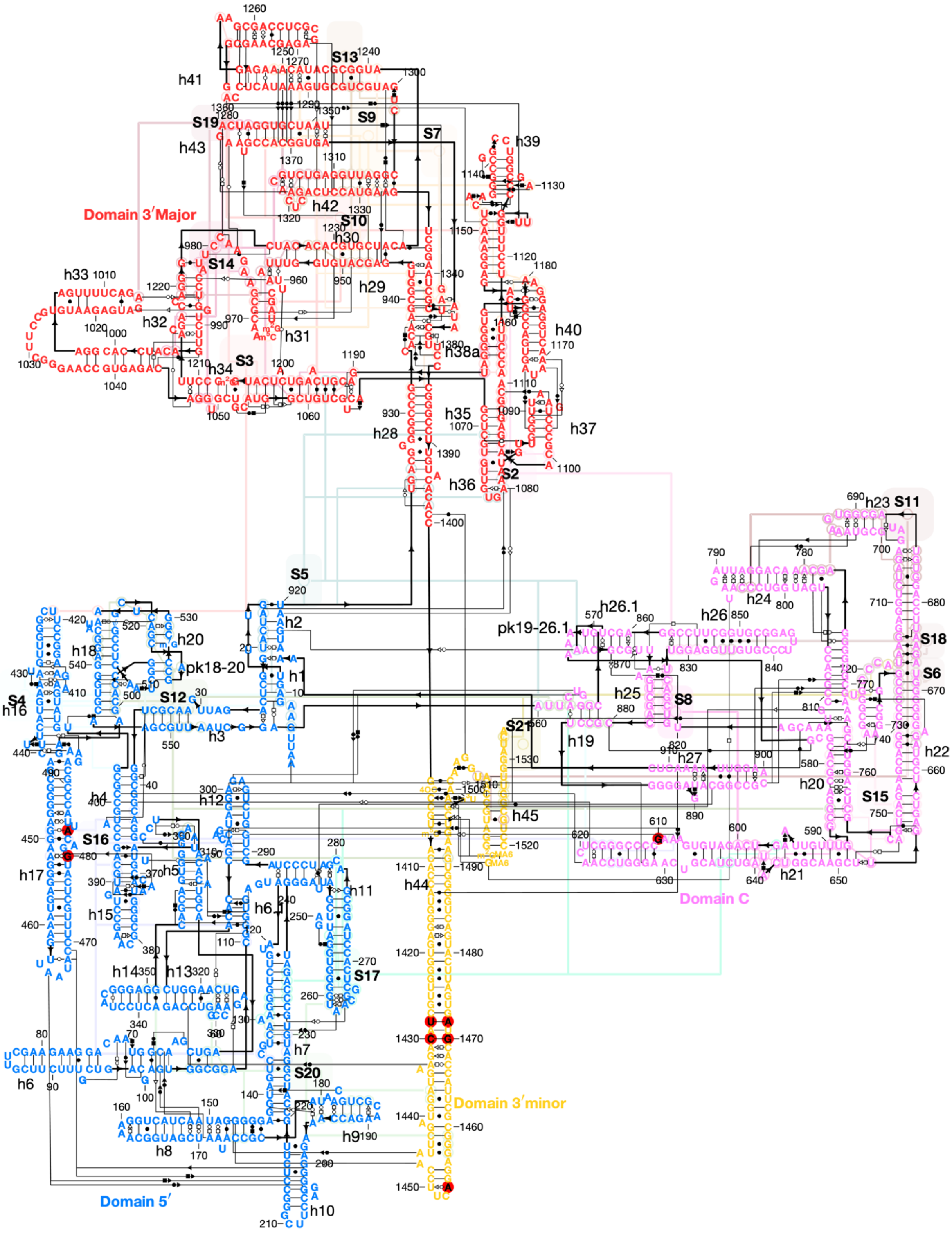

Supplementary Figure 85. Eterna participants'-designed ribosomal RNA design R2-11 prepared with RiboDraw[1].

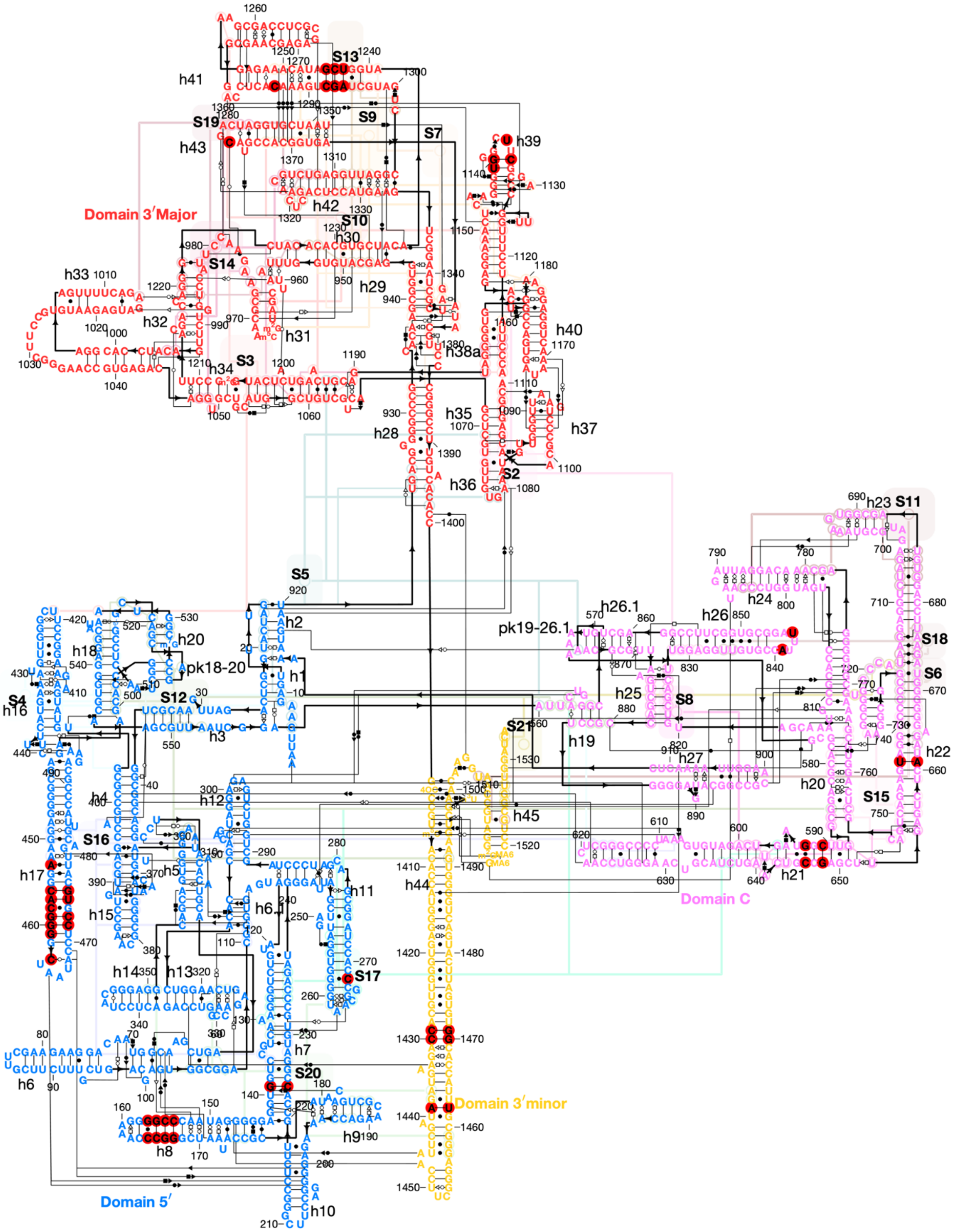

Supplementary Figure 86. Eterna participants'-designed ribosomal RNA design R2-12 prepared with RiboDraw[1].

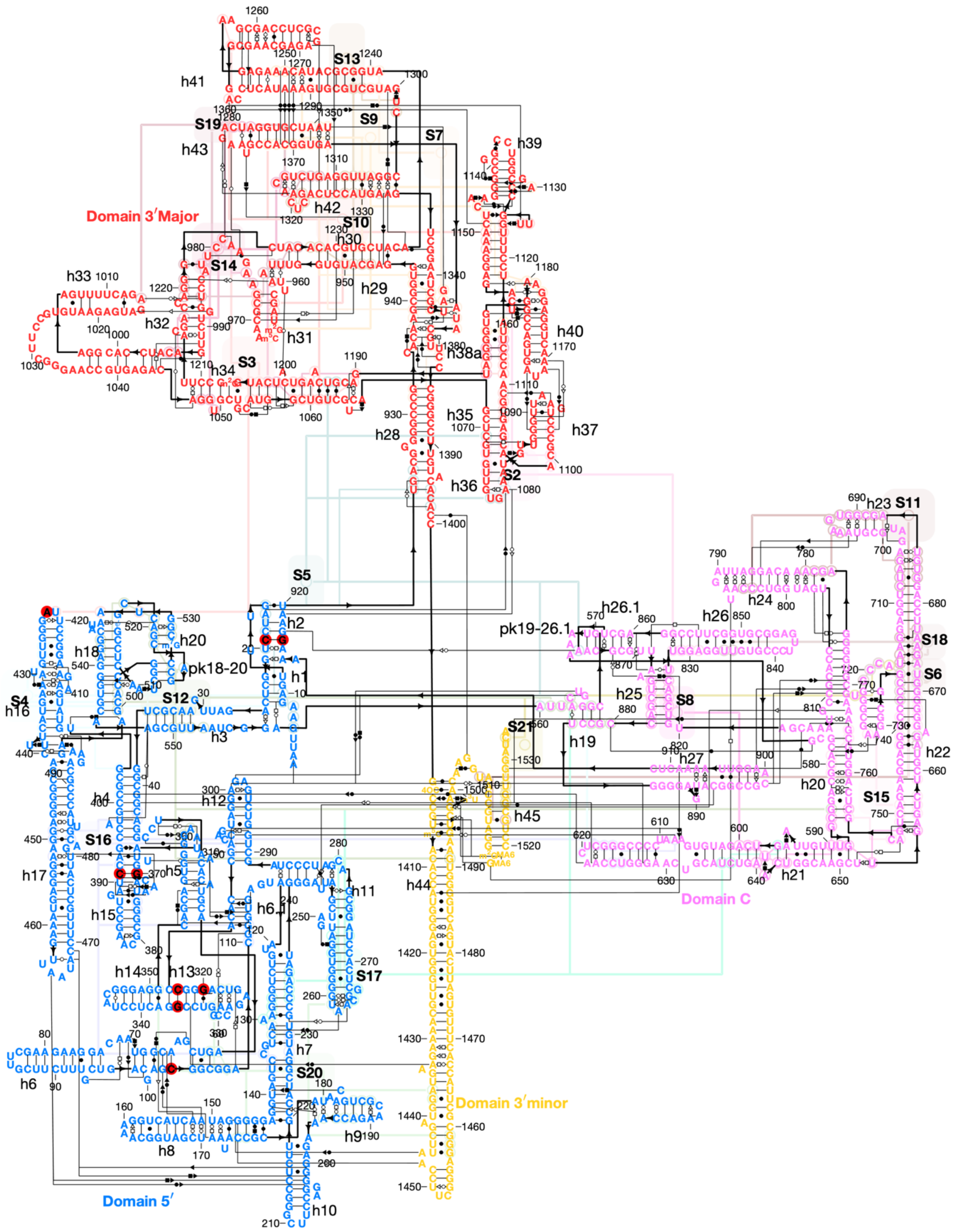

Supplementary Figure 87. Eterna participants'-designed ribosomal RNA design R2-13 prepared with RiboDraw[1].

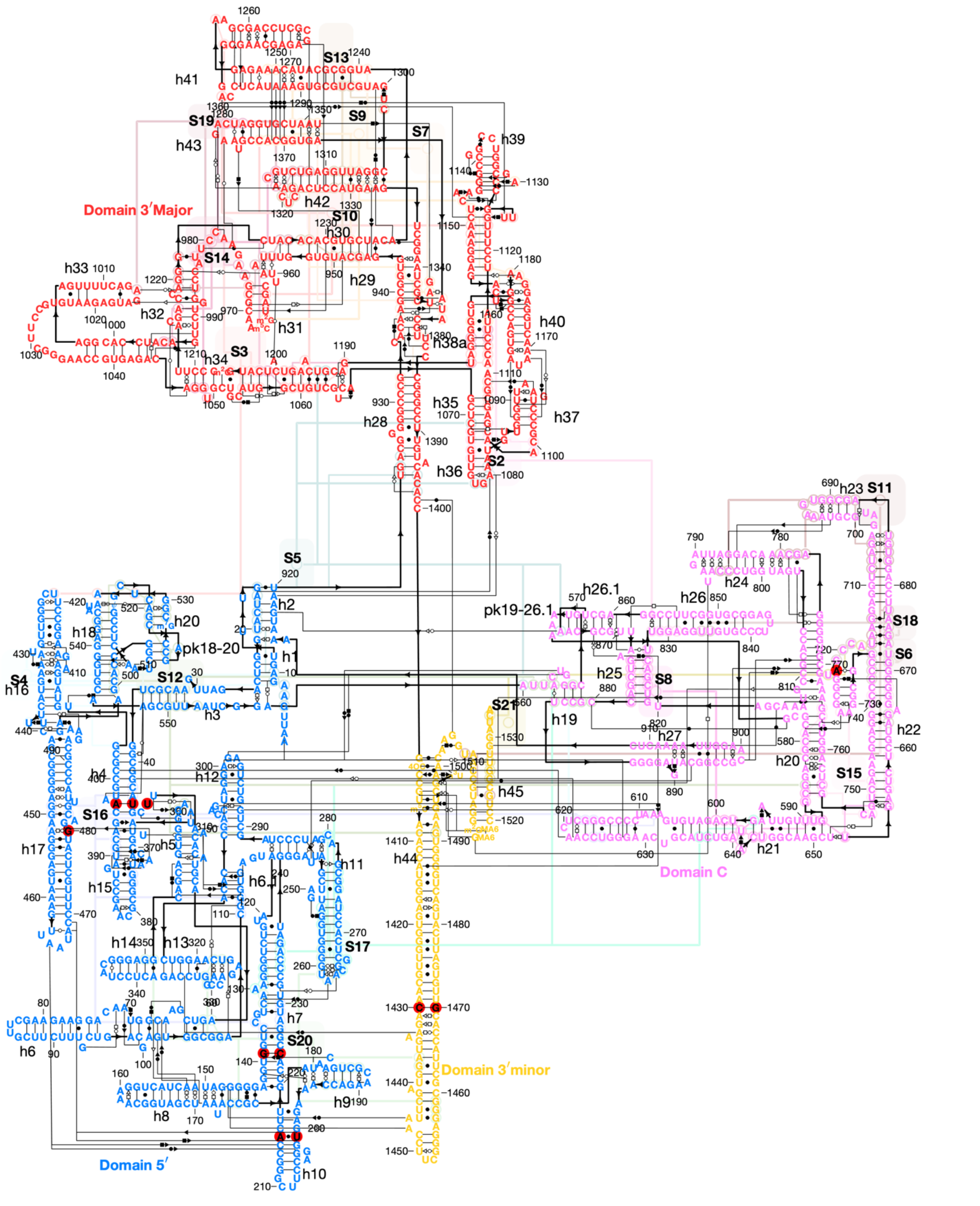

Supplementary Figure 88. Eterna participants'-designed ribosomal RNA design R2-14 prepared with RiboDraw[1].

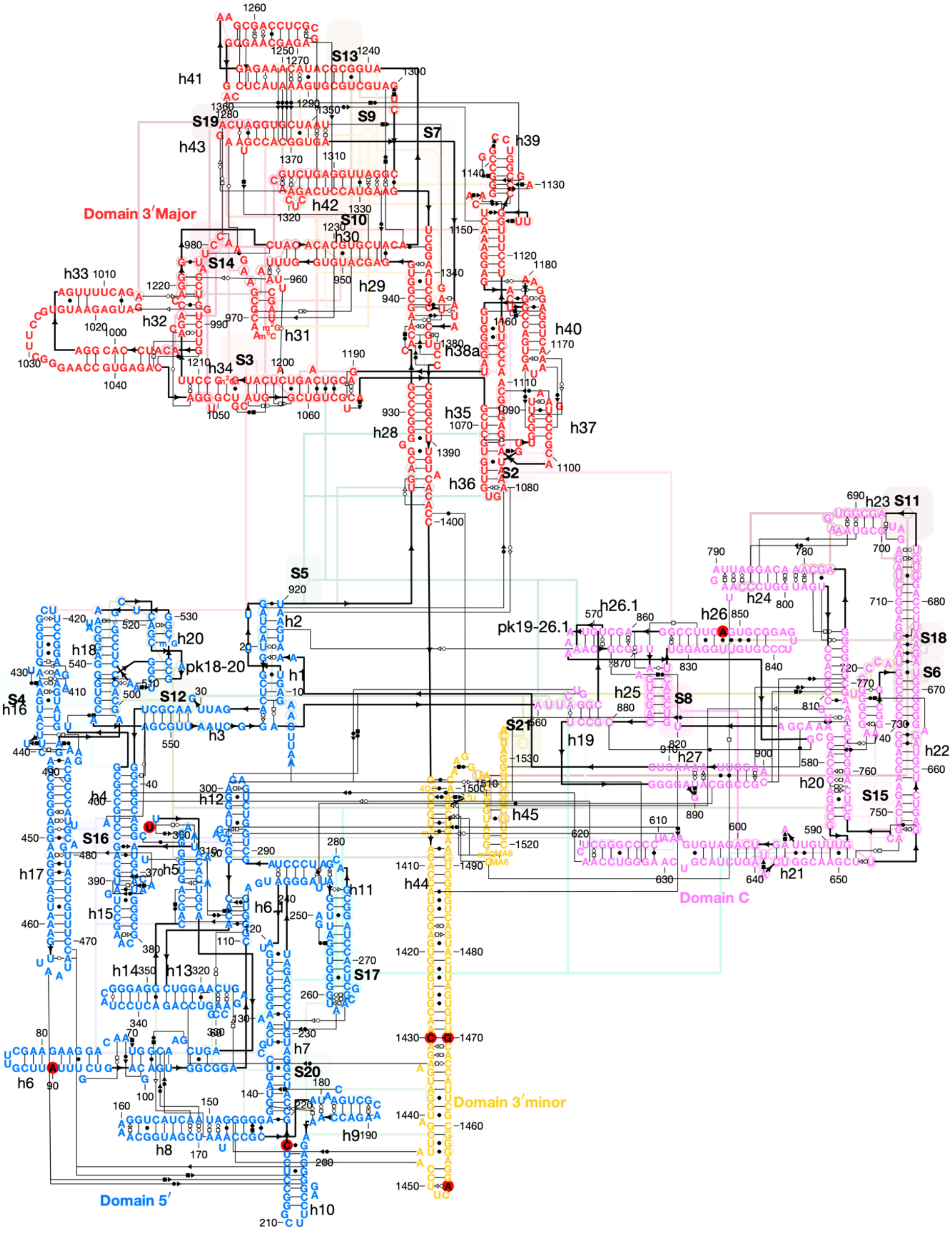

Supplementary Figure 89. Eterna participants'-designed ribosomal RNA design R2-15 prepared with RiboDraw[1]**.**

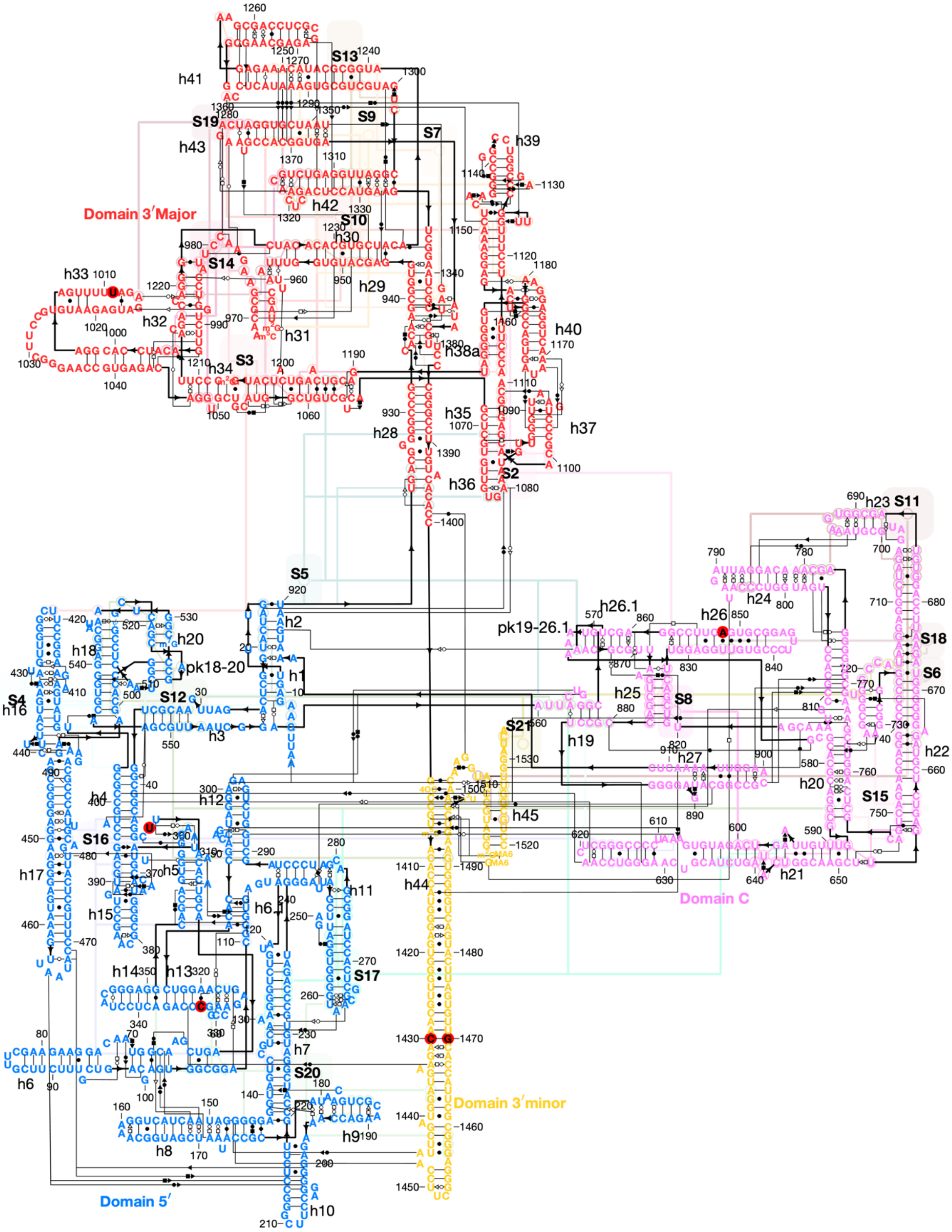

Supplementary Figure 90. Eterna participants'-designed ribosomal RNA design R2-16 prepared with RiboDraw[1].

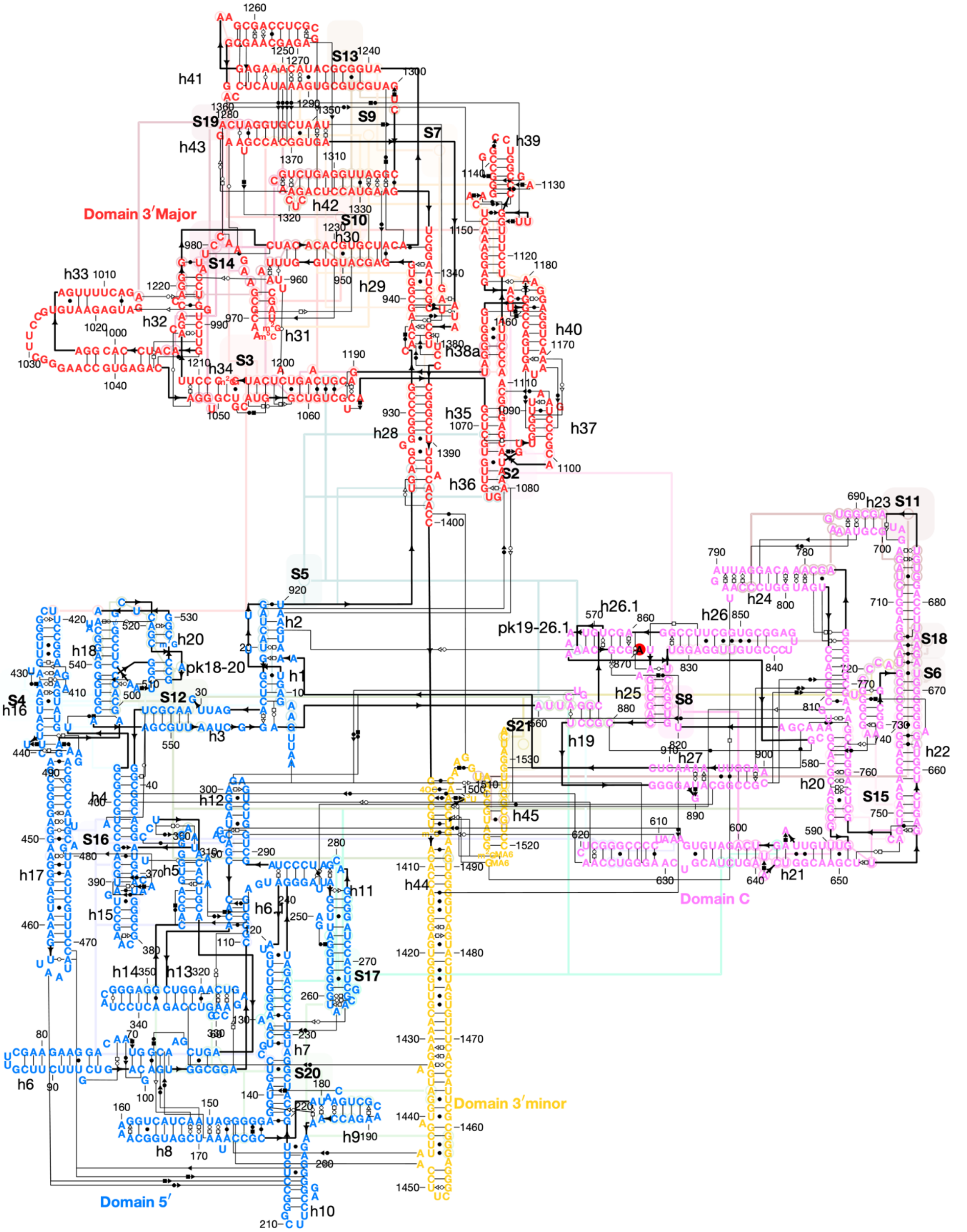

Supplementary Figure 91. Eterna participants'-designed ribosomal RNA design R2-17 prepared with RiboDraw[1].

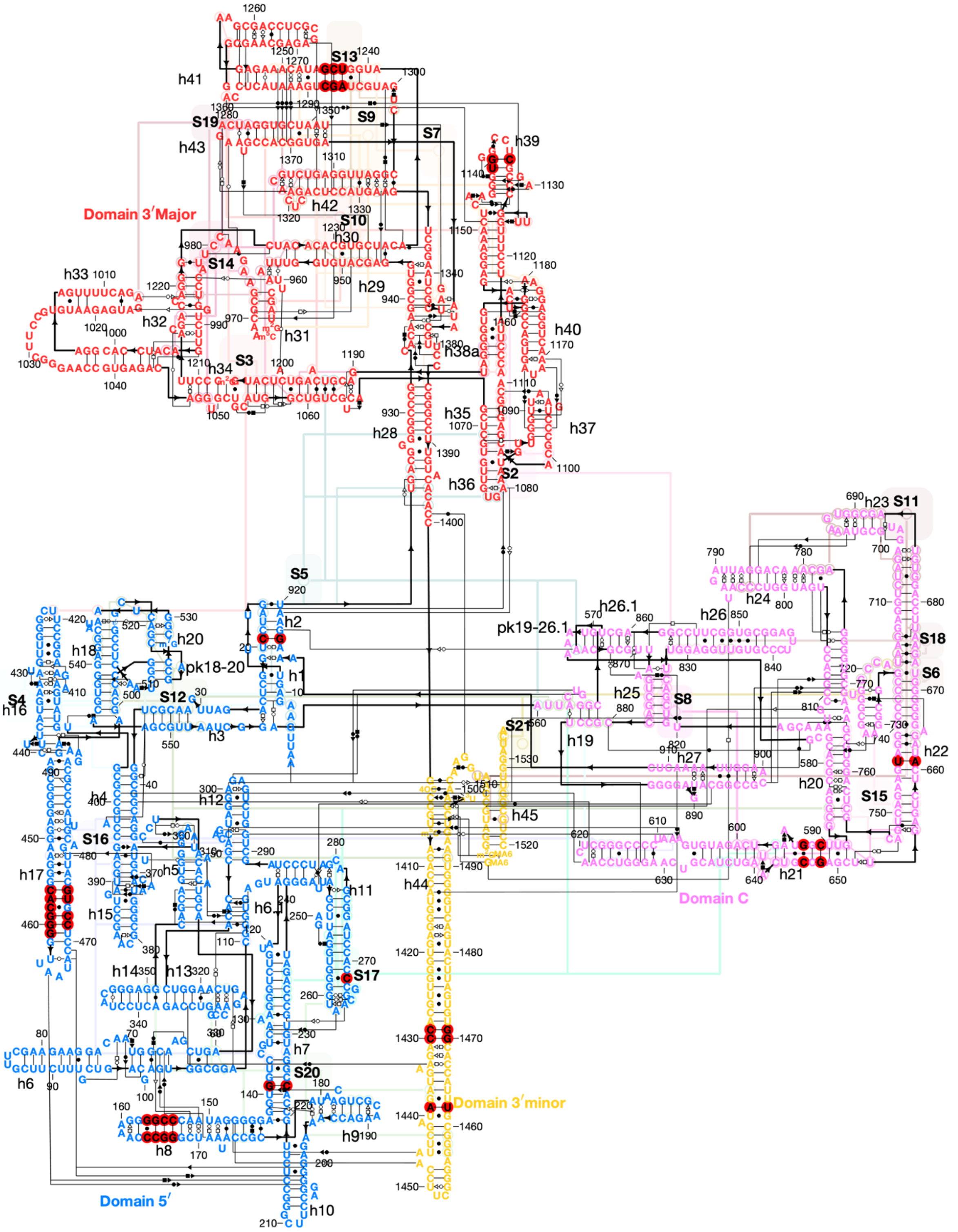

Supplementary Figure 92. Eterna participants'-designed ribosomal RNA design R2-18 prepared with RiboDraw[1].

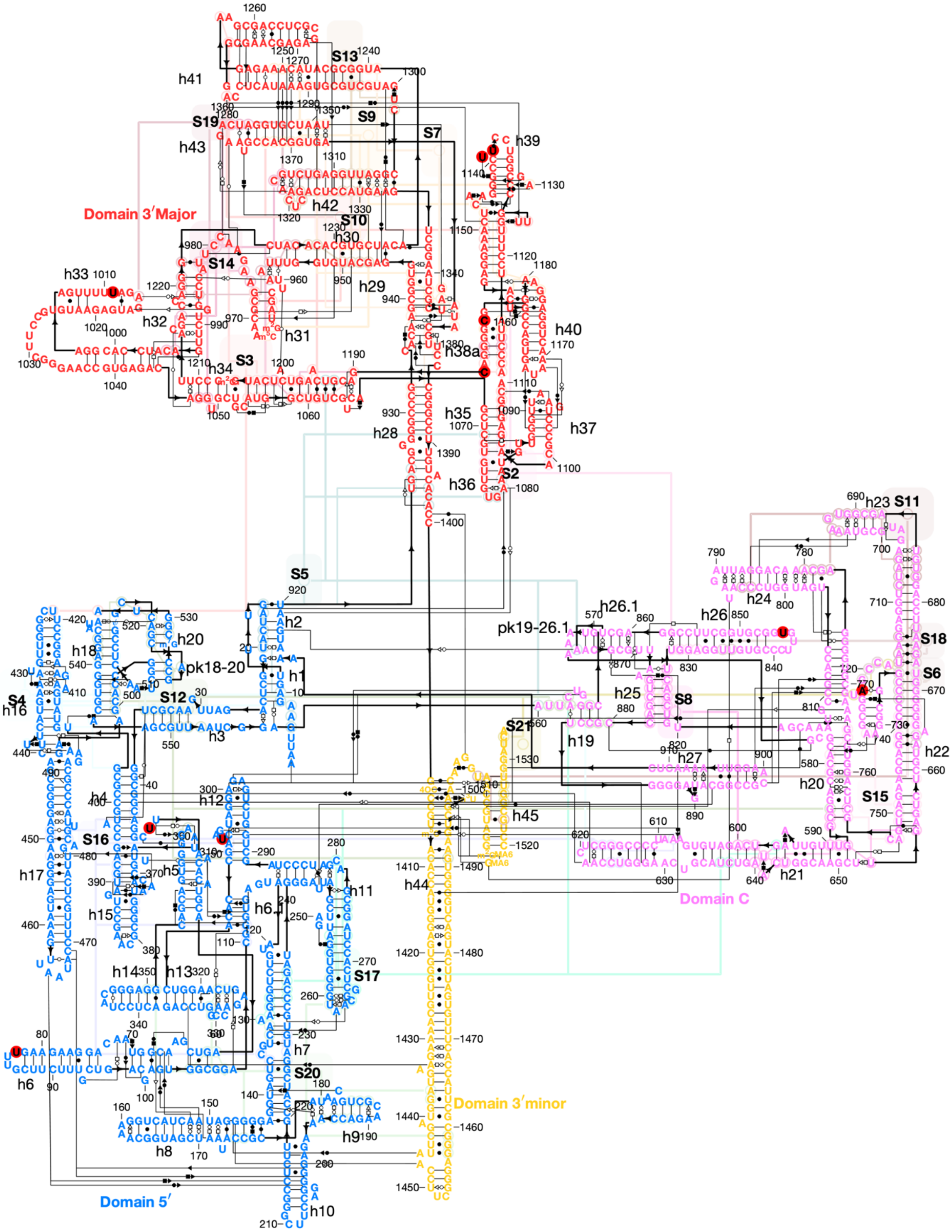

Supplementary Figure 93. Eterna participants'-designed ribosomal RNA design R2-19 prepared with RiboDraw[1].

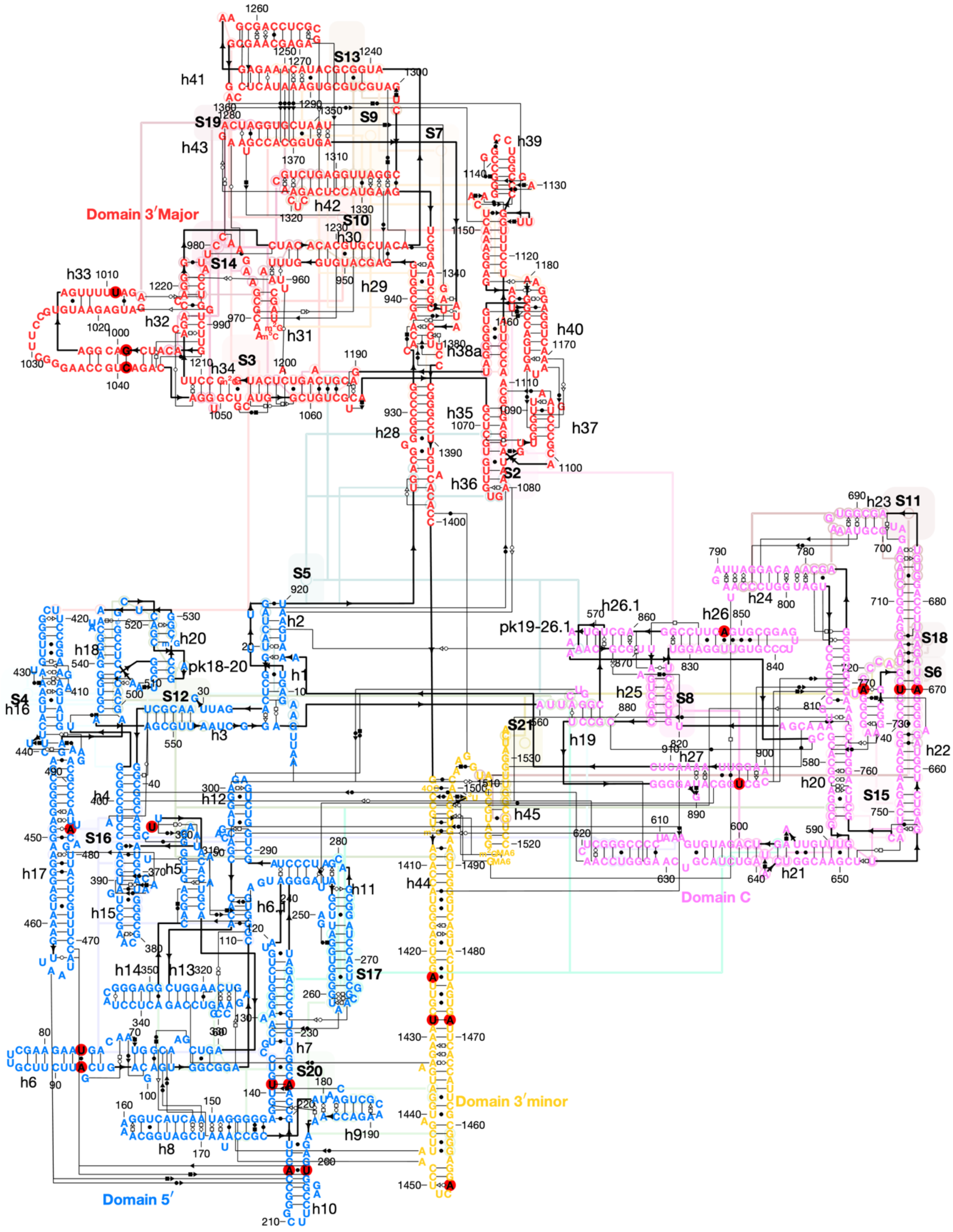

Supplementary Figure 94. Eterna participants'-designed ribosomal RNA design R2-20 prepared with RiboDraw[1].

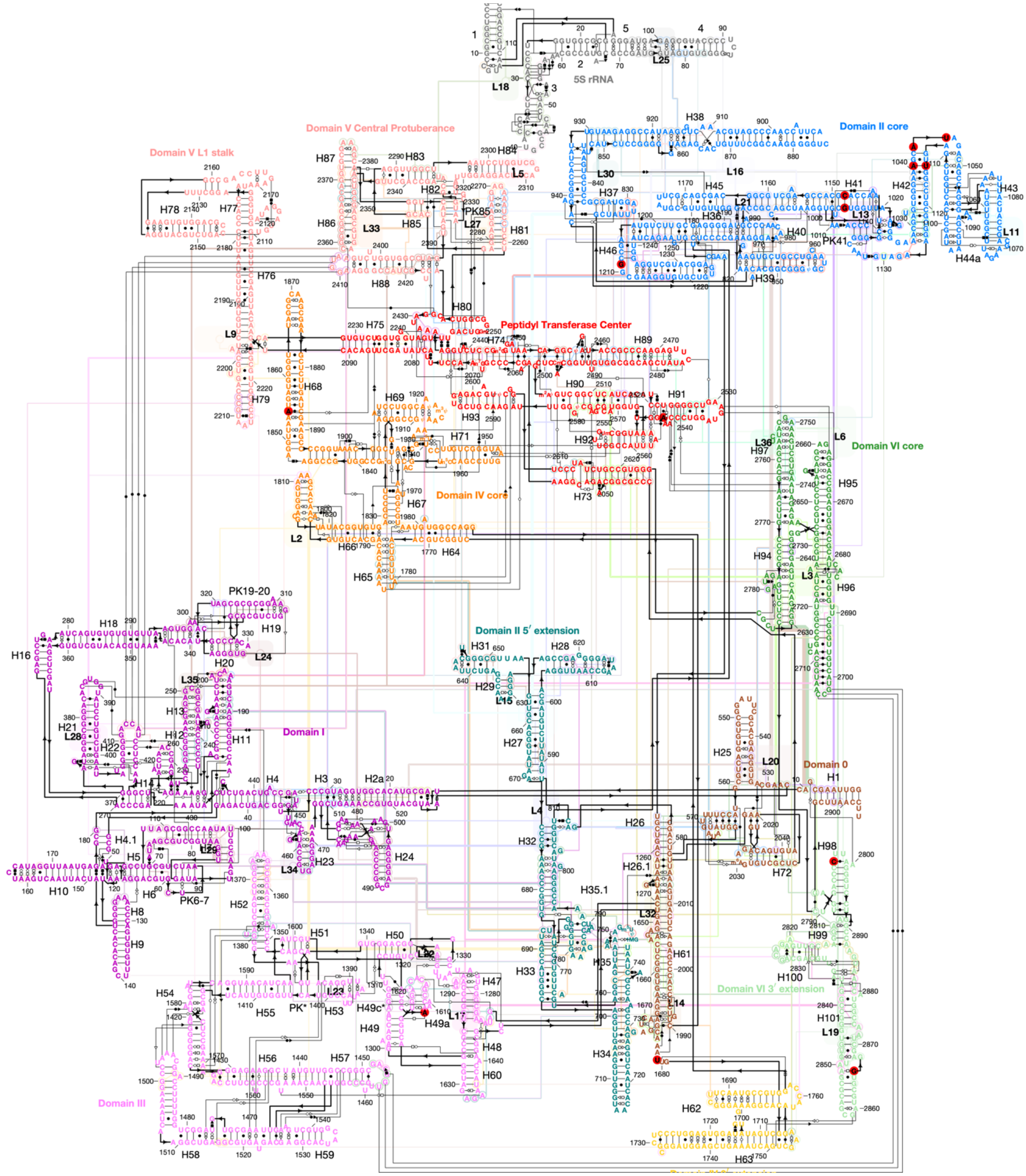

Supplementary Figure 95. Eterna participants'-designed ribosomal RNA design R2-21 prepared with RiboDraw[1].

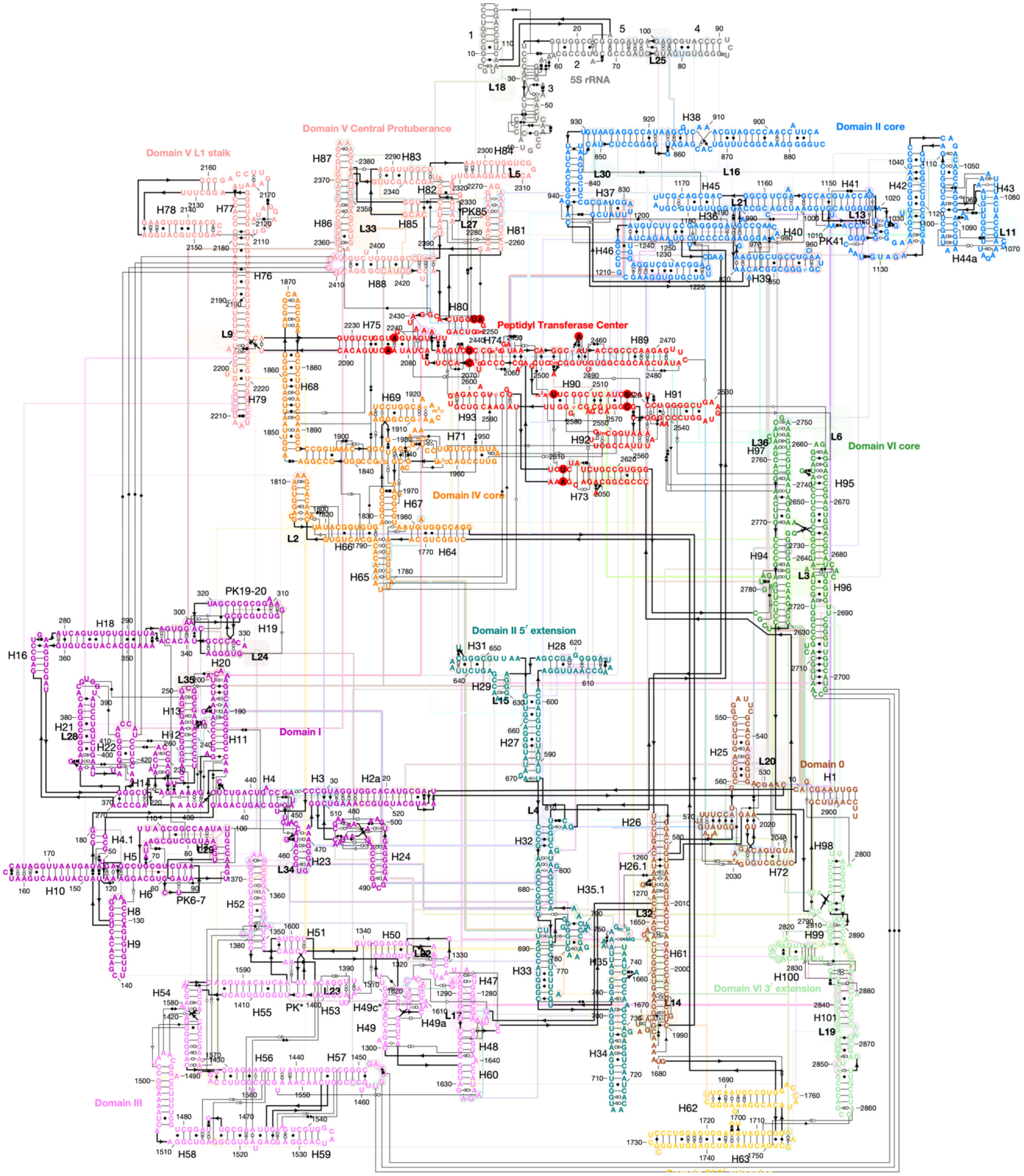

Supplementary Figure 96. Eterna participants'-designed ribosomal RNA design R2-22 prepared with RiboDraw[1].

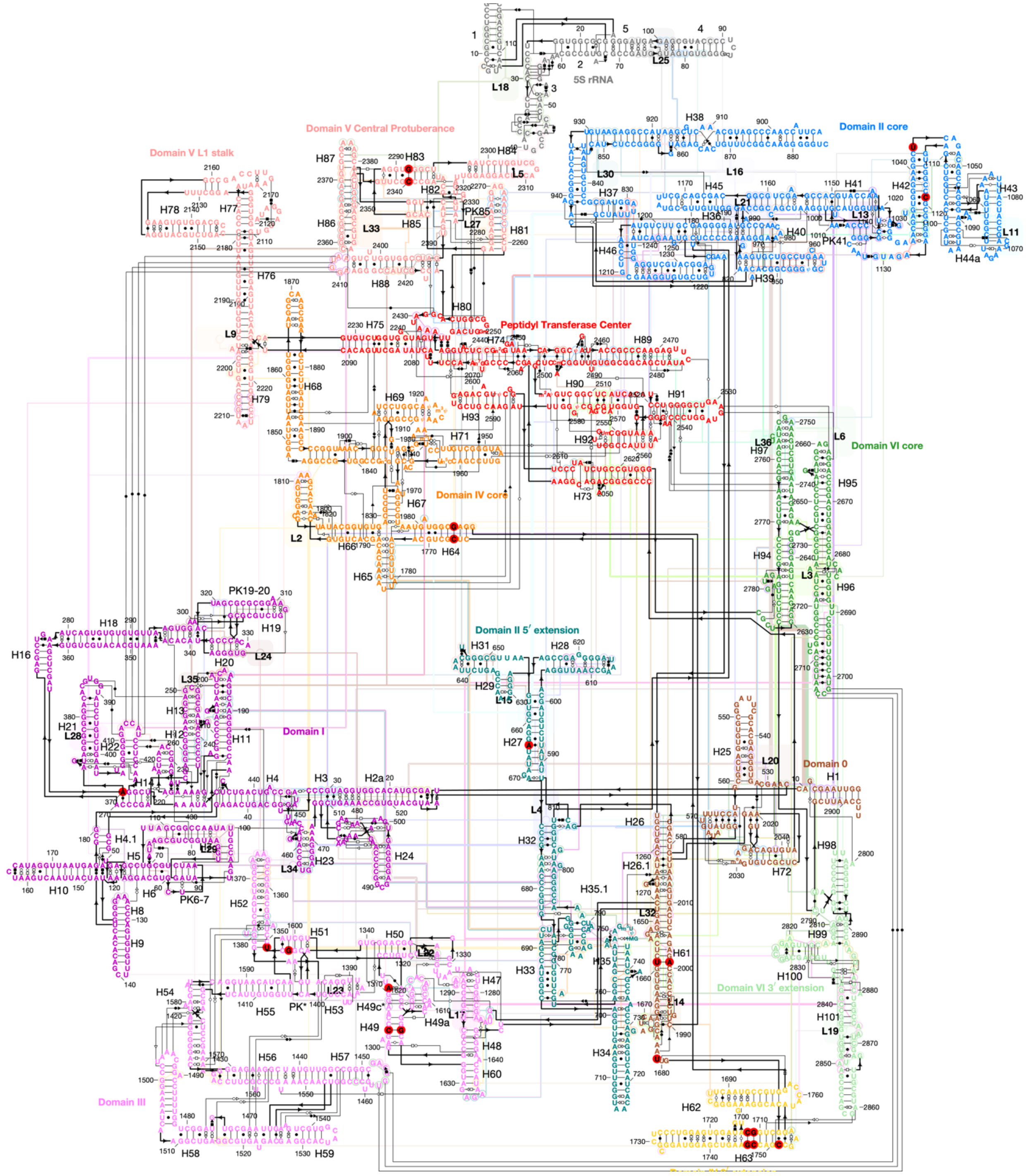

Supplementary Figure 97. Eterna participants'-designed ribosomal RNA design R2-23 prepared with RiboDraw[1].

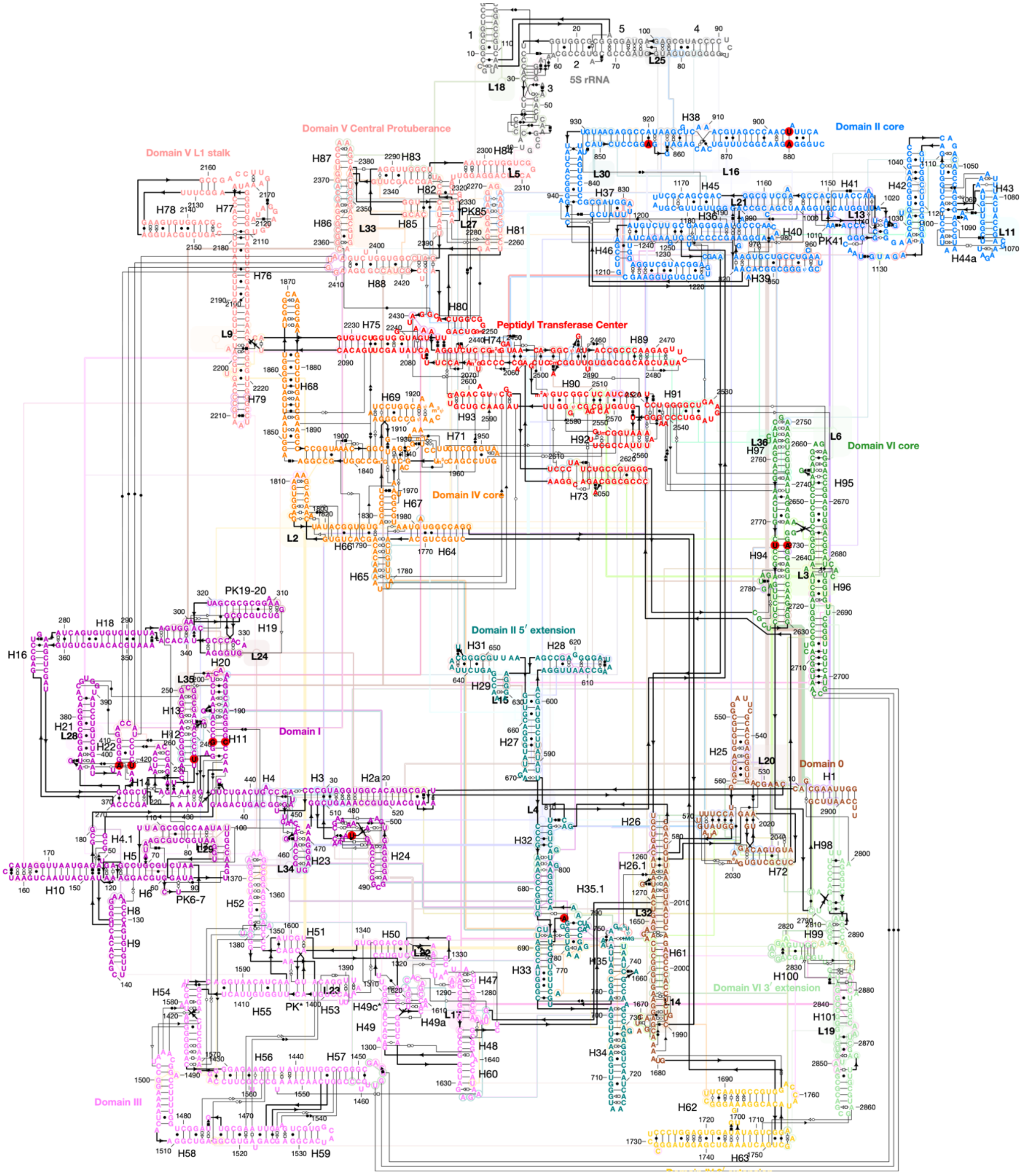

Supplementary Figure 98. Eterna participants'-designed ribosomal RNA design R2-24 prepared with RiboDraw[1].

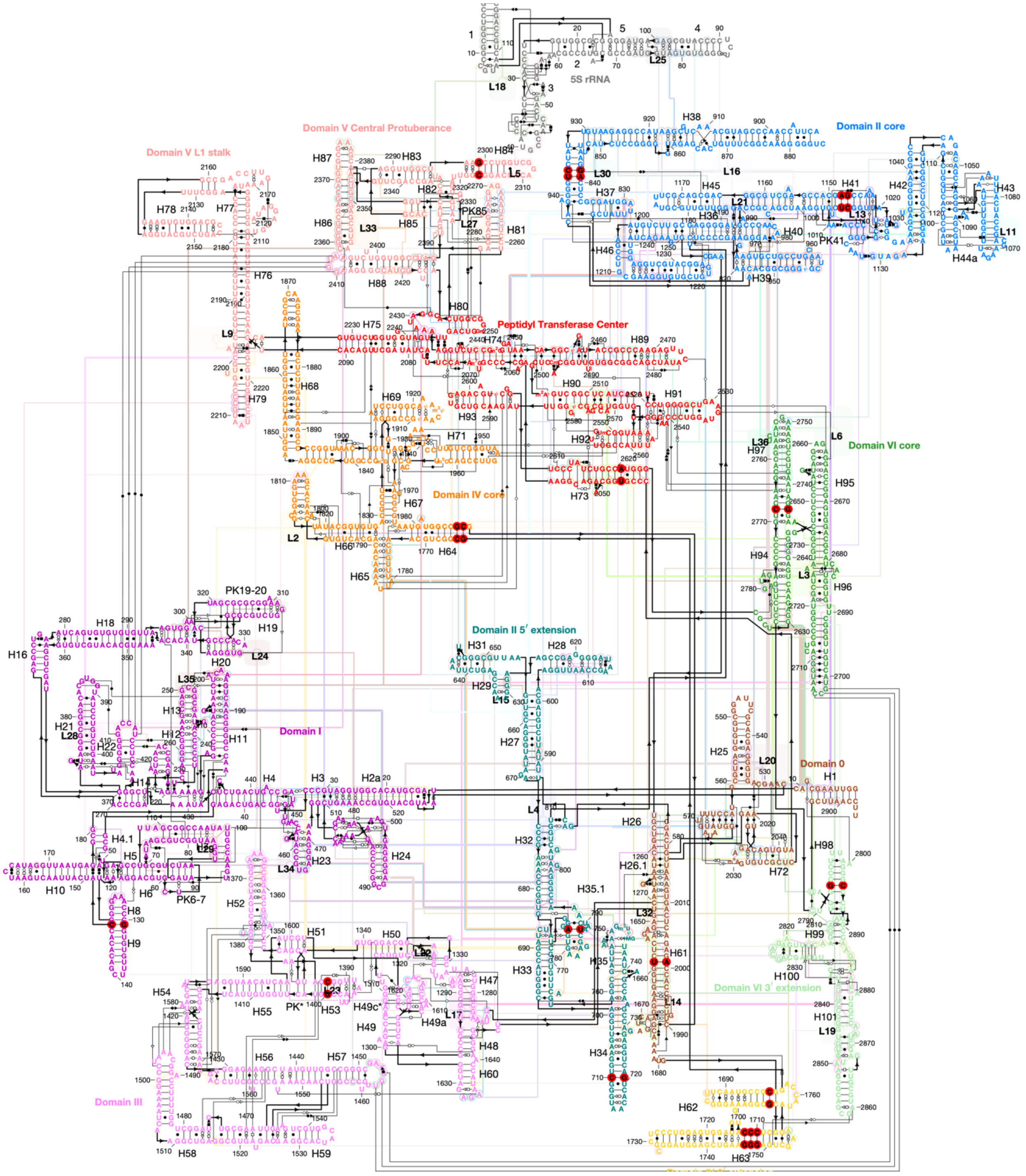

Supplementary Figure 99. Eterna participants'-designed ribosomal RNA design R2-25 prepared with RiboDraw[1].

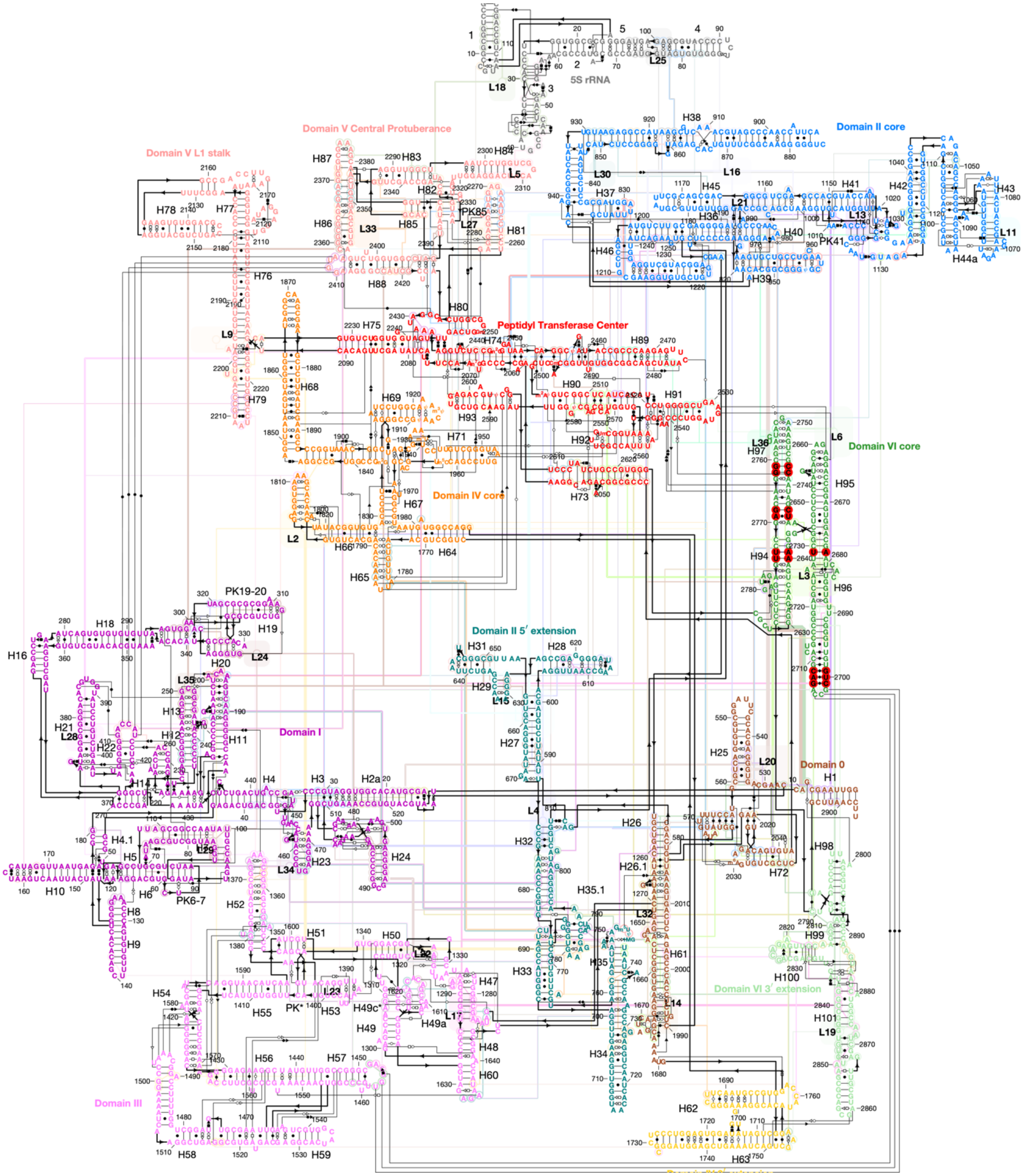

Supplementary Figure 100. Eterna participants'-designed ribosomal RNA design R2-26 prepared with RiboDraw[1].

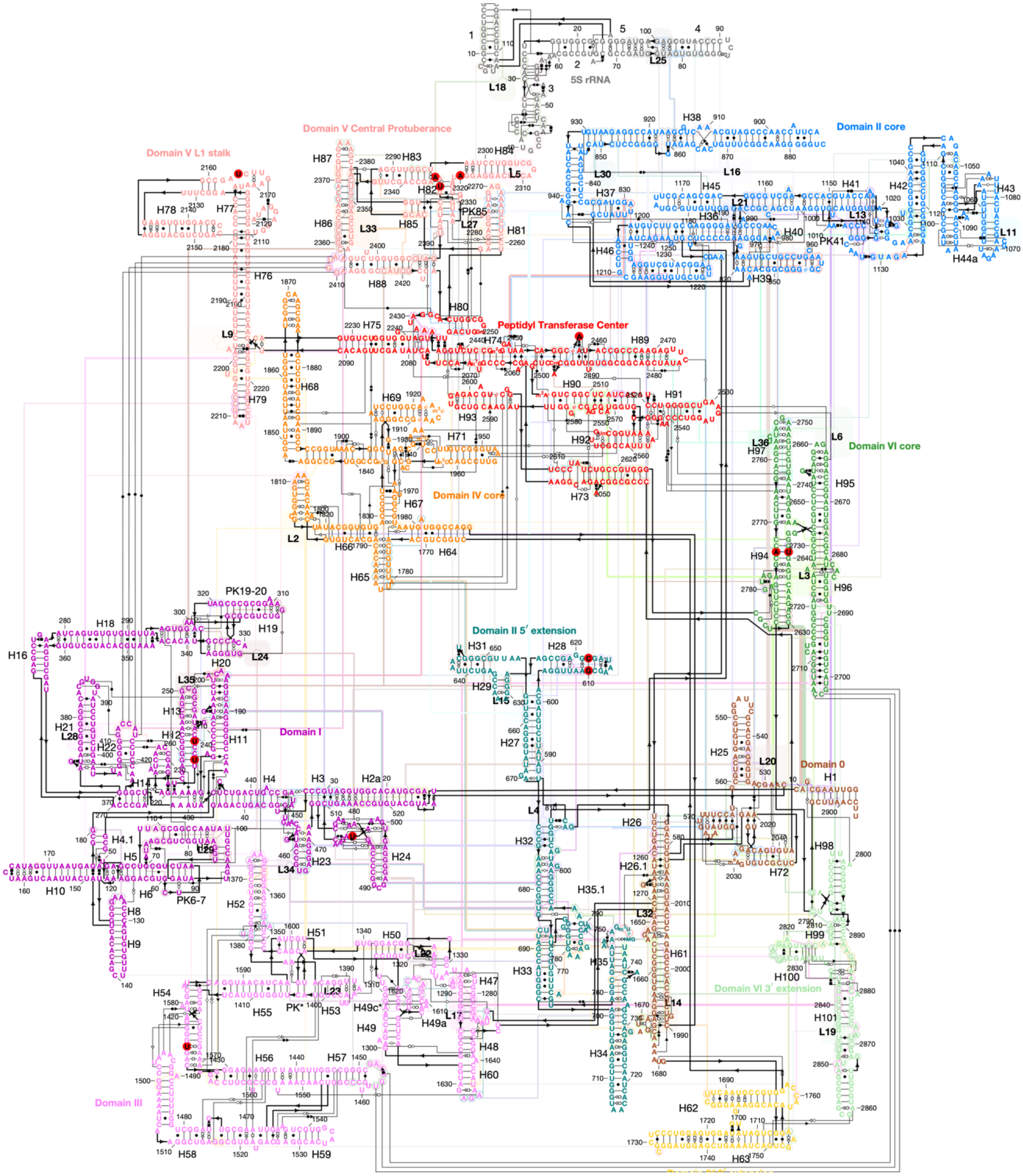

Supplementary Figure 101. Eterna participants'-designed ribosomal RNA design R2-27 prepared with RiboDraw[1].

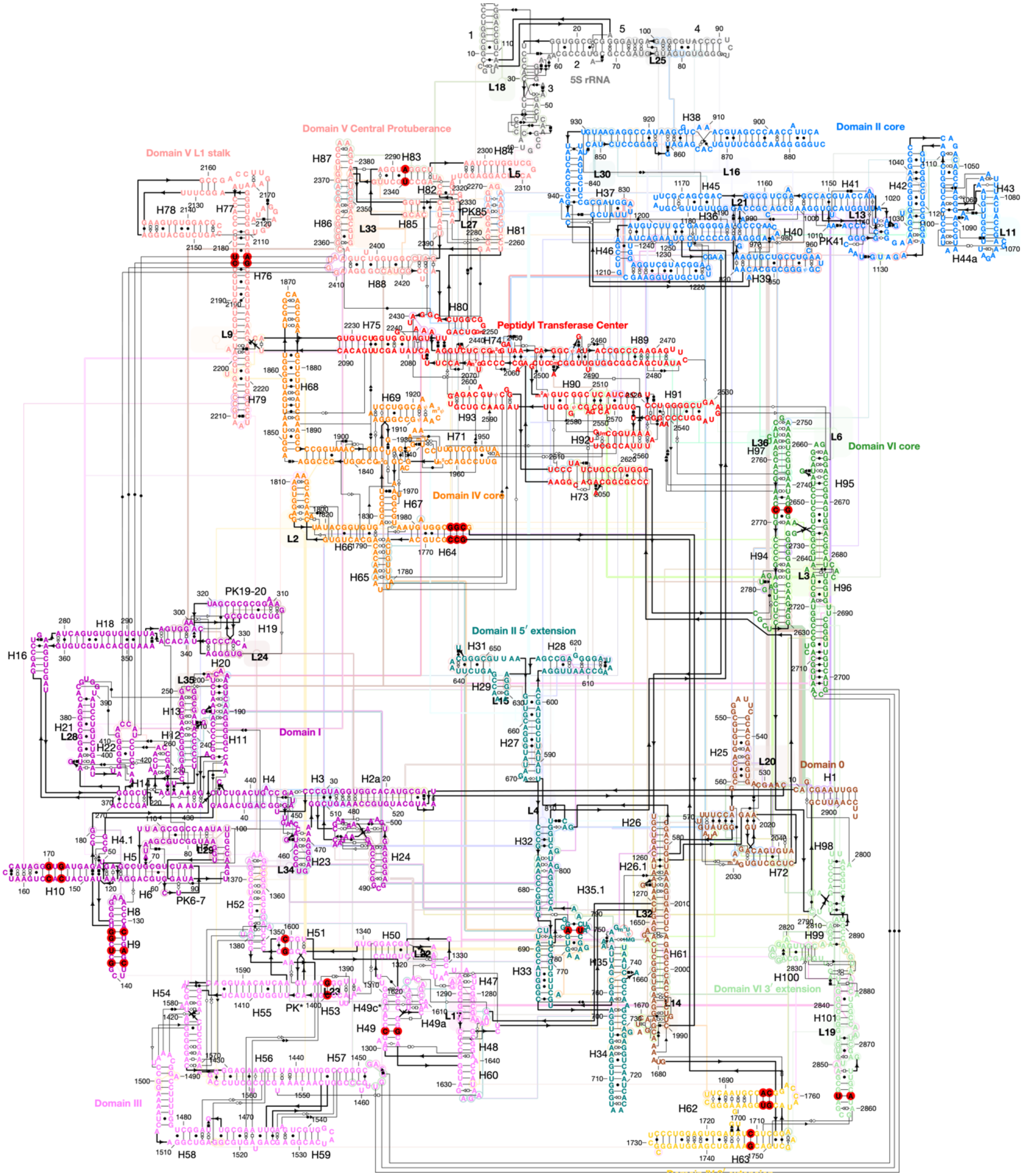

Supplementary Figure 102. Eterna participants'-designed ribosomal RNA design R2-28 prepared with RiboDraw[1].

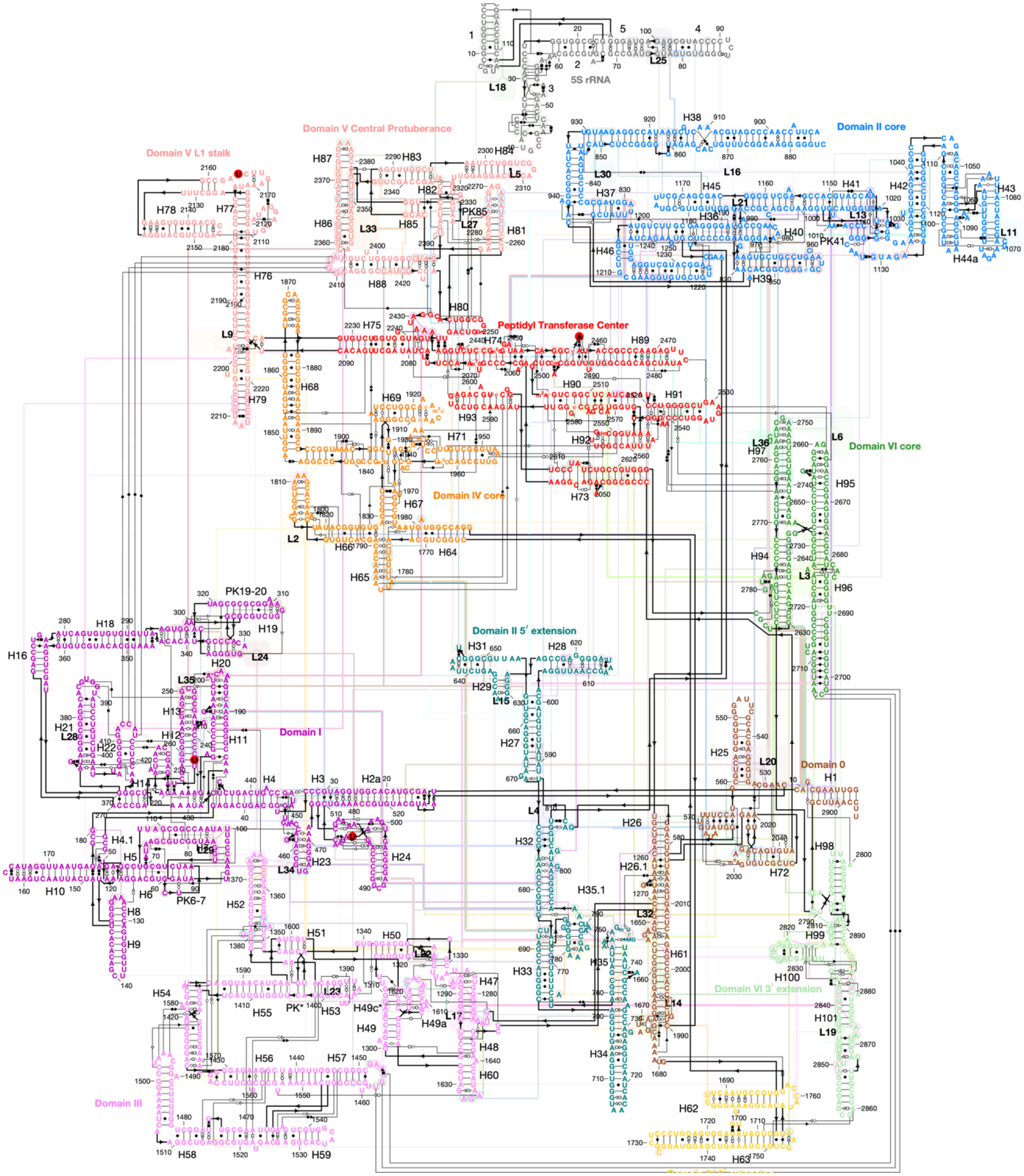

Supplementary Figure 103. Eterna participants'-designed ribosomal RNA design R2-29 prepared with RiboDraw[1].

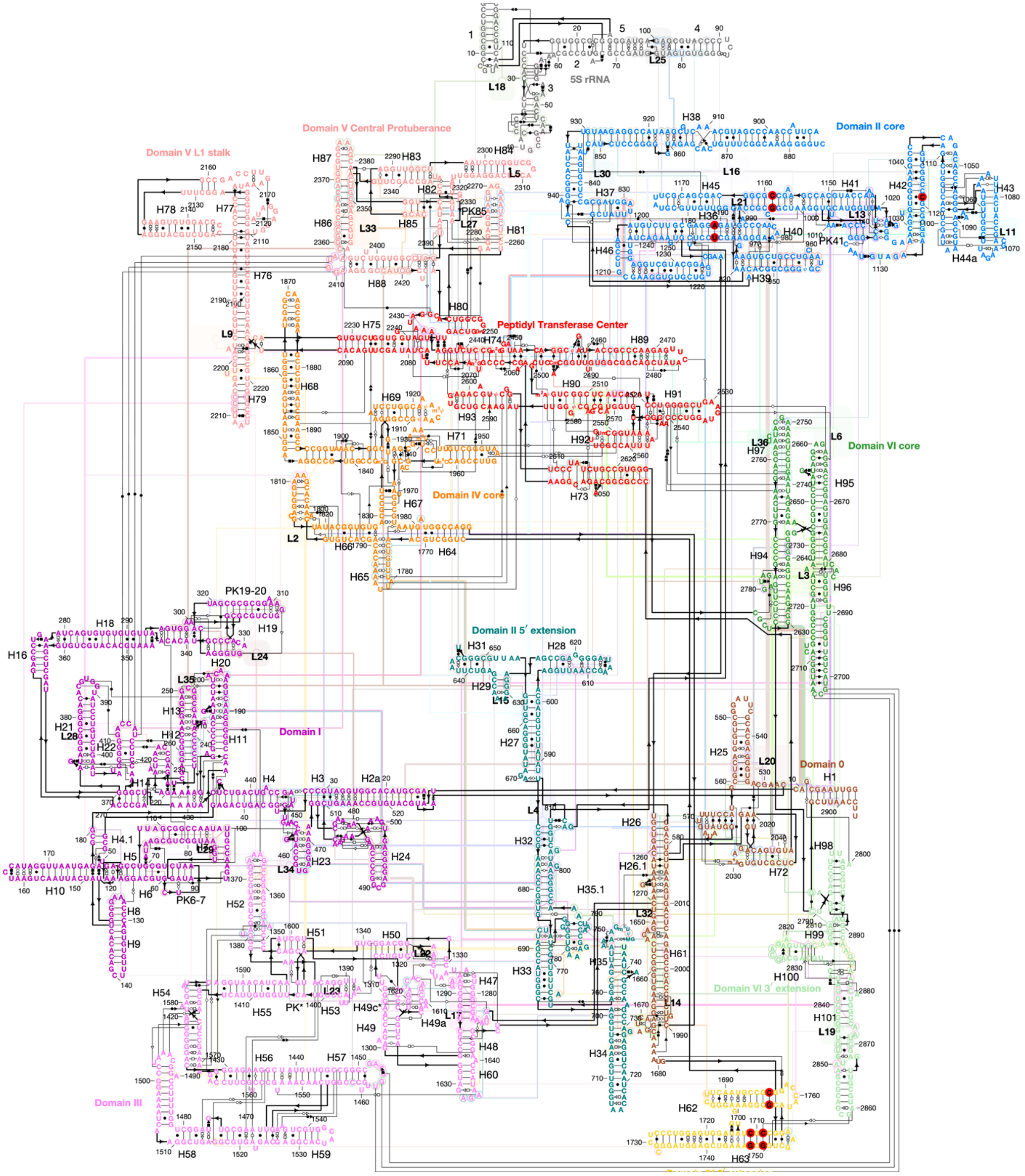

Supplementary Figure 104. Eterna participants'-designed ribosomal RNA design R2-30 prepared with RiboDraw[1].

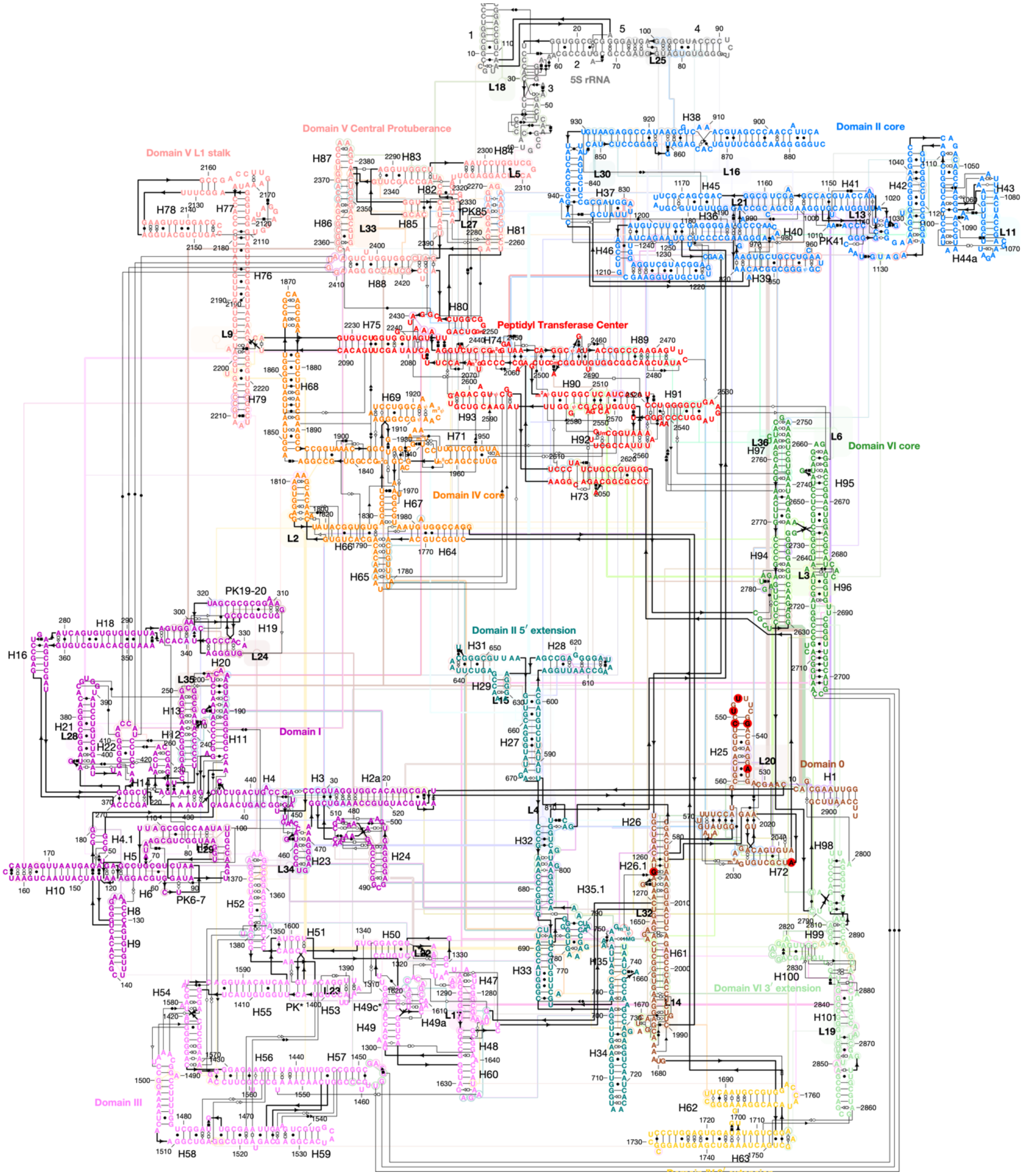

Supplementary Figure 105. Eterna participants'-designed ribosomal RNA design R2-31 prepared with RiboDraw[1].

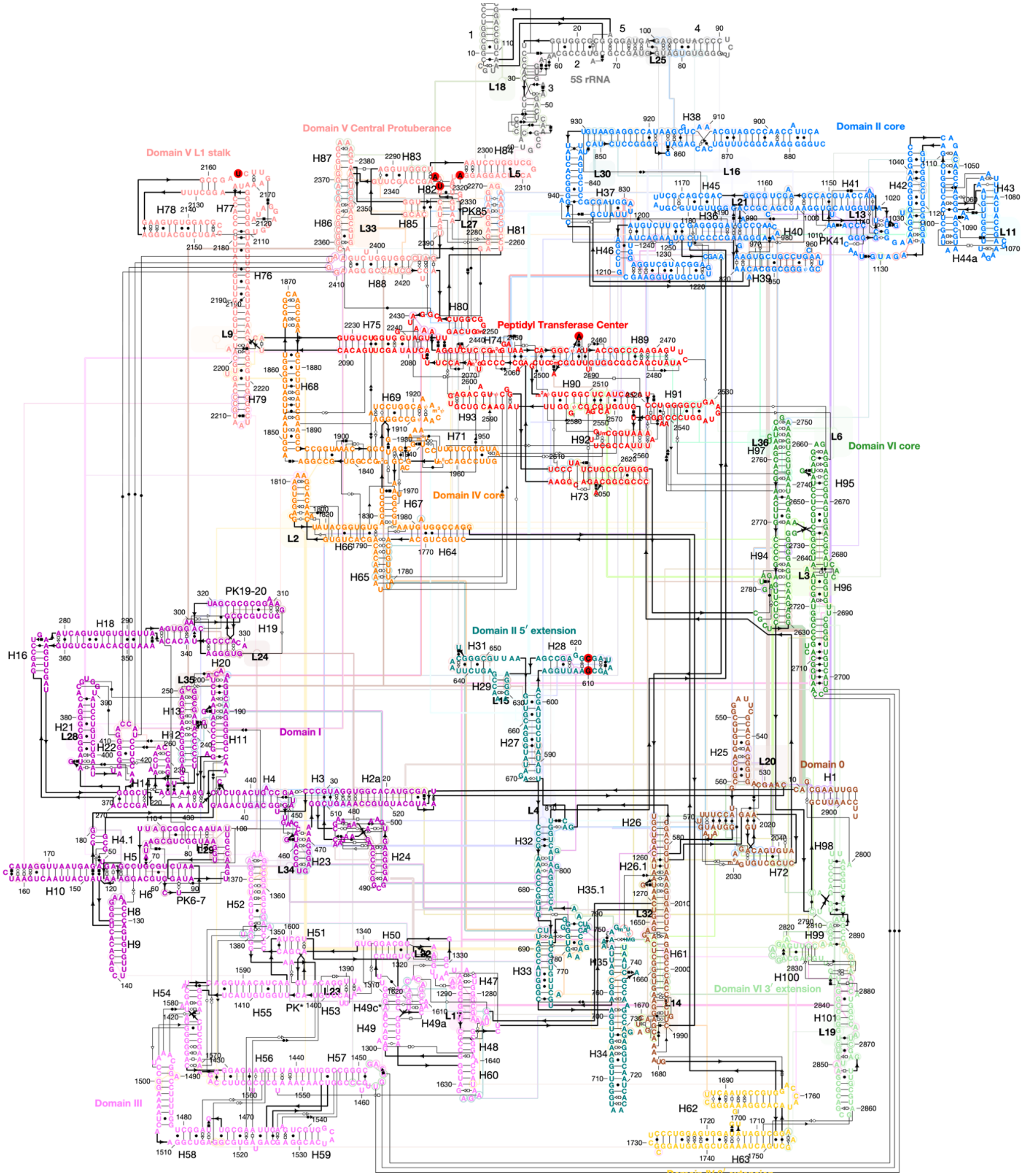

Supplementary Figure 106. Eterna participants'-designed ribosomal RNA design R2-32 prepared with RiboDraw[1].

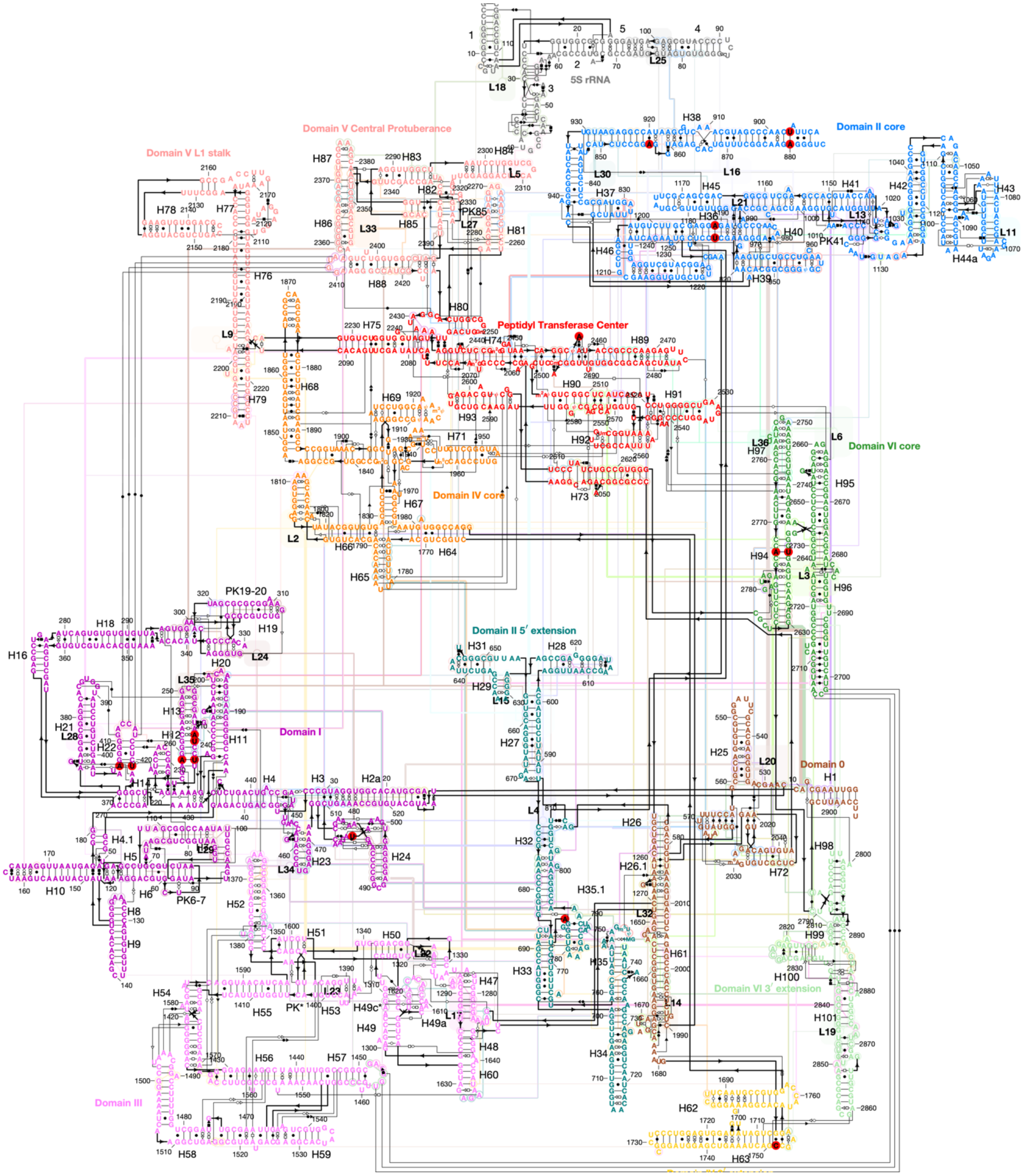

Supplementary Figure 107. Eterna participants'-designed ribosomal RNA design R2-33 prepared with RiboDraw[1].

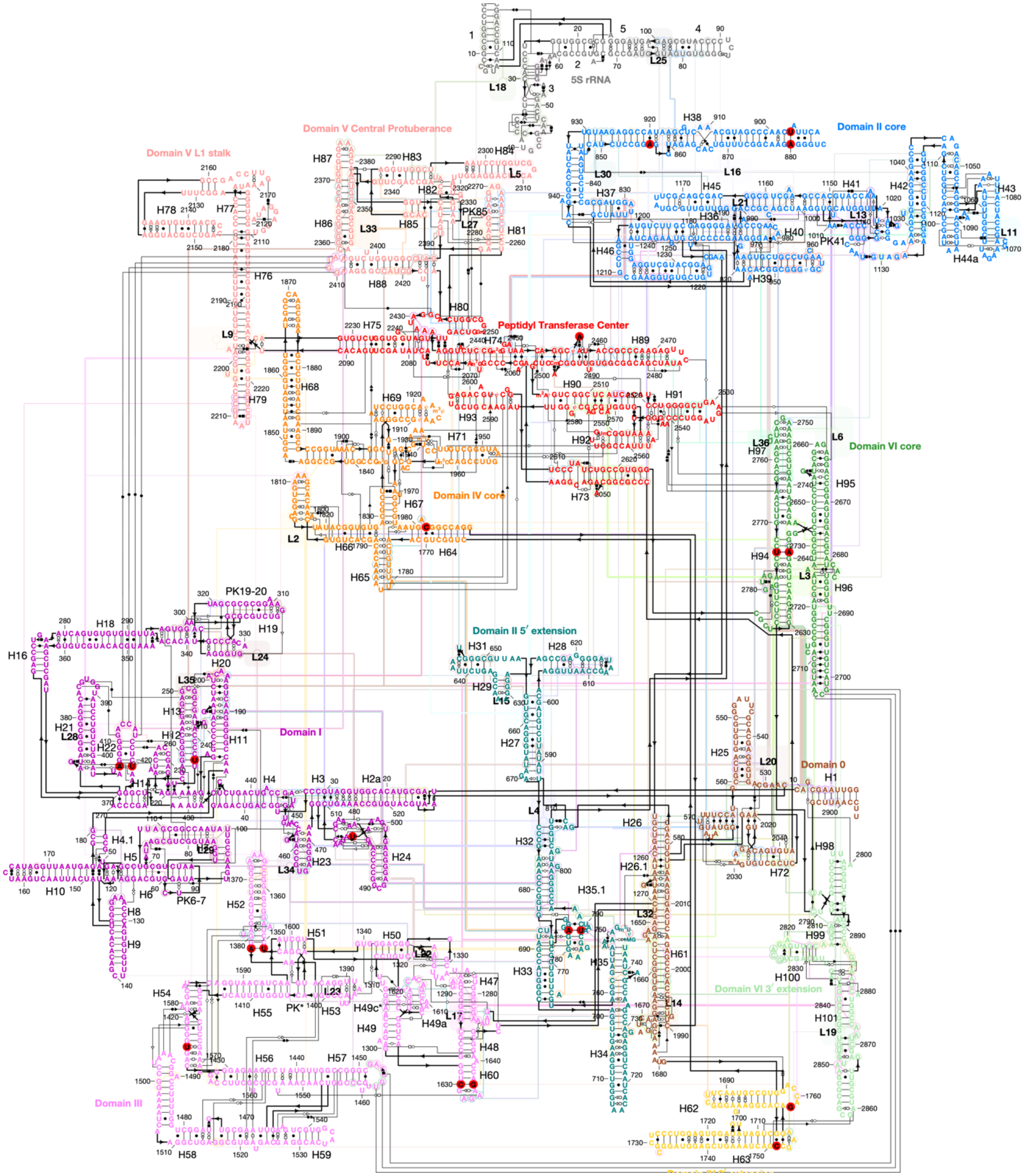

Supplementary Figure 108. Eterna participants'-designed ribosomal RNA design R2-34 prepared with RiboDraw[1].

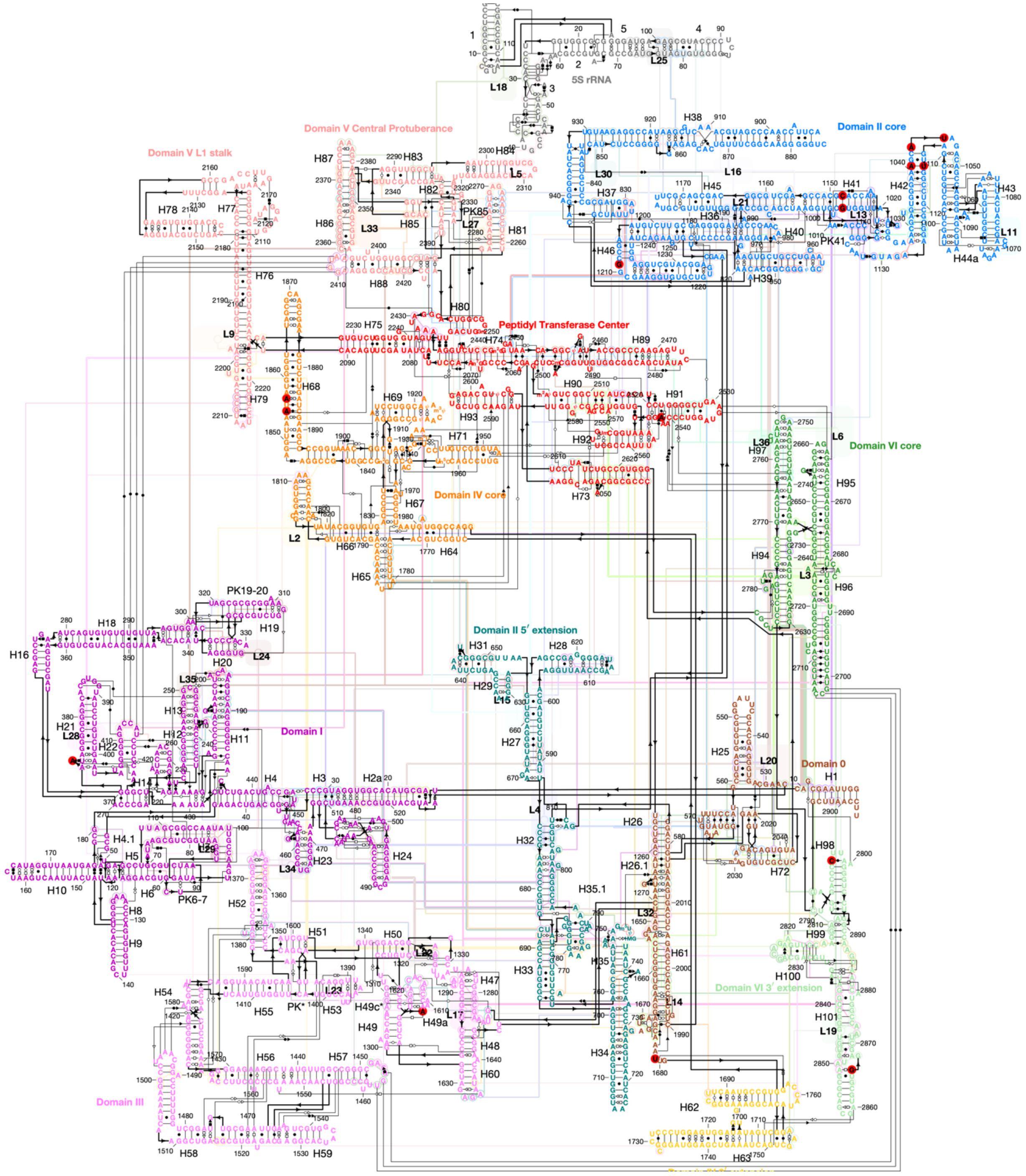

Supplementary Figure 109. Eterna participants'-designed ribosomal RNA design R2-35 prepared with RiboDraw[1].

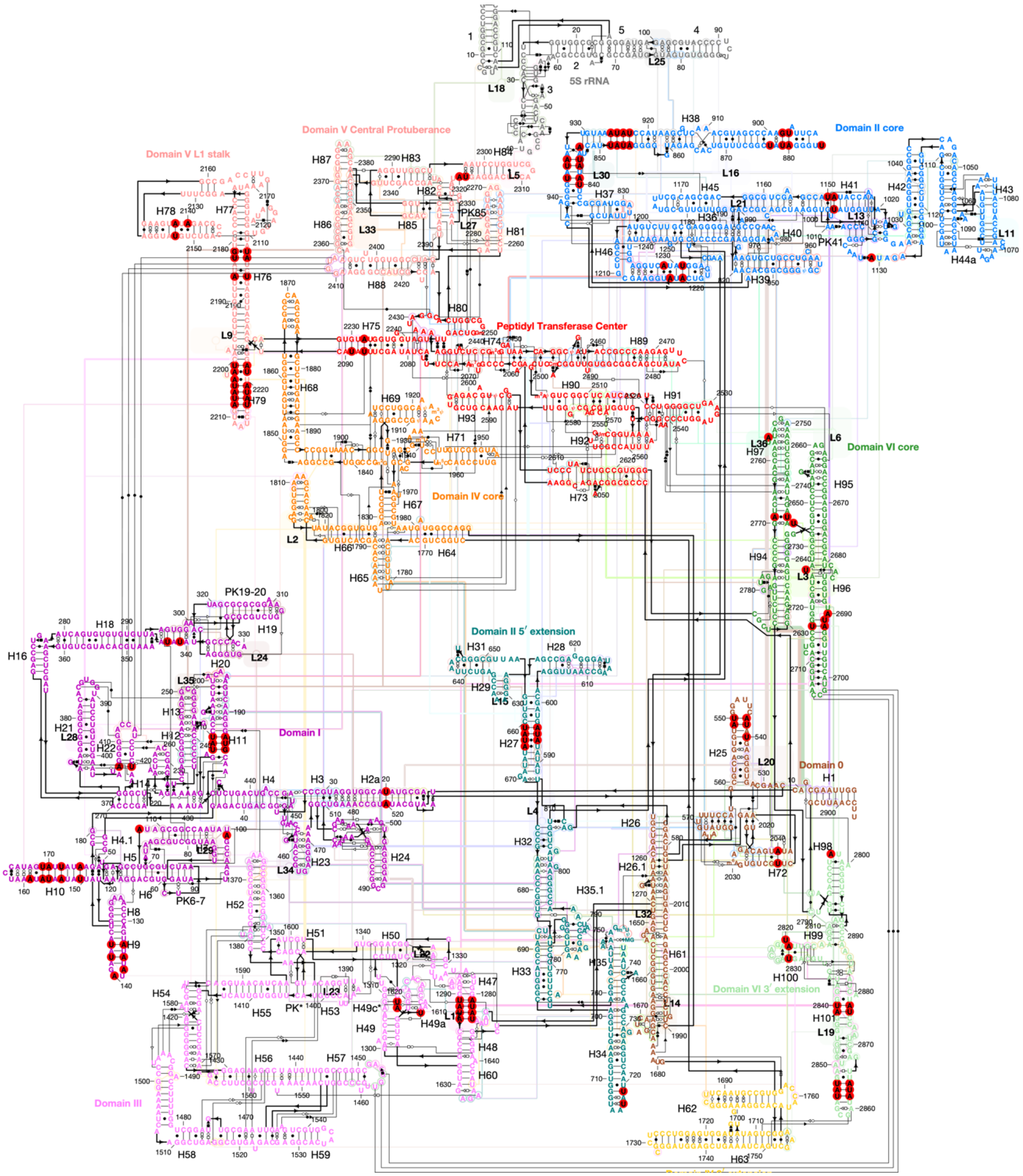

Supplementary Figure 110. Eterna participants'-designed ribosomal RNA design R2-36 prepared with RiboDraw[1].

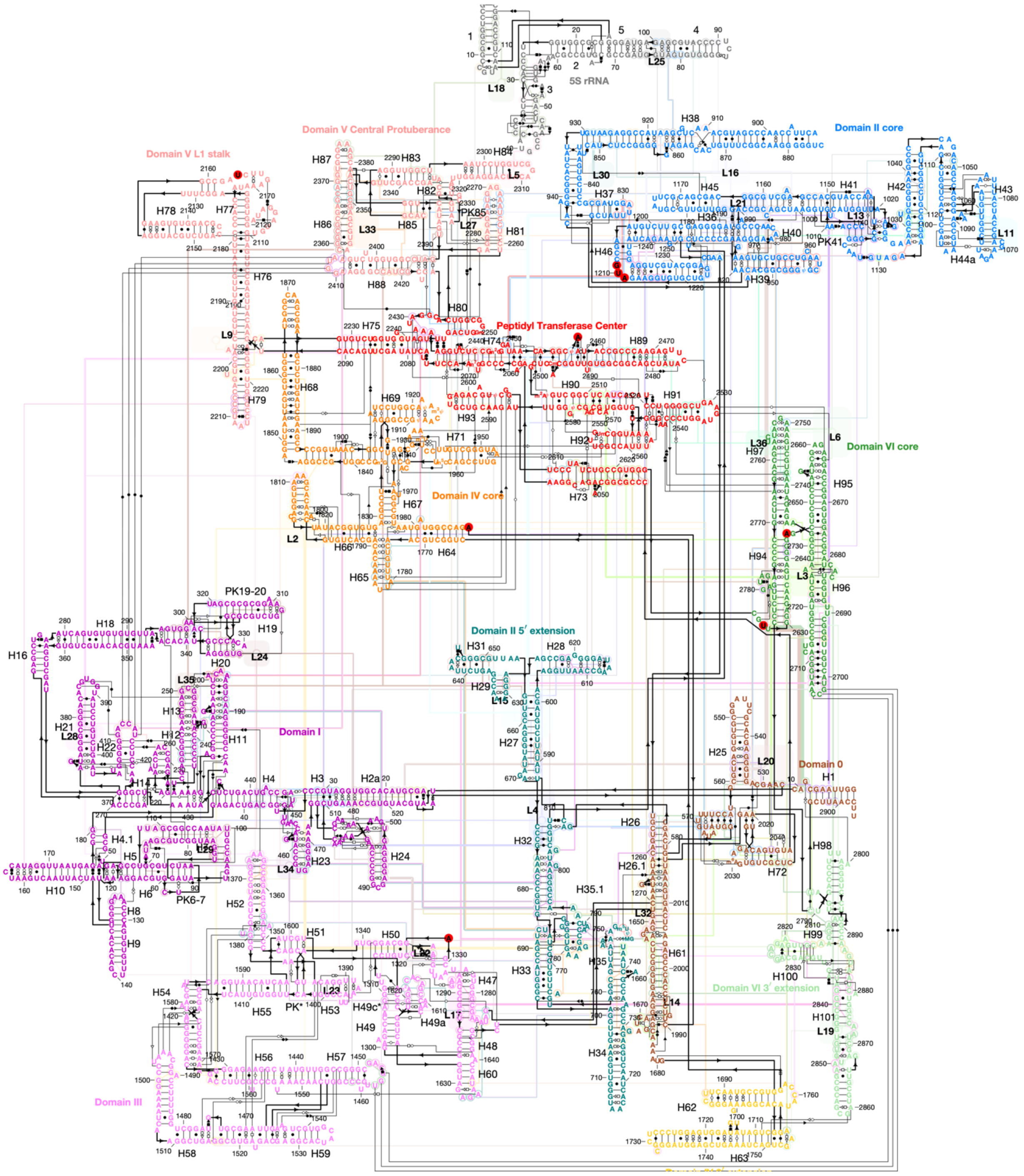

Supplementary Figure 111. Eterna participants'-designed ribosomal RNA design R2-37 prepared with RiboDraw[1].

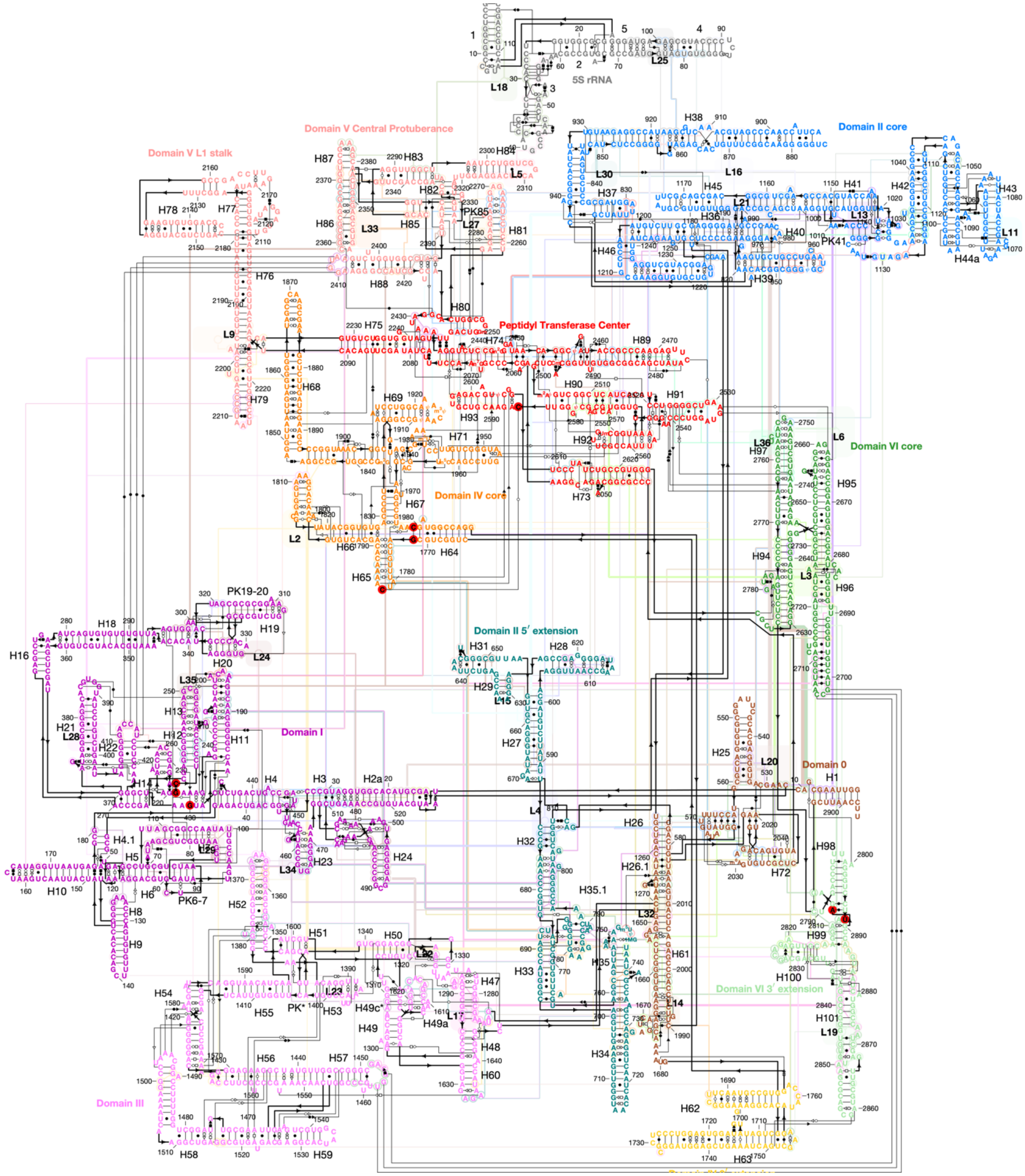

Supplementary Figure 112. Eterna participants'-designed ribosomal RNA design R2-38 prepared with RiboDraw[1].

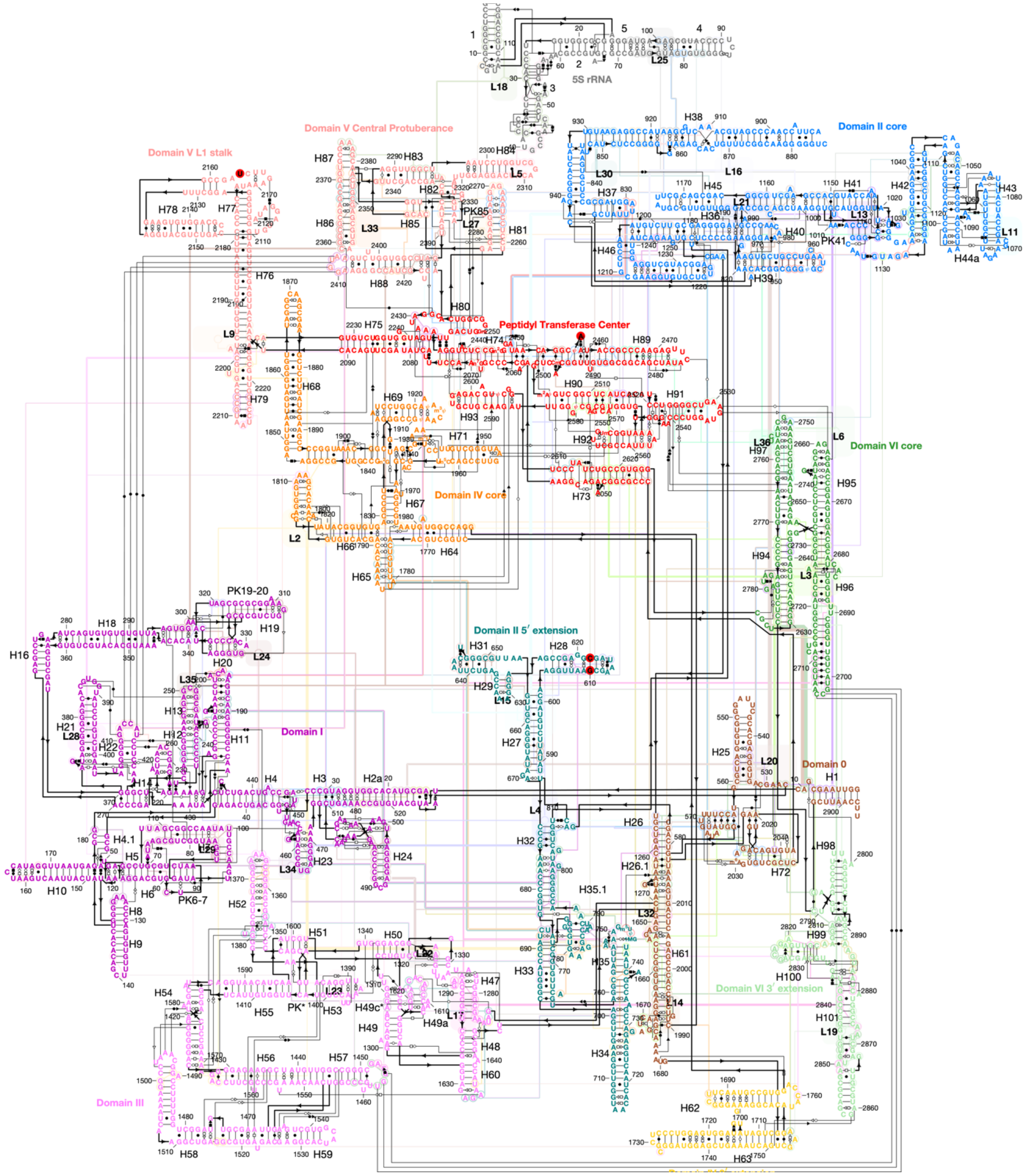

Supplementary Figure 113. Eterna participants'-designed ribosomal RNA design R2-39 prepared with RiboDraw[1].

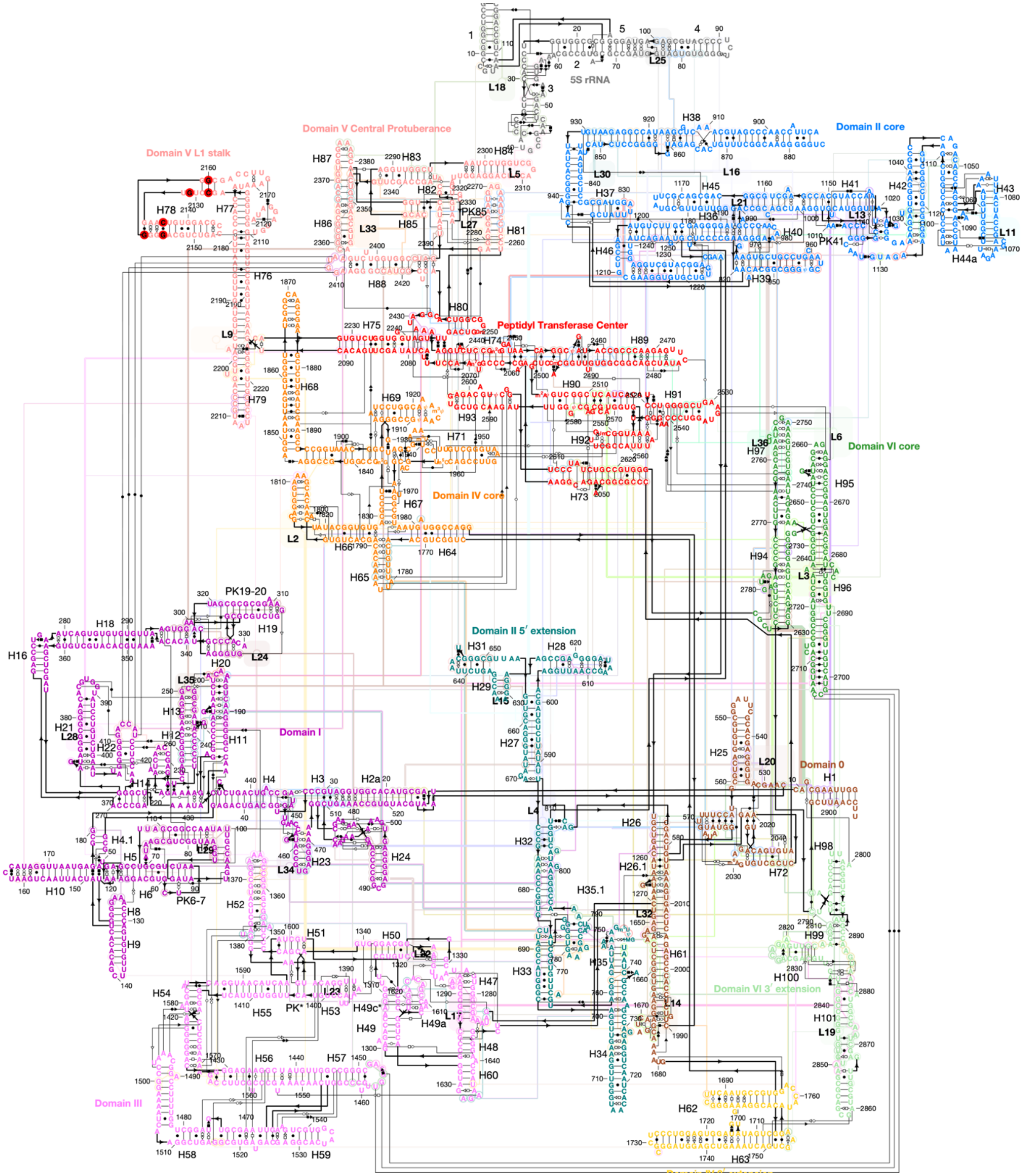

Supplementary Figure 114. Eterna participants'-designed ribosomal RNA design R2-40 prepared with RiboDraw[1].

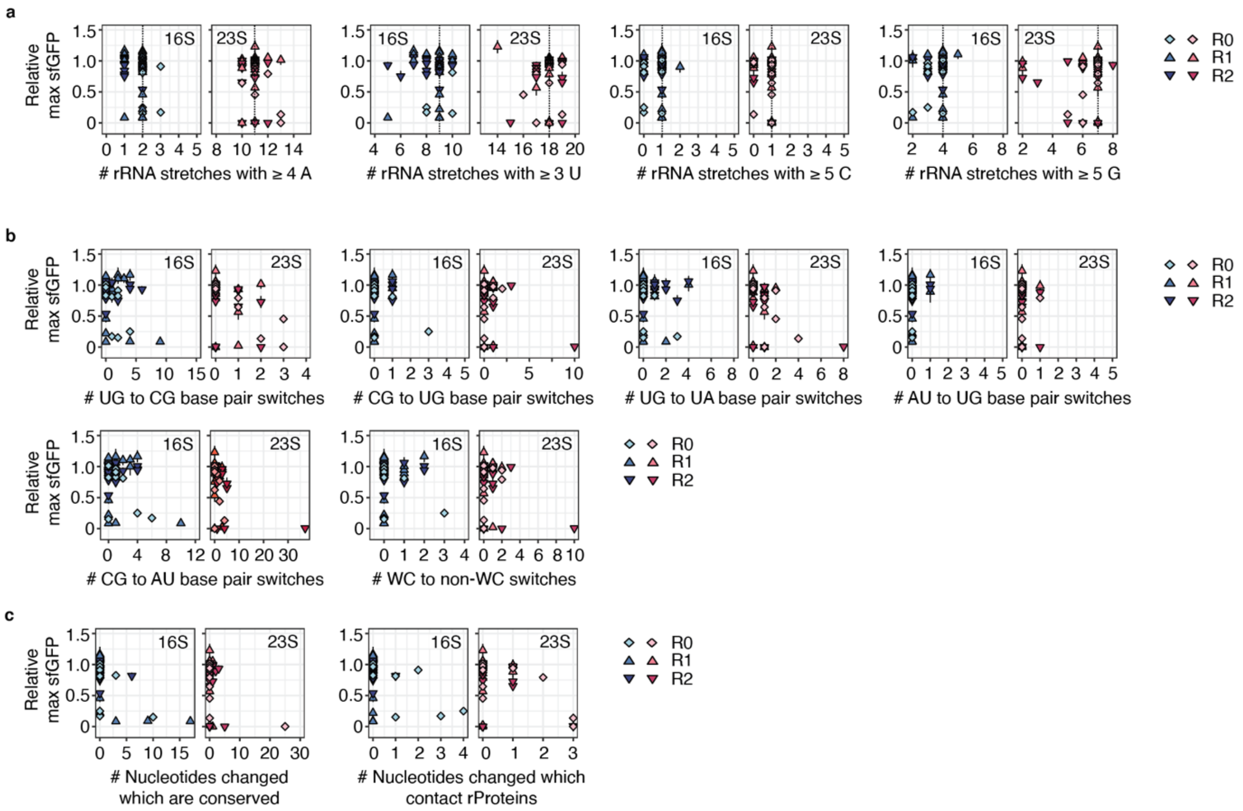

Supplementary Figure 115. Community scientists followed and combined different strategies to improve rRNA performance. Relative maximum sfGFP expression made in iSAT reactions by each design was plotted against the instances of (**a**) stretches of consecutive identical nucleotides, (**b**) altered base pairing in rRNA secondary structures, or (**c**) changes in conserved nucleotides or nucleotides which contact rProteins. Data are shown from the “pilot round” (R0) and round 1 (R1) and round 2 (R2) as mean ± s.d.; n ≥ 3. Source data are provided as a Source Data file. Dotted line in (**a**) indicates wild type value. rProteins: ribosomal proteins; WC: Watson-Crick.

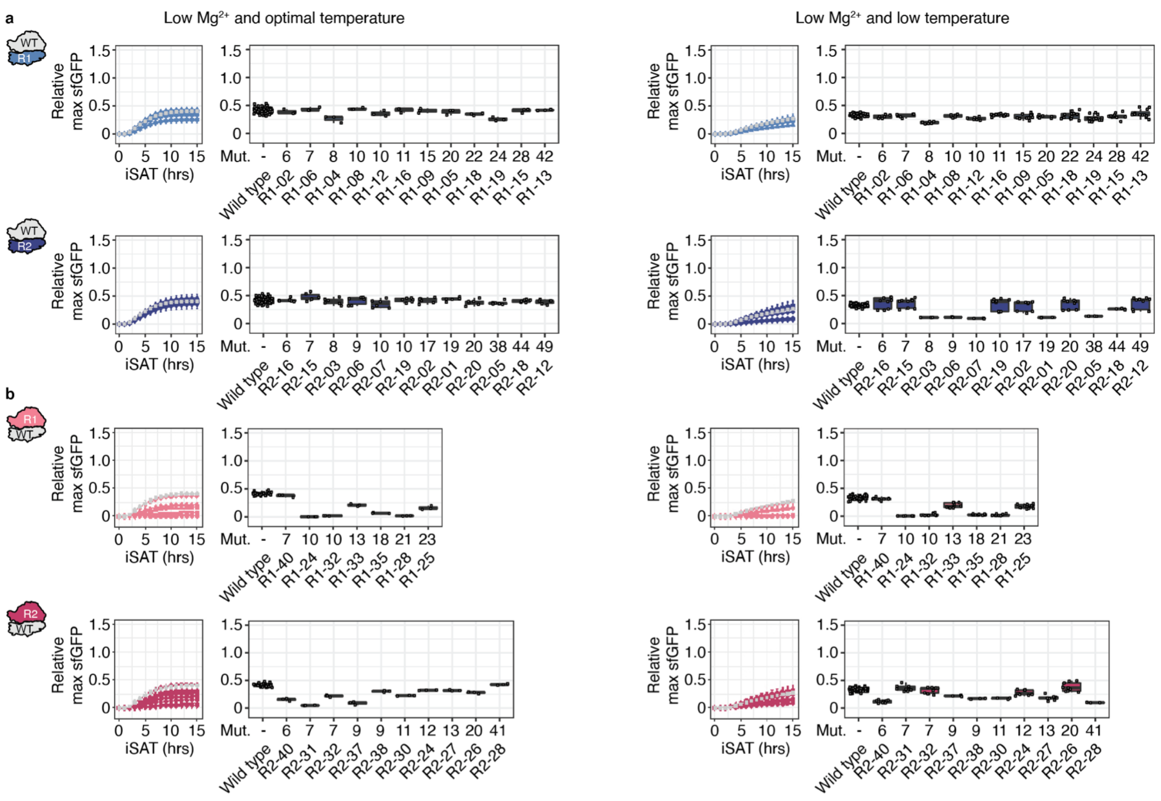

Supplementary Figure 116. iSAT kinetics and maximum yields for Eterna designed ribosomes under folding stress. sfGFP expression of (**a**) pT7-rrnB-16S R1 and R2 designs and (**b**) pT7-rrnB-23S R1 and R2 designs in iSAT at low Mg^{2+} concentration (3.75 mM) and optimal temperature (37° C) (left panels) and low Mg^{2+} concentration (3.75 mM) and low temperature (30° C) (right panels). sfGFP expression was determined in 15 hour iSAT reactions by fluorescence and normalized to max sfGFP of pT7-rrnB-WT at optimal iSAT conditions. Data are shown as mean. Error bars represent s.d.; n ≥ 3. Source data are provided as a Source Data file. These data were used to generate the heat maps in Main Figure 3. Mut: mutations; R1: round 1, R2: round 2, WT: wild type.

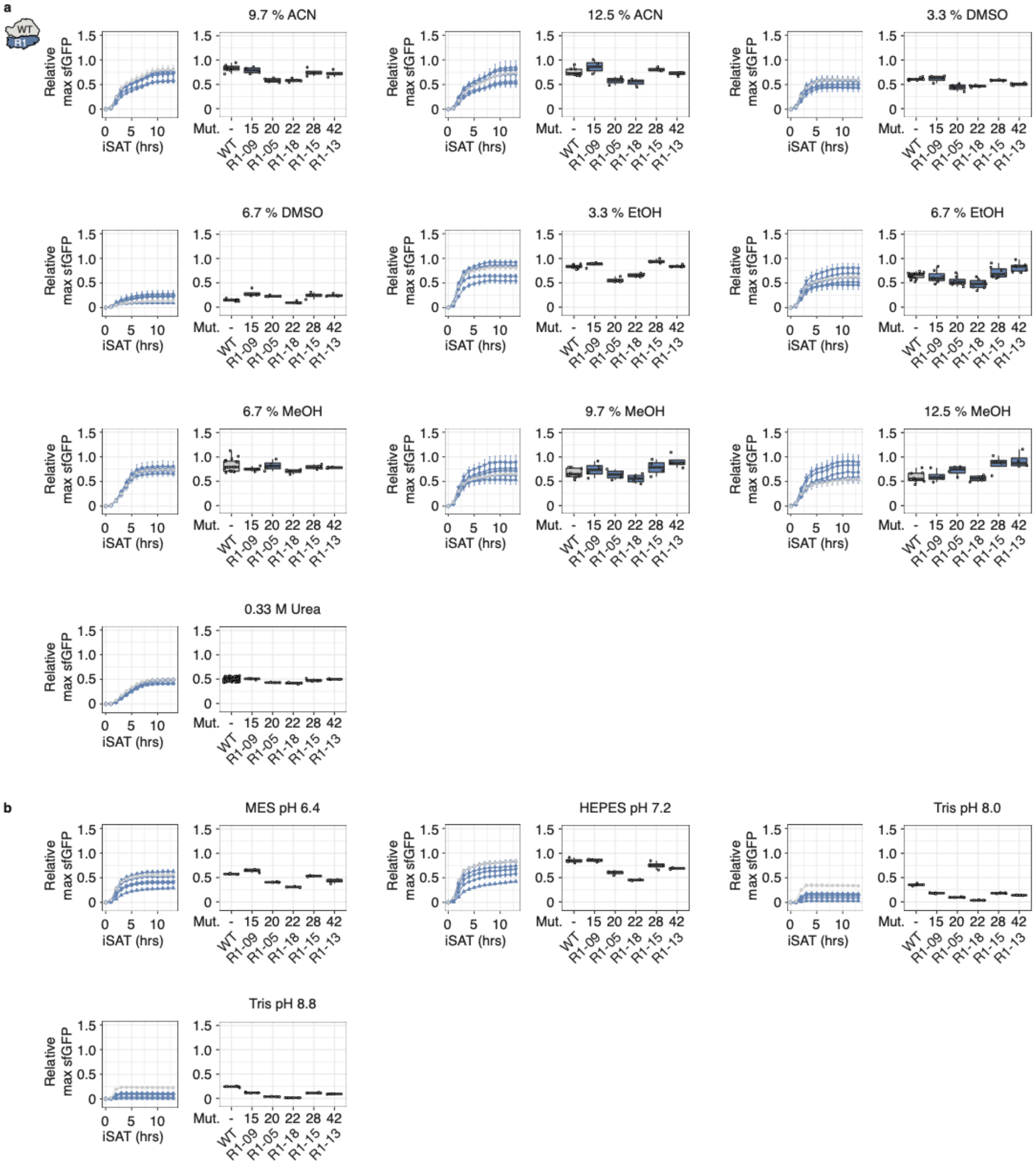

Supplementary Figure 117. Influence of solvents and pH on iSAT reactions of selected R1 16S rRNA designs. (**a**) Solvents (v/v %). (**b**) pH. Time course data are shown as mean ± s.d. Maximum sfGFP expression was determined in iSAT reactions by fluorescence and normalized to max sfGFP of pT7-rrnB-wild type at optimal iSAT conditions. Maximal sfGFP expression data are presented as boxplots. Error bars represent s.d.; n ≥ 3. Source data are provided as a Source

Data file. ACN: acetonitrile, DMSO: dimethylsulfoxide, EtOH: ethanol, MeOH: methanol, HEPES: (4-(2-hydroxyethyl)-1-piperazineethanesulfonic acid), MES: 2-(N-morpholino)ethanesulfonic acid, Tris: tris(hydroxymethyl)aminomethane, Mut: mutations, R1: round 1, WT: wild type.

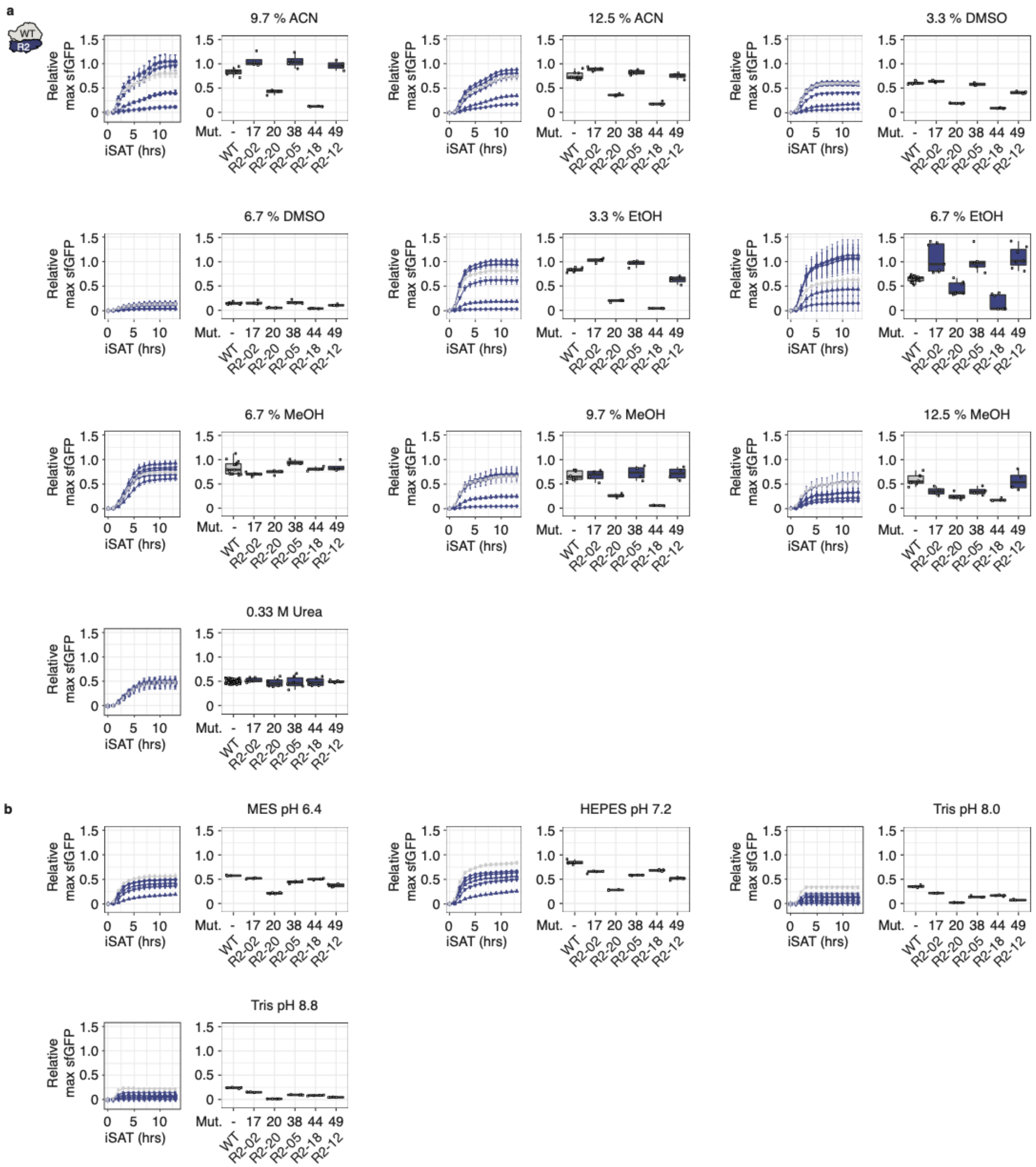

Supplementary Figure 118. Influence of solvents and pH on iSAT reactions of selected R2 16S rRNA designs. (**a**) Solvents (v/v %). (**b**) pH. Time course data are shown as mean ± s.d. Maximum sfGFP expression was determined in iSAT reactions by fluorescence and normalized to max sfGFP of pT7-rrnB-wild type at optimal iSAT conditions. Maximal sfGFP expression data

are presented as boxplots. Error bars represent s.d.; $n \geq 3$. Source data are provided as a Source Data file. ACN: acetonitrile, DMSO: dimethylsulfoxide, EtOH: ethanol, MeOH: methanol, HEPES: (4-(2-hydroxyethyl)-1-piperazineethanesulfonic acid), MES: 2-(N-morpholino)ethanesulfonic acid, Tris: tris(hydroxymethyl)aminomethane, Mut: mutations, R2: round 2, WT: wild type

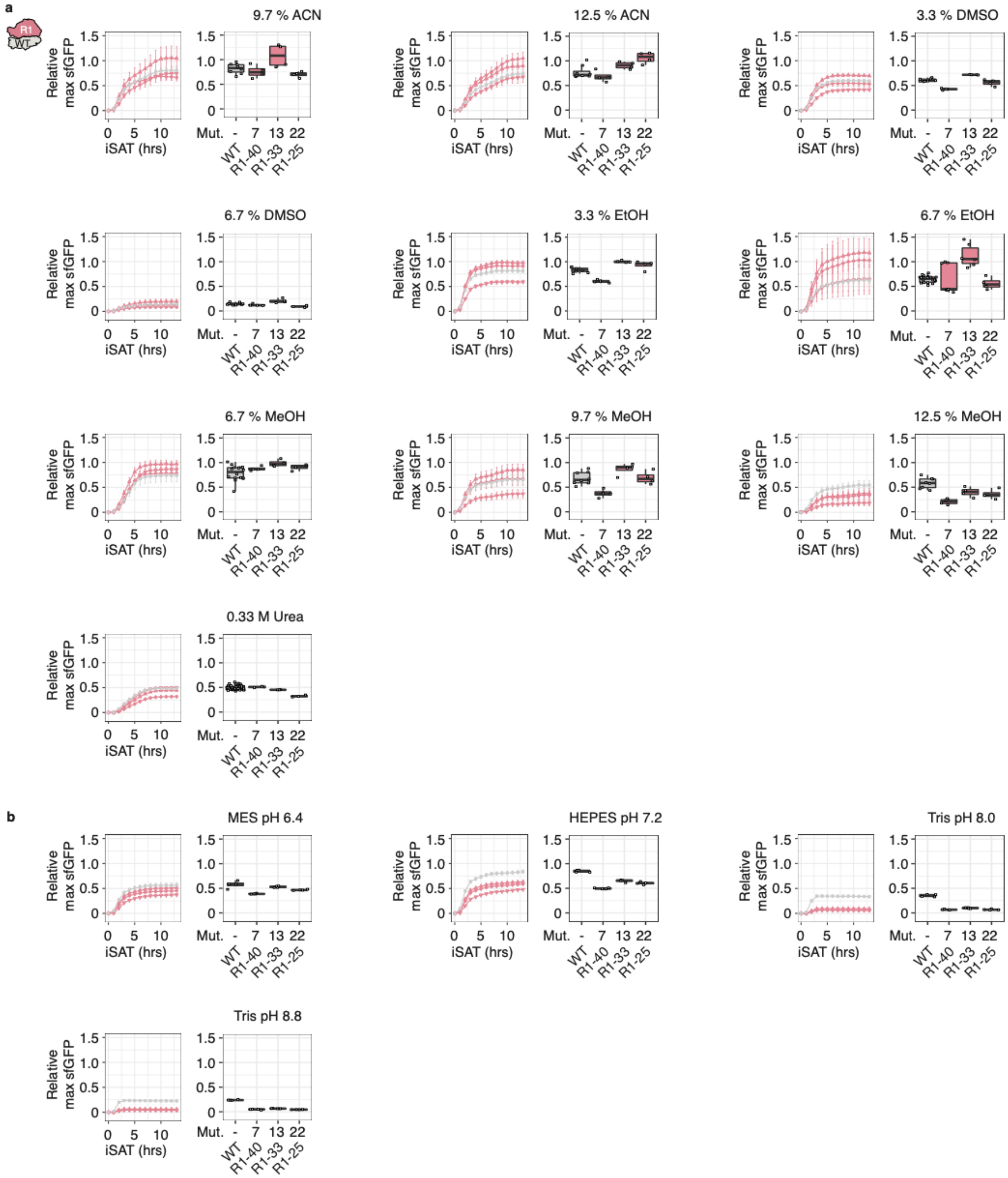

Supplementary Figure 119. Influence of solvents and pH on iSAT reactions of selected R1 23S rRNA designs. (**a**) Solvents (v/v %). (**b**) pH. Time course data are shown as mean ± s.d. Maximum sfGFP expression was determined in iSAT reactions by fluorescence and normalized to max sfGFP of pT7-rrnB-wild type at optimal iSAT conditions. Maximal sfGFP expression data are presented as boxplots. Error bars represent s.d.; n ≥ 3. Source data are provided as a Source

Data file. ACN: acetonitrile, DMSO: dimethylsulfoxide, EtOH: ethanol, MeOH: methanol, HEPES: (4-(2-hydroxyethyl)-1-piperazineethanesulfonic acid), MES: 2-(N-morpholino)ethanesulfonic acid, Tris: tris(hydroxymethyl)aminomethane, Mut: mutations, R1: round 1, WT: wild type.

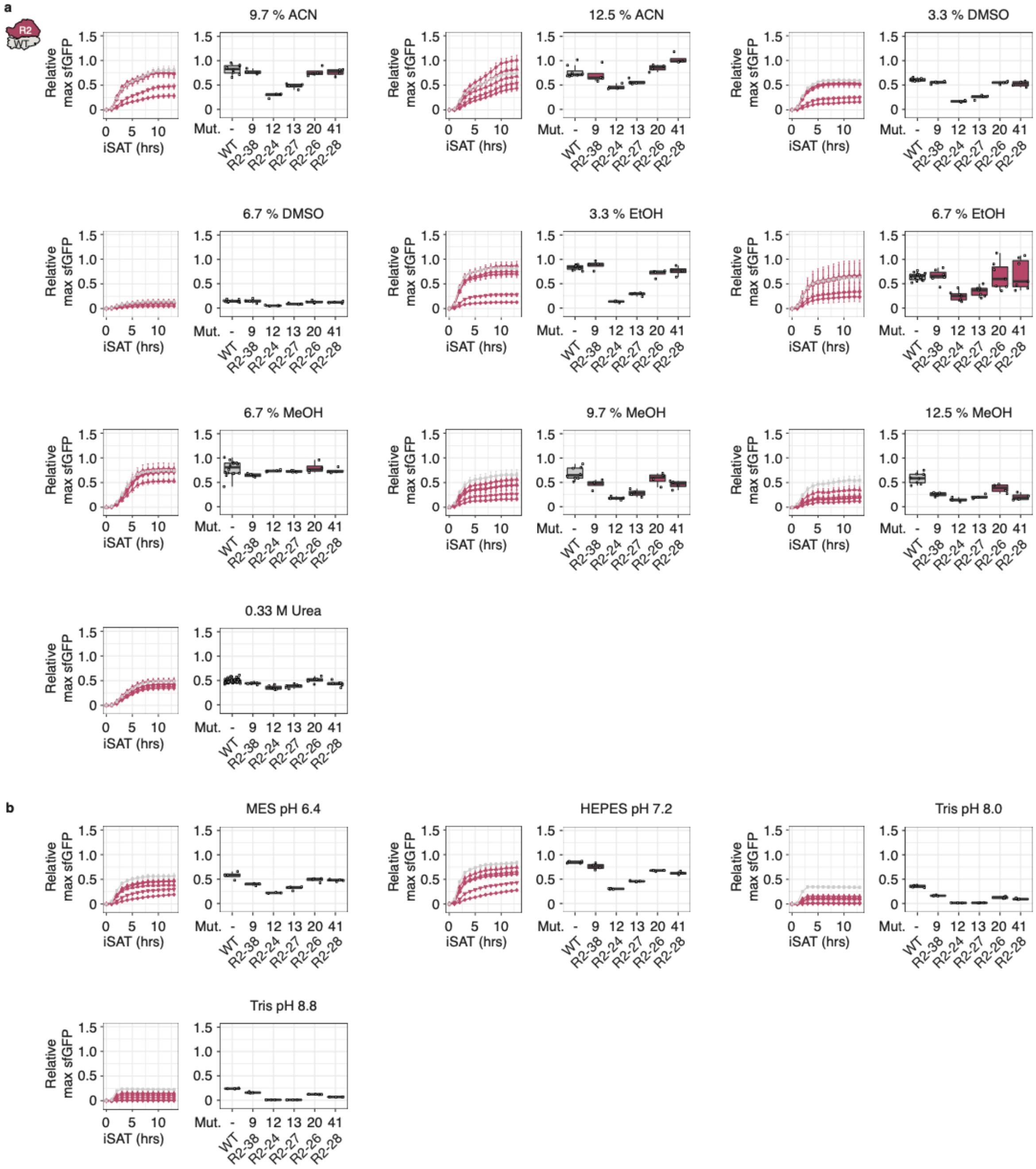

Supplementary Figure 120. Influence of solvents and pH on iSAT reactions of selected R2 23S designs. (**a**) Solvents (v/v %). (**b**) pH. Time course data are shown as mean ± sd. Maximum sfGFP expression was determined in iSAT reactions by fluorescence and normalized to max sfGFP of pT7-rrnB-wild type at optimal iSAT conditions. Maximal sfGFP expression data are presented as boxplots. Error bars represent s.d.; n ≥ 3. Source data are provided as a Source

Data file. ACN: acetonitrile, DMSO: dimethylsulfoxide, EtOH: ethanol, MeOH: methanol, HEPES: (4-(2-hydroxyethyl)-1-piperazineethanesulfonic acid), MES: 2-(N-morpholino)ethanesulfonic acid, Tris: tris(hydroxymethyl)aminomethane, Mut: mutations, R2: round 2, WT: wild type.

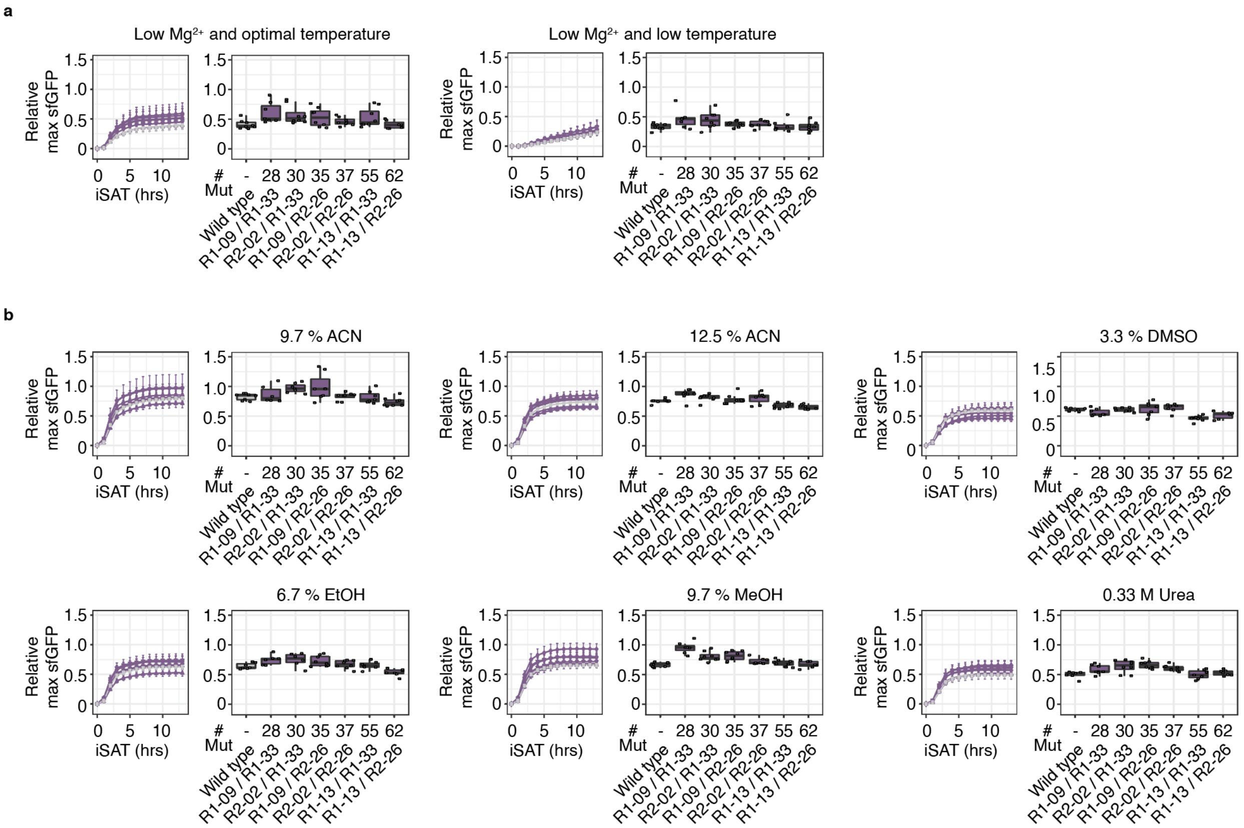

Supplementary Figure 121. Influence of *in vitro* solvent conditions on iSAT reactions of Eterna ribosomes. (**a**) Relative sfGFP expression of ribosomes with both 16S rRNA and 23S rRNA designs under iSAT conditions at low (3.75 mM)-magnesium (Mg^{2+}) and optimal or low (3.75 mM)-magnesium (Mg^{2+}) and low temperature. (**b**) Solvents (v/v %). Time course data are shown as mean ± s.d. Maximum sfGFP expression was determined in iSAT reactions by fluorescence and normalized to max sfGFP of pT7-rrnB-wild type at optimal iSAT conditions. Maximal sfGFP expression data are presented as boxplots. Error bars represent s.d.; n ≥ 3. Source data are provided as a Source Data file. ACN: acetonitrile, DMSO: dimethylsulfoxide, EtOH: ethanol, MeOH: methanol, Mut: mutations.

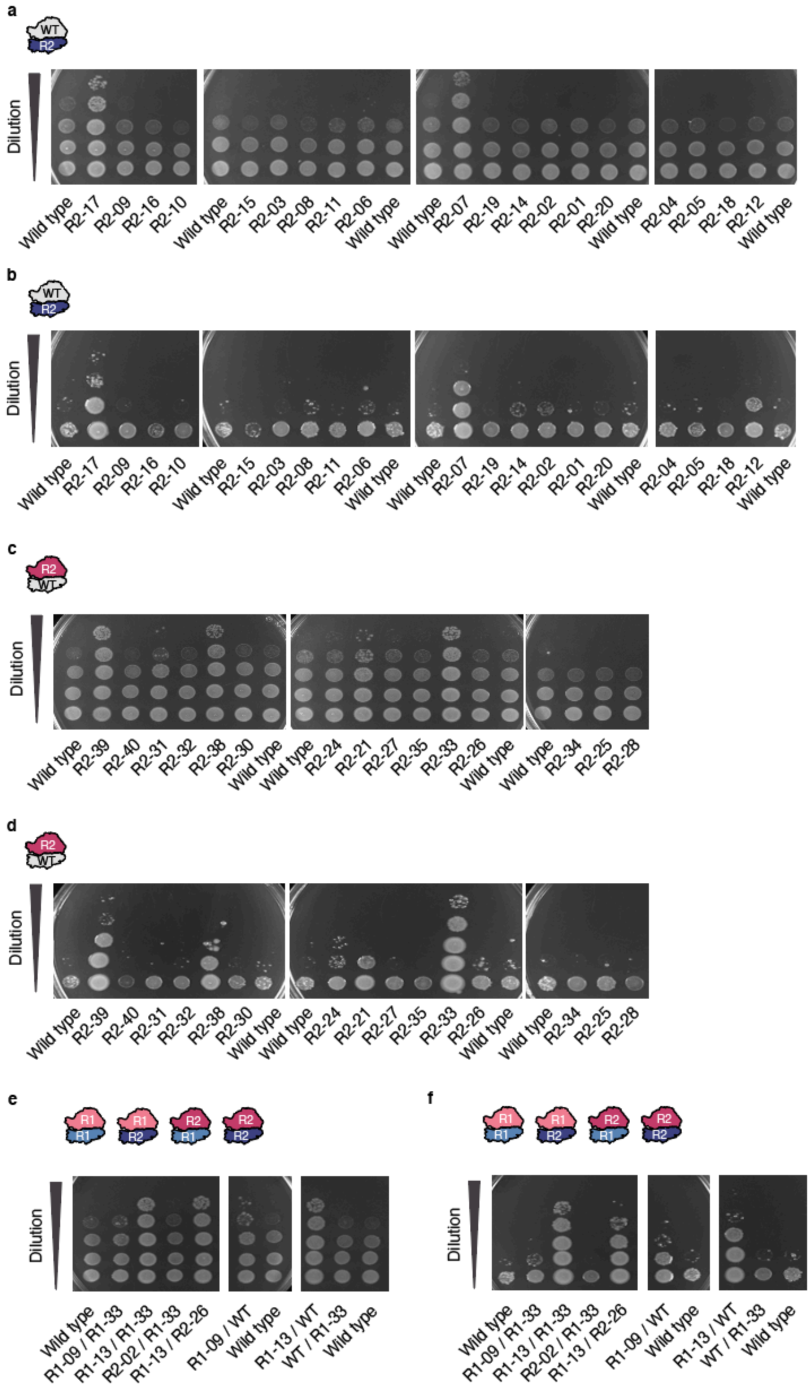

Supplementary Figure 122. R2 and combinatorial Eterna designs support life. (**a-d**) Un-cut images of spotted SQ171fg cells growing with pL-rrnB-wild type and pL-rrnB-R2 16S rRNA (**a, b**) and 23S rRNA (**c, d**) designs imaged after 24 hours at 37 °C (**a, c**) or 72 hours at 30 °C (**b, d**).

(**e-f**) Un-cut images of spotted SQ171fg cells growing with pL-rrnB-wild type and pL-rrnB-combinations imaged after 24 hours at 37 °C (**e**) or 72 hours at 30 °C (**f**). Stationary cells were diluted to an OD600 = 1, diluted stepwise 1:10, and spotted onto LB + $Carb_{100}$ plates. Data are representative of n ≥ 3. R1: round 1, R2: round 2, WT: wild type.

SUPPLEMENTARY TABLES

Supplementary Table 1. Wild type ribosome activity in iSAT in the presence of solvent concentrations.

Solvent Concentration	Concentration Unit	Solvent	Mean	Standard deviation
0	% (v/v)	Acetonitrile	1.004	0.070
0	% (v/v)	DMSO	1.004	0.070
0	% (v/v)	EtOH	1.004	0.070
0	% (v/v)	MeOH	1.004	0.070
0	% (v/v)	Urea	1.004	0.070
0.5	% (v/v)	Acetonitrile	1.033	0.021
1	% (v/v)	Acetonitrile	1.027	0.015
2.5	% (v/v)	Acetonitrile	0.997	0.014
5	% (v/v)	Acetonitrile	0.896	0.064
6.67	% (v/v)	Acetonitrile	0.889	0.111
9.38	% (v/v)	Acetonitrile	0.874	0.028
12.5	% (v/v)	Acetonitrile	0.708	0.046
0.5	% (v/v)	DMSO	0.994	0.027
1	% (v/v)	DMSO	0.933	0.027
2.5	% (v/v)	DMSO	0.779	0.028
5	% (v/v)	DMSO	0.376	0.012
6.67	% (v/v)	DMSO	0.113	0.002
9.38	% (v/v)	DMSO	0.057	0.003
12.5	% (v/v)	DMSO	0.015	0.001
0.5	% (v/v)	EtOH	0.939	0.084
1	% (v/v)	EtOH	0.994	0.007
2.5	% (v/v)	EtOH	0.914	0.038
5	% (v/v)	EtOH	0.700	0.009
6.67	% (v/v)	EtOH	0.619	0.104
9.38	% (v/v)	EtOH	0.504	0.013
12.5	% (v/v)	EtOH	0.302	0.040
0.5	% (v/v)	MeOH	0.985	0.032
1	% (v/v)	MeOH	0.929	0.076
2.5	% (v/v)	MeOH	0.971	0.051
5	% (v/v)	MeOH	0.735	0.009
6.67	% (v/v)	MeOH	0.817	0.033
9.38	% (v/v)	MeOH	0.652	0.035
12.5	% (v/v)	MeOH	0.574	0.063
0.05	M	Urea	0.983	0.058
0.1	M	Urea	0.941	0.027
0.25	M	Urea	0.703	0.014
0.5	M	Urea	0.152	0.002
0.6	M	Urea	0.029	0.002
0.94	M	Urea	0.020	0.001
1.13	M	Urea	0.017	0.000

Maximum sfGFP expression was determined in iSAT reactions by fluorescence and normalized to max sfGFP of pT7-rrnB-WT at optimal iSAT conditions. Maximal sfGFP expression data are presented as mean and s.d.; n ≥ 3.

SUPPLEMENTARY NOTES

Supplementary Notes summarizing resources and online tools that players used for designing 16S and 23S rRNAs.

- Ribosome images: For example, from the Noller lab: http://rna.ucsc.edu/rnacenter/ribosome_images.html
- Online videos on ribosomes and related topics. For example, via Khan academy and YouTube: https://www.khanacademy.org; https://www.youtube.com
- Scientific literature on ribosomes and related topics. For example, papers about RNA motifs and r-protein binding sites[1–3]
- Comparative RNA web site and project[4]: https://crw-site.chemistry.gatech.edu/FAM/
- Protein Data Bank: https://www.rcsb.org[5] and https://www.ebi.ac.uk/pdbe/[6]
- The non-coding RNA sequence database[7,8]: https://rnacentral.org
- Molecular visualization software: Chimera[9,10] and PyMOL[11]
- Multiple sequence alignment tool from the EMBL-EBI web site: https://www.ebi.ac.uk/Tools/msa/muscle/[12]

SUPPLEMENTARY METHODS

Energetic rationale for base locks in ribosome puzzle definitions

Most base locks were chosen based on intra- or inter-subunit tertiary contacts, particularly when Watson-Crick, and protein-RNA contacts that would both enormously influence the folding energetics of the rRNA under investigation but could not be represented in a secondary structure folding model. Pseudoknotted residues were also locked; folding the ribosome with a pseudoknot-aware secondary structure model would be both physically unrealistic (ribosome folding is chaperoned; the only pseudoknots likely to form in designed ribosomes are those that form in the wild type) and computationally intractable.

Some “singlet” base pairs, however, were also locked. In large, folded RNAs, tertiary folding influences the secondary structure ensemble and can render stable features that otherwise might struggle to form. “Singlet” base pairs – those that do not form part of a secondary structure stem – are not favorable on their own, since they contribute no stabilizing stacking energy, and in energy models like Vienna will typically destabilize large loops.

Because this “secondary structure” constraint could not be satisfied in the energy model, we omitted it from the target secondary structure to make the objective more achievable for players, but we locked the nucleotides to ensure that these destabilized bases were not mutated.

Plasmid sequences

pL-rrnB- wild type:

GATCTCTCACCTACCAAACAATGCCCCCCTGCAAAAAATAAATTCATATAAAAAACATACAG
ATAACCATCTGCGGTGATAAATTATCTCTGGCGGTGTTGACATAAATACCACTGGCGGTTAT
ACTGAGCACGGGTACCGGCCGCTGAGAAAAAGCGAAGCGGCACTGCTCTTTAACAATTTA
TCAGACAATCTGTGTGGGCACTCGAAGATACGGATTCTTAACGTCGCAAGACGAAAAATGA
ATACCAAGTCTCAAGAGTGAACACGTAATTCATTACGAAGTTTAATTCTTTGAGCGTCAAAC
TTTTAAATTGAAGAGTTTGATCATGGCTCAGATTGAACGCTGGCGGCAGGCCTAACACATG
CAAGTCGAACGGTAACAGGAAGAAGCTTGCTTCTTTGCTGACGAGTGGCGGACGGGTGAG
TAATGTCTGGGAAACTGCCTGATGGAGGGGGATAACTACTGGAAACGGTAGCTAATACCG
CATAACGTCGCAAGACCAAAGAGGGGGACCTTCGGGCCTCTTGCCATCGGATGTGCCCAG
ATGGGATTAGCTAGTAGGTGGGGTAACGGCTCACCTAGGCGACGATCCCTAGCTGGTCTG
AGAGGATGACCAGCCACACTGGAACTGAGACACGGTCCAGACTCCTACGGGAGGCAGCA
GTGGGGAATATTGCACAATGGGCGCAAGCCTGATGCAGCCATGCCGCGTGTATGAAGAAG
GCCTTCGGGTTGTAAAGTACTTTCAGCGGGGAGGAAGGGAGTAAAGTTAATACCTTTGCTC
ATTGACGTTACCCGCAGAAGAAGCACCGGCTAACTCCGTGCCAGCAGCCGCGGTAATACG
GAGGGTGCAAGCGTTAATCGGAATTACTGGGCGTAAAGCGCACGCAGGCGGTTTGTTAAG
TCAGATGTGAAATCCCCGGGCTCAACCTGGGAACTGCATCTGATACTGGCAAGCTTGAGT
CTCGTAGAGGGGGGTAGAATTCCAGGTGTAGCGGTGAAATGCGTAGAGATCTGGAGGAAT
ACCGGTGGCGAAGGCGGCCCCCTGGACGAAGACTGACGCTCAGGTGCGAAAGCGTGGG
GAGCAAACAGGATTAGATACCCTGGTAGTCCACGCCGTAAACGATGTCGACTTGGAGGTT
GTGCCCTTGAGGCGTGGCTTCCGGAGCTAACGCGTTAAGTCGACCGCCTGGGGAGTACG
GCCGCAAGGTTAAAACTCAAATGAATTGACGGGGGCCCGCACAAGCGGTGGAGCATGTG
GTTTAATTCGATGCAACGCGAAGAACCTTACCTGGTCTTGACATCCACGGAAGTTTTCAGA

GATGAGAATGTGCCTTCGGGAACCGTGAGACAGGTGCTGCATGGCTGTCGTCAGCTCGTG
TTGTGAAATGTTGGGTTAAGTCCCGCAACGAGCGCAACCCTTATCCTTTGTTGCCAGCGGT
CCGGCCGGGAACTCAAAGGAGACTGCCAGTGATAAACTGGAGGAAGGTGGGGATGACGT
CAAGTCATCATGGCCCTTACGACCAGGGCTACACACGTGCTACAATGGCGCATACAAAGA
GAAGCGACCTCGCGAGAGCAAGCGGACCTCATAAAGTGCGTCGTAGTCCGGATTGGAGTC
TGCAACTCGACTCCATGAAGTCGGAATCGCTAGTAATCGTGGATCAGAATGCCACGGTGAA
TACGTTCCCGGGCCTTGTACACACCGCCCGTCACACCATGGGAGTGGGTTGCAAAAGAAG
TAGGTAGCTTAACCTTCGGGAGGGCGCTTACCACTTTGTGATTCATGACTGGGGTGAAGTC
GTAACAAGGTAACCGTAGGGGAACCTGCGGTTGGATCACCTCCTTACCTTAAAGAAGCGTA
CTTTGTAGTGCTCACACAGATTGTCTGATAGAAAGTGAAAAGCAAGGCGTTTACGCGTTGG
GAGTGAGGCTGAAGAGAATAAGGCCGTTCGCTTTCTATTAATGAAAGCTCACCCTACACGA
AAATATCACGCAACGCGTGATAAGCAATTTTCGTGTCCCCTTCGTCTAGAGGCCCAGGACA
CCGCCCTTTCACGGCGGTAACAGGGGTTCGAATCCCCTAGGGGACGCCACTTGCTGGTTT
GTGAGTGAAAGTCGCCGACCTTAATATCTCAAAACTCATCTTCGGGTGATGTTTGAGATATT
TGCTCTTTAAAAATCTGGATCAAGCTGAAAATTGAAACACTGAACAACGAGAGTTGTTCGTG
AGTCTCTCAAATTTTCGCAACACGATGATGAATCGAAAGAAACATCTTCGGGTTGTGAGGTT
AAGCGACTAAGCGTACACGGTGGATGCCCTGGCAGTCAGAGGCGATGAAGGACGTGCTA
ATCTGCGATAAGCGTCGGTAAGGTGATATGAACCGTTATAACCGGCGATTTCCGAATGGG
GAAACCCAGTGTGTTTCGACACACTATCATTAACTGAATCCATAGGTTAATGAGGCGAACC
GGGGGAACTGAAACATCTAAGTACCCCGAGGAAAAGAAATCAACCGAGATTCCCCCAGTA
GCGGCGAGCGAACGGGGAGCAGCCCAGAGCCTGAATCAGTGTGTGTGTTAGTGGAAGCG
TCTGGAAAGGCGCGCGATACAGGGTGACAGCCCCGTACACAAAAATGCACATGCTGTGAG
CTCGATGAGTAGGGCGGGACACGTGGTATCCTGTCTGAATATGGGGGGACCATCCTCCAA
GGCTAAATACTCCTGACTGACCGATAGTGAACCAGTACCGTGAGGGAAAGGCGAAAAGAA
CCCCGGCGAGGGGAGTGAAAAAGAACCTGAAACCGTGTACGTACAAGCAGTGGGAGCAC
GCTTAGGCGTGTGACTGCGTACCTTTTGTATAATGGGTCAGCGACTTATATTCTGTAGCAA

GGTTAACCGAATAGGGGAGCCGAAGGGAAACCGAGTCTTAACTGGGCGTTAAGTTGCAGG
GTATAGACCCGAAACCCGGTGATCTAGCCATGGGCAGGTTGAAGGTTGGGTAACACTAAC
TGGAGGACCGAACCGACTAATGTTGAAAAATTAGCGGATGACTTGTGGCTGGGGGTGAAA
GGCCAATCAAACCGGGAGATAGCTGGTTCTCCCCGAAAGCTATTTAGGTAGCGCCTCGTG
AATTCATCTCCGGGGGTAGAGCACTGTTTCGGCAAGGGGGTCATCCCGACTTACCAACCC
GATGCAAACTGCGAATACCGGAGAATGTTATCACGGGAGACACACGGCGGGTGCTAACGT
CCGTCGTGAAGAGGGAAACAACCCAGACCGCCAGCTAAGGTCCCAAAGTCATGGTTAAGT
GGGAAACGATGTGGGAAGGCCCAGACAGCCAGGATGTTGGCTTAGAAGCAGCCATCATTT
AAAGAAAGCGTAATAGCTCACTGGTCGAGTCGGCCTGCGCGGAAGATGTAACGGGGCTAA
ACCATGCACCGAAGCTGCGGCAGCGACGCTTATGCGTTGTTGGGTAGGGGAGCGTTCTGT
AAGCCTGCGAAGGTGTGCTGTGAGGCATGCTGGAGGTATCAGAAGTGCGAATGCTGACAT
AAGTAACGATAAAGCGGGTGAAAAGCCCGCTCGCCGGAAGACCAAGGGTTCCTGTCCAAC
GTTAATCGGGGCAGGGTGAGTCGACCCCTAAGGCGAGGCCGAAAGGCGTAGTCGATGGG
AAACAGGTTAATATTCCTGTACTTGGTGTTACTGCGAAGGGGGGACGGAGAAGGCTATGTT
GGCCGGGCGACGGTTGTCCCGGTTTAAGCGTGTAGGCTGGTTTTCCAGGCAAATCCGGAA
AATCAAGGCTGAGGCGTGATGACGAGGCACTACGGTGCTGAAGCAACAAATGCCCTGCTT
CCAGGAAAAGCCTCTAAGCATCAGGTAACATCAAATCGTACCCCAAACCGACACAGGTGGT
CAGGTAGAGAATACCAAGGCGCTTGAGAGAACTCGGGTGAAGGAACTAGGCAAAATGGTG
CCGTAACTTCGGGAGAAGGCACGCTGATATGTAGGTGAGGTCCCTCGCGGATGGAGCTGA
AATCAGTCGAAGATACCAGCTGGCTGCAACTGTTTATTAAAAACACAGCACTGTGCAAACA
CGAAAGTGGACGTATACGGTGTGACGCCTGCCCGGTGCCGGAAGGTTAATTGATGGGGTT
AGCGCAAGCGAAGCTCTTGATCGAAGCCCCGGTAAACGGCGGCCGTAACTATAACGGTCC
TAAGGTAGCGAAATTCCTTGTCGGGTAAGTTCCGACCTGCACGAATGGCGTAATGATGGC
CAGGCTGTCTCCACCCGAGACTCAGTGAAATTGAACTCGCTGTGAAGATGCAGTGTACCC
GCGGCAAGACGGAAAGACCCCGTGAACCTTTACTATAGCTTGACACTGAACATTGAGCCTT
GATGTGTAGGATAGGTGGGAGGCTTTGAAGTGTGGACGCCAGTCTGCATGGAGCCGACCT

TGAAATACCACCCTTTAATGTTTGATGTTCTAACGTTGACCCGTAATCCGGGTTGCGGACA
GTGTCTGGTGGGTAGTTTGACTGGGGCGGTCTCCTCCTAAAGAGTAACGGAGGAGCACGA
AGGTTGGCTAATCCTGGTCGGACATCAGGAGGTTAGTGCAATGGCATAAGCCAGCTTGAC
TGCGAGCGTGACGGCGCGAGCAGGTGCGAAAGCAGGTCATAGTGATCCGGTGGTTCTGA
ATGGAAGGGCCATCGCTCAACGGATAAAAGGTACTCCGGGGATAACAGGCTGATACCGCC
CAAGAGTTCATATCGACGGCGGTGTTTGGCACCTCGATGTCGGCTCATCACATCCTGGGG
CTGAAGTAGGTCCCAAGGGTATGGCTGTTCGCCATTTAAAGTGGTACGCGAGCTGGGTTT
AGAACGTCGTGAGACAGTTCGGTCCCTATCTGCCGTGGGCGCTGGAGAACTGAGGGGGG
CTGCTCCTAGTACGAGAGGACCGGAGTGGACGCATCACTGGTGTTCGGGTTGTCATGCCA
ATGGCACTGCCCGGTAGCTAAATGCGGAAGAGATAAGTGCTGAAAGCATCTAAGCACGAA
ACTTGCCCCGAGATGAGTTCTCCCTGACCCTTTAAGGGTCCTGAAGGAACGTTGAAGACG
ACGACGTTGATAGGCCGGGTGTGTAAGCGCAGCGATGCGTTGAGCTAACCGGTACTAATG
AACCGTGAGGCTTAACCTTACAACGCCGAAGCTGTTTTGGCGGATGAGAGAAGATTTTCAG
CCTGATACAGATTAAATCAGAACGCAGAAGCGGTCTGATAAAACAGAATTTGCCTGGCGGC
AGTAGCGCGGTGGTCCCACCTGACCCCATGCCGAACTCAGAAGTGAAACGCCGTAGCGC
CGATGGTAGTGTGGGGTCTCCCCATGCGAGAGTAGGGAACTGCCAGGCATCAAATAAAAC
GAAAGGCTCAGTCGAAAGACTGGGCCTTTCGTTTTATCTGTTGTTTGTCGGTGAACGCTCT
CCTGAGTAGGACAAATCCGCCGGGAGCGGATTTGAACGTTGCGAAGCAACGGCCCGGAG
GGTGGCGGGCAGGACGCCCGCCATAAACTGCCAGGCATCAAATTAAGCAGAAGGCCATC
CTGACGGATGGCCTTTTTGCGTTTCTACAAACTCTTCCTGTCGTCATATCTACAAGCCGGC
GCGCCGGGAAATGTGCGCGGAACCCCTATTTGTTTATTTTTCTAAATACATTCAAATATGTA
TCCGCTCATGAGACAATAACCCTGATAAATGCTTCAATAATATTGAAAAAGGAAGAGTATGA
GTATTCAACATTTCCGTGTCGCCCTTATTCCCTTTTTTGCGGCATTTTGCCTTCCTGTTTTTG
CTCACCCAGAAACGCTGGTGAAAGTAAAAGATGCTGAAGATCAGTTGGGTGCACGAGTGG
GTTACATCGAACTGGATCTCAACAGCGGTAAGATCCTTGAGAGTTTTCGCCCCGAAGAACG
TTTTCCAATGATGAGCACTTTTAAAGTTCTGCTATGTGGCGCGGTATTATCCCGTGTTGACG

CCGGGCAAGAGCAACTCGGTCGCCGCATACACTATTCTCAGAATGACTTGGTTGAGTACTC
ACCAGTCACAGAAAAGCATCTTACGGATGGCATGACAGTAAGAGAATTATGCAGTGCTGCA
ATAACCATGAGTGATAACACTGCGGCCAACTTACTTCTGACAACGATCGGAGGACCGAAG
GAGCTAACCGCTTTTTTGCACAACATGGGGGATCATGTAACTCGCCTTGATCGTTGGGAAC
CGGAGCTGAATGAAGCCATACCAAACGACGAGCGTGACACCACGATGCCTGCAGCAATGG
CAACAACGTTGCGCAAACTATTAACTGGCGAACTACTTACTCTAGCTTCCCGGCAACAATTA
ATAGACTGGATGGAGGCGGATAAAGTTGCAGGACCACTTCTGCGCTCGGCCCTTCCGGCT
AGCTGGTTTATTGCTGATAAATCTGGAGCCGGTGAGCGTGGGTCTCGCGGTATCATTGCA
GCACTGGGGCCAGATGGTAAGCCCTCCCGTATCGTAGTTATCTACACGACGGGGAGTCAG
GCAACTATGGATGAACGAAATAGACAGATCGCTGAGATAGGTGCCTCACTGATTAAGCATT
GGTAACTGCAGACCAAGTTTACTCATATATACTTTAGATTGATTTAAAACTTCATTTTTAATTT
AAAAGGATCTAGGTGAAGATCCTTTTTGATAATCTCATGACCAAAATCCCTTAACGTGAGTT
TTCGTTCCACTGAGCGTCAGACCCCGTAGAAAAGATCAAAGGATCTTCTTGAGATCCTTTTT
TTCTGCGCGTAATCTGCTGCTTGCAAACAAAAAAACCACCGCTACCAGCGGTGGTTTGTTT
GCCGGATCAAGAGCTACCAACTCTTTTTCCGAAGGTAACTGGCTTCAGCAGAGCGCAGATA
CCAAATACTGTCCTTCTAGTGTAGCCGTAGTTAGGCCACCACTTCAAGAACTCTGTAGCAC
CGCCTACATACCTCGCTCTGCTAATCCTGTTACCAGTGGCTGCTGCCAGTGGCGATAAGTC
GTGTCTTACCGGGTTGGACTCAAGACGATAGTTACCGGATAAGGCGCAGCGGTCGGGCTG
AACGGGGGGTTCGTGCACACAGCCCAGCTTGGAGCGAACGACCTACACCGAACTGAGATA
CCTACAGCGTGAGCTATGAGAAAGCGCCACGCTTCCCGAAGGGAGAAAGGCGGACAGGT
ATCCGGTAAGCGGCAGGGTCGGAACAGGAGAGCGCACGAGGGAGCTTCCAGGGGGAAA
CGCCTGGTATCTTTATAGTCCTGTCGGGTTTCGCCACCTCTGACTTGAGCGTCGATTTTTG
TGATGCTCGTCAGGGGGGCGGAGCCTATGGAAAAACGCCAGCAACGCGGCCTTTTTACG
GTTCCTGGCCTTTTGCTGGGCGGCCGC

pT7-rrnB- wild type:

TAATACGACTCACTATAGGGGCCGCTGAGAAAAAGCGAAGCGGCACTGCTCTTTAACAATTTATCAGACAATCTGTGTGGGCACTCGAAGATACGGATTCTTAACGTCGCAAGACGAAAAATGAATACCAAGTCTCAAGAGTGAACACGTAATTCATTACGAAGTTTAATTCTTTGAGCGTCAAACTTTTAAATTGAAGAGTTTGATCATGGCTCAGATTGAACGCTGGCGGCAGGCCTAACACATGCAAGTCGAACGGTAACAGGAAGAAGCTTGCTTCTTTGCTGACGAGTGGCGGACGGGTGAGTAATGTCTGGGAAACTGCCTGATGGAGGGGGATAACTACTGGAAACGGTAGCTAATACCGCATAACGTCGCAAGACCAAAGAGGGGGACCTTCGGGCCTCTTGCCATCGGATGTGCCCAGATGGGATTAGCTAGTAGGTGGGGTAACGGCTCACCTAGGCGACGATCCCTAGCTGGTCTGAGAGGATGACCAGCCACACTGGAACTGAGACACGGTCCAGACTCCTACGGGAGGCAGCAGTGGGGAATATTGCACAATGGGCGCAAGCCTGATGCAGCCATGCCGCGTGTATGAAGAAGGCCTTCGGGTTGTAAAGTACTTTCAGCGGGGAGGAAGGGAGTAAAGTTAATACCTTTGCTCATTGACGTTACCCGCAGAAGAAGCACCGGCTAACTCCGTGCCAGCAGCCGCGGTAATACGGAGGGTGCAAGCGTTAATCGGAATTACTGGGCGTAAAGCGCACGCAGGCGGTTTGTTAAGTCAGATGTGAAATCCCCGGGCTCAACCTGGGAACTGCATCTGATACTGGCAAGCTTGAGTCTCGTAGAGGGGGGTAGAATTCCAGGTGTAGCGGTGAAATGCGTAGAGATCTGGAGGAATACCGGTGGCGAAGGCGGCCCCCTGGACGAAGACTGACGCTCAGGTGCGAAAGCGTGGGGAGCAAACAGGATTAGATACCCTGGTAGTCCACGCCGTAAACGATGTCGACTTGGAGGTTGTGCCCTTGAGGCGTGGCTTCCGGAGCTAACGCGTTAAGTCGACCGCCTGGGGAGTACGGCCGCAAGGTTAAAACTCAAATGAATTGACGGGGGCCCGCACAAGCGGTGGAGCATGTGGTTTAATTCGATGCAACGCGAAGAACCTTACCTGGTCTTGACATCCACGGAAGTTTTCAGAGATGAGAATGTGCCTTCGGGAACCGTGAGACAGGTGCTGCATGGCTGTCGTCAGCTCGTGTTGTGAAATGTTGGGTTAAGTCCCGCAACGAGCGCAACCCTTATCCTTTGTTGCCAGCGGTCCGGCCGGGAACTCAAAGGAGACTGCCAGTGATAAACTGGAGGAAGGTGGGGATGACGTCAAGTCATCATGGCCCTTACGACCAGGGCTACACACGTGCTACAATGGCGCATACAAAGA

GAAGCGACCTCGCGAGAGCAAGCGGACCTCATAAAGTGCGTCGTAGTCCGGATTGGAGTC
TGCAACTCGACTCCATGAAGTCGGAATCGCTAGTAATCGTGGATCAGAATGCCACGGTGAA
TACGTTCCCGGGCCTTGTACACACCGCCCGTCACACCATGGGAGTGGGTTGCAAAAGAAG
TAGGTAGCTTAACCTTCGGGAGGGCGCTTACCACTTTGTGATTCATGACTGGGGTGAAGTC
GTAACAAGGTAACCGTAGGGGAACCTGCGGTTGGATCACCTCCTTACCTTAAAGAAGCGTA
CTTTGTAGTGCTCACACAGATTGTCTGATAGAAAGTGAAAAGCAAGGCGTTTACGCGTTGG
GAGTGAGGCTGAAGAGAATAAGGCCGTTCGCTTTCTATTAATGAAAGCTCACCCTACACGA
AAATATCACGCAACGCGTGATAAGCAATTTTCGTGTCCCCTTCGTCTAGAGGCCCAGGACA
CCGCCCTTTCACGGCGGTAACAGGGGTTCGAATCCCCTAGGGGACGCCACTTGCTGGTTT
GTGAGTGAAAGTCGCCGACCTTAATATCTCAAAACTCATCTTCGGGTGATGTTTGAGATATT
TGCTCTTTAAAAATCTGGATCAAGCTGAAAATTGAAACACTGAACAACGAGAGTTGTTCGTG
AGTCTCTCAAATTTTCGCAACACGATGATGAATCGAAAGAAACATCTTCGGGTTGTGAGGTT
AAGCGACTAAGCGTACACGGTGGATGCCCTGGCAGTCAGAGGCGATGAAGGACGTGCTA
ATCTGCGATAAGCGTCGGTAAGGTGATATGAACCGTTATAACCGGCGATTTCCGAATGGG
GAAACCCAGTGTGTTTCGACACACTATCATTAACTGAATCCATAGGTTAATGAGGCGAACC
GGGGGAACTGAAACATCTAAGTACCCCGAGGAAAAGAAATCAACCGAGATTCCCCCAGTA
GCGGCGAGCGAACGGGGAGCAGCCCAGAGCCTGAATCAGTGTGTGTGTTAGTGGAAGCG
TCTGGAAAGGCGCGCGATACAGGGTGACAGCCCCGTACACAAAAATGCACATGCTGTGAG
CTCGATGAGTAGGGCGGGACACGTGGTATCCTGTCTGAATATGGGGGGACCATCCTCCAA
GGCTAAATACTCCTGACTGACCGATAGTGAACCAGTACCGTGAGGGAAAGGCGAAAAGAA
CCCCGGCGAGGGGAGTGAAAAAGAACCTGAAACCGTGTACGTACAAGCAGTGGGAGCAC
GCTTAGGCGTGTGACTGCGTACCTTTTGTATAATGGGTCAGCGACTTATATTCTGTAGCAA
GGTTAACCGAATAGGGGAGCCGAAGGGAAACCGAGTCTTAACTGGGCGTTAAGTTGCAGG
GTATAGACCCGAAACCCGGTGATCTAGCCATGGGCAGGTTGAAGGTTGGGTAACACTAAC
TGGAGGACCGAACCGACTAATGTTGAAAAATTAGCGGATGACTTGTGGCTGGGGGTGAAA
GGCCAATCAAACCGGGAGATAGCTGGTTCTCCCCGAAAGCTATTTAGGTAGCGCCTCGTG

AATTCATCTCCGGGGGTAGAGCACTGTTTCGGCAAGGGGGTCATCCCGACTTACCAACCC
GATGCAAACTGCGAATACCGGAGAATGTTATCACGGGAGACACACGGCGGGTGCTAACGT
CCGTCGTGAAGAGGGAAACAACCCAGACCGCCAGCTAAGGTCCCAAAGTCATGGTTAAGT
GGGAAACGATGTGGGAAGGCCCAGACAGCCAGGATGTTGGCTTAGAAGCAGCCATCATTT
AAAGAAAGCGTAATAGCTCACTGGTCGAGTCGGCCTGCGCGGAAGATGTAACGGGGCTAA
ACCATGCACCGAAGCTGCGGCAGCGACGCTTATGCGTTGTTGGGTAGGGGAGCGTTCTGT
AAGCCTGCGAAGGTGTGCTGTGAGGCATGCTGGAGGTATCAGAAGTGCGAATGCTGACAT
AAGTAACGATAAAGCGGGTGAAAAGCCCGCTCGCCGGAAGACCAAGGGTTCCTGTCCAAC
GTTAATCGGGGCAGGGTGAGTCGACCCCTAAGGCGAGGCCGAAAGGCGTAGTCGATGGG
AAACAGGTTAATATTCCTGTACTTGGTGTTACTGCGAAGGGGGGACGGAGAAGGCTATGTT
GGCCGGGCGACGGTTGTCCCGGTTTAAGCGTGTAGGCTGGTTTTCCAGGCAAATCCGGAA
AATCAAGGCTGAGGCGTGATGACGAGGCACTACGGTGCTGAAGCAACAAATGCCCTGCTT
CCAGGAAAAGCCTCTAAGCATCAGGTAACATCAAATCGTACCCCAAACCGACACAGGTGGT
CAGGTAGAGAATACCAAGGCGCTTGAGAGAACTCGGGTGAAGGAACTAGGCAAAATGGTG
CCGTAACTTCGGGAGAAGGCACGCTGATATGTAGGTGAGGTCCCTCGCGGATGGAGCTGA
AATCAGTCGAAGATACCAGCTGGCTGCAACTGTTTATTAAAAACACAGCACTGTGCAAACA
CGAAAGTGGACGTATACGGTGTGACGCCTGCCCGGTGCCGGAAGGTTAATTGATGGGGTT
AGCGCAAGCGAAGCTCTTGATCGAAGCCCCGGTAAACGGCGGCCGTAACTATAACGGTCC
TAAGGTAGCGAAATTCCTTGTCGGGTAAGTTCCGACCTGCACGAATGGCGTAATGATGGC
CAGGCTGTCTCCACCCGAGACTCAGTGAAATTGAACTCGCTGTGAAGATGCAGTGTACCC
GCGGCAAGACGGAAAGACCCCGTGAACCTTTACTATAGCTTGACACTGAACATTGAGCCTT
GATGTGTAGGATAGGTGGGAGGCTTTGAAGTGTGGACGCCAGTCTGCATGGAGCCGACCT
TGAAATACCACCCTTTAATGTTTGATGTTCTAACGTTGACCCGTAATCCGGGTTGCGGACA
GTGTCTGGTGGGTAGTTTGACTGGGGCGGTCTCCTCCTAAAGAGTAACGGAGGAGCACGA
AGGTTGGCTAATCCTGGTCGGACATCAGGAGGTTAGTGCAATGGCATAAGCCAGCTTGAC
TGCGAGCGTGACGGCGCGAGCAGGTGCGAAAGCAGGTCATAGTGATCCGGTGGTTCTGA

ATGGAAGGGCCATCGCTCAACGGATAAAAGGTACTCCGGGGATAACAGGCTGATACCGCC
CAAGAGTTCATATCGACGGCGGTGTTTGGCACCTCGATGTCGGCTCATCACATCCTGGGG
CTGAAGTAGGTCCCAAGGGTATGGCTGTTCGCCATTTAAAGTGGTACGCGAGCTGGGTTT
AGAACGTCGTGAGACAGTTCGGTCCCTATCTGCCGTGGGCGCTGGAGAACTGAGGGGGG
CTGCTCCTAGTACGAGAGGACCGGAGTGGACGCATCACTGGTGTTCGGGTTGTCATGCCA
ATGGCACTGCCCGGTAGCTAAATGCGGAAGAGATAAGTGCTGAAAGCATCTAAGCACGAA
ACTTGCCCCGAGATGAGTTCTCCCTGACCCTTTAAGGGTCCTGAAGGAACGTTGAAGACG
ACGACGTTGATAGGCCGGGTGTGTAAGCGCAGCGATGCGTTGAGCTAACCGGTACTAATG
AACCGTGAGGCTTAACCTTACAACGCCGAAGCTGTTTTGGCGGATGAGAGAAGATTTTCAG
CCTGATACAGATTAAATCAGAACGCAGAAGCGGTCTGATAAAACAGAATTTGCCTGGCGGC
AGTAGCGCGGTGGTCCCACCTGACCCCATGCCGAACTCAGAAGTGAAACGCCGTAGCGC
CGATGGTAGTGTGGGGTCTCCCCATGCGAGAGTAGGGAACTGCCAGGCATCAAATAAAAC
GAAAGGCTCAGTCGAAAGACTGGGCCTTTCGTTTTATCTGTTGTTTGTCGGTGAACGCTCT
CCTGAGTAGGACAAATCCGCCGGGAGCGGATTTGAACGTTGCGAAGCAACGGCCCGGAG
GGTGGCGGGCAGGACGCCCGCCATAAACTGCCAGGCATCAAATTAAGCAGAAGGCCATC
CTGACGGATGGCCTTTTTGCGTTTCTACAAACTCTTCCTGTCGTCATATCTACAAGCCGGC
GCGCCAAATTGACAATTACTCATCCGGCTCGAATAATGTGTGGAACTTAAACACACACAGG
AGGAAAACATATGTCTATCCAGCACTTCCGTGTTGCGCTGATCCCGTTCTTCGCGGCGTTC
TGCCTGCCGGTTTTCGCGCACCCGGAAACCCTGGTTAAAGTTAAAGACGCGGAAGACCAG
CTGGGTGCGCGTGTTGGTTACATCGAACTGGACCTGAACTCTGGTAAAATCCTGGAATCTT
TCCGTCCGGAAGAACGTTTCCCGATGATGTCTACCTTCAAAGTTCTGCTGTGCGGTGCGGT
TCTGTCTCGTGTTGACGCGGGTCAGGAACAGCTGGGTCGTCGTATCCACTACTCTCAGAA
CGACCTGGTTGAATACTCTCCCGTTACCGAAAAACACCTGACCGACGGTATGACCGTTCGT
GAACTGTGCTCTGCGGCGATCACCATGTCTGACAACACCGCAGCGAACCTGCTGCTGACC
ACCATCGGTGGTCCGAAAGAACTGACCGCGTTCCTGCACAACATGGGCGACCACGTTACC
CGTCTGGACCGTTGGGAACCGGAACTGAACGAAGCGATCCCGAACGACGAACGTGACAC

CACCATGCCTGCGGCGATGGCGACCACCCTGCGTAAACTGCTGACCGGTGAACTGCTGAC
CCTGGCATCTCGTCAGCAGCTGATCGACTGGATGGAAGCGGACAAAGTTGCGGGTCCGCT
GCTGCGTTCTGCGCTGCCTGCGGGTTGGTTCATCGCGGACAAATCTGGTGCGGGTGAAC
GTGGTTCTCGTGGTATCATCGCGGCGCTGGGTCCGGACGGTAAACCGTCTCGTATCGTTG
TTATCTACACCACCGGTTCTCAGGCGACCATGGACGAACGTAACCGTCAGATCGCGGAAA
TCGGTGCGTCTCTGATTAAACACTGGTAAACTCACTCCTAGCCCGCCTAATAAGCGGGCTT
TTTTTCTGCAGACCAAGTTTACTCATATATACTTTAGATTGATTTAAAACTTCATTTTTAATTT
AAAAGGATCTAGGTGAAGATCCTTTTTGATAATCTCATGACCAAAATCCCTTAACGTGAGTT
TTCGTTCCACTGAGCGTCAGACCCCGTAGAAAAGATCAAAGGATCTTCTTGAGATCCTTTTT
TTCTGCGCGTAATCTGCTGCTTGCAAACAAAAAAACCACCGCTACCAGCGGTGGTTTGTTT
GCCGGATCAAGAGCTACCAACTCTTTTTCCGAAGGTAACTGGCTTCAGCAGAGCGCAGATA
CCAAATACTGTCCTTCTAGTGTAGCCGTAGTTAGGCCACCACTTCAAGAACTCTGTAGCAC
CGCCTACATACCTCGCTCTGCTAATCCTGTTACCAGTGGCTGCTGCCAGTGGCGATAAGTC
GTGTCTTACCGGGTTGGACTCAAGACGATAGTTACCGGATAAGGCGCAGCGGTCGGGCTG
AACGGGGGGTTCGTGCACACAGCCCAGCTTGGAGCGAACGACCTACACCGAACTGAGATA
CCTACAGCGTGAGCTATGAGAAAGCGCCACGCTTCCCGAAGGGAGAAAGGCGGACAGGT
ATCCGGTAAGCGGCAGGGTCGGAACAGGAGAGCGCACGAGGGAGCTTCCAGGGGGAAA
CGCCTGGTATCTTTATAGTCCTGTCGGGTTTCGCCACCTCTGACTTGAGCGTCGATTTTTG
TGATGCTCGTCAGGGGGGCGGAGCCTATGGAAAAACGCCAGCAACGCGGCCTTTTTACG
GTTCCTGGCCTTTTGCTGGT

SUPPLEMENTARY REFERENCES

1. Das, R. & Watkins, A. M. RiboDraw: semiautomated two-dimensional drawing of RNA tertiary structure diagrams. *NAR genomics Bioinforma.* 3, (2021).
2. Steinberg, S. V & Boutorine, Y. I. G-ribo: A new structural motif in ribosomal RNA. *RNA* 13, 549–554 (2007).
3. Noeske, J. *et al.* High-resolution structure of the Escherichia coli ribosome. *Nat. Struct. Mol. Biol.* 22, 336–341 (2015).
4. Cannone, J. J. *et al.* The Comparative RNA Web (CRW) Site: An online database of comparative sequence and structure information for ribosomal, intron, and other RNAs. *BMC Bioinformatics* 3, 1–31 (2002).
5. Rose, P. W. *et al.* The RCSB Protein Data Bank: Redesigned web site and web services. *Nucleic Acids Res.* 39, D392–D401 (2011).
6. Gutmanas, A. *et al.* PDBe: Protein data bank in Europe. *Nucleic Acids Res.* 42, D285-291 (2014).
7. RNAcentral Consortium. RNAcentral 2021: Secondary structure integration, improved sequence search and new member databases. *Nucleic Acids Res.* 49, D212-220 (2021).
8. RNAcentral Consortium. RNAcentral: A hub of information for non-coding RNA sequences. *Nucleic Acids Res.* 47, D221-229 (2019).
9. Goddard, T. D. *et al.* UCSF ChimeraX: Meeting modern challenges in visualization and analysis. *Protein Sci.* 27, 14–25 (2018).
10. Pettersen, E. F. *et al.* UCSF Chimera - A visualization system for exploratory research and analysis. *J. Comput. Chem.* 25, 1605–1612 (2004).
11. DeLano, W. The PyMOL Molecular Graphics System. (2008).
12. Edgar, R. C. MUSCLE: Multiple sequence alignment with high accuracy and high throughput. *Nucleic Acids Res.* 32, 1792–1797 (2004).

1

ETERNA PARTICIPANTS

Omei*, JR*, Gerry Smith*, spvincent*, DigitalEmbrace*, dl2007*, Astromon*, tone*, Willyanto*, mee2*, Eli Fisker*, jandersonlee*, Poll na gColm*, kriss888*, Analyzer*, kingboo*, salish99*, Tesla'sDisciple*, wawan151*, Jieux*, hi1000*, Malcolm*, QuantumTiger*, Brourd*, Dishmi*, nikovnikov*, TheDomBom13*, prestonzen*, rhiju*, 21shashkovn*, MasterStormer*, rxmullin*, booti386*, stratus*, SPIRALHELIX*, aparker314159*, Discovery*, MParad0x*, EcceruElme*, c-quence*, Zenith_Lord*, Korosi*, eleda77*, Norbika*, Renton_Innes*, Quez*, Arthuriel*, FurElise*, Max Goff*, whbob*, Rajab Natshah*, WaaaKen*, Dodom*, doobster101*, coolbacon134*, chemistry123*, wwei23*, Acuarion*, rnadab*, Marculius*, clollin*, AndrewKae*, Carbon Dioxide*, Clotho*, Derplord51*, cynwulf28*, wateronthemoon*, jaxman821*, philipeterna*, olpxe*, Frostuh*, rocketdog42*, atanas.atanasov*, Rivalium*, Cublex*, televisaos*, LFP6*, novice*, jyoshimi*, alx-001*, skyblue*, voyager1*, worseize*, JSci*, zahrahaghnazari*, aet36*, stevetclark*, Xnessax*, DeNa*, Bmayer47*, Mathlouk*, PixelHearts*, entreko*, idk what i'm doing8*, Benbennett1*, Alexa*, lwenger93*, Jterna*, LynnC*, jdbakermn*, pmlkjn*, 55Firehawk55*, No4b*, katakolm*, Manix*, MistressRana*, qwed117*, Drew3425*, edderiofer*, Trentis1*, IceBolt*, hoglahoo*, nfried4*, natybob*, süß-saurer-senf*, crombie78*, 21chenb*, IntuitiveNightmare*, ptrw*, averice*, rna27*, Patchapo*, unfriendyday*, AIchallenger*, suf.agent*, dfilias*, 200611736*, abdi*, BirdKing*, Pumilio*, jal*, BirbLord*, Ramlyn*, lcurtisadams*, ch.parushev@gmail.com*, Xenxc*, cparks*, mrsethbell*, 21lambertm*, NyanDoggo*, Hannibal83DK*, tommyd*, bob1029384756*, Sarc Gen*, SirMafu*, mgotrik*, cherry39*, katling*, mircea*, cool12*, lets*, RNAcoder1*, bitgamma*, ruben970105*, ahamedsc26*, ThatPerson*, netagor*, akio123*, Cat159p*, TheInverted*, Guzz*, amoyes*, temimam*, Zampa*, tronckh*, UnbiasedJazz*, SharkCrazy7*, lucagymnast*, Playingood*, Amunre*, MeowNow360*, Earthsea*, jonathan324*, cameron.turner@stargateschool.org*, NeuroDragn*, Billy Reuben*, D'Wydd*, Ahalb*, ch1ck3n*, joshuacleverley*, DogeSka*, rynomachine*, iluaee*, Dan94Sh*, BurtHarris*, syrthael*, rna-key*,

amybarish*, Iroppy*, alecpikachu*, greenmovie13*, dzynr64*, Chellow*, BugacMan*, NedyahW*, asiaa*, HarryS*

*Eterna Massive Open Laboratory, Stanford, CA 94305, USA
